# Induction of Senescence and Identification of Differentially Expressed Genes in Tomato in Response to Monoterpene

**DOI:** 10.1371/journal.pone.0076029

**Published:** 2013-09-30

**Authors:** Sumit Ghosh, Upendra Kumar Singh, Vijaykumar S. Meli, Vinay Kumar, Anil Kumar, Mohammad Irfan, Niranjan Chakraborty, Subhra Chakraborty, Asis Datta

**Affiliations:** National Institute of Plant Genome Research, New Delhi, India; CSIR-Central Drug Research Institute, India

## Abstract

Monoterpenes, which are among the major components of plant essential oils, are known for their ecological roles as well for pharmaceutical properties. Geraniol, an acyclic monoterpene induces cell cycle arrest and apoptosis/senescence in various cancer cells and plants; however, the genes involved in the process and the underlying molecular mechanisms are not well understood. In this study, we demonstrate that treatment of tomato plants with geraniol results in induction of senescence due to a substantial alteration in transcriptome. We have identified several geraniol-responsive protein encoding genes in tomato using suppression subtractive hybridization (SSH) approach. These genes comprise of various components of signal transduction, cellular metabolism, reactive oxygen species (ROS), ethylene signalling, apoptosis and DNA damage response. Upregulation of NADPH oxidase and antioxidant genes, and increase in ROS level after geraniol treatment point towards the involvement of ROS in geraniol-mediated senescence. The delayed onset of seedling death and induced expression of geraniol-responsive genes in geraniol-treated ethylene receptor mutant (*Nr*) suggest that geraniol-mediated senescence involves both ethylene dependent and independent pathways. Moreover, expression analysis during tomato ripening revealed that geraniol-responsive genes are also associated with the natural organ senescence process.

## Introduction

Plants produce a large variety of phytochemicals with specialized/secondary functions. Among these terpenes constitute the largest and most diverse class of plant specialized metabolites with more than 40,000 structural variants in nature [1]. In contrast to terpenes with primary roles in plant growth and development as pigment and hormone components, many specialized terpenes are involved in plant’s interaction with the environment [2]. A diverse array of volatile terpenes are emitted from plants to the environment for mediating chemical communications between plants and other organisms during pollination, seed dispersal and defence response, by attracting/repelling a variety of organisms [3]–[6]. The involvement of volatile terpenes in mediating plant-plant interactions has also been proposed [7]–[10].

Besides their ecological roles, plant-derived terpenes have a multitude of pharmaceutical and industrial applications as flavours, fragrances, antioxidants, anti-malarial and anti-cancer drugs etc [1]. However their availability, in most cases, is limited to the natural source, where they are synthesised and accumulated in specialized tissue types like glandular trichomes, possibly for the autotoxicity avoidance [11]. As many of them are phytotoxic, successful metabolic engineering of the terpenes not only requires an in-depth understanding of the biosynthetic pathway, but also how these compounds affect the physiology of the plants [12].

Monoterpenes by far represent the majority of the plant origin volatile terpenes. These C10-isoprenoids are synthesized in plants by various types of monoterpene synthases from geranyl diphosphate (GDP) and/or neryl diphosphate [13]. Several reports indicated that monoterpenes have the ability to play role in plant defense and apoptosis-like cell death [14]–[20]. It has been shown that plant responds to the monoterpene volatiles myrcene and ocimene by substantial changes in transcriptome [21].

Geraniol is an acyclic monoterpene alcohol emitted from the flowers of many species, notably roses [22], [23]. It is also present in vegetative tissues of many herbs and likely to be synthesized from GDP [24], [25]. Geraniol is also a key precursor of other volatile compounds such as geranial, neral, nerol, citronellol, geranyl acetate and citronellol acetate [26]. It has been noticed that geraniol induces apoptosis-like cell death, including DNA and nuclei fragmentation in cultured shoot primordia of *Matricaria chamomilla* [27]. In cultured soybean cells and shoot primordia of *M. chamomilla*, up-regulation of glutathione S- transferase (GST) and transcription factors of ethylene response element binding protein (EREBP) and WRKY families was noticed after geraniol treatment [28], [29]. Moreover, geraniol exerts *in vitro* and *in vivo* antitumor activity against murine leukemia, hepatoma, and melanoma cells [30]–[32]. Geraniol binds and inhibits the activity of 3-hydroxy-3-methylglutaryl-CoA reductase and subsequently reduces the cell growth [33]. Taken together, these reports suggest that monoterpenes, including geraniol have the ability to induce physiological changes in cancer cells and plants. However, the regulatory networks and metabolic pathways governing the cellular responses to monoterpene are not completely elucidated. Here, we have identified a large number of geraniol-responsive genes and described the geraniol-induced physiological and molecular events in tomato.

## Results and Discussion

### Geraniol and its Derivatives Induce Senescence in Tomato

**Figure 1:**
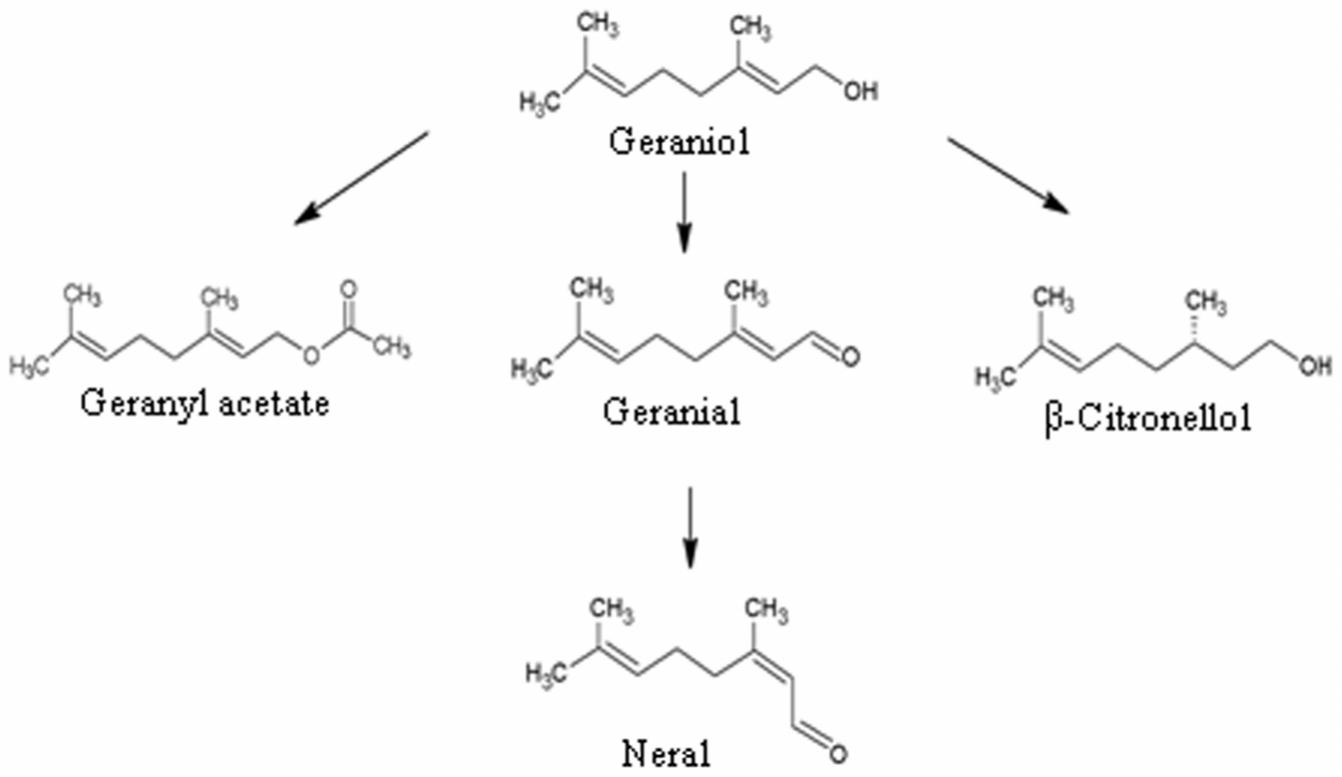
Monoterpenes used to study the plant responses. Geraniol can be metabolized into geranyl acetate, β-citronellol, geranial and neral by the action endogenous plant enzymes. The geometric isomers geranial and neral are known as *trans*-citral (citral-A) and *cis*-citral (citral-B), respectively.

**Figure 2:**
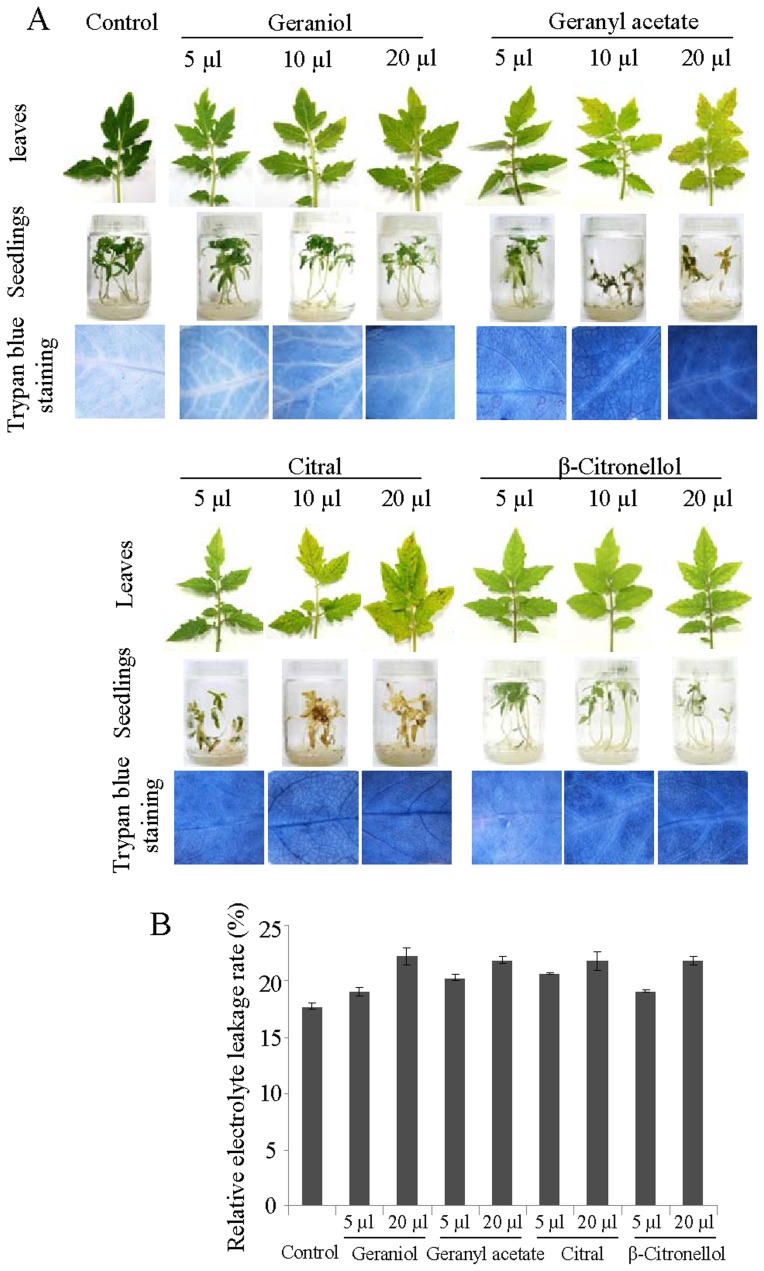
Monoterpene-induced physiological changes in tomato. (A) Development of necrosis in leaves of 45-days old tomato plants and 15-days old tomato seedlings-treated with monoterpene volatiles geraniol, geranyl acetate, citral and β-citronellol. Photographs were taken at 48 and 72 hr after volatile treatments to tomato seedlings and plants, respectively. Trypan blue staining of the leaves, taken from 48 hr monoterpene volatile-treated plants, was carried out to determine the extent of cell death. (B) The electrolyte leakage rate from the leaves was measured at 12 hr after treatment of tomato plants with monoterpene volatiles. Data are mean ± SEM (n = 3).

To know how plants respond to monoterpene, tomato plants were treated with geraniol and its derivatives geranyl acetate, citral (comprises of two geometric isomers, geranial and neral) and β-citronellol (Figure 1) [26]. For the treatment, 15-days old tomato seedlings and 45-days old plants were either exposed to monoterpene vapour or roots of the seedlings were placed into different concentrations of monoterpene solution (50 µM to 10 mM) as described in Materials and Methods. To maintain an atmosphere rich in monoterpene vapour, 45-days old potted plants were entirely covered with polypropylene bag (12 inches×16 inches) and a cotton ball (1.5 cm in diameter) containing 5–20 µl of monoterpene compound was placed inside the bag. However, monoterpene vapour treatment of seedlings was carried out by germinating seeds in 200 ml glass culture vessel (62.4 mm×95.8 mm) for 15 days and then keeping the cotton ball within the vessel with tighten cap. As shown in Figure 2A, monoterpene vapour treatment resulted in induction of senescence in both tomato seedlings and plants. This effect on seedlings/plants was found to be dose-dependent. The leaves of the seedlings/plants were green and healthy in the absence of monoterpene treatment, however, when exposed to monoterpene vapour they became chlorotic, suggesting damage of chloroplast and loss of chlorophyll. Among the monoterpenes, geranyl acetate and citral were found to exert more lethal effect to seedlings/plants compared to geraniol and β-citronellol. Trypan blue staining and the measurement of electrolyte leakage rate were used as the indicators of cell death during senescence in monoterpene-treated plants. Leaves of the monoterpene-treated plants acquired more intense staining and showed higher rate of electrolyte leakage in comparison to control plants (Figure 2A, B), which is an indicator of cell membrane damage.

**Figure 3:**
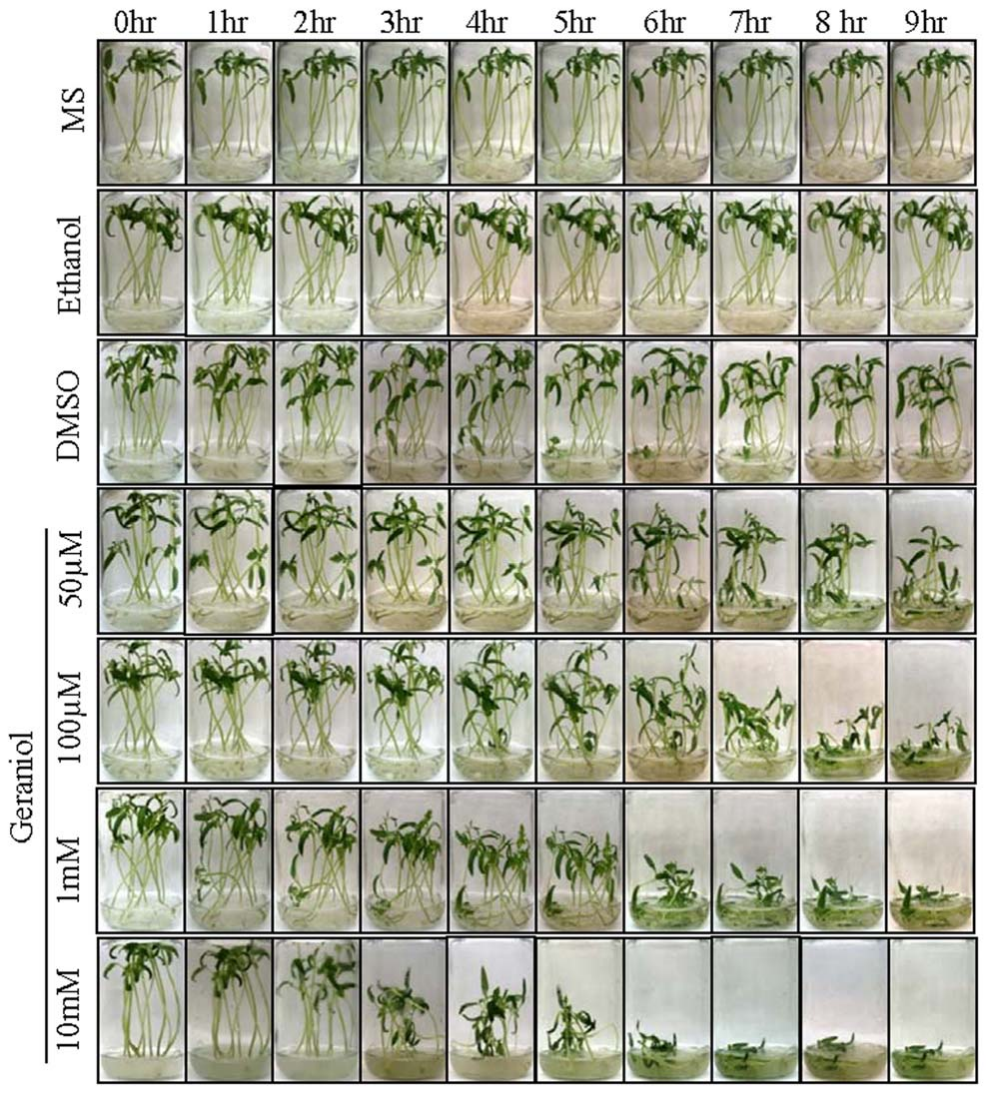
Phytotoxic effect of geraniol on tomato seedlings. Seedlings were fed with MS medium containing 50 µM to 10 mM geraniol, 20% DMSO and 100 mM ethanol. Seedlings treated with geraniol showed rapid loss of vigor and viability.

The exposure of roots to different concentration of monoterpene caused phytotoxicity in seedlings (Figure 3, S1). In the presence of 10 mM geraniol, seedlings started losing vigor and viability within 2 hr of incubation and the effect was aggravated thereafter; whereas at low concentrations delayed effect was noticed (Figure 3). Seedlings were also treated with 100 mM ethanol to know whether the cell death/senescence was due to the dehydration properties of alcohol, since geraniol is a monoterpene alcohol. However, ethanol-treated seedlings showed no visible changes; thus, the loss of seedlings vigor after geraniol treatment may be because of its cell death/senescence-inducing properties. Similar to geraniol, its derivatives were also phytotoxic to seedlings (Figure S1). In contrast to the vapour treatments where variable degree of sensitivity to different monoterpenes was exhibited by seedlings/plants (Figure 2), the effects of treatment with different monoterpenes solution were more or less comparable with slight early seedling death recorded in geraniol treatment compared to other monoterpenes (Figure 3). This may be due to the differential penetration of monoterpene vapours through the leaf surface. Altogether, these observations indicate that geraniol and its derivatives are the inducer of cell death/senescence.

### Cloning and Expression Profiling of Geraniol Responsive Transcripts

To eliminate damaged or unwanted cells, plants undergo highly regulated cell death process which may result in tissue/organ senescence [34]. Programmed cell death is critical for plant growth and development as well as response to environmental stresses. In contrast to animal, the regulatory and molecular mechanisms of programmed cell death are not well understood in plants. The knowledge gained from the monoterpene induced cell death/senescence may be applied to some extent to understand the natural programmed cell death process at the molecular level.

**Figure 4:**
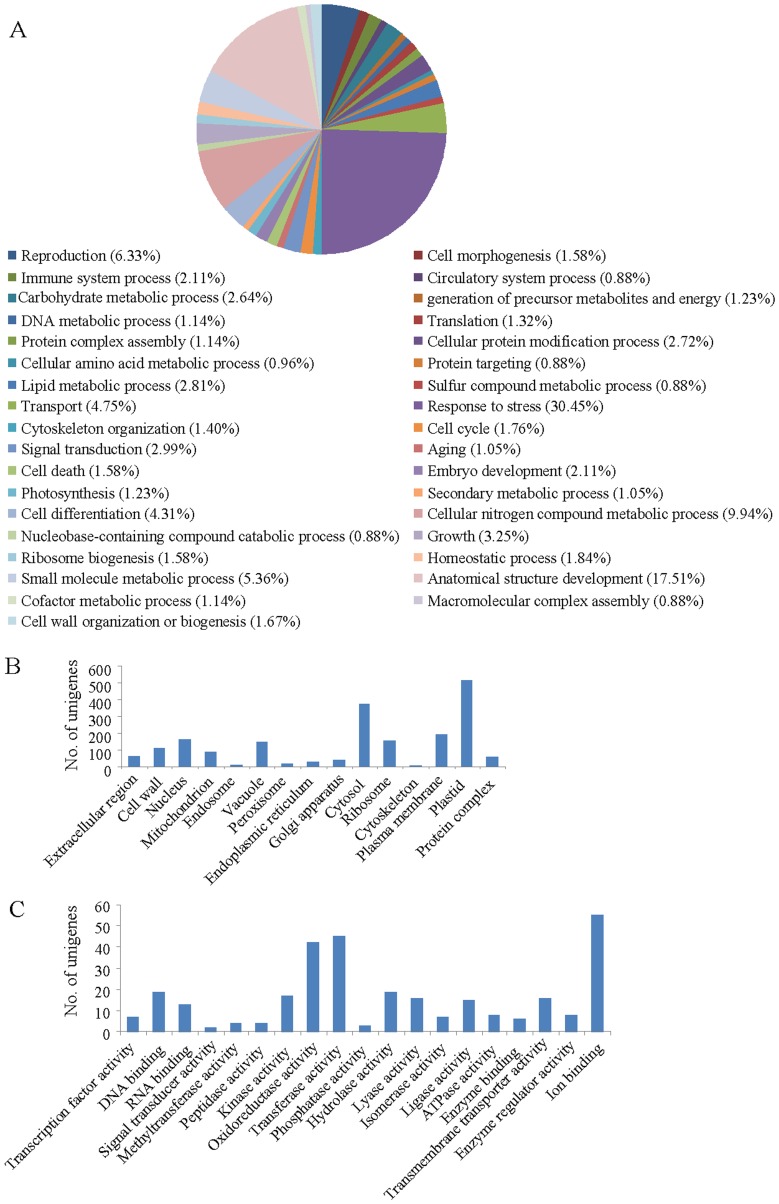
Functional categorization of geraniol-responsive transcripts based on Gene Ontology (GO) biological process (A), cellular component (B) and molecular function (C).

Backed by many observations, identification of geraniol-responsive genes became imperative to gain insights into geraniol-induced molecular responses in plants. Expressed sequence tags (ESTs) have developed into a powerful tool for the identification of the differentially expressed genes [35], [36]. To clone the tomato transcripts expressed in response to geraniol treatment, suppression subtractive hybridization (SSH) technology was used because of its high efficiency in enriching low expressing genes and normalization of targeted fragments. SSH library was generated from tomato seedlings treated with 10 mM geraniol for 30 min vs. control (20% DMSO treatment for 30 min). Although, geraniol treatment in both solution and vapour form caused visible changes in seedlings (Figure 2, 3), former method was preferred for the construction of SSH library because of the quick response of the seedlings to the geraniol treatment in solution compared to vapour form. The treatment with 10 mM geraniol resulted visible changes on seedlings vigour at ∼90 min of exposure. Therefore, transcript analysis was carried out with seedlings exposed to geraniol for 30 min to eliminate the undesired effect of general toxicity response. Both control and geraniol-treated seedlings looked normal after 30 min of exposure with geraniol. This particular experimental approach enabled us to target the transcripts which are geraniol-responsive. A total of 1765 individual geraniol-responsive ESTs were cloned and the sequences were deposited in the GenBank database (Table S1). Analysis of the ESTs revealed a total of 1136 unigenes: 778 singletons and 358 contigs. Similarity search (BLASTX) against the tomato genome database [ITAG release 2.3 predicted proteins (SL2.40); www.solgenomics.net] identified 892 protein encoding genes (Table S2). The unigene sequences were also functionally categorized based on the biological process, cellular component and molecular function (Figure 4A–C) using a Gene Ontology (GO) scheme (www.agbase.msstate.edu). Majority of the transcripts were predicted to be associated with plastid (45.15%), cytosol (33.01%), plasma membrane (16.9%), nucleus (13.99%), ribosome (13.73%) and vacuole (13.02%); suggesting that these cellular compartments play important role in mediating geraniol-induced cell death/senescence. Some of the gene families upregulated by geraniol include the *heat shock proteins* (*HSPs*), *chaperonins*, *14-3-3 proteins*, *pectinesterases*, *peroxidases*, *thioredoxins*, *aquaporins*, *expansins, NAM-like proteins, C2H2-type zinc finger proteins, ERF and WRKY family transcription factors*.

**Figure 5:**
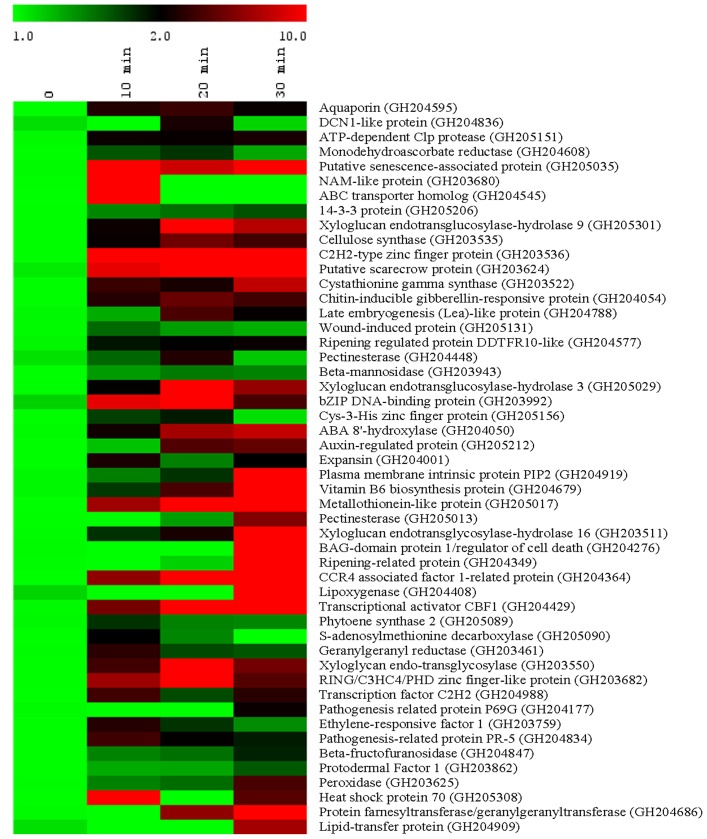
Expression profiles of geraniol-responsive transcripts. Tomato seedlings were treated with 10-PCR analysis was carried out for 50 genes selected from SSH library. Data are mean (n = 3). Tomato actin was used as endogenous control.

In order to confirm geraniol-induced expression of the transcripts, 50 unigenes which are senescence and/or stress-related were selected from the SSH library for qRT-PCR analysis (Figure 5). Tomato seedlings were treated with 10 mM geraniol for 10, 20 and 30 min and cDNAs were prepared for qRT-PCR analysis. Among the 50 unigenes, 36 exhibited more than 3- fold higher relative expression after geraniol treatment. Rest of the unigenes (14) showed at least 1.5- fold up-regulation in geraniol-treated seedlings. Few early responsive genes showed high level of expression within 10 min of geraniol treatment. Some of them belong to transcriptional regulators e.g. *NAM-like protein*, *C2H2-type zinc finger protein*, *scarecrow-like protein*, *bZIP DNA binding protein, transcriptional activator CBF1* and *transcription factor C_2_H_2_*. Functional characterization of these geraniol-responsive genes will lead to a better understanding of the underlying molecular mechanism of cell death/senescence process.

### Geraniol-responsive Metabolic and Regulatory Genes

A number of geraniol-responsive genes are related to glycolysis and tricarboxylic acid (TCA) cycle (Table S2). These are *fructose 1,6 bisphosphate aldolases*, *glyceraldehyde-3-phosphate dehydrogenases*, *triose-phosphate isomerases*, *isocitrate lyase* and *succinate dehydrogenase*. During cell death/senescence a high rate of respiration may be required for the continuous supply of energy to sustain the degradation of cellular structure. Up-regulation of respiration related genes during natural senescence process is also known [37]–[39]. Several metabolic genes and transporters were also present in the SSH library (Table S2). These include *glutamate dehydrogenase*, *alcohol dehydrogenases*, *lipoxygenase*, *phytoene synthase 2*, *S-adenosylmethionine decarboxylases*, *carboxylesterase*, *aquaporins*, *ABC transporter* and protein transport *SEC13*-like protein. Previous reports also suggested that these genes are involved in senescence-related process [40]–[46].

Some candidate geraniol-responsive regulatory genes are *mitogen-activated protein kinase* kinase kinase (*MAP3K*), *mitogen-activated protein kinase* (*MPK3*), *CBL-interacting serine/threonine-protein kinase* (*CIPK*), *C2H2-type zinc finger proteins*, *NAM-like proteins*, *regulatory associated protein of TOR* (*RAPTOR*), *extra-large G-protein*, *XLG*, GRAS family transcription factors such as *scarecrow-like protein* and *GRAS4*, *bZIP, WRKY and ERF family transcription factors*, *transcriptional activator CBF1, CCR4 associated factor 1-related protein*, *14-3-3 proteins* and calmodulin-binding protein similar to *ATCAMBP25* (Table S2). Homologs of these regulatory genes are known to play important role in plant growth and development, stress response, cell death and senescence by serving as the signal transduction components and/or by controlling transcription and translation [39], [47]–[57]. These data suggest that geraniol treatment activates plasma membrane transport function, MAP kinase signalling cascade and several transcription factors. These ultimately lead to changes in cellular metabolism required for the progression and completion of cell death/senescence.

### Effects of Geraniol on Protein Fate and Cell Wall Metabolism

Post-translational modifications and folding of proteins and the regulated degradation process play crucial role in mediating cellular responses to various developmental and environmental changes. Previously, the expression of heat shock proteins (HSPs) and chaperonins which generally participate in protein folding has been correlated with the natural senescence process [37], [39]. The identification of several HSPs and chaperonins in the SSH library suggests that the protection of some proteins is necessary for the progression of geraniol-induced cell death/senescence (Table S2). The presence of some transcripts related to 26S proteasomal protein degradation such as, 26S proteasome subunits alpha 6, alpha 7, RPN5b, AAA-ATPase subunit RPT4a and ubiquitin-conjugating enzyme 9 was also noticed in SSH library (Table S2). 26S proteasome is the eukaryotic protein degradation system responsible for the protein turnover, which plays an important role in the precise removal of short-lived regulatory proteins such as transcription factors [58]. Thus, it could be speculated that the activation of 26S proteasome pathway is required during geraniol-induced cell death/senescence. Few proteases such as, subtilisin-like proteases, pre-pro-cysteine proteinase, serine carboxypeptidase, serine-type endopeptidase, aminopeptidase, serine protease and aspartic-type endopeptidases were found to be geraniol-responsive (Table S2). Proteases are implicated in various plant processes including senescence-associated cell death, protein turnover during biotic and abiotic stresses and storage protein utilization during seed germination [59], [60]. During senescence, these proteases may be associated with the turnover and remobilization of cellular materials out of the senescing tissues to the actively growing tissues. Geraniol-induced expression of transcriptional regulators and genes involved in protein modifications and degradation points towards the existence of both transcriptional and post-transcriptional regulation of cell death/senescence process.

Cell wall of plants undergoes precise changes in metabolism during growth and development and senescence-related processes. Some geraniol-responsive genes which are related to cell wall metabolism are *polygalacturonase*, *xyloglucan endotransglucosylase-hydrolases*, *β-D-glucan exohydrolase*, *endo-1,4-β-glucanases*, *β-galactosidase*, *glucan endo-1,3-β-glucosidases*, *pectin methylesterases*, *β-fructofuranosidases*, pectinesterases, *β-mannosidase* and *expansions* (Table S2). These data suggest that cell wall cellulose, hemicellulose and pectin polysaccharides are targeted for degradation during geraniol-induced cell death/senescence.

### Role of Reactive Oxygen Species and Ethylene in Geraniol Mediated Senescence

**Figure 6:**
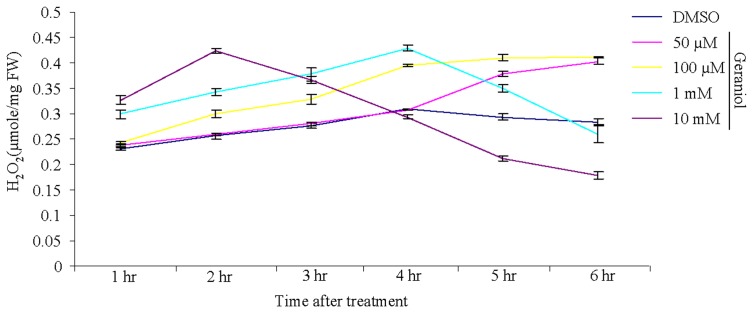
H_2_O_2_ concentration measured in tomato seedlings after treatment with geraniol. Seedlings were placed in MS media containing geraniol (50 µM to 10 mM) or 20% DMSO (control). Data are mean ± SEM (n = 3).

Cell death/senescence processes are oxidative phenomena involving ROS such as, H_2_O_2_ and superoxide anion [34], [61]. Geraniol-treated seedlings accumulated elevated level of H_2_O_2_ as compared to control seedlings, suggesting the involvement of ROS in geraniol-mediated cell death/senescence process (Figure 6). Geraniol-induced expression of the ROS synthesizing enzyme NADPH oxidase (RBOHC) may be a mechanism to build up oxidative burst during geraniol-induced cell death/senescence (Table S2). ROS scavenging enzymes and antioxidants which are the early markers of oxidative stress were also geraniol inducible: peroxidases, ascorbate peroxidase, glutathione peroxidases, GST, catalases, Mn superoxide dismutase, thioredoxins and peroxiredoxin were identified in SSH library (Table S2). ROS can promote the oxidative deterioration process in such a way that it affects the cellular functions required for the cell death/senescence. Thus, cellular antioxidant activities may be important to keep the ROS level under control.

Apoptosis-like cell death and DNA damage response are the events of plant senescence process which is regulated by ethylene [62], [63]. *BAG-domain protein 1/regulator of cell death*, *apoptosis inhibitory protein 5* (*API5*) and *putative senescence-associated protein* were found to be geraniol-responsive (Table S2). These genes were previously linked with apoptosis-like cell death and senescence [64]–[66]. Moreover, genes which are associated with DNA-damage repair/tolerance were identified in the SSH library (Table S2). These are *DNA-DAMAGE REPAIR/TOLERATION 100,* (*DRT100*) and *DNA photolyase*. Genes which are involved in the synthesis of methionine (*methionine synthase* and *cystathionine gamma-synthase*) and S-adenosylmethionine (*S-adenosylmethionine synthase*), the precursors of ethylene, were also found to be geraniol-responsive (Table S2). Furthermore, ethylene-regulated genes such as *ethylene insensitive 3 (EIN3)-like protein*, *ethylene response factors* (*ERFs*) were identified to be geraniol-responsive (Table S2). Hence, geraniol-responsive expression of ethylene-related genes suggests the involvement of ethylene in geraniol induced cell death/senescence. Therefore, the involvement of ethylene was tested by analysing the geraniol-induced cell death/senescence in an ethylene receptor mutant (*Never ripe*, *Nr*) which is known to have defect in ethylene perception [67]. Delayed onset of seedling death in *Nr* mutant seedlings as compared to wild type confirmed the role of ethylene (Figure S2).

### Geraniol Acts in Ethylene Dependent and Independent Ways

**Figure 7:**
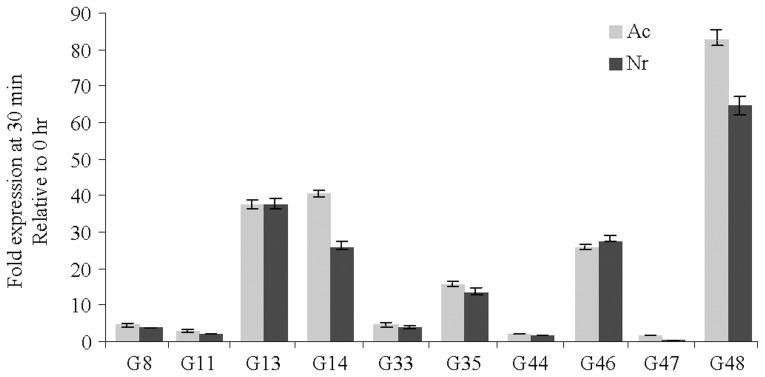
Induced expression of geraniol-responsive genes in ethylene receptor mutant (*Nr*) seedlings after geraniol treatment. Transcript level of geraniol-responsive genes at 30 min of treatment with 10 mM geraniol, relative to 0 hr. Data are mean ± SEM (n = 3). *G8- NAM-like protein* (GH203680), *G11- xyloglucan endotransglucosylase-hydrolase* (GH205301), *G13- C2H2-type zinc finger protein* (GH203536), *G14- putative scarecrow protein* (GH204307), *G33- bZIP DNA-binding protein* (GH203992), *G35- ABA 8′-hydroxylase* (GH204050), *G44- BAG-domain protein 1/regulator of cell death* (GH204276), *G46- CCR4 associated factor 1-related protein* (GH204364), *G47- lipoxygenase* (GH204408), *G48- transcriptional activator CBF1* (GH204429).

**Figure 8:**
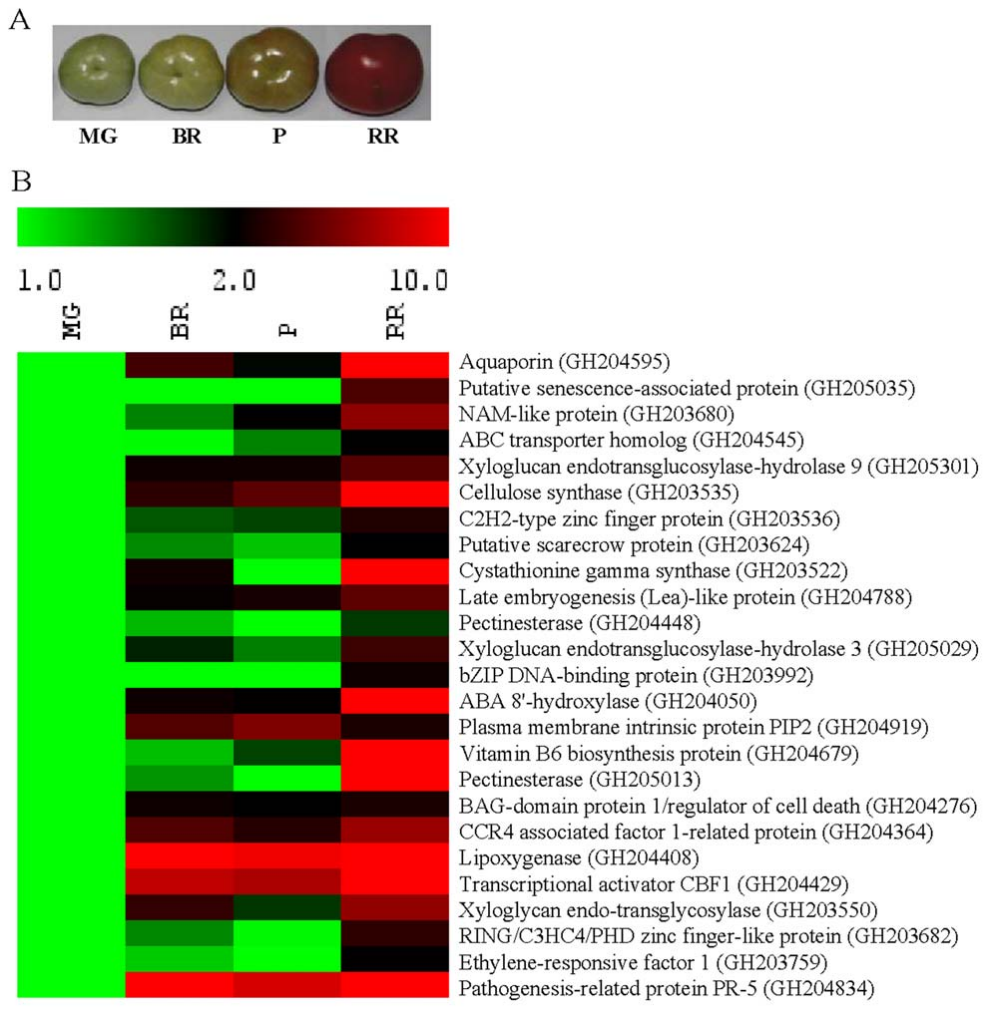
Up-regulation of geraniol-responsive genes at the onset of fruit senescence. (A) Tomato fruits used in this study: mature green (MG), breaker (BR), pink (P) and red ripe (RR). (B) Transcript level of 25 geraniol-responsive genes was determined in different ripening stages of fruits by qRT-PCR analysis. Tomato actin was used as endogenous control. Data are mean (n = 3).

The geraniol-responsive expression of S-adenosylmethionine biosynthesis and ethylene signalling genes (Table S2) and delayed onset of cell death/senescence in *Nr* mutant seedlings (Figure S2) led us to examine the expression of geraniol-regulated genes in *Nr*. Expression analysis, in this mutant revealed that the transcript level of some genes was elevated after the geraniol treatment (Figure 7). Although ethylene signalling genes were upregulated in wild type seedlings after geraniol treatment (Table S2), the geraniol-induced expression of these genes in ethylene receptor mutant seedlings suggests that the effect of geraniol in cell death/senescence may not be completely dependent on ethylene. These observations point towards the existence of both ethylene dependent and independent pathways for the geraniol-induced cell death/senescence process. Ethylene independent pathway may comprise of genes which show induced expression during natural senescence process (Figure 8); however, their geraniol-regulation was mostly unaffected by *Nr* mutation (Figure 7). These genes include *NAM-like protein*, *C2H2-type zinc finger protein*, *putative scarecrow protein*, *BAG-domain protein 1/regulator of cell death*, *CCR4 associated factor 1-related protein*, *bZIP DNA-binding protein* and *transcriptional activator CBF1*.

### Geraniol-regulated Genes Exhibit Induced Expression at the Onset of Natural Organ Senescence

We next tested whether the genes identified to be geraniol-responsive are also involved in natural senescence of plant organs. Fruit senescence process has received considerable experimental attention to identify the ways to optimize the fruit quality. Based on the putative function of the geraniol-responsive genes, an overlapping gene expression pattern between fruit senescence and geraniol-induced cell death/senescence could be predicted. Several geraniol-responsive transcripts which belong to regulatory components, cell wall modification, metabolism, transport and ethylene response could be associated with the fruit senescence during ripening process (Figure 4; Table S2). In order to know whether geraniol-responsive genes are associated with natural organ senescence, their expression during tomato fruit ripening was determined by qRT-PCR analysis (Figure 8A, B). For this, geraniol-regulated genes (Figure 5) whose role in fruit ripening is unknown to date were selected for the analysis, alongwith some ripening-related genes as reference. Ripening-specific expression of transcriptional regulators such as, *NAM-like protein*, *C2H2-type zinc finger protein*, GRAS family *scarecrow-like protein*, *bZIP DNA binding protein* and *transcriptional activator CBF1* was noticed. Some of the other genes which showed induced expression during tomato ripening include *ABA 8′ hydroxylase, pathogenesis-related protein PR5*, few transporters (*aquaporin*, *ABC transporter* and *plasma membrane intrinsic protein PIP2*) and regulator of cell death (*BAG-domain protein 1*) and mRNA stability (*CCR4 associated factor 1*). Taken together, significant overlap in gene expression suggests that the geraniol-induced cell death/senescence and fruit ripening associated senescence may share some common regulators.

### Conclusions

Monoterpenes are among the most commonly produced volatile secondary metabolites in plants which elicit responses in a wide range of organisms. Some monoterpenes are also emerging as promising therapeutic agents. Although, geraniol was previously associated with apoptosis-like cell death/senescence in plants and cancer cell lines, a comprehensive analysis of the geraniol-responsive transcripts has not been reported till date. Therefore, we took the advantage of SSH approach and identified 892 candidate geraniol-responsive genes in tomato. The involvement of ROS and ethylene during the geraniol-mediated cell death/senescence has been proposed. The data also suggests that continuous demand for energy during senescence process may be met through upregulation of glycolysis and tricarboxylic acid (TCA) pathway. The geraniol induced senescence is possibly regulated at the transcriptional as well as post-transcriptional levels because differential expression of several transcriptional regulators and protein modifications-related genes was observed. The data presented here also indicates significant overlap in gene expression associated with geraniol-induced cell death/senescence and fruit ripening associated senescence. A number of ripening-specific and geraniol-responsive novel genes have been identified. Functional characterization of such genes will be of particular interest to explore some previously unknown regulators of fruit ripening and/or senescence. Taken together, these results advance our understanding of cell death/senescence process and suggest that geraniol can serve as inducer of cell death/senescence.

## Materials and Methods

### Plant Materials and Growth Conditions

Tomato (cv. Pusa Ruby) seeds were obtained from the National Seeds Corporation Ltd., New Delhi. *Nr* mutant used in the study was procured from the Tomato Genetics Resource Center, University of California at Davis and was in Ailsa Craig background. Seeds were germinated in pre-sterilized soil and later transplanted in pots containing soil, agropeat and vermiculite (2∶1∶1). Plants were grown in a growth chamber with ∼25/22°C day/night temperature, ∼65% relative humidity and 16/8 hr light/dark regimen. For the analysis, fruits were harvested at the mature green (MG), breaker (BR), pink (P) and red ripe (RR) stages after tagging the flowers at anthesis. Fruits after ∼40 days of anthesis were considered mature green (the surface of the tomato was completely green, the shade of the colour varied from light to dark), MG+4 days as breaker stage, B+2 days as pink and P+3 days as red ripe stage.

### Monoterpene Vapour Treatment

For monoterpene vapour treatment, tomato plants (45-days old) grown under controlled condition of the growth chamber (as mentioned above) were entirely closed in polypropylene bag (12 inches×16 inches) containing different amounts (5 µl, 10 µl and 20 µl) of monoterpenes (geraniol, citral, geranyl acetate and β-citronellol) shocked in equal size (1.5 cm in diameter) of cotton balls (Figure S3A). Second leaf was taken for photograph, trypan blue staining and electrolyte leakage analysis. For treatment of seedling, seeds were germinated in 200 ml glass culture vessels (62.4 mm×95.8 mm) with MS media and 15-days old seedling were treated with monoterpene vapour by placing cotton ball shocked in monoterpene solution within the vessel and tightening with the cap (Figure S3B). The monoterpenes obtained from Sigma-Aldrich were of high purity: geraniol (98%), citral (95%), geranyl acetate (98%) and β-citronellol (99%).

### Monoterpene Treatment in Solution

Fifteen days old tomato seedlings, germinated and grown in MS medium (pH 5.7±0.1) were used for geraniol, geranyl acetate, citral and β-citronellol (Sigma-Aldrich) treatments. Seedlings were transferred to liquid MS media containing 50 µM to 10 mM monoterpene in 20% dimethyl sulfoxide (DMSO). As these monoterpenes are not soluble in water, 20% DMSO was used as solvent to achieve a uniform solution. Control experiment was carried out by transferring seedlings to liquid MS media with/without 20% DMSO or 100 mM ethanol. Seedling placed on MS media with/without 100 mM ethanol looked normal even after 24 hours of incubation. However, seedlings started deteriorating if incubated for longer time (>12 hrs) with MS media containing 20% DMSO. Thus, suppression subtractive hybridization and gene expression experiments were carried out with 10 mM geraniol and treatment was completed within 30 minutes to avoid any undesired effect of DMSO. After the specified time of treatment, seedlings were harvested, frozen immediately in liquid nitrogen and stored at −80°C.

### Trypan Blue Staining

Cell death was estimated by trypan blue staining. Leaves from monoterpene-treated plants were harvested and immediately submerged in lactic acid-glycerol-phenol-trypan blue solution (0.25 mg/ml trypan blue in H_2_O) prepared in 1∶1∶1∶1 ratio. Then samples were heated over boiling water for 2 min and left at room temperature over night. After multiple exchanges of destaining solution (lactic acid-glycerol-phenol-water in 1∶1∶1∶1 ratio), the samples were visualized using a multizoom microscope (Nikon AZ100).

### Determination of Electrolyte Leakage Rate

Electrolyte leakage was assayed by estimating the ion leaching from the leaves into ultra pure MilliQ water. Leaves discs of equal dimension (10 mm) and number (4) were prepared from the second leaf of monoterpene-treated plants and placed into 10 ml of MilliQ water in two sets. The first set was kept at room temperature for 2 h and its conductivity (C1) was recorded using a conductivity meter. The second set was autoclaved and its conductivity (C2) was recorded and relative electrolyte leakage [(C1/C2)x 100] was calculated. The experiments were carried out in triplicate.

### Construction of Subtraction cDNA Library

SSH library was generated according to the instruction manual of PCR-Select cDNA Substraction kit (Clontech). PolyA+ RNA was isolated from total RNA using Dynabeads mRNA purification kit (Invitrogen) and 2 µg of purified polyA+ RNA was reverse transcribed using AMV reverse transcriptase. In order to produce a cDNA library representative of geraniol-induced genes, double-stranded cDNA sample from tomato seedlings-treated with 10 mM geraniol in 20% DMSO for 30 min was used as the tester while seedlings-treated with 20% DMSO for 30 min was used as the driver. Both tester and driver cDNAs were digested with RsaI for producing blunt ends cDNAs. Two population of adapter ligated tester cDNA sample were prepared and two rounds of hybridization between driver and adaptor linked tester were performed to remove common or non-induced sequences. Tester specific cDNAs were then PCR amplified (Advantage 2 PCR kit, Clontech). PCR products were ligated into the pGEM-T Easy vector (Promega) and transformed into *E. coli* (DH5α) for propagation. Recombinant clones were selected based on blue-white selection on LB agar plate containing 50 µg/ml ampicillin and grown in 96-well plates with 2X YT media at 37°C for plasmid isolation and glycerol stock preparation.

### Sequencing and Analysis of ESTs

In order to obtain the sequence of ESTs, plasmid DNAs were isolated from the individual recombinant clones following the Perfectprep Plasmid 96 Vac kit according to the manufacturer’s instructions (5PRIME). Purified plasmid DNAs were analyzed on 0.8% agarose gel before going for sequencing. Plasmid DNAs were single-pass sequenced using the Big Dye terminator kit (Applied Biosystems, CA) with M13 forward and reverse primers, in a ABI Prism 3700 DNA analyzer (Applied biosystems, CA). A total of 2300 independent recombinant clones were randomly picked and after single pass sequencing, 1987 individual sequences were obtained. Among these, 222 sequences were discarded due to low quality, short sequence length (<100 bp), organelle source and vector sequence. Remaining, 1765 high quality EST sequences (Table S1) were assembled into contigs [68]. Functional annotation of the unigenes (contigs and singletons) was carried by homology search against tomato genome database (www.solgenomics.net), non-redundant protein database of NCBI using BLASTX program (www.ncbi.nlm.nih.gov) and by following the Gene Ontology scheme (www.agbase.msstate.edu).

### RNA Isolation and Quantitative RT-PCR

RNA was isolated according to the LiCl precipitation method [69] and purified using RNeasy Mini Kit (Qiagen). Three different tomato fruits or ten seedlings were taken for each group. RNA quality was checked by running 1.2% agarose gel and by determining the A260/280 ratio. Five microgram of total RNAs, quantified using a nanodrop (ND 1000, Thermo Scientific) were reverse transcribed using superscript II (Invitrogen) at 42°C for 50 min in 20 µl reaction volume following the manufacture’s instructions. cDNAs were diluted three times before using in the quantitative real-time PCR reaction (10 µl reaction volume) containing 5 µl 2X SYBR green mixture (Applied Biosystems), 1.5 µl of diluted cDNA and 500 nM of each forward and reverse gene specific primers. Quantitative RT-PCR was performed using One Step Real Time RT-PCR (step one v1.0) or 7900 HT Fast Real Time PCR (SDS 2.3/RQ Manager 1.2) of Applied Biosystems. Gene specific primers were designed using Primer Express version 3.0 (Applied Biosystems). List of genes and oligonucleotide primers used are shown in Table S3. Melting curves were analyzed at the dissociation step to examine the specificity of amplification. Relative gene expression was analyzed using the 2^−ΔΔCq^ method.

### Determination of H_2_O_2_ Level

H_2_O_2_ was extracted and quantified from tomato seedlings as described previously [70]. In brief, tissue was homogenised in 3 ml of ice cold 0.01 M phosphate buffer (pH 7.0) and homogenate was centrifuged at 15000 rpm for 15 min at 4°C. Further, 500 µl of the clear homogenate was added to 1.5 ml of 0.01 M phosphate buffer (pH 7.0). Then, 2 ml of 5% potassium dichromate and glacial acetic acid (1∶3, v/v) was added to the mixture. The absorbance was read at 570 nm against the reagent blank without sample extract in a Shimadzu UV 2550 uv viz spectrophotometer. The quantity of H_2_O_2_ was determined based on a standard curved (Y = 0.004X, R^2^ = 0.9972) generated using 10 to 100 µmol of H_2_O_2_.

## Supporting Information

Figure S1 **Effect of citral, geranyl acetate and β-citronellol treatment on seedling vigor of wild type tomato Pusa Ruby.**
(TIF)

Figure S2 **Effect of gerainol treatment on seedling vigor of wild type tomato Ailsa Craig (A) and Never Ripe (** ***Nr*** **) mutant (B).** Delayed seedling death was observed in *Nr* mutant as compared to wild type.
(TIF)

Figure S3 **Closed environment used for the treatment of 45-days old tomato plantlets (A) and 15-days old seedlings (B).**
(TIF)

Table S1 **GenBank accession numbers of ESTs.**
(DOC)

Table S2 **List of geraniol-responsive tomato unigenes (contigs and singletons).**
(DOC)

Table S3 **List of primers used for quantitative RT-PCR analysis.**
(DOC)
