# Supplementary figures and images for: Induction of Senescence and Identification of Differentially Expressed Genes in Tomato in Response to Monoterpene
Source: PLoS One. 2013 Sep 30;8(9):e76029. doi: 10.1371/journal.pone.0076029 (PMC3786903; doi:10.1371/journal.pone.0076029)

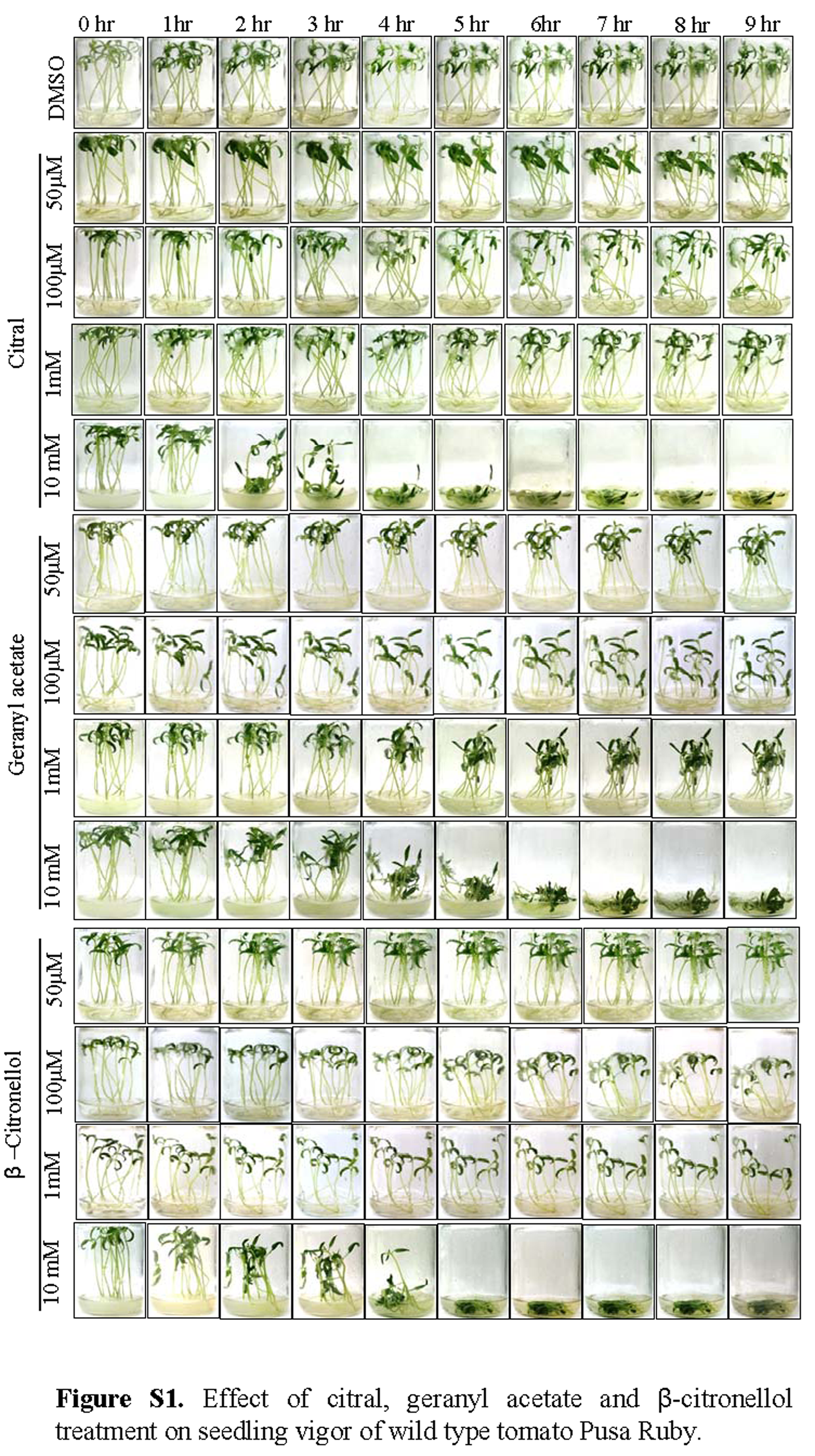

Supplement: Figure S1 — Effect of citral, geranyl acetate and β-citronellol treatment on seedling vigor of wild type tomato Pusa Ruby. (TIF) [file pone.0076029.s001.tif]

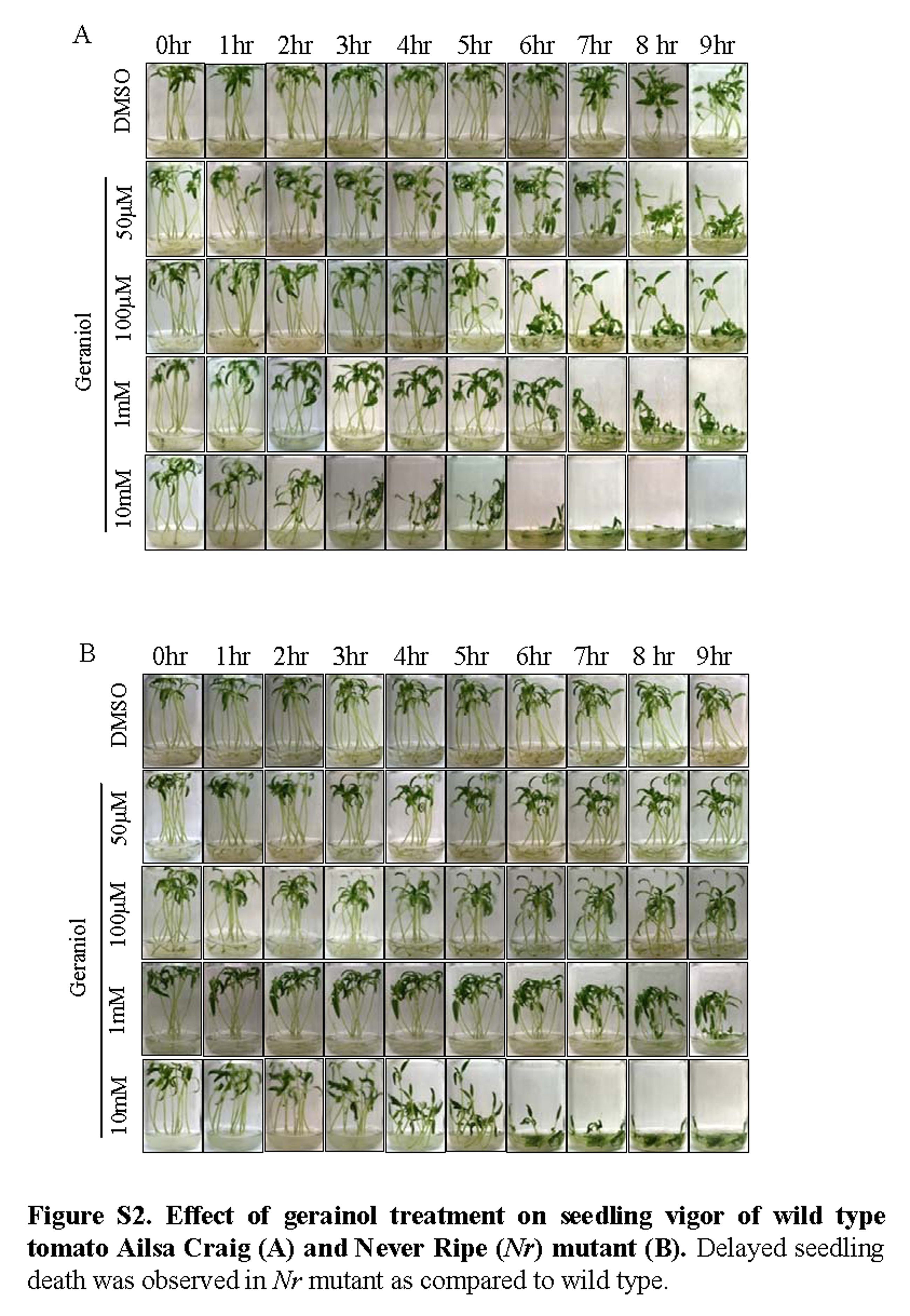

Supplement: Figure S2 — Effect of gerainol treatment on seedling vigor of wild type tomato Ailsa Craig (A) and Never Ripe ( Nr ) mutant (B). Delayed seedling death was observed in Nr mutant as compared to wild type. (TIF) [file pone.0076029.s002.tif]

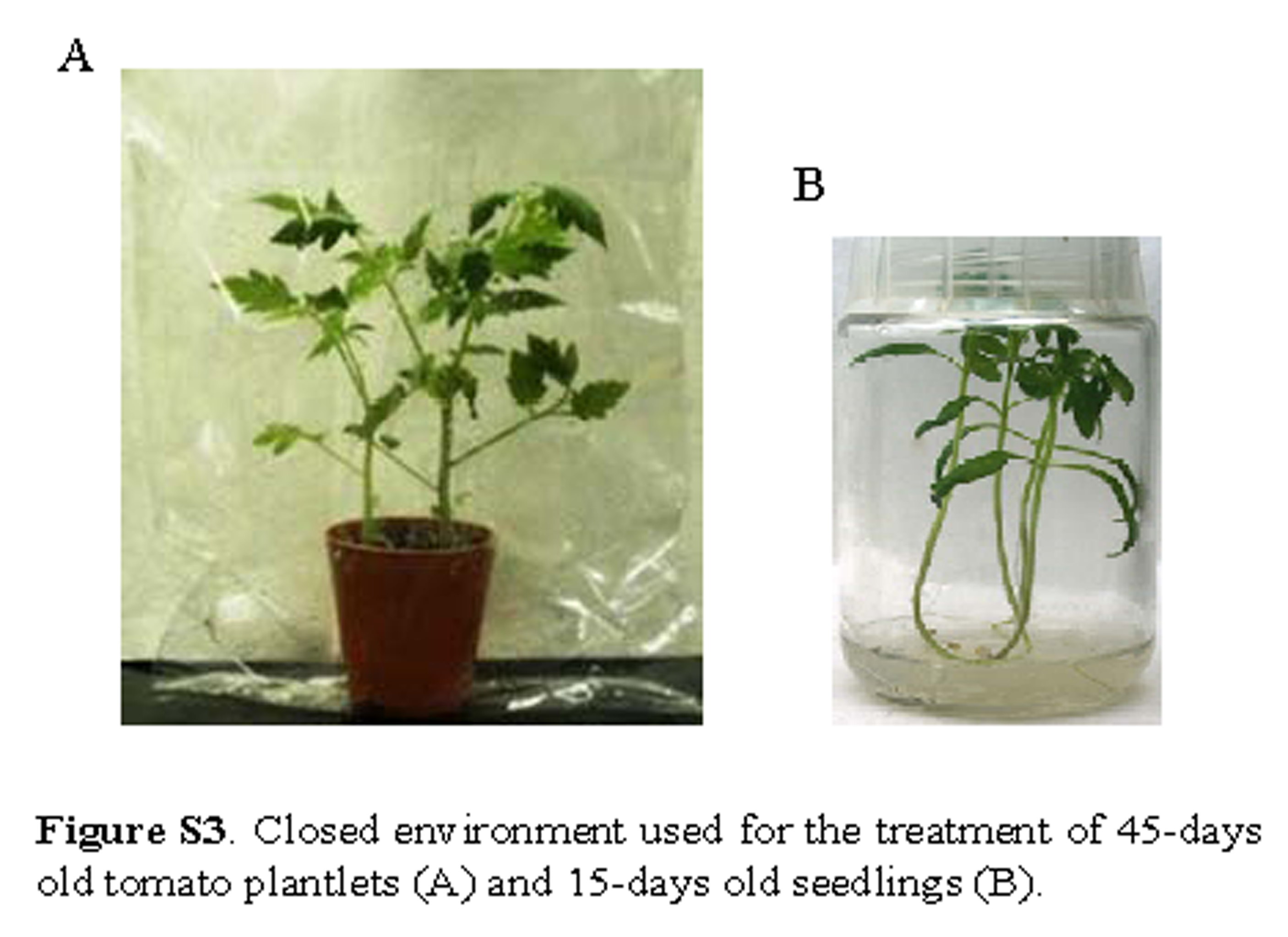

Supplement: Figure S3 — Closed environment used for the treatment of 45-days old tomato plantlets (A) and 15-days old seedlings (B). (TIF) [file pone.0076029.s003.tif]
