# Supplementary material for: Induction of Senescence and Identification of Differentially Expressed Genes in Tomato in Response to Monoterpene
Source: PLoS One. 2013 Sep 30;8(9):e76029. doi: 10.1371/journal.pone.0076029 (PMC3786903; doi:10.1371/journal.pone.0076029)
Supplement: Table S1 — GenBank accession numbers of ESTs. (DOC) [file pone.0076029.s004.doc]

**Table S1. GenBank accession numbers of ESTs.**

| **Sl No** | **EST ID** | **Length (bp)** | **Accession No** |
| --- | --- | --- | --- |
| 1 | P1A1 | 569 | GH203413 |
| 2 | P1A2 | 336 | GH203414 |
| 3 | P1A4 | 744 | GH203415 |
| 4 | P1A6 | 691 | GH203417 |
| 5 | P1A7 | 407 | GH203418 |
| 6 | P1A8 | 588 | GH203419 |
| 7 | P1A10 | 531 | GH203421 |
| 8 | P1A11 | 736 | GH203422 |
| 9 | P1A12 | 714 | GH203423 |
| 10 | P1B1 | 282 | GH203424 |
| 11 | P1B2 | 289 | GH203425 |
| 12 | P1B4 | 545 | GH203427 |
| 13 | P1B6 | 269 | GH203428 |
| 14 | P1B8 | 250 | GH203430 |
| 15 | P1B9 | 283 | GH203431 |
| 16 | P1B10 | 530 | GH203432 |
| 17 | P1B12 | 174 | GH203434 |
| 18 | P1C1 | 261 | GH203435 |
| 19 | P1C2 | 273 | GH203436 |
| 20 | P1C3 | 534 | GH203437 |
| 21 | P1C4 | 366 | GH203438 |
| 22 | P1C5 | 466 | GH203439 |
| 23 | P1C6 | 383 | GH203440 |
| 24 | P1C7 | 257 | GH203441 |
| 25 | P1C8 | 573 | GH203442 |
| 26 | P1C9 | 601 | GH203443 |
| 27 | P1C10 | 741 | GH203444 |
| 28 | P1C11 | 465 | GH203445 |
| 29 | P1C12 | 717 | GH203446 |
| 30 | P1D2 | 380 | GH203448 |
| 31 | P1D3 | 513 | GH203449 |
| 32 | P1D4 | 165 | GH203450 |
| 33 | P1D6 | 282 | GH203451 |
| 34 | P1D7 | 567 | GH203452 |
| 35 | P1D8 | 263 | GH203453 |
| 36 | P1D9 | 338 | GH203454 |
| 37 | P1D10 | 366 | GH203455 |
| 38 | P1D11 | 514 | GH203456 |
| 39 | P1D12 | 438 | GH203457 |
| 40 | P1E2 | 172 | GH203459 |
| 41 | P1E3 | 364 | GH203460 |
| 42 | P1E4 | 379 | GH203461 |
| 43 | P1E6 | 303 | GH203463 |
| 44 | P1E7 | 179 | GH203464 |
| 45 | P1E8 | 126 | GH203465 |
| 46 | P1E9 | 650 | GH203466 |
| 47 | P1E10 | 396 | GH203467 |
| 48 | P1E11 | 293 | GH203468 |
| 49 | P1E12 | 444 | GH203469 |
| 50 | P1F2 | 492 | GH203470 |
| 51 | P1F3 | 255 | GH203471 |
| 52 | P1F6 | 460 | GH203474 |
| 53 | P1F7 | 403 | GH203475 |
| 54 | P1F8 | 651 | GH203476 |
| 55 | P1F9 | 209 | GH203477 |
| 56 | P1F10 | 477 | GH203478 |
| 57 | P1F11 | 431 | GH203479 |
| 58 | P1F12 | 365 | GH203480 |
| 59 | P1G1 | 102 | GH203481 |
| 60 | P1G6 | 440 | GH203486 |
| 61 | P1G7 | 522 | GH203487 |
| 62 | P1G8 | 279 | GH203488 |
| 63 | P1G9 | 383 | GH203489 |
| 64 | P1G10 | 435 | GH203490 |
| 65 | P1G11 | 605 | GH203491 |
| 66 | P1G12 | 547 | GH203492 |
| 67 | P1H2 | 604 | GH203493 |
| 68 | P1H4 | 178 | GH203495 |
| 69 | P1H5 | 245 | GH203496 |
| 70 | P1H6 | 365 | GH203497 |
| 71 | P1H7 | 892 | GH203498 |
| 72 | P1H8 | 652 | GH203499 |
| 73 | P1H9 | 905 | GH203500 |
| 74 | P1H10 | 392 | GH203501 |
| 75 | P1H11 | 479 | GH203502 |
| 76 | P1H12 | 587 | GH203503 |
| 77 | P2A1 | 369 | GH203504 |
| 78 | P2A2 | 455 | GH203505 |
| 79 | P2A3 | 139 | GH203506 |
| 80 | P2A4 | 883 | GH203507 |
| 81 | P2A5 | 172 | GH203508 |
| 82 | P2A6 | 288 | GH203509 |
| 83 | P2A7 | 666 | GH203510 |
| 84 | P2A8 | 387 | GH203511 |
| 85 | P2A9 | 664 | GH203512 |
| 86 | P2A10 | 385 | GH203513 |
| 87 | P2A11 | 260 | GH203514 |
| 88 | P2A12 | 443 | GH203515 |
| 89 | P2B1 | 315 | GH203516 |
| 90 | P2B2 | 144 | GH203517 |
| 91 | P2B3 | 385 | GH203518 |
| 92 | P2B5 | 662 | GH203519 |
| 93 | P2B6 | 772 | GH203520 |
| 94 | P2B7 | 397 | GH203521 |
| 95 | P2B8 | 662 | GH203522 |
| 96 | P2B9 | 104 | GH203523 |
| 97 | P2B10 | 396 | GH203524 |
| 98 | P2B11 | 535 | GH203525 |
| 99 | P2B12 | 261 | GH203526 |
| 100 | P2C1 | 177 | GH203527 |
| 101 | P2C2 | 675 | GH203528 |
| 102 | P2C3 | 145 | GH203529 |
| 103 | P2C4 | 653 | GH203530 |
| 104 | P2C5 | 424 | GH203531 |
| 105 | P2C6 | 574 | GH203532 |
| 106 | P2C7 | 301 | GH203533 |
| 107 | P2C8 | 170 | GH203534 |
| 108 | P2C9 | 769 | GH203535 |
| 109 | P2C10 | 431 | GH203536 |
| 110 | P2C11 | 458 | GH203537 |
| 111 | P2C12 | 169 | GH203538 |
| 112 | P2D1 | 738 | GH203539 |
| 113 | P2D2 | 673 | GH203540 |
| 114 | P2D3 | 419 | GH203541 |
| 115 | P2D4 | 479 | GH203542 |
| 116 | P2D5 | 479 | GH203543 |
| 117 | P2D6 | 587 | GH203544 |
| 118 | P2D7 | 303 | GH203545 |
| 119 | P2D8 | 378 | GH203546 |
| 120 | P2D9 | 353 | GH203547 |
| 121 | P2D11 | 572 | GH203549 |
| 122 | P2D12 | 567 | GH203550 |
| 123 | P2E1 | 296 | GH203551 |
| 124 | P2E3 | 513 | GH203553 |
| 125 | P2E4 | 360 | GH203554 |
| 126 | P2E5 | 232 | GH203555 |
| 127 | P2E6 | 643 | GH203556 |
| 128 | P2E7 | 554 | GH203557 |
| 129 | P2E8 | 315 | GH203558 |
| 130 | P2E10 | 486 | GH203559 |
| 131 | P2E11 | 570 | GH203560 |
| 132 | P2E12 | 432 | GH203561 |
| 133 | P2F1 | 646 | GH203562 |
| 134 | P2F2 | 361 | GH203563 |
| 135 | P2F3 | 748 | GH203564 |
| 136 | P2F4 | 330 | GH203565 |
| 137 | P2F6 | 414 | GH203566 |
| 138 | P2F7 | 182 | GH203567 |
| 139 | P2F8 | 571 | GH203568 |
| 140 | P2F9 | 513 | GH203569 |
| 141 | P2F10 | 469 | GH203570 |
| 142 | P2F12 | 772 | GH203572 |
| 143 | P2G1 | 677 | GH203573 |
| 144 | P2G2 | 675 | GH203574 |
| 145 | P2G3 | 228 | GH203575 |
| 146 | P2G4 | 283 | GH203576 |
| 147 | P2G5 | 383 | GH203577 |
| 148 | P2G6 | 147 | GH203578 |
| 149 | P2G7 | 770 | GH203579 |
| 150 | P2G8 | 665 | GH203580 |
| 151 | P2G9 | 346 | GH203581 |
| 152 | P2G10 | 173 | GH203582 |
| 153 | P2G12 | 311 | GH203584 |
| 154 | P2H1 | 206 | GH203585 |
| 155 | P2H2 | 621 | GH203586 |
| 156 | P2H3 | 794 | GH203587 |
| 157 | P2H4 | 391 | GH203588 |
| 158 | P2H5 | 692 | GH203589 |
| 159 | P2H6 | 525 | GH203590 |
| 160 | P2H7 | 339 | GH203591 |
| 161 | P2H9 | 210 | GH203593 |
| 162 | P2H10 | 173 | GH203594 |
| 163 | P2H11 | 586 | GH203595 |
| 164 | P2H12 | 569 | GH203596 |
| 165 | P3A1 | 189 | GH203597 |
| 166 | P3A2 | 396 | GH203598 |
| 167 | P3A4 | 296 | GH203599 |
| 168 | P3A5 | 599 | GH203600 |
| 169 | P3A6 | 335 | GH203601 |
| 170 | P3A7 | 211 | GH203602 |
| 171 | P3A8 | 383 | GH203603 |
| 172 | P3A10 | 571 | GH203604 |
| 173 | P3A11 | 901 | GH203605 |
| 174 | P3A12 | 702 | GH203606 |
| 175 | P3B1 | 934 | GH203607 |
| 176 | P3B2 | 271 | GH203608 |
| 177 | P3B3 | 159 | GH203609 |
| 178 | P3B4 | 360 | GH203610 |
| 179 | P3B5 | 327 | GH203611 |
| 180 | P3B6 | 583 | GH203612 |
| 181 | P3B7 | 750 | GH203613 |
| 182 | P3B8 | 239 | GH203614 |
| 183 | P3B9 | 565 | GH203615 |
| 184 | P3B11 | 516 | GH203616 |
| 185 | P3B12 | 641 | GH203617 |
| 186 | P3C1 | 247 | GH203618 |
| 187 | P3C2 | 810 | GH203619 |
| 188 | P3C3 | 793 | GH203620 |
| 189 | P3C4 | 673 | GH203621 |
| 190 | P3C5 | 628 | GH203622 |
| 191 | P3C6 | 542 | GH203623 |
| 192 | P3C7 | 823 | GH203624 |
| 193 | P3C8 | 592 | GH203625 |
| 194 | P3C10 | 153 | GH203627 |
| 195 | P3C11 | 220 | GH203628 |
| 196 | P3C12 | 674 | GH203629 |
| 197 | P3D1 | 280 | GH203630 |
| 198 | P3D2 | 326 | GH203631 |
| 199 | P3D3 | 361 | GH203632 |
| 200 | P3D4 | 927 | GH203633 |
| 201 | P3D5 | 730 | GH203634 |
| 202 | P3D6 | 233 | GH203635 |
| 203 | P3D7 | 224 | GH203636 |
| 204 | P3D8 | 597 | GH203637 |
| 205 | P3D10 | 636 | GH203638 |
| 206 | P3D12 | 562 | GH203639 |
| 207 | P3E4 | 580 | GH203642 |
| 208 | P3E5 | 590 | GH203643 |
| 209 | P3E8 | 161 | GH203645 |
| 210 | P3E10 | 192 | GH203647 |
| 211 | P3E11 | 498 | GH203648 |
| 212 | P3E12 | 646 | GH203649 |
| 213 | P3F1 | 782 | GH203650 |
| 214 | P3F2 | 586 | GH203651 |
| 215 | P3F3 | 460 | GH203652 |
| 216 | P3F5 | 730 | GH203653 |
| 217 | P3F6 | 327 | GH203654 |
| 218 | P3F7 | 670 | GH203655 |
| 219 | P3F8 | 161 | GH203656 |
| 220 | P3F10 | 577 | GH203657 |
| 221 | P3F11 | 190 | GH203658 |
| 222 | P3F12 | 908 | GH203659 |
| 223 | P3G1 | 301 | GH203660 |
| 224 | P3G2 | 692 | GH203661 |
| 225 | P3G3 | 523 | GH203662 |
| 226 | P3G4 | 627 | GH203663 |
| 227 | P3G6 | 542 | GH203665 |
| 228 | P3G7 | 441 | GH203666 |
| 229 | P3G9 | 570 | GH203668 |
| 230 | P3G10 | 496 | GH203669 |
| 231 | P3G11 | 327 | GH203670 |
| 232 | P3G12 | 615 | GH203671 |
| 233 | P3H1 | 844 | GH203672 |
| 234 | P3H2 | 589 | GH203673 |
| 235 | P3H3 | 345 | GH203674 |
| 236 | P3H4 | 516 | GH203675 |
| 237 | P3H6 | 202 | GH203677 |
| 238 | P3H7 | 635 | GH203678 |
| 239 | P3H9 | 841 | GH203680 |
| 240 | P3H11 | 370 | GH203682 |
| 241 | P4A1 | 195 | GH203684 |
| 242 | P4A2 | 189 | GH203685 |
| 243 | P4A4 | 420 | GH203687 |
| 244 | P4A5 | 432 | GH203688 |
| 245 | P4A8 | 861 | GH203691 |
| 246 | P4A9 | 285 | GH203692 |
| 247 | P4A10 | 325 | GH203693 |
| 248 | P4A11 | 481 | GH203694 |
| 249 | P4A12 | 420 | GH203695 |
| 250 | P4B1 | 402 | GH203696 |
| 251 | P4B2 | 554 | GH203697 |
| 252 | P4B3 | 219 | GH203698 |
| 253 | P4B4 | 419 | GH203699 |
| 254 | P4B5 | 716 | GH203700 |
| 255 | P4B6 | 716 | GH203701 |
| 256 | P4B7 | 419 | GH203702 |
| 257 | P4B8 | 824 | GH203703 |
| 258 | P4B9 | 698 | GH203704 |
| 259 | P4B10 | 254 | GH203705 |
| 260 | P4B11 | 566 | GH203706 |
| 261 | P4B12 | 895 | GH203707 |
| 262 | P4C1 | 891 | GH203708 |
| 263 | P4C2 | 197 | GH203709 |
| 264 | P4C3 | 574 | GH203710 |
| 265 | P4C4 | 471 | GH203711 |
| 266 | P4C5 | 467 | GH203712 |
| 267 | P4C6 | 467 | GH203713 |
| 268 | P4C7 | 336 | GH203714 |
| 269 | P4C8 | 538 | GH203715 |
| 270 | P4C9 | 455 | GH203716 |
| 271 | P4C10 | 744 | GH203717 |
| 272 | P4C11 | 709 | GH203718 |
| 273 | P4C12 | 274 | GH203719 |
| 274 | P4D1 | 229 | GH203720 |
| 275 | P4D2 | 416 | GH203721 |
| 276 | P4D3 | 472 | GH203722 |
| 277 | P4D4 | 648 | GH203723 |
| 278 | P4D5 | 367 | GH203724 |
| 279 | P4D7 | 181 | GH203726 |
| 280 | P4D8 | 556 | GH203727 |
| 281 | P4D10 | 314 | GH203729 |
| 282 | P4D11 | 402 | GH203730 |
| 283 | P4D12 | 402 | GH203731 |
| 284 | P4E1 | 126 | GH203732 |
| 285 | P4E2 | 215 | GH203733 |
| 286 | P4E3 | 583 | GH203734 |
| 287 | P4E4 | 554 | GH203735 |
| 288 | P4E6 | 460 | GH203737 |
| 289 | P4E9 | 328 | GH203740 |
| 290 | P4E10 | 220 | GH203741 |
| 291 | P4E11 | 832 | GH203742 |
| 292 | P4E12 | 740 | GH203743 |
| 293 | P4F1 | 468 | GH203744 |
| 294 | P4F2 | 518 | GH203745 |
| 295 | P4F3 | 351 | GH203746 |
| 296 | P4F4 | 895 | GH203747 |
| 297 | P4F5 | 920 | GH203748 |
| 298 | P4F6 | 858 | GH203749 |
| 299 | P4F7 | 162 | GH203750 |
| 300 | P4F8 | 189 | GH203751 |
| 301 | P4F9 | 261 | GH203752 |
| 302 | P4F10 | 530 | GH203753 |
| 303 | P4F11 | 165 | GH203754 |
| 304 | P4F12 | 335 | GH203755 |
| 305 | P4G1 | 692 | GH203756 |
| 306 | P4G4 | 540 | GH203759 |
| 307 | P4G5 | 236 | GH203760 |
| 308 | P4G6 | 267 | GH203761 |
| 309 | P4G7 | 637 | GH203762 |
| 310 | P4G8 | 586 | GH203763 |
| 311 | P4G9 | 505 | GH203764 |
| 312 | P4G10 | 403 | GH203765 |
| 313 | P4G11 | 165 | GH203766 |
| 314 | P4G12 | 273 | GH203767 |
| 315 | P4H1 | 692 | GH203768 |
| 316 | P4H2 | 320 | GH203769 |
| 317 | P4H3 | 309 | GH203770 |
| 318 | P4H4 | 517 | GH203771 |
| 319 | P4H5 | 654 | GH203772 |
| 320 | P4H6 | 360 | GH203773 |
| 321 | P4H7 | 949 | GH203774 |
| 322 | P4H8 | 229 | GH203775 |
| 323 | P4H9 | 300 | GH203776 |
| 324 | P4H10 | 351 | GH203777 |
| 325 | P4H11 | 181 | GH203778 |
| 326 | P4H12 | 729 | GH203779 |
| 327 | P5A1 | 229 | GH203780 |
| 328 | P5A2 | 132 | GH203781 |
| 329 | P5A6 | 565 | GH203783 |
| 330 | P5A7 | 851 | GH203784 |
| 331 | P5A8 | 522 | GH203785 |
| 332 | P5A10 | 403 | GH203787 |
| 333 | P5A11 | 231 | GH203788 |
| 334 | P5A12 | 269 | GH203789 |
| 335 | P5B1 | 572 | GH203790 |
| 336 | P5B2 | 446 | GH203791 |
| 337 | P5B4 | 189 | GH203793 |
| 338 | P5B5 | 399 | GH203794 |
| 339 | P5B6 | 551 | GH203795 |
| 340 | P5B7 | 401 | GH203796 |
| 341 | P5B8 | 468 | GH203797 |
| 342 | P5B9 | 256 | GH203798 |
| 343 | P5B11 | 186 | GH203799 |
| 344 | P5C1 | 419 | GH203800 |
| 345 | P5C2 | 443 | GH203801 |
| 346 | P5C5 | 512 | GH203803 |
| 347 | P5C6 | 342 | GH203804 |
| 348 | P5C7 | 590 | GH203805 |
| 349 | P5C8 | 611 | GH203806 |
| 350 | P5C9 | 371 | GH203807 |
| 351 | P5C10 | 254 | GH203808 |
| 352 | P5C11 | 570 | GH203809 |
| 353 | P5C12 | 181 | GH203810 |
| 354 | P5D1 | 195 | GH203811 |
| 355 | P5D2 | 438 | GH203812 |
| 356 | P5D4 | 437 | GH203814 |
| 357 | P5D6 | 450 | GH203815 |
| 358 | P5D7 | 732 | GH203816 |
| 359 | P5D9 | 312 | GH203817 |
| 360 | P5D11 | 744 | GH203818 |
| 361 | P5E1 | 365 | GH203819 |
| 362 | P5E2 | 190 | GH203820 |
| 363 | P5E4 | 617 | GH203822 |
| 364 | P5E6 | 703 | GH203823 |
| 365 | P5E7 | 369 | GH203824 |
| 366 | P5E9 | 433 | GH203825 |
| 367 | P5E11 | 338 | GH203827 |
| 368 | P5E12 | 233 | GH203828 |
| 369 | P5F1 | 870 | GH203829 |
| 370 | P5F2 | 635 | GH203830 |
| 371 | P5F5 | 232 | GH203832 |
| 372 | P5F6 | 195 | GH203833 |
| 373 | P5F7 | 738 | GH203834 |
| 374 | P5F9 | 321 | GH203836 |
| 375 | P5F10 | 443 | GH203837 |
| 376 | P5F11 | 399 | GH203838 |
| 377 | P5F12 | 138 | GH203839 |
| 378 | P5G2 | 655 | GH203840 |
| 379 | P5G5 | 212 | GH203842 |
| 380 | P5G6 | 445 | GH203843 |
| 381 | P5G7 | 353 | GH203844 |
| 382 | P5G9 | 110 | GH203846 |
| 383 | P5G10 | 183 | GH203847 |
| 384 | P5G11 | 333 | GH203848 |
| 385 | P5G12 | 221 | GH203849 |
| 386 | P5H1 | 248 | GH203850 |
| 387 | P5H6 | 499 | GH203855 |
| 388 | P5H7 | 723 | GH203856 |
| 389 | P5H9 | 391 | GH203857 |
| 390 | P5H10 | 342 | GH203858 |
| 391 | P5H11 | 508 | GH203859 |
| 392 | P5H12 | 455 | GH203860 |
| 393 | P6A1 | 269 | GH203861 |
| 394 | P6A2 | 591 | GH203862 |
| 395 | P6A3 | 454 | GH203863 |
| 396 | P6A4 | 479 | GH203864 |
| 397 | P6A6 | 283 | GH203865 |
| 398 | P6A7 | 437 | GH203866 |
| 399 | P6A8 | 229 | GH203867 |
| 400 | P6A9 | 327 | GH203868 |
| 401 | P6A10 | 134 | GH203869 |
| 402 | P6A11 | 764 | GH203870 |
| 403 | P6A12 | 211 | GH203871 |
| 404 | P6B1 | 327 | GH203872 |
| 405 | P6B2 | 393 | GH203873 |
| 406 | P6B4 | 878 | GH203874 |
| 407 | P6B6 | 270 | GH203875 |
| 408 | P6B7 | 555 | GH203876 |
| 409 | P6B8 | 246 | GH203877 |
| 410 | P6B9 | 445 | GH203878 |
| 411 | P6B10 | 652 | GH203879 |
| 412 | P6B11 | 416 | GH203880 |
| 413 | P6B12 | 832 | GH203881 |
| 414 | P6C1 | 139 | GH203882 |
| 415 | P6C2 | 651 | GH203883 |
| 416 | P6C5 | 144 | GH203885 |
| 417 | P6C7 | 331 | GH203886 |
| 418 | P6C8 | 906 | GH203887 |
| 419 | P6C9 | 547 | GH203888 |
| 420 | P6C10 | 673 | GH203889 |
| 421 | P6C11 | 512 | GH203890 |
| 422 | P6C12 | 476 | GH203891 |
| 423 | P6D1 | 340 | GH203892 |
| 424 | P6D2 | 597 | GH203893 |
| 425 | P6D3 | 233 | GH203894 |
| 426 | P6D4 | 298 | GH203895 |
| 427 | P6D5 | 606 | GH203896 |
| 428 | P6D7 | 372 | GH203898 |
| 429 | P6D9 | 455 | GH203899 |
| 430 | P6D10 | 912 | GH203900 |
| 431 | P6D11 | 407 | GH203901 |
| 432 | P6E1 | 429 | GH203902 |
| 433 | P6E2 | 496 | GH203903 |
| 434 | P6E3 | 680 | GH203904 |
| 435 | P6E4 | 470 | GH203905 |
| 436 | P6E5 | 244 | GH203906 |
| 437 | P6E6 | 614 | GH203907 |
| 438 | P6E7 | 630 | GH203908 |
| 439 | P6E8 | 577 | GH203909 |
| 440 | P6E9 | 593 | GH203910 |
| 441 | P6E10 | 187 | GH203911 |
| 442 | P6E11 | 181 | GH203912 |
| 443 | P6E12 | 224 | GH203913 |
| 444 | P6F1 | 218 | GH203914 |
| 445 | P6F2 | 606 | GH203915 |
| 446 | P6F3 | 783 | GH203916 |
| 447 | P6F5 | 713 | GH203918 |
| 448 | P6F7 | 369 | GH203919 |
| 449 | P6F8 | 701 | GH203920 |
| 450 | P6F10 | 283 | GH203921 |
| 451 | P6F11 | 482 | GH203922 |
| 452 | P6F12 | 828 | GH203923 |
| 453 | P6G1 | 191 | GH203924 |
| 454 | P6G2 | 822 | GH203925 |
| 455 | P6G3 | 288 | GH203926 |
| 456 | P6G4 | 238 | GH203927 |
| 457 | P6G5 | 587 | GH203928 |
| 458 | P6G7 | 242 | GH203930 |
| 459 | P6G8 | 637 | GH203931 |
| 460 | P6G9 | 174 | GH203932 |
| 461 | P6G10 | 667 | GH203933 |
| 462 | P6G11 | 219 | GH203934 |
| 463 | P6G12 | 269 | GH203935 |
| 464 | P6H1 | 387 | GH203936 |
| 465 | P6H2 | 468 | GH203937 |
| 466 | P6H3 | 652 | GH203938 |
| 467 | P6H4 | 454 | GH203939 |
| 468 | P6H5 | 576 | GH203940 |
| 469 | P6H6 | 258 | GH203941 |
| 470 | P6H7 | 471 | GH203942 |
| 471 | P6H8 | 316 | GH203943 |
| 472 | P6H9 | 195 | GH203944 |
| 473 | P6H10 | 386 | GH203945 |
| 474 | P6H11 | 211 | GH203946 |
| 475 | P6H12 | 467 | GH203947 |
| 476 | P7A1 | 395 | GH203948 |
| 477 | P7A4 | 401 | GH203951 |
| 478 | P7A5 | 434 | GH203952 |
| 479 | P7A6 | 409 | GH203953 |
| 480 | P7A7 | 893 | GH203954 |
| 481 | P7A9 | 402 | GH203956 |
| 482 | P7A10 | 416 | GH203957 |
| 483 | P7A11 | 179 | GH203958 |
| 484 | P7A12 | 239 | GH203959 |
| 485 | P7B1 | 601 | GH203960 |
| 486 | P7B3 | 707 | GH203962 |
| 487 | P7B4 | 547 | GH203963 |
| 488 | P7B5 | 212 | GH203964 |
| 489 | P7B6 | 316 | GH203965 |
| 490 | P7B8 | 455 | GH203967 |
| 491 | P7B9 | 455 | GH203968 |
| 492 | P7B10 | 682 | GH203969 |
| 493 | P7B11 | 396 | GH203970 |
| 494 | P7B12 | 220 | GH203971 |
| 495 | P7C1 | 819 | GH203972 |
| 496 | P7C2 | 972 | GH203973 |
| 497 | P7C3 | 530 | GH203974 |
| 498 | P7C5 | 581 | GH203976 |
| 499 | P7C6 | 395 | GH203977 |
| 500 | P7C8 | 438 | GH203979 |
| 501 | P7C11 | 231 | GH203981 |
| 502 | P7C12 | 151 | GH203982 |
| 503 | P7D1 | 812 | GH203983 |
| 504 | P7D5 | 143 | GH203985 |
| 505 | P7D7 | 574 | GH203986 |
| 506 | P7D8 | 466 | GH203987 |
| 507 | P7D9 | 542 | GH203988 |
| 508 | P7D10 | 677 | GH203989 |
| 509 | P7D11 | 315 | GH203990 |
| 510 | P7E1 | 811 | GH203992 |
| 511 | P7E2 | 399 | GH203993 |
| 512 | P7E5 | 373 | GH203996 |
| 513 | P7E6 | 785 | GH203997 |
| 514 | P7E7 | 263 | GH203998 |
| 515 | P7E8 | 670 | GH203999 |
| 516 | P7E9 | 571 | GH204000 |
| 517 | P7E10 | 247 | GH204001 |
| 518 | P7E12 | 283 | GH204002 |
| 519 | P7F1 | 229 | GH204003 |
| 520 | P7F2 | 348 | GH204004 |
| 521 | P7F5 | 330 | GH204007 |
| 522 | P7F7 | 336 | GH204009 |
| 523 | P7F8 | 132 | GH204010 |
| 524 | P7F9 | 364 | GH204011 |
| 525 | P7F10 | 490 | GH204012 |
| 526 | P7F11 | 314 | GH204013 |
| 527 | P7F12 | 211 | GH204014 |
| 528 | P7G1 | 352 | GH204015 |
| 529 | P7G3 | 438 | GH204016 |
| 530 | P7G4 | 150 | GH204017 |
| 531 | P7G5 | 445 | GH204018 |
| 532 | P7G6 | 457 | GH204019 |
| 533 | P7G7 | 598 | GH204020 |
| 534 | P7G8 | 583 | GH204021 |
| 535 | P7G9 | 119 | GH204022 |
| 536 | P7G10 | 251 | GH204023 |
| 537 | P7G11 | 804 | GH204024 |
| 538 | P7G12 | 649 | GH204025 |
| 539 | P7H1 | 597 | GH204026 |
| 540 | P7H2 | 348 | GH204027 |
| 541 | P7H8 | 251 | GH204029 |
| 542 | P7H9 | 626 | GH204030 |
| 543 | P7H10 | 241 | GH204031 |
| 544 | P7H11 | 231 | GH204032 |
| 545 | P7H12 | 220 | GH204033 |
| 546 | P8A1 | 417 | GH204034 |
| 547 | P8A2 | 738 | GH204035 |
| 548 | P8A3 | 265 | GH204036 |
| 549 | P8A4 | 656 | GH204037 |
| 550 | P8A5 | 262 | GH204038 |
| 551 | P8A6 | 397 | GH204039 |
| 552 | P8A7 | 658 | GH204040 |
| 553 | P8A8 | 385 | GH204041 |
| 554 | P8A9 | 545 | GH204042 |
| 555 | P8A10 | 746 | GH204043 |
| 556 | P8A11 | 257 | GH204044 |
| 557 | P8B1 | 620 | GH204045 |
| 558 | P8B2 | 246 | GH204046 |
| 559 | P8B3 | 153 | GH204047 |
| 560 | P8B5 | 477 | GH204048 |
| 561 | P8B6 | 401 | GH204049 |
| 562 | P8B7 | 639 | GH204050 |
| 563 | P8B8 | 644 | GH204051 |
| 564 | P8B10 | 546 | GH204052 |
| 565 | P8B11 | 573 | GH204053 |
| 566 | P8B12 | 878 | GH204054 |
| 567 | P8C1 | 710 | GH204055 |
| 568 | P8C3 | 545 | GH204056 |
| 569 | P8C4 | 333 | GH204057 |
| 570 | P8C5 | 477 | GH204058 |
| 571 | P8C6 | 501 | GH204059 |
| 572 | P8C11 | 262 | GH204062 |
| 573 | P8C12 | 811 | GH204063 |
| 574 | P8D1 | 319 | GH204064 |
| 575 | P8D2 | 272 | GH204065 |
| 576 | P8D4 | 216 | GH204066 |
| 577 | P8D5 | 460 | GH204067 |
| 578 | P8D6 | 300 | GH204068 |
| 579 | P8D7 | 323 | GH204069 |
| 580 | P8D8 | 925 | GH204070 |
| 581 | P8D10 | 153 | GH204072 |
| 582 | P8D11 | 633 | GH204073 |
| 583 | P8D12 | 421 | GH204074 |
| 584 | P8E1 | 606 | GH204075 |
| 585 | P8E3 | 376 | GH204076 |
| 586 | P8E4 | 341 | GH204077 |
| 587 | P8E5 | 512 | GH204078 |
| 588 | P8E6 | 711 | GH204079 |
| 589 | P8E9 | 360 | GH204080 |
| 590 | P8E10 | 137 | GH204081 |
| 591 | P8E11 | 730 | GH204082 |
| 592 | P8E12 | 659 | GH204083 |
| 593 | P8F1 | 241 | GH204084 |
| 594 | P8F2 | 325 | GH204085 |
| 595 | P8F3 | 488 | GH204086 |
| 596 | P8F4 | 738 | GH204087 |
| 597 | P8F5 | 589 | GH204088 |
| 598 | P8F7 | 450 | GH204089 |
| 599 | P8F8 | 340 | GH204090 |
| 600 | P8F12 | 571 | GH204091 |
| 601 | P8G1 | 630 | GH204092 |
| 602 | P8G2 | 498 | GH204093 |
| 603 | P8G3 | 722 | GH204094 |
| 604 | P8G4 | 432 | GH204095 |
| 605 | P8G5 | 607 | GH204096 |
| 606 | P8G6 | 675 | GH204097 |
| 607 | P8G7 | 568 | GH204098 |
| 608 | P8G9 | 470 | GH204099 |
| 609 | P8G10 | 368 | GH204100 |
| 610 | P8G11 | 587 | GH204101 |
| 611 | P8G12 | 191 | GH204102 |
| 612 | P8H1 | 663 | GH204103 |
| 613 | P8H2 | 185 | GH204104 |
| 614 | P8H4 | 545 | GH204105 |
| 615 | P8H5 | 814 | GH204106 |
| 616 | P8H6 | 270 | GH204107 |
| 617 | P8H7 | 531 | GH204108 |
| 618 | P8H8 | 331 | GH204109 |
| 619 | P8H9 | 274 | GH204110 |
| 620 | P8H10 | 386 | GH204111 |
| 621 | P8H11 | 265 | GH204112 |
| 622 | P8H12 | 234 | GH204113 |
| 623 | P9A1 | 614 | GH204114 |
| 624 | P9A2 | 692 | GH204115 |
| 625 | P9A3 | 238 | GH204116 |
| 626 | P9A4 | 415 | GH204117 |
| 627 | P9A5 | 586 | GH204118 |
| 628 | P9A6 | 495 | GH204119 |
| 629 | P9A7 | 326 | GH204120 |
| 630 | P9A8 | 194 | GH204121 |
| 631 | P9A9 | 134 | GH204122 |
| 632 | P9A10 | 726 | GH204123 |
| 633 | P9A11 | 697 | GH204124 |
| 634 | P9B1 | 703 | GH204125 |
| 635 | P9B3 | 401 | GH204126 |
| 636 | P9B4 | 605 | GH204127 |
| 637 | P9B5 | 572 | GH204128 |
| 638 | P9B6 | 691 | GH204129 |
| 639 | P9B7 | 338 | GH204130 |
| 640 | P9B8 | 568 | GH204131 |
| 641 | P9B10 | 436 | GH204132 |
| 642 | P9B11 | 455 | GH204133 |
| 643 | P9C1 | 879 | GH204134 |
| 644 | P9C2 | 231 | GH204135 |
| 645 | P9C4 | 569 | GH204136 |
| 646 | P9C6 | 394 | GH204137 |
| 647 | P9C8 | 317 | GH204138 |
| 648 | P9C9 | 362 | GH204139 |
| 649 | P9C10 | 816 | GH204140 |
| 650 | P9C11 | 566 | GH204141 |
| 651 | P9C12 | 823 | GH204142 |
| 652 | P9D1 | 827 | GH204143 |
| 653 | P9D2 | 269 | GH204144 |
| 654 | P9D4 | 333 | GH204145 |
| 655 | P9D5 | 264 | GH204146 |
| 656 | P9D6 | 617 | GH204147 |
| 657 | P9D7 | 794 | GH204148 |
| 658 | P9D8 | 362 | GH204149 |
| 659 | P9D9 | 489 | GH204150 |
| 660 | P9D10 | 355 | GH204151 |
| 661 | P9D11 | 403 | GH204152 |
| 662 | P9D12 | 481 | GH204153 |
| 663 | P9E2 | 181 | GH204154 |
| 664 | P9E4 | 600 | GH204156 |
| 665 | P9E5 | 417 | GH204157 |
| 666 | P9E6 | 395 | GH204158 |
| 667 | P9E7 | 440 | GH204159 |
| 668 | P9E9 | 899 | GH204161 |
| 669 | P9E10 | 436 | GH204162 |
| 670 | P9E11 | 427 | GH204163 |
| 671 | P9F2 | 789 | GH204165 |
| 672 | P9F3 | 411 | GH204166 |
| 673 | P9F4 | 237 | GH204167 |
| 674 | P9F5 | 584 | GH204168 |
| 675 | P9F6 | 232 | GH204169 |
| 676 | P9F7 | 508 | GH204170 |
| 677 | P9F8 | 192 | GH204171 |
| 678 | P9F10 | 375 | GH204172 |
| 679 | P9F11 | 697 | GH204173 |
| 680 | P9F12 | 226 | GH204174 |
| 681 | P9G1 | 461 | GH204175 |
| 682 | P9G2 | 227 | GH204176 |
| 683 | P9G3 | 359 | GH204177 |
| 684 | P9G4 | 444 | GH204178 |
| 685 | P9G6 | 304 | GH204180 |
| 686 | P9G7 | 790 | GH204181 |
| 687 | P9G8 | 265 | GH204182 |
| 688 | P9G9 | 585 | GH204183 |
| 689 | P9G10 | 432 | GH204184 |
| 690 | P9G11 | 618 | GH204185 |
| 691 | P9G12 | 653 | GH204186 |
| 692 | P9H1 | 140 | GH204187 |
| 693 | P9H2 | 585 | GH204188 |
| 694 | P9H3 | 571 | GH204189 |
| 695 | P9H4 | 245 | GH204190 |
| 696 | P9H5 | 292 | GH204191 |
| 697 | P9H6 | 528 | GH204192 |
| 698 | P9H7 | 756 | GH204193 |
| 699 | P9H8 | 445 | GH204194 |
| 700 | P9H12 | 487 | GH204196 |
| 701 | P10A1 | 728 | GH204197 |
| 702 | P10A3 | 537 | GH204199 |
| 703 | P10A4 | 102 | GH204200 |
| 704 | P10A5 | 573 | GH204201 |
| 705 | P10A6 | 245 | GH204202 |
| 706 | P10A7 | 797 | GH204203 |
| 707 | P10A9 | 635 | GH204205 |
| 708 | P10A11 | 205 | GH204206 |
| 709 | P10A12 | 715 | GH204207 |
| 710 | P10B1 | 289 | GH204208 |
| 711 | P10B2 | 387 | GH204209 |
| 712 | P10B3 | 538 | GH204210 |
| 713 | P10B4 | 546 | GH204211 |
| 714 | P10B5 | 592 | GH204212 |
| 715 | P10B6 | 318 | GH204213 |
| 716 | P10B8 | 265 | GH204215 |
| 717 | P10B9 | 635 | GH204216 |
| 718 | P10B10 | 558 | GH204217 |
| 719 | P10B11 | 624 | GH204218 |
| 720 | P10B12 | 287 | GH204219 |
| 721 | P10C1 | 487 | GH204220 |
| 722 | P10C2 | 415 | GH204221 |
| 723 | P10C3 | 696 | GH204222 |
| 724 | P10C4 | 375 | GH204223 |
| 725 | P10C5 | 703 | GH204224 |
| 726 | P10C6 | 712 | GH204225 |
| 727 | P10C7 | 681 | GH204226 |
| 728 | P10C8 | 125 | GH204227 |
| 729 | P10C9 | 309 | GH204228 |
| 730 | P10C10 | 769 | GH204229 |
| 731 | P10C11 | 159 | GH204230 |
| 732 | P10C12 | 825 | GH204231 |
| 733 | P10D1 | 791 | GH204232 |
| 734 | P10D2 | 231 | GH204233 |
| 735 | P10D3 | 467 | GH204234 |
| 736 | P10D4 | 299 | GH204235 |
| 737 | P10D5 | 386 | GH204236 |
| 738 | P10D6 | 787 | GH204237 |
| 739 | P10D8 | 424 | GH204239 |
| 740 | P10D9 | 298 | GH204240 |
| 741 | P10D10 | 380 | GH204241 |
| 742 | P10D11 | 613 | GH204242 |
| 743 | P10D12 | 833 | GH204243 |
| 744 | P10E1 | 694 | GH204244 |
| 745 | P10E2 | 702 | GH204245 |
| 746 | P10E3 | 321 | GH204246 |
| 747 | P10E4 | 350 | GH204247 |
| 748 | P10E5 | 417 | GH204248 |
| 749 | P10E6 | 728 | GH204249 |
| 750 | P10E7 | 681 | GH204250 |
| 751 | P10E8 | 435 | GH204251 |
| 752 | P10E9 | 518 | GH204252 |
| 753 | P10E10 | 739 | GH204253 |
| 754 | P10E11 | 385 | GH204254 |
| 755 | P10E12 | 205 | GH204255 |
| 756 | P10F1 | 450 | GH204256 |
| 757 | P10F2 | 370 | GH204257 |
| 758 | P10F3 | 653 | GH204258 |
| 759 | P10F4 | 841 | GH204259 |
| 760 | P10F5 | 683 | GH204260 |
| 761 | P10F6 | 738 | GH204261 |
| 762 | P10F8 | 583 | GH204263 |
| 763 | P10F10 | 295 | GH204265 |
| 764 | P10F11 | 107 | GH204266 |
| 765 | P10F12 | 799 | GH204267 |
| 766 | P10G1 | 677 | GH204268 |
| 767 | P10G3 | 191 | GH204270 |
| 768 | P10G4 | 875 | GH204271 |
| 769 | P10G5 | 665 | GH204272 |
| 770 | P10G6 | 696 | GH204273 |
| 771 | P10G7 | 526 | GH204274 |
| 772 | P10G8 | 613 | GH204275 |
| 773 | P10G9 | 714 | GH204276 |
| 774 | P10G10 | 565 | GH204277 |
| 775 | P10G11 | 499 | GH204278 |
| 776 | P10G12 | 666 | GH204279 |
| 777 | P10H1 | 251 | GH204280 |
| 778 | P10H2 | 408 | GH204281 |
| 779 | P10H3 | 587 | GH204282 |
| 780 | P10H4 | 712 | GH204283 |
| 781 | P10H6 | 458 | GH204285 |
| 782 | P10H7 | 696 | GH204286 |
| 783 | P10H10 | 749 | GH204289 |
| 784 | P10H11 | 502 | GH204290 |
| 785 | P10H12 | 331 | GH204291 |
| 786 | P11A1 | 498 | GH204292 |
| 787 | P11A3 | 562 | GH204293 |
| 788 | P11A5 | 565 | GH204295 |
| 789 | P11A7 | 602 | GH204296 |
| 790 | P11A8 | 763 | GH204297 |
| 791 | P11A9 | 519 | GH204298 |
| 792 | P11A11 | 343 | GH204300 |
| 793 | P11B1 | 476 | GH204301 |
| 794 | P11B2 | 444 | GH204302 |
| 795 | P11B3 | 778 | GH204303 |
| 796 | P11B4 | 179 | GH204304 |
| 797 | P11B5 | 214 | GH204305 |
| 798 | P11B6 | 520 | GH204306 |
| 799 | P11B7 | 746 | GH204307 |
| 800 | P11B8 | 583 | GH204308 |
| 801 | P11B9 | 367 | GH204309 |
| 802 | P11B12 | 646 | GH204312 |
| 803 | P11C1 | 251 | GH204313 |
| 804 | P11C2 | 547 | GH204314 |
| 805 | P11C3 | 306 | GH204315 |
| 806 | P11C4 | 386 | GH204316 |
| 807 | P11C5 | 776 | GH204317 |
| 808 | P11C6 | 652 | GH204318 |
| 809 | P11C7 | 764 | GH204319 |
| 810 | P11C8 | 543 | GH204320 |
| 811 | P11C9 | 519 | GH204321 |
| 812 | P11C10 | 749 | GH204322 |
| 813 | P11C12 | 583 | GH204324 |
| 814 | P11D1 | 581 | GH204325 |
| 815 | P11D2 | 547 | GH204326 |
| 816 | P11D3 | 429 | GH204327 |
| 817 | P11D6 | 185 | GH204329 |
| 818 | P11D7 | 514 | GH204330 |
| 819 | P11D8 | 543 | GH204331 |
| 820 | P11D10 | 438 | GH204333 |
| 821 | P11D11 | 661 | GH204334 |
| 822 | P11D12 | 270 | GH204335 |
| 823 | P11E1 | 370 | GH204336 |
| 824 | P11E3 | 220 | GH204337 |
| 825 | P11E4 | 607 | GH204338 |
| 826 | P11E7 | 472 | GH204341 |
| 827 | P11E8 | 269 | GH204342 |
| 828 | P11E9 | 577 | GH204343 |
| 829 | P11E10 | 331 | GH204344 |
| 830 | P11E11 | 558 | GH204345 |
| 831 | P11F1 | 333 | GH204347 |
| 832 | P11F2 | 650 | GH204348 |
| 833 | P11F3 | 652 | GH204349 |
| 834 | P11F4 | 538 | GH204350 |
| 835 | P11F5 | 524 | GH204351 |
| 836 | P11F6 | 495 | GH204352 |
| 837 | P11F7 | 255 | GH204353 |
| 838 | P11F8 | 833 | GH204354 |
| 839 | P11F9 | 443 | GH204355 |
| 840 | P11F10 | 438 | GH204356 |
| 841 | P11F11 | 306 | GH204357 |
| 842 | P11F12 | 620 | GH204358 |
| 843 | P11G1 | 468 | GH204359 |
| 844 | P11G2 | 323 | GH204360 |
| 845 | P11G3 | 747 | GH204361 |
| 846 | P11G4 | 834 | GH204362 |
| 847 | P11G5 | 187 | GH204363 |
| 848 | P11G6 | 718 | GH204364 |
| 849 | P11G7 | 600 | GH204365 |
| 850 | P11G8 | 371 | GH204366 |
| 851 | P11G9 | 583 | GH204367 |
| 852 | P11G10 | 350 | GH204368 |
| 853 | P11G11 | 480 | GH204369 |
| 854 | P11G12 | 270 | GH204370 |
| 855 | P11H1 | 240 | GH204371 |
| 856 | P11H3 | 455 | GH204372 |
| 857 | P11H4 | 763 | GH204373 |
| 858 | P11H5 | 361 | GH204374 |
| 859 | P11H7 | 469 | GH204375 |
| 860 | P11H8 | 204 | GH204376 |
| 861 | P11H9 | 310 | GH204377 |
| 862 | P11H10 | 469 | GH204378 |
| 863 | P11H11 | 581 | GH204379 |
| 864 | P11H12 | 638 | GH204380 |
| 865 | P12A1 | 844 | GH204381 |
| 866 | P12A4 | 661 | GH204383 |
| 867 | P12A6 | 608 | GH204384 |
| 868 | P12A7 | 464 | GH204385 |
| 869 | P12A8 | 210 | GH204386 |
| 870 | P12A9 | 533 | GH204387 |
| 871 | P12A10 | 124 | GH204388 |
| 872 | P12A12 | 190 | GH204390 |
| 873 | P12B1 | 611 | GH204391 |
| 874 | P12B4 | 430 | GH204393 |
| 875 | P12B5 | 343 | GH204394 |
| 876 | P12B6 | 275 | GH204395 |
| 877 | P12B7 | 359 | GH204396 |
| 878 | P12B8 | 808 | GH204397 |
| 879 | P12B9 | 716 | GH204398 |
| 880 | P12B10 | 450 | GH204399 |
| 881 | P12B11 | 419 | GH204400 |
| 882 | P12B12 | 661 | GH204401 |
| 883 | P12C1 | 254 | GH204402 |
| 884 | P12C3 | 747 | GH204404 |
| 885 | P12C4 | 430 | GH204405 |
| 886 | P12C5 | 670 | GH204406 |
| 887 | P12C6 | 678 | GH204407 |
| 888 | P12C7 | 411 | GH204408 |
| 889 | P12C8 | 549 | GH204409 |
| 890 | P12C9 | 332 | GH204410 |
| 891 | P12C10 | 556 | GH204411 |
| 892 | P12C12 | 229 | GH204412 |
| 893 | P12D1 | 530 | GH204413 |
| 894 | P12D2 | 538 | GH204414 |
| 895 | P12D3 | 819 | GH204415 |
| 896 | P12D4 | 331 | GH204416 |
| 897 | P12D5 | 798 | GH204417 |
| 898 | P12D6 | 141 | GH204418 |
| 899 | P12D9 | 443 | GH204420 |
| 900 | P12D10 | 441 | GH204421 |
| 901 | P12D11 | 779 | GH204422 |
| 902 | P12E2 | 274 | GH204424 |
| 903 | P12E3 | 499 | GH204425 |
| 904 | P12E5 | 184 | GH204427 |
| 905 | P12E7 | 398 | GH204429 |
| 906 | P12E8 | 298 | GH204430 |
| 907 | P12E9 | 103 | GH204431 |
| 908 | P12E11 | 420 | GH204433 |
| 909 | P12E12 | 518 | GH204434 |
| 910 | P12F1 | 788 | GH204435 |
| 911 | P12F2 | 203 | GH204436 |
| 912 | P12F3 | 593 | GH204437 |
| 913 | P12F4 | 411 | GH204438 |
| 914 | P12F6 | 723 | GH204439 |
| 915 | P12F7 | 683 | GH204440 |
| 916 | P12F8 | 644 | GH204441 |
| 917 | P12F9 | 311 | GH204442 |
| 918 | P12F10 | 473 | GH204443 |
| 919 | P12F11 | 597 | GH204444 |
| 920 | P12F12 | 630 | GH204445 |
| 921 | P12G2 | 437 | GH204446 |
| 922 | P12G3 | 712 | GH204447 |
| 923 | P12G4 | 630 | GH204448 |
| 924 | P12G5 | 301 | GH204449 |
| 925 | P12G6 | 815 | GH204450 |
| 926 | P12G7 | 736 | GH204451 |
| 927 | P12G8 | 664 | GH204452 |
| 928 | P12G9 | 325 | GH204453 |
| 929 | P12G10 | 134 | GH204454 |
| 930 | P12H2 | 790 | GH204457 |
| 931 | P12H3 | 428 | GH204458 |
| 932 | P12H4 | 245 | GH204459 |
| 933 | P12H5 | 466 | GH204460 |
| 934 | P12H6 | 432 | GH204461 |
| 935 | P12H7 | 637 | GH204462 |
| 936 | P12H8 | 901 | GH204463 |
| 937 | P12H9 | 729 | GH204464 |
| 938 | P12H10 | 518 | GH204465 |
| 939 | P12H11 | 689 | GH204466 |
| 940 | P12H12 | 423 | GH204467 |
| 941 | P13A2 | 690 | GH204469 |
| 942 | P13A3 | 583 | GH204470 |
| 943 | P13A4 | 765 | GH204471 |
| 944 | P13A5 | 498 | GH204472 |
| 945 | P13A6 | 700 | GH204473 |
| 946 | P13A7 | 268 | GH204474 |
| 947 | P13A8 | 283 | GH204475 |
| 948 | P13A10 | 273 | GH204477 |
| 949 | P13A11 | 316 | GH204478 |
| 950 | P13A12 | 615 | GH204479 |
| 951 | P13B1 | 227 | GH204480 |
| 952 | P13B2 | 406 | GH204481 |
| 953 | P13B3 | 323 | GH204482 |
| 954 | P13B5 | 420 | GH204483 |
| 955 | P13B6 | 383 | GH204484 |
| 956 | P13B9 | 797 | GH204487 |
| 957 | P13B10 | 405 | GH204488 |
| 958 | P13B12 | 352 | GH204489 |
| 959 | P13C1 | 260 | GH204490 |
| 960 | P13C2 | 803 | GH204491 |
| 961 | P13C3 | 406 | GH204492 |
| 962 | P13C5 | 340 | GH204493 |
| 963 | P13C6 | 816 | GH204494 |
| 964 | P13C7 | 422 | GH204495 |
| 965 | P13C8 | 694 | GH204496 |
| 966 | P13C10 | 179 | GH204498 |
| 967 | P13C11 | 635 | GH204499 |
| 968 | P13C12 | 483 | GH204500 |
| 969 | P13D2 | 508 | GH204502 |
| 970 | P13D3 | 347 | GH204503 |
| 971 | P13D4 | 672 | GH204504 |
| 972 | P13D5 | 253 | GH204505 |
| 973 | P13D7 | 463 | GH204507 |
| 974 | P13D8 | 415 | GH204508 |
| 975 | P13D10 | 772 | GH204509 |
| 976 | P13D11 | 513 | GH204510 |
| 977 | P13E1 | 139 | GH204512 |
| 978 | P13E2 | 385 | GH204513 |
| 979 | P13E3 | 512 | GH204514 |
| 980 | P13E4 | 569 | GH204515 |
| 981 | P13E5 | 370 | GH204516 |
| 982 | P13E6 | 450 | GH204517 |
| 983 | P13E7 | 250 | GH204518 |
| 984 | P13E8 | 386 | GH204519 |
| 985 | P13E10 | 213 | GH204521 |
| 986 | P13E11 | 479 | GH204522 |
| 987 | P13E12 | 456 | GH204523 |
| 988 | P13F1 | 578 | GH204524 |
| 989 | P13F2 | 390 | GH204525 |
| 990 | P13F3 | 291 | GH204526 |
| 991 | P13F5 | 546 | GH204528 |
| 992 | P13F6 | 776 | GH204529 |
| 993 | P13F7 | 121 | GH204530 |
| 994 | P13F8 | 181 | GH204531 |
| 995 | P13F9 | 291 | GH204532 |
| 996 | P13F10 | 391 | GH204533 |
| 997 | P13F11 | 735 | GH204534 |
| 998 | P13F12 | 756 | GH204535 |
| 999 | P13G1 | 353 | GH204536 |
| 1000 | P13G2 | 780 | GH204537 |
| 1001 | P13G3 | 454 | GH204538 |
| 1002 | P13G4 | 283 | GH204539 |
| 1003 | P13G6 | 228 | GH204541 |
| 1004 | P13G7 | 416 | GH204542 |
| 1005 | P13G8 | 150 | GH204543 |
| 1006 | P13G9 | 714 | GH204544 |
| 1007 | P13G10 | 660 | GH204545 |
| 1008 | P13G12 | 813 | GH204547 |
| 1009 | P13H1 | 572 | GH204548 |
| 1010 | P13H2 | 373 | GH204549 |
| 1011 | P13H3 | 455 | GH204550 |
| 1012 | P13H5 | 675 | GH204551 |
| 1013 | P13H6 | 212 | GH204552 |
| 1014 | P13H7 | 635 | GH204553 |
| 1015 | P13H8 | 485 | GH204554 |
| 1016 | P13H10 | 325 | GH204555 |
| 1017 | P13H11 | 385 | GH204556 |
| 1018 | P13H12 | 496 | GH204557 |
| 1019 | P14A3 | 210 | GH204559 |
| 1020 | P14A4 | 466 | GH204560 |
| 1021 | P14A5 | 187 | GH204561 |
| 1022 | P14A6 | 421 | GH204562 |
| 1023 | P14A7 | 711 | GH204563 |
| 1024 | P14A8 | 762 | GH204564 |
| 1025 | P14A10 | 211 | GH204565 |
| 1026 | P14A11 | 256 | GH204566 |
| 1027 | P14A12 | 810 | GH204567 |
| 1028 | P14B2 | 709 | GH204569 |
| 1029 | P14B3 | 202 | GH204570 |
| 1030 | P14B4 | 123 | GH204571 |
| 1031 | P14B5 | 690 | GH204572 |
| 1032 | P14B6 | 186 | GH204573 |
| 1033 | P14B7 | 232 | GH204574 |
| 1034 | P14B8 | 391 | GH204575 |
| 1035 | P14B9 | 660 | GH204576 |
| 1036 | P14B10 | 571 | GH204577 |
| 1037 | P14B11 | 529 | GH204578 |
| 1038 | P14B12 | 262 | GH204579 |
| 1039 | P14C2 | 798 | GH204580 |
| 1040 | P14C3 | 363 | GH204581 |
| 1041 | P14C5 | 311 | GH204583 |
| 1042 | P14C6 | 497 | GH204584 |
| 1043 | P14C8 | 677 | GH204586 |
| 1044 | P14C9 | 657 | GH204587 |
| 1045 | P14C10 | 786 | GH204588 |
| 1046 | P14C12 | 681 | GH204589 |
| 1047 | P14D1 | 612 | GH204590 |
| 1048 | P14D2 | 181 | GH204591 |
| 1049 | P14D4 | 357 | GH204592 |
| 1050 | P14D5 | 163 | GH204593 |
| 1051 | P14D7 | 711 | GH204595 |
| 1052 | P14D8 | 806 | GH204596 |
| 1053 | P14D9 | 407 | GH204597 |
| 1054 | P14D10 | 537 | GH204598 |
| 1055 | P14D11 | 673 | GH204599 |
| 1056 | P14D12 | 291 | GH204600 |
| 1057 | P14E1 | 321 | GH204601 |
| 1058 | P14E2 | 388 | GH204602 |
| 1059 | P14E3 | 509 | GH204603 |
| 1060 | P14E4 | 571 | GH204604 |
| 1061 | P14E5 | 377 | GH204605 |
| 1062 | P14E6 | 558 | GH204606 |
| 1063 | P14E7 | 463 | GH204607 |
| 1064 | P14E8 | 806 | GH204608 |
| 1065 | P14E9 | 179 | GH204609 |
| 1066 | P14E10 | 313 | GH204610 |
| 1067 | P14E11 | 406 | GH204611 |
| 1068 | P14E12 | 600 | GH204612 |
| 1069 | P14F1 | 526 | GH204613 |
| 1070 | P14F2 | 638 | GH204614 |
| 1071 | P14F3 | 525 | GH204615 |
| 1072 | P14F4 | 434 | GH204616 |
| 1073 | P14F5 | 456 | GH204617 |
| 1074 | P14F6 | 344 | GH204618 |
| 1075 | P14F7 | 213 | GH204619 |
| 1076 | P14F8 | 583 | GH204620 |
| 1077 | P14F9 | 495 | GH204621 |
| 1078 | P14F10 | 407 | GH204622 |
| 1079 | P14F11 | 757 | GH204623 |
| 1080 | P14G1 | 493 | GH204625 |
| 1081 | P14G2 | 532 | GH204626 |
| 1082 | P14G3 | 172 | GH204627 |
| 1083 | P14G4 | 341 | GH204628 |
| 1084 | P14G5 | 754 | GH204629 |
| 1085 | P14G6 | 501 | GH204630 |
| 1086 | P14G8 | 619 | GH204632 |
| 1087 | P14G9 | 297 | GH204633 |
| 1088 | P14G10 | 299 | GH204634 |
| 1089 | P14G12 | 322 | GH204636 |
| 1090 | P14H2 | 504 | GH204638 |
| 1091 | P14H3 | 532 | GH204639 |
| 1092 | P14H4 | 736 | GH204640 |
| 1093 | P14H5 | 387 | GH204641 |
| 1094 | P14H6 | 403 | GH204642 |
| 1095 | P14H9 | 694 | GH204644 |
| 1096 | P14H10 | 232 | GH204645 |
| 1097 | P14H11 | 699 | GH204646 |
| 1098 | P14H12 | 259 | GH204647 |
| 1099 | P15A1 | 452 | GH204648 |
| 1100 | P15A2 | 169 | GH204649 |
| 1101 | P15A3 | 555 | GH204650 |
| 1102 | P15A5 | 485 | GH204651 |
| 1103 | P15A6 | 481 | GH204652 |
| 1104 | P15A7 | 122 | GH204653 |
| 1105 | P15A8 | 231 | GH204654 |
| 1106 | P15A9 | 672 | GH204655 |
| 1107 | P15A10 | 375 | GH204656 |
| 1108 | P15A11 | 201 | GH204657 |
| 1109 | P15A12 | 559 | GH204658 |
| 1110 | P15B1 | 302 | GH204659 |
| 1111 | P15B2 | 781 | GH204660 |
| 1112 | P15B5 | 178 | GH204662 |
| 1113 | P15B6 | 603 | GH204663 |
| 1114 | P15B7 | 581 | GH204664 |
| 1115 | P15B8 | 582 | GH204665 |
| 1116 | P15B9 | 351 | GH204666 |
| 1117 | P15B11 | 556 | GH204668 |
| 1118 | P15B12 | 210 | GH204669 |
| 1119 | P15C1 | 246 | GH204670 |
| 1120 | P15C2 | 519 | GH204671 |
| 1121 | P15C3 | 779 | GH204672 |
| 1122 | P15C4 | 737 | GH204673 |
| 1123 | P15C5 | 437 | GH204674 |
| 1124 | P15C6 | 333 | GH204675 |
| 1125 | P15C7 | 682 | GH204676 |
| 1126 | P15C9 | 333 | GH204677 |
| 1127 | P15C10 | 653 | GH204678 |
| 1128 | P15C11 | 525 | GH204679 |
| 1129 | P15C12 | 461 | GH204680 |
| 1130 | P15D2 | 609 | GH204682 |
| 1131 | P15D3 | 731 | GH204683 |
| 1132 | P15D4 | 103 | GH204684 |
| 1133 | P15D5 | 678 | GH204685 |
| 1134 | P15D6 | 826 | GH204686 |
| 1135 | P15D8 | 192 | GH204687 |
| 1136 | P15D9 | 808 | GH204688 |
| 1137 | P14510 | 591 | GH204689 |
| 1138 | P14511 | 293 | GH204690 |
| 1139 | P15E1 | 651 | GH204691 |
| 1140 | P15E2 | 254 | GH204692 |
| 1141 | P15E4 | 626 | GH204693 |
| 1142 | P15E5 | 214 | GH204694 |
| 1143 | P15E6 | 282 | GH204695 |
| 1144 | P15E7 | 362 | GH204696 |
| 1145 | P15E8 | 380 | GH204697 |
| 1146 | P15E9 | 756 | GH204698 |
| 1147 | P15E10 | 425 | GH204699 |
| 1148 | P15E11 | 602 | GH204700 |
| 1149 | P15E12 | 462 | GH204701 |
| 1150 | P15F1 | 377 | GH204702 |
| 1151 | P15F2 | 650 | GH204703 |
| 1152 | P15F3 | 591 | GH204704 |
| 1153 | P15F6 | 792 | GH204706 |
| 1154 | P15F7 | 135 | GH204707 |
| 1155 | P15F8 | 675 | GH204708 |
| 1156 | P15F9 | 643 | GH204709 |
| 1157 | P15F10 | 514 | GH204710 |
| 1158 | P15F11 | 567 | GH204711 |
| 1159 | P15F12 | 662 | GH204712 |
| 1160 | P15G1 | 419 | GH204713 |
| 1161 | P15G2 | 574 | GH204714 |
| 1162 | P15G3 | 503 | GH204715 |
| 1163 | P15G4 | 387 | GH204716 |
| 1164 | P15G5 | 707 | GH204717 |
| 1165 | P15G6 | 502 | GH204718 |
| 1166 | P15G7 | 327 | GH204719 |
| 1167 | P15G8 | 161 | GH204720 |
| 1168 | P15G9 | 669 | GH204721 |
| 1169 | P15G10 | 319 | GH204722 |
| 1170 | P15G11 | 286 | GH204723 |
| 1171 | P15G12 | 639 | GH204724 |
| 1172 | P15H1 | 259 | GH204725 |
| 1173 | P15H2 | 593 | GH204726 |
| 1174 | P15H3 | 503 | GH204727 |
| 1175 | P15H4 | 228 | GH204728 |
| 1176 | P15H5 | 254 | GH204729 |
| 1177 | P15H6 | 758 | GH204730 |
| 1178 | P15H7 | 770 | GH204731 |
| 1179 | P15H10 | 381 | GH204733 |
| 1180 | P15H11 | 529 | GH204734 |
| 1181 | P15H12 | 1124 | GH204735 |
| 1182 | P16A1 | 567 | GH204736 |
| 1183 | P16A2 | 767 | GH204737 |
| 1184 | P16A3 | 271 | GH204738 |
| 1185 | P16A4 | 760 | GH204739 |
| 1186 | P16A6 | 287 | GH204740 |
| 1187 | P16A7 | 688 | GH204741 |
| 1188 | P16A8 | 164 | GH204742 |
| 1189 | P16A10 | 419 | GH204744 |
| 1190 | P16A11 | 628 | GH204745 |
| 1191 | P16A12 | 510 | GH204746 |
| 1192 | P16B1 | 668 | GH204747 |
| 1193 | P16B2 | 374 | GH204748 |
| 1194 | P16B3 | 413 | GH204749 |
| 1195 | P16B5 | 877 | GH204750 |
| 1196 | P16B6 | 543 | GH204751 |
| 1197 | P16B7 | 552 | GH204752 |
| 1198 | P16B9 | 385 | GH204754 |
| 1199 | P16B10 | 212 | GH204755 |
| 1200 | P16B11 | 309 | GH204756 |
| 1201 | P16C1 | 518 | GH204758 |
| 1202 | P16C2 | 718 | GH204759 |
| 1203 | P16 C3 | 449 | GH204760 |
| 1204 | P16C4 | 700 | GH204761 |
| 1205 | P16C5 | 471 | GH204762 |
| 1206 | P16C6 | 353 | GH204763 |
| 1207 | P16C7 | 782 | GH204764 |
| 1208 | P16C8 | 401 | GH204765 |
| 1209 | P16C9 | 329 | GH204766 |
| 1210 | P16C10 | 450 | GH204767 |
| 1211 | P16C11 | 122 | GH204768 |
| 1212 | P16C12 | 203 | GH204769 |
| 1213 | P16D1 | 406 | GH204770 |
| 1214 | P16D2 | 406 | GH204771 |
| 1215 | P16D3 | 617 | GH204772 |
| 1216 | P16D4 | 183 | GH204773 |
| 1217 | P16D5 | 476 | GH204774 |
| 1218 | P16D7 | 386 | GH204776 |
| 1219 | P16D8 | 413 | GH204777 |
| 1220 | P16D9 | 112 | GH204778 |
| 1221 | P16D10 | 591 | GH204779 |
| 1222 | P16D11 | 292 | GH204780 |
| 1223 | P16D12 | 427 | GH204781 |
| 1224 | P16E2 | 175 | GH204783 |
| 1225 | P16E4 | 607 | GH204784 |
| 1226 | P16E5 | 421 | GH204785 |
| 1227 | P16E6 | 255 | GH204786 |
| 1228 | P16E7 | 511 | GH204787 |
| 1229 | P16E8 | 421 | GH204788 |
| 1230 | P16E10 | 108 | GH204790 |
| 1231 | P16E11 | 189 | GH204791 |
| 1232 | P16E12 | 560 | GH204792 |
| 1233 | P16F1 | 517 | GH204793 |
| 1234 | P16F2 | 406 | GH204794 |
| 1235 | P16F3 | 854 | GH204795 |
| 1236 | P16F4 | 236 | GH204796 |
| 1237 | P16F5 | 315 | GH204797 |
| 1238 | P16F6 | 332 | GH204798 |
| 1239 | P16F7 | 478 | GH204799 |
| 1240 | P16F8 | 605 | GH204800 |
| 1241 | P16F9 | 103 | GH204801 |
| 1242 | P16F10 | 190 | GH204802 |
| 1243 | P16F11 | 567 | GH204803 |
| 1244 | P16F12 | 332 | GH204804 |
| 1245 | P16G1 | 172 | GH204805 |
| 1246 | P16G2 | 356 | GH204806 |
| 1247 | P16G3 | 569 | GH204807 |
| 1248 | P16G4 | 472 | GH204808 |
| 1249 | P16G5 | 276 | GH204809 |
| 1250 | P16G6 | 788 | GH204810 |
| 1251 | P16G7 | 791 | GH204811 |
| 1252 | P16G8 | 176 | GH204812 |
| 1253 | P16G9 | 381 | GH204813 |
| 1254 | P16G10 | 583 | GH204814 |
| 1255 | P16G12 | 415 | GH204815 |
| 1256 | P16H1 | 896 | GH204816 |
| 1257 | P16H2 | 911 | GH204817 |
| 1258 | P16H4 | 632 | GH204819 |
| 1259 | P16H5 | 548 | GH204820 |
| 1260 | P16H6 | 231 | GH204821 |
| 1261 | P16H7 | 713 | GH204822 |
| 1262 | P16H9 | 565 | GH204823 |
| 1263 | P16H10 | 969 | GH204824 |
| 1264 | P16H11 | 615 | GH204825 |
| 1265 | P16H12 | 723 | GH204826 |
| 1266 | P17A1 | 707 | GH204827 |
| 1267 | P17A3 | 485 | GH204829 |
| 1268 | P17A4 | 466 | GH204830 |
| 1269 | P17A5 | 317 | GH204831 |
| 1270 | P17A6 | 619 | GH204832 |
| 1271 | P17A7 | 785 | GH204833 |
| 1272 | P17A8 | 273 | GH204834 |
| 1273 | P17A10 | 849 | GH204836 |
| 1274 | P17A11 | 646 | GH204837 |
| 1275 | P17A12 | 716 | GH204838 |
| 1276 | P17B3 | 690 | GH204839 |
| 1277 | P17B4 | 211 | GH204840 |
| 1278 | P17B6 | 640 | GH204841 |
| 1279 | P17B8 | 787 | GH204842 |
| 1280 | P17B9 | 471 | GH204843 |
| 1281 | P17B10 | 753 | GH204844 |
| 1282 | P17B11 | 528 | GH204845 |
| 1283 | P17B12 | 734 | GH204846 |
| 1284 | P17C1 | 494 | GH204847 |
| 1285 | P17C3 | 122 | GH204849 |
| 1286 | P17C4 | 211 | GH204850 |
| 1287 | P17C5 | 438 | GH204851 |
| 1288 | P17C6 | 432 | GH204852 |
| 1289 | P17C7 | 604 | GH204853 |
| 1290 | P17C10 | 413 | GH204856 |
| 1291 | P17C11 | 820 | GH204857 |
| 1292 | P17C12 | 827 | GH204858 |
| 1293 | P17D1 | 569 | GH204859 |
| 1294 | P17D2 | 404 | GH204860 |
| 1295 | P17D3 | 351 | GH204861 |
| 1296 | P17D5 | 460 | GH204862 |
| 1297 | P17D6 | 479 | GH204863 |
| 1298 | P17D7 | 173 | GH204864 |
| 1299 | P17D8 | 690 | GH204865 |
| 1300 | P17D10 | 789 | GH204867 |
| 1301 | P17D11 | 602 | GH204868 |
| 1302 | P17D12 | 794 | GH204869 |
| 1303 | P17E1 | 835 | GH204870 |
| 1304 | P17E2 | 612 | GH204871 |
| 1305 | P17E3 | 217 | GH204872 |
| 1306 | P17E4 | 236 | GH204873 |
| 1307 | P17E5 | 459 | GH204874 |
| 1308 | P17E9 | 228 | GH204877 |
| 1309 | P17E11 | 253 | GH204879 |
| 1310 | P17E12 | 815 | GH204880 |
| 1311 | P17F1 | 433 | GH204881 |
| 1312 | P17F2 | 231 | GH204882 |
| 1313 | P17F3 | 495 | GH204883 |
| 1314 | P17F5 | 235 | GH204884 |
| 1315 | P17F6 | 565 | GH204885 |
| 1316 | P17F7 | 757 | GH204886 |
| 1317 | P17F8 | 463 | GH204887 |
| 1318 | P17F9 | 540 | GH204888 |
| 1319 | P17F10 | 363 | GH204889 |
| 1320 | P17F11 | 569 | GH204890 |
| 1321 | P17F12 | 196 | GH204891 |
| 1322 | P17G3 | 495 | GH204893 |
| 1323 | P17G4 | 477 | GH204894 |
| 1324 | P17G5 | 344 | GH204895 |
| 1325 | P17G7 | 459 | GH204896 |
| 1326 | P17G10 | 500 | GH204898 |
| 1327 | P17G12 | 760 | GH204900 |
| 1328 | P17H3 | 131 | GH204902 |
| 1329 | P17H5 | 489 | GH204903 |
| 1330 | P17H7 | 265 | GH204905 |
| 1331 | P17H8 | 753 | GH204906 |
| 1332 | P17H10 | 571 | GH204907 |
| 1333 | P17H11 | 202 | GH204908 |
| 1334 | P17H12 | 639 | GH204909 |
| 1335 | P18A1 | 518 | GH204910 |
| 1336 | P18A2 | 455 | GH204911 |
| 1337 | P18A3 | 329 | GH204912 |
| 1338 | P18A4 | 552 | GH204913 |
| 1339 | P18A6 | 306 | GH204914 |
| 1340 | P18A7 | 591 | GH204915 |
| 1341 | P18A8 | 653 | GH204916 |
| 1342 | P18A9 | 256 | GH204917 |
| 1343 | P18A10 | 580 | GH204918 |
| 1344 | P18A11 | 449 | GH204919 |
| 1345 | P18B1 | 301 | GH204920 |
| 1346 | P18B2 | 120 | GH204921 |
| 1347 | P18B4 | 569 | GH204923 |
| 1348 | P18B5 | 599 | GH204924 |
| 1349 | P18B6 | 569 | GH204925 |
| 1350 | P18B7 | 321 | GH204926 |
| 1351 | P18B8 | 382 | GH204927 |
| 1352 | P18B9 | 768 | GH204928 |
| 1353 | P18B11 | 478 | GH204930 |
| 1354 | P18B12 | 122 | GH204931 |
| 1355 | P18C1 | 276 | GH204932 |
| 1356 | P18C3 | 360 | GH204934 |
| 1357 | P18C4 | 278 | GH204935 |
| 1358 | P18C5 | 651 | GH204936 |
| 1359 | P18C6 | 596 | GH204937 |
| 1360 | P18C7 | 485 | GH204938 |
| 1361 | P18C8 | 786 | GH204939 |
| 1362 | P18C9 | 705 | GH204940 |
| 1363 | P18C10 | 339 | GH204941 |
| 1364 | P18C11 | 796 | GH204942 |
| 1365 | P18C12 | 491 | GH204943 |
| 1366 | P18D1 | 201 | GH204944 |
| 1367 | P18D2 | 349 | GH204945 |
| 1368 | P18D3 | 513 | GH204946 |
| 1369 | P18D4 | 147 | GH204947 |
| 1370 | P18D5 | 430 | GH204948 |
| 1371 | P18D6 | 583 | GH204949 |
| 1372 | P18D7 | 362 | GH204950 |
| 1373 | P18D8 | 884 | GH204951 |
| 1374 | P18D9 | 208 | GH204952 |
| 1375 | P18D10 | 546 | GH204953 |
| 1376 | P18D11 | 484 | GH204954 |
| 1377 | P18D12 | 305 | GH204955 |
| 1378 | P18E1 | 340 | GH204956 |
| 1379 | P18E2 | 680 | GH204957 |
| 1380 | P18E3 | 239 | GH204958 |
| 1381 | P18E5 | 611 | GH204959 |
| 1382 | P18E6 | 506 | GH204960 |
| 1383 | P18E7 | 583 | GH204961 |
| 1384 | P18E8 | 192 | GH204962 |
| 1385 | P18E9 | 445 | GH204963 |
| 1386 | P18E10 | 513 | GH204964 |
| 1387 | P18E11 | 349 | GH204965 |
| 1388 | P18E12 | 747 | GH204966 |
| 1389 | P18F1 | 378 | GH204967 |
| 1390 | P18F2 | 594 | GH204968 |
| 1391 | P18F3 | 442 | GH204969 |
| 1392 | P18F4 | 259 | GH204970 |
| 1393 | P18F5 | 324 | GH204971 |
| 1394 | P18F6 | 432 | GH204972 |
| 1395 | P18F7 | 699 | GH204973 |
| 1396 | P18F9 | 229 | GH204975 |
| 1397 | P18F10 | 127 | GH204976 |
| 1398 | P18F11 | 311 | GH204977 |
| 1399 | P18G1 | 434 | GH204978 |
| 1400 | P18G2 | 758 | GH204979 |
| 1401 | P18G3 | 233 | GH204980 |
| 1402 | P18G4 | 584 | GH204981 |
| 1403 | P18G5 | 602 | GH204982 |
| 1404 | P18G6 | 473 | GH204983 |
| 1405 | P18G7 | 682 | GH204984 |
| 1406 | P18G8 | 543 | GH204985 |
| 1407 | P18G9 | 426 | GH204986 |
| 1408 | P18G11 | 235 | GH204987 |
| 1409 | P18G12 | 576 | GH204988 |
| 1410 | P18H2 | 460 | GH204989 |
| 1411 | P18H3 | 370 | GH204990 |
| 1412 | P18H4 | 491 | GH204991 |
| 1413 | P18H6 | 101 | GH204993 |
| 1414 | P18H7 | 409 | GH204994 |
| 1415 | P18H9 | 552 | GH204996 |
| 1416 | P18H10 | 184 | GH204997 |
| 1417 | P18H11 | 745 | GH204998 |
| 1418 | P18H12 | 638 | GH204999 |
| 1419 | P19A1 | 473 | GH205000 |
| 1420 | P19A2 | 821 | GH205001 |
| 1421 | P19A3 | 564 | GH205002 |
| 1422 | P19A5 | 587 | GH205003 |
| 1423 | P19A6 | 563 | GH205004 |
| 1424 | P19A7 | 709 | GH205005 |
| 1425 | P19A8 | 517 | GH205006 |
| 1426 | P19A9 | 292 | GH205007 |
| 1427 | P19A10 | 264 | GH205008 |
| 1428 | P19A11 | 714 | GH205009 |
| 1429 | P19A12 | 626 | GH205010 |
| 1430 | P19B1 | 313 | GH205011 |
| 1431 | P19B2 | 612 | GH205012 |
| 1432 | P19B3 | 367 | GH205013 |
| 1433 | P19B5 | 677 | GH205014 |
| 1434 | P19B6 | 182 | GH205015 |
| 1435 | P19B7 | 494 | GH205016 |
| 1436 | P19B8 | 476 | GH205017 |
| 1437 | P19B9 | 454 | GH205018 |
| 1438 | P19B10 | 468 | GH205019 |
| 1439 | P19B11 | 265 | GH205020 |
| 1440 | P19B12 | 787 | GH205021 |
| 1441 | P19C1 | 237 | GH205022 |
| 1442 | P19C2 | 261 | GH205023 |
| 1443 | P19C3 | 380 | GH205024 |
| 1444 | P19C4 | 183 | GH205025 |
| 1445 | P19C5 | 439 | GH205026 |
| 1446 | P19C6 | 758 | GH205027 |
| 1447 | P19C8 | 535 | GH205028 |
| 1448 | P19C9 | 785 | GH205029 |
| 1449 | P19C11 | 455 | GH205031 |
| 1450 | P19C12 | 216 | GH205032 |
| 1451 | P19D2 | 733 | GH205034 |
| 1452 | P19D3 | 293 | GH205035 |
| 1453 | P19D4 | 485 | GH205036 |
| 1454 | P19D5 | 618 | GH205037 |
| 1455 | P19D6 | 798 | GH205038 |
| 1456 | P19D7 | 293 | GH205039 |
| 1457 | P19D8 | 752 | GH205040 |
| 1458 | P19D9 | 376 | GH205041 |
| 1459 | P19D11 | 764 | GH205043 |
| 1460 | P19D12 | 813 | GH205044 |
| 1461 | P19E1 | 502 | GH205045 |
| 1462 | P19E2 | 390 | GH205046 |
| 1463 | P19E5 | 143 | GH205048 |
| 1464 | P19E7 | 773 | GH205050 |
| 1465 | P19E8 | 295 | GH205051 |
| 1466 | P19E10 | 749 | GH205053 |
| 1467 | P19E11 | 198 | GH205054 |
| 1468 | P19E12 | 758 | GH205055 |
| 1469 | P19F1 | 543 | GH205056 |
| 1470 | P19F2 | 762 | GH205057 |
| 1471 | P19F3 | 219 | GH205058 |
| 1472 | P19F5 | 628 | GH205059 |
| 1473 | P19F6 | 480 | GH205060 |
| 1474 | P19F7 | 403 | GH205061 |
| 1475 | P19F8 | 536 | GH205062 |
| 1476 | P19F9 | 693 | GH205063 |
| 1477 | P19F10 | 220 | GH205064 |
| 1478 | P19F11 | 523 | GH205065 |
| 1479 | P19F12 | 453 | GH205066 |
| 1480 | P19G1 | 472 | GH205067 |
| 1481 | P19G2 | 612 | GH205068 |
| 1482 | P19G3 | 472 | GH205069 |
| 1483 | P19G4 | 603 | GH205070 |
| 1484 | P19G5 | 788 | GH205071 |
| 1485 | P19G6 | 403 | GH205072 |
| 1486 | P19G7 | 403 | GH205073 |
| 1487 | P19G8 | 419 | GH205074 |
| 1488 | P19G9 | 273 | GH205075 |
| 1489 | P19G10 | 390 | GH205076 |
| 1490 | P19G11 | 640 | GH205077 |
| 1491 | P19G12 | 178 | GH205078 |
| 1492 | P19H1 | 764 | GH205079 |
| 1493 | P19H2 | 481 | GH205080 |
| 1494 | P19H3 | 755 | GH205081 |
| 1495 | P19H4 | 391 | GH205082 |
| 1496 | P19H5 | 617 | GH205083 |
| 1497 | P19H6 | 667 | GH205084 |
| 1498 | P19H7 | 342 | GH205085 |
| 1499 | P19H8 | 542 | GH205086 |
| 1500 | P19H9 | 677 | GH205087 |
| 1501 | P19H10 | 227 | GH205088 |
| 1502 | P19H11 | 548 | GH205089 |
| 1503 | P19H12 | 795 | GH205090 |
| 1504 | P20A1 | 351 | GH205091 |
| 1505 | P20A2 | 345 | GH205092 |
| 1506 | P20A3 | 239 | GH205093 |
| 1507 | P20A5 | 392 | GH205095 |
| 1508 | P20A6 | 396 | GH205096 |
| 1509 | P20A7 | 709 | GH205097 |
| 1510 | P20A8 | 435 | GH205098 |
| 1511 | P20A9 | 279 | GH205099 |
| 1512 | P20A11 | 684 | GH205100 |
| 1513 | P20B1 | 515 | GH205101 |
| 1514 | P20B2 | 612 | GH205102 |
| 1515 | P20B4 | 741 | GH205104 |
| 1516 | P20B5 | 518 | GH205105 |
| 1517 | P20B6 | 177 | GH205106 |
| 1518 | P20B7 | 238 | GH205107 |
| 1519 | P20B8 | 386 | GH205108 |
| 1520 | P20B9 | 686 | GH205109 |
| 1521 | P20B10 | 453 | GH205110 |
| 1522 | P20B11 | 568 | GH205111 |
| 1523 | P20B12 | 842 | GH205112 |
| 1524 | P20C1 | 249 | GH205113 |
| 1525 | P20C3 | 630 | GH205115 |
| 1526 | P20C4 | 377 | GH205116 |
| 1527 | P20C5 | 584 | GH205117 |
| 1528 | P20C7 | 950 | GH205118 |
| 1529 | P20C8 | 381 | GH205119 |
| 1530 | P20C10 | 251 | GH205120 |
| 1531 | P20D4 | 228 | GH205121 |
| 1532 | P20D5 | 438 | GH205122 |
| 1533 | P20D6 | 761 | GH205123 |
| 1534 | P20D7 | 198 | GH205124 |
| 1535 | P20D8 | 749 | GH205125 |
| 1536 | P20D10 | 444 | GH205127 |
| 1537 | P20D11 | 577 | GH205128 |
| 1538 | P20D12 | 533 | GH205129 |
| 1539 | P20E1 | 734 | GH205130 |
| 1540 | P20E2 | 390 | GH205131 |
| 1541 | P20E3 | 566 | GH205132 |
| 1542 | P20E4 | 476 | GH205133 |
| 1543 | P20E5 | 724 | GH205134 |
| 1544 | P20E6 | 785 | GH205135 |
| 1545 | P20E7 | 765 | GH205136 |
| 1546 | P20E11 | 620 | GH205138 |
| 1547 | P20E12 | 530 | GH205139 |
| 1548 | P20F1 | 442 | GH205140 |
| 1549 | P20F2 | 449 | GH205141 |
| 1550 | P20F3 | 569 | GH205142 |
| 1551 | P20F4 | 322 | GH205143 |
| 1552 | P20F5 | 573 | GH205144 |
| 1553 | P20F7 | 802 | GH205146 |
| 1554 | P20F8 | 569 | GH205147 |
| 1555 | P20F9 | 291 | GH205148 |
| 1556 | P20F10 | 217 | GH205149 |
| 1557 | P20F11 | 311 | GH205150 |
| 1558 | P20G1 | 858 | GH205151 |
| 1559 | P20G2 | 330 | GH205152 |
| 1560 | P20G3 | 782 | GH205153 |
| 1561 | P20G6 | 798 | GH205154 |
| 1562 | P20G7 | 772 | GH205155 |
| 1563 | P20G8 | 313 | GH205156 |
| 1564 | P20G9 | 273 | GH205157 |
| 1565 | P20G11 | 158 | GH205158 |
| 1566 | P20H1 | 766 | GH205159 |
| 1567 | P20H2 | 462 | GH205160 |
| 1568 | P20H3 | 480 | GH205161 |
| 1569 | P20H4 | 394 | GH205162 |
| 1570 | P20H5 | 296 | GH205163 |
| 1571 | P20H6 | 539 | GH205164 |
| 1572 | P20H8 | 175 | GH205166 |
| 1573 | P20H9 | 673 | GH205167 |
| 1574 | P20H11 | 158 | GH205169 |
| 1575 | P20H12 | 399 | GH205170 |
| 1576 | P21A1 | 472 | GH205171 |
| 1577 | P21A3 | 697 | GH205173 |
| 1578 | P21A4 | 396 | GH205174 |
| 1579 | P21A5 | 412 | GH205175 |
| 1580 | P21A6 | 216 | GH205176 |
| 1581 | P21A7 | 639 | GH205177 |
| 1582 | P21A8 | 560 | GH205178 |
| 1583 | P21A9 | 529 | GH205179 |
| 1584 | P21A11 | 691 | GH205180 |
| 1585 | P21A12 | 174 | GH205181 |
| 1586 | P21B1 | 550 | GH205182 |
| 1587 | P21B2 | 689 | GH205183 |
| 1588 | P21B3 | 790 | GH205184 |
| 1589 | P21B4 | 402 | GH205185 |
| 1590 | P21B6 | 513 | GH205186 |
| 1591 | P21B7 | 694 | GH205187 |
| 1592 | P21B8 | 385 | GH205188 |
| 1593 | P21B9 | 245 | GH205189 |
| 1594 | P21B10 | 711 | GH205190 |
| 1595 | P21B11 | 673 | GH205191 |
| 1596 | P21C1 | 560 | GH205192 |
| 1597 | P21C3 | 646 | GH205193 |
| 1598 | P21C4 | 259 | GH205194 |
| 1599 | P21C6 | 633 | GH205196 |
| 1600 | P21C7 | 615 | GH205197 |
| 1601 | P21C8 | 467 | GH205198 |
| 1602 | P21C9 | 361 | GH205199 |
| 1603 | P21C10 | 435 | GH205200 |
| 1604 | P21C11 | 178 | GH205201 |
| 1605 | P21C12 | 728 | GH205202 |
| 1606 | P21D2 | 680 | GH205203 |
| 1607 | P21D3 | 545 | GH205204 |
| 1608 | P21D4 | 313 | GH205205 |
| 1609 | P21D6 | 442 | GH205206 |
| 1610 | P21D7 | 339 | GH205207 |
| 1611 | P21D8 | 643 | GH205208 |
| 1612 | P21D9 | 548 | GH205209 |
| 1613 | P21D11 | 685 | GH205210 |
| 1614 | P21D12 | 351 | GH205211 |
| 1615 | P21E1 | 683 | GH205212 |
| 1616 | P21E2 | 477 | GH205213 |
| 1617 | P21E3 | 480 | GH205214 |
| 1618 | P21E5 | 203 | GH205216 |
| 1619 | P21E7 | 143 | GH205217 |
| 1620 | P21E8 | 748 | GH205218 |
| 1621 | P21E10 | 328 | GH205219 |
| 1622 | P21E11 | 670 | GH205220 |
| 1623 | P21E12 | 677 | GH205221 |
| 1624 | P21F1 | 407 | GH205222 |
| 1625 | P21F2 | 584 | GH205223 |
| 1626 | P21F3 | 633 | GH205224 |
| 1627 | P21F4 | 332 | GH205225 |
| 1628 | P21F5 | 604 | GH205226 |
| 1629 | P21F6 | 133 | GH205227 |
| 1630 | P21F9 | 287 | GH205228 |
| 1631 | P21F10 | 753 | GH205229 |
| 1632 | P21F11 | 283 | GH205230 |
| 1633 | P21F12 | 256 | GH205231 |
| 1634 | P21G1 | 831 | GH205232 |
| 1635 | P21G2 | 644 | GH205233 |
| 1636 | P21G3 | 218 | GH205234 |
| 1637 | P21G5 | 644 | GH205236 |
| 1638 | P21G6 | 121 | GH205237 |
| 1639 | P21G7 | 269 | GH205238 |
| 1640 | P21G9 | 365 | GH205240 |
| 1641 | P21G11 | 691 | GH205241 |
| 1642 | P21G12 | 607 | GH205242 |
| 1643 | P21H1 | 557 | GH205243 |
| 1644 | P21H2 | 407 | GH205244 |
| 1645 | P21H3 | 667 | GH205245 |
| 1646 | P21H5 | 419 | GH205246 |
| 1647 | P21H6 | 533 | GH205247 |
| 1648 | P21H7 | 385 | GH205248 |
| 1649 | P21H8 | 414 | GH205249 |
| 1650 | P21H9 | 277 | GH205250 |
| 1651 | P21H10 | 575 | GH205251 |
| 1652 | P21H11 | 529 | GH205252 |
| 1653 | P21H12 | 327 | GH205253 |
| 1654 | P22A1 | 259 | GH205254 |
| 1655 | P22A3 | 265 | GH205256 |
| 1656 | P22A4 | 634 | GH205257 |
| 1657 | P22A6 | 410 | GH205259 |
| 1658 | P22A7 | 450 | GH205260 |
| 1659 | P22A8 | 434 | GH205261 |
| 1660 | P22A10 | 349 | GH205262 |
| 1661 | P22A11 | 530 | GH205263 |
| 1662 | P22A12 | 453 | GH205264 |
| 1663 | P22B1 | 662 | GH205265 |
| 1664 | P22B2 | 658 | GH205266 |
| 1665 | P22B7 | 743 | GH205269 |
| 1666 | P22B11 | 313 | GH205271 |
| 1667 | P22B12 | 198 | GH205272 |
| 1668 | P22C1 | 166 | GH205273 |
| 1669 | P22C2 | 163 | GH205274 |
| 1670 | P22C3 | 350 | GH205275 |
| 1671 | P22C5 | 388 | GH205276 |
| 1672 | P22C6 | 575 | GH205277 |
| 1673 | P22C7 | 196 | GH205278 |
| 1674 | P22C8 | 786 | GH205279 |
| 1675 | P22C11 | 402 | GH205282 |
| 1676 | P22C12 | 640 | GH205283 |
| 1677 | P22D1 | 473 | GH205284 |
| 1678 | P22D2 | 600 | GH205285 |
| 1679 | P22D3 | 116 | GH205286 |
| 1680 | P22D4 | 377 | GH205287 |
| 1681 | P22D5 | 298 | GH205288 |
| 1682 | P22D6 | 219 | GH205289 |
| 1683 | P22D7 | 596 | GH205290 |
| 1684 | P22D9 | 395 | GH205291 |
| 1685 | P22D10 | 410 | GH205292 |
| 1686 | P22D12 | 422 | GH205293 |
| 1687 | P22E1 | 229 | GH205294 |
| 1688 | P22E2 | 407 | GH205295 |
| 1689 | P22E3 | 229 | GH205296 |
| 1690 | P22E4 | 699 | GH205297 |
| 1691 | P22E5 | 131 | GH205298 |
| 1692 | P22E6 | 762 | GH205299 |
| 1693 | P22E7 | 565 | GH205300 |
| 1694 | P22E8 | 706 | GH205301 |
| 1695 | P22E10 | 461 | GH205303 |
| 1696 | P22E11 | 468 | GH205304 |
| 1697 | P22F1 | 337 | GH205305 |
| 1698 | P22F2 | 407 | GH205306 |
| 1699 | P22F3 | 116 | GH205307 |
| 1700 | P22F4 | 449 | GH205308 |
| 1701 | P22F6 | 783 | GH205309 |
| 1702 | P22F7 | 374 | GH205310 |
| 1703 | P22F8 | 677 | GH205311 |
| 1704 | P22F9 | 341 | GH205312 |
| 1705 | P22F10 | 409 | GH205313 |
| 1706 | P22F11 | 245 | GH205314 |
| 1707 | P22G1 | 494 | GH205316 |
| 1708 | P22G2 | 554 | GH205317 |
| 1709 | P22G4 | 800 | GH205319 |
| 1710 | P22G6 | 189 | GH205321 |
| 1711 | P22G7 | 229 | GH205322 |
| 1712 | P22G8 | 713 | GH205323 |
| 1713 | P22G9 | 337 | GH205324 |
| 1714 | P22G11 | 330 | GH205325 |
| 1715 | P22G12 | 439 | GH205326 |
| 1716 | P22H2 | 462 | GH205328 |
| 1717 | P22H4 | 131 | GH205330 |
| 1718 | P22H5 | 654 | GH205331 |
| 1719 | P22H7 | 653 | GH205333 |
| 1720 | P22H9 | 301 | GH205335 |
| 1721 | P22H10 | 234 | GH205336 |
| 1722 | P22H11 | 245 | GH205337 |
| 1723 | P22H12 | 736 | GH205338 |
| 1724 | P23A1 | 496 | GH205339 |
| 1725 | P23A2 | 743 | GH205340 |
| 1726 | P23A3 | 371 | GH205341 |
| 1727 | P23A4 | 316 | GH205342 |
| 1728 | P23A6 | 110 | GH205343 |
| 1729 | P23A8 | 737 | GH205344 |
| 1730 | P23B1 | 215 | GH205346 |
| 1731 | P23B2 | 429 | GH205347 |
| 1732 | P23B3 | 496 | GH205348 |
| 1733 | P23B4 | 216 | GH205349 |
| 1734 | P23B5 | 515 | GH205350 |
| 1735 | P23B7 | 161 | GH205352 |
| 1736 | P23B8 | 524 | GH205353 |
| 1737 | P23B9 | 630 | GH205354 |
| 1738 | P23C1 | 429 | GH205355 |
| 1739 | P23C2 | 577 | GH205356 |
| 1740 | P23C5 | 365 | GH205359 |
| 1741 | P23C7 | 506 | GH205361 |
| 1742 | P23D1 | 429 | GH205364 |
| 1743 | P23D4 | 329 | GH205366 |
| 1744 | P23D7 | 369 | GH205368 |
| 1745 | P23D8 | 529 | GH205369 |
| 1746 | P23E1 | 678 | GH205371 |
| 1747 | P23E2 | 395 | GH205372 |
| 1748 | P23E4 | 311 | GH205374 |
| 1749 | P23E6 | 318 | GH205375 |
| 1750 | P23E7 | 333 | GH205376 |
| 1751 | P23E8 | 129 | GH205377 |
| 1752 | P23F1 | 724 | GH205378 |
| 1753 | P23F3 | 361 | GH205380 |
| 1754 | P23F6 | 777 | GH205383 |
| 1755 | P23F7 | 181 | GH205384 |
| 1756 | P23G1 | 288 | GH205386 |
| 1757 | P23G2 | 547 | GH205387 |
| 1758 | P23G5 | 747 | GH205388 |
| 1759 | P23G8 | 206 | GH205391 |
| 1760 | P23G9 | 415 | GH205392 |
| 1761 | P23H2 | 336 | GH205393 |
| 1762 | P23H3 | 554 | GH205394 |
| 1763 | P23H4 | 506 | GH205395 |
| 1764 | P23H5 | 550 | GH205396 |
| 1765 | P23H8 | 337 | GH205399 |
