# Supplementary material for: Induction of Senescence and Identification of Differentially Expressed Genes in Tomato in Response to Monoterpene
Source: PLoS One. 2013 Sep 30;8(9):e76029. doi: 10.1371/journal.pone.0076029 (PMC3786903; doi:10.1371/journal.pone.0076029)
Supplement: Table S2 — List of geraniol-responsive tomato unigenes (contigs and singletons). (DOC) [file pone.0076029.s005.doc]

**Table S2. List of geraniol-responsive tomato unigenes (contigs and singletons).**

| **Sl No** | **Unigene ID** | **Length (bp)** | **No of ESTs** | **GenBank Accession Number*** | **Functional annotation to tomato genome database [ITAG release 2.3 predicted proteins (SL2.40); www.solgenomics.net]** | **E-Value** |
| --- | --- | --- | --- | --- | --- | --- |
| 1 | Contig 1 | 382 | 13 | GH204806 | Solyc00g012440.1.1 evidence_code:10F0H0E0IEG genomic_reference:SL2.40ch00 gene_region:10511541-10511921 transcript_region:SL2.40ch00:10511541..10511921+ functional_description:"TO54-2 (Fragment) (AHRD V1 ***- Q3LVL5_TAROF)" | 2E-22 |
| 2 | Contig 2 | 1124 | 1 | GH204735 | ********* |  |
| 3 | Contig 3 | 918 | 6 | GH204792 | Solyc09g010630.2.1 genomic_reference:SL2.40ch09 gene_region:3965253-3968837 transcript_region:SL2.40ch09:3965253..3968837+ go_terms:GO:0051082 functional_description:"heat shock protein (AHRD V1 ***- B2D2G5_CAPSN); contains Interpro domain(s) IPR013126 Heat shock protein 70 " | 1E-109 |
| 4 | Contig 4 | 519 | 2 | GH204298 | Solyc01g103450.2.1 genomic_reference:SL2.40ch01 gene_region:83821528-83826037 transcript_region:SL2.40ch01:83821528..83826037+ go_terms:GO:0005524,GO:0006457 functional_description:"Chaperone DnaK (AHRD V1 ***- Q1SKX2_MEDTR); contains Interpro domain(s) IPR012725 Chaperone DnaK " | 1E-76 |
| 5 | Contig 5 | 969 | 1 | GH204824 | ********* |  |
| 6 | Contig 6 | 896 | 21 | GH204079 | Solyc09g010630.2.1 genomic_reference:SL2.40ch09 gene_region:3965253-3968837 transcript_region:SL2.40ch09:3965253..3968837+ go_terms:GO:0051082 functional_description:"heat shock protein (AHRD V1 ***- B2D2G5_CAPSN); contains Interpro domain(s) IPR013126 Heat shock protein 70 " | 1E-112 |
| 7 | Contig 7 | 245 | 2 | GH205314 | ********* |  |
| 8 | Contig 8 | 343 | 1 | GH204300 | Solyc11g066060.1.1 evidence_code:10F0H1E1IEG genomic_reference:SL2.40ch11 gene_region:48824058-48826931 transcript_region:SL2.40ch11:48824058..48826931+ go_terms:GO:0005524 functional_description:"heat shock protein (AHRD V1 ***- B2D2G5_CAPSN); contains Interpro domain(s) IPR013126 Heat shock protein 70 " | 3E-51 |
| 9 | Contig 9 | 329 | 1 | GH204766 | Solyc03g007740.2.1 genomic_reference:SL2.40ch03 gene_region:2271270-2273817 transcript_region:SL2.40ch03:2271270..2273817+ go_terms:GO:0005783 functional_description:"Reticulon family protein (AHRD V1 ***- B2WS91_9BRAS); contains Interpro domain(s) IPR003388 Reticulon " | 2E-57 |
| 10 | Contig 10 | 798 | 4 | GH204869 | Solyc08g076730.2.1 genomic_reference:SL2.40ch08 gene_region:57847867-57849297 transcript_region:SL2.40ch08:57847867..57849297- go_terms:GO:0005488 functional_description:"TPR domain protein (AHRD V1 *-*- B6U810_MAIZE); contains Interpro domain(s) IPR011990 Tetratricopeptide-like helical " | 5E-33 |
| 11 | Contig 11 | 672 | 1 | GH204504 | Solyc12g099030.1.1 evidence_code:10F0H1E1IEG genomic_reference:SL2.40ch12 gene_region:64677240-64679496 transcript_region:SL2.40ch12:64677240..64679496+ go_terms:GO:0005840 functional_description:"Ubiquitin (AHRD V1 ***- D3PHG3_9MAXI); contains Interpro domain(s) IPR019956 Ubiquitin subgroup " | 3E-69 |
| 12 | Contig 12 | 571 | 1 | GH204091 | ********* |  |
| 13 | Contig 13 | 831 | 8 | GH204457 | ********* |  |
| 14 | Contig 14 | 554 | 5 | GH204845 | Solyc01g104370.2.1 genomic_reference:SL2.40ch01 gene_region:84525541-84526490 transcript_region:SL2.40ch01:84525541..84526490- go_terms:GO:0005515 functional_description:"60S acidic ribosomal protein P1 (AHRD V1 ***- B6T3C1_MAIZE); contains Interpro domain(s) IPR001813 Ribosomal protein 60S " | 9E-18 |
| 15 | Contig 15 | 765 | 2 | GH204404 | Solyc08g068180.2.1 genomic_reference:SL2.40ch08 gene_region:54505411-54508853 transcript_region:SL2.40ch08:54505411..54508853+ go_terms:GO:0005840 functional_description:"Ribosomal protein L37 (AHRD V1 ***- B9RS60_RICCO); contains Interpro domain(s) IPR001569 Ribosomal protein L37e " | 2E-27 |
| 16 | Contig 16 | 748 | 3 | GH204361 | Solyc10g079420.1.1 evidence_code:10F0H1E1IEG genomic_reference:SL2.40ch10 gene_region:60282482-60283099 transcript_region:SL2.40ch10:60282482..60283099+ go_terms:GO:0005509 functional_description:"Calmodulin (AHRD V1 ***- B6TKX0_MAIZE); contains Interpro domain(s) IPR011992 EF-Hand type " | 2E-41 |
| 17 | Contig 17 | 287 | 2 | GH204740 | ********* |  |
| 18 | Contig 18 | 737 | 1 | GH204673 | Solyc01g091560.2.1 genomic_reference:SL2.40ch01 gene_region:76930674-76931868 transcript_region:SL2.40ch01:76930674..76931868+ go_terms:GO:0016291 functional_description:"Thioesterase family protein (AHRD V1 **** D7LS72_ARALY); contains Interpro domain(s) IPR006683 Thioesterase superfamily " | 6E-40 |
| 19 | Contig 19 | 181 | 2 | GH204783 | ********* |  |
| 20 | Contig 20 | 432 | 1 | GH204972 | Solyc12g008700.1.1 evidence_code:10F1H0E1IEG genomic_reference:SL2.40ch12 gene_region:2070165-2071739 transcript_region:SL2.40ch12:2070165..2071739- go_terms:GO:0005840 functional_description:"40S ribosomal protein S30-like (AHRD V1 ***- Q6K853_ORYSJ); contains Interpro domain(s) IPR006846 Ribosomal protein S30 " | 3E-22 |
| 21 | Contig 21 | 624 | 2 | GH204183 | Solyc06g009630.1.1 evidence_code:10F1H1E1IEG genomic_reference:SL2.40ch06 gene_region:3584164-3584547 transcript_region:SL2.40ch06:3584164..3584547+ go_terms:GO:0005515 functional_description:"CP12 (AHRD V1 ***- O24136_TOBAC); contains Interpro domain(s) IPR003823 Protein of unknown function CP12 " | 1E-27 |
| 22 | Contig 22 | 373 | 1 | GH204549 | ********* |  |
| 23 | Contig 23 | 434 | 1 | GH204978 | ********* |  |
| 24 | Contig 24 | 558 | 1 | GH204606 | Solyc03g112850.2.1 genomic_reference:SL2.40ch03 gene_region:57204158-57205860 transcript_region:SL2.40ch03:57204158..57205860- go_terms:GO:0003735,GO:0005515 functional_description:"60S ribosomal protein L44 (AHRD V1 ***- B9RH89_RICCO); contains Interpro domain(s) IPR000552 Ribosomal protein L44e " | 2E-47 |
| 25 | Contig 25 | 819 | 1 | GH204415 | Solyc10g005830.2.1 genomic_reference:SL2.40ch10 gene_region:646209-647697 transcript_region:SL2.40ch10:646209..647697+ functional_description:"Homoserine dehydrogenase-like (AHRD V1 ***- Q5QLC2_ORYSJ)" | 3E-72 |
| 26 | Contig 26 | 798 | 1 | GH205038 | Solyc03g119360.2.1 genomic_reference:SL2.40ch03 gene_region:62044635-62047323 transcript_region:SL2.40ch03:62044635..62047323- go_terms:GO:0032183,GO:0003735 functional_description:"40S ribosomal protein S7-like protein (AHRD V1 ***- Q3HRX6_SOLTU); contains Interpro domain(s) IPR000554 Ribosomal protein S7e " | 2E-74 |
| 27 | Contig 27 | 354 | 3 | GH204366 | Solyc06g069090.2.1 genomic_reference:SL2.40ch06 gene_region:39269885-39272377 transcript_region:SL2.40ch06:39269885..39272377- go_terms:GO:0005840 functional_description:"40S ribosomal protein S7-like protein (AHRD V1 ***- Q3HRX6_SOLTU); contains Interpro domain(s) IPR000554 Ribosomal protein S7e " | 4E-40 |
| 28 | Contig 28 | 361 | 1 | GH203632 | Solyc03g119360.2.1 genomic_reference:SL2.40ch03 gene_region:62044635-62047323 transcript_region:SL2.40ch03:62044635..62047323- go_terms:GO:0032183,GO:0003735 functional_description:"40S ribosomal protein S7-like protein (AHRD V1 ***- Q3HRX6_SOLTU); contains Interpro domain(s) IPR000554 Ribosomal protein S7e " | 9E-62 |
| 29 | Contig 29 | 216 | 1 | GH205032 | ********* |  |
| 30 | Contig 30 | 567 | 1 | GH204736 | ********* |  |
| 31 | Contig 31 | 462 | 1 | GH205160 | Solyc10g084020.1.1 evidence_code:10F0H0E0IEG genomic_reference:SL2.40ch10 gene_region:63012410-63012805 transcript_region:SL2.40ch10:63012410..63012805+ functional_description:"Auxin responsive SAUR protein (AHRD V1 *-*- Q2HTF3_MEDTR); contains Interpro domain(s) IPR003676 Auxin responsive SAUR protein " | 3E-33 |
| 32 | Contig 32 | 560 | 1 | GH205192 | ********* |  |
| 33 | Contig 33 | 808 | 1 | GH204397 | Solyc04g081570.2.1 genomic_reference:SL2.40ch04 gene_region:63110553-63116088 transcript_region:SL2.40ch04:63110553..63116088- go_terms:GO:0042623,GO:0050750 functional_description:"Chaperone protein htpG (AHRD V1 **-- HTPG_MYCA1); contains Interpro domain(s) IPR015566 Molecular chaperone, heat shock protein, endoplasmin " | 5E-52 |
| 34 | Contig 34 | 467 | 1 | GH204234 | Solyc08g076340.2.1 genomic_reference:SL2.40ch08 gene_region:57472737-57475415 transcript_region:SL2.40ch08:57472737..57475415- go_terms:GO:0003735,GO:0003723 functional_description:"40S ribosomal protein S30-like (AHRD V1 ***- Q6K853_ORYSJ); contains Interpro domain(s) IPR006846 Ribosomal protein S30 " | 8E-24 |
| 35 | Contig 35 | 290 | 2 | GH204729 | ********* |  |
| 36 | Contig 36 | 972 | 1 | GH203973 | ********* |  |
| 37 | Contig 37 | 603 | 2 | GH204663 | Solyc01g009860.2.1 genomic_reference:SL2.40ch01 gene_region:4382336-4384348 transcript_region:SL2.40ch01:4382336..4384348- go_terms:GO:0003700 functional_description:"NAC domain transcription factor (AHRD V1 **** Q5DM36_WHEAT); contains Interpro domain(s) IPR003441 No apical meristem (NAM) protein " | 1E-100 |
| 38 | Contig 38 | 841 | 2 | GH203680 | Solyc06g060230.2.1 genomic_reference:SL2.40ch06 gene_region:34577474-34578909 transcript_region:SL2.40ch06:34577474..34578909- go_terms:GO:0003700,GO:0016563 functional_description:"NAC domain protein IPR003441 (AHRD V1 ***- B9GU14_POPTR); contains Interpro domain(s) IPR003441 No apical meristem (NAM) protein " | 1E-115 |
| 39 | Contig 39 | 690 | 1 | GH204865 | Solyc01g009860.2.1 genomic_reference:SL2.40ch01 gene_region:4382336-4384348 transcript_region:SL2.40ch01:4382336..4384348- go_terms:GO:0003700 functional_description:"NAC domain transcription factor (AHRD V1 **** Q5DM36_WHEAT); contains Interpro domain(s) IPR003441 No apical meristem (NAM) protein " | 2E-57 |
| 40 | Contig 40 | 675 | 1 | GH204551 | ********* |  |
| 41 | Contig 41 | 329 | 1 | GH205366 | ********* |  |
| 42 | Contig 42 | 270 | 1 | GH204370 | ********* |  |
| 43 | Contig 43 | 581 | 1 | GH204664 | Solyc01g096290.2.1 genomic_reference:SL2.40ch01 gene_region:79140943-79142950 transcript_region:SL2.40ch01:79140943..79142950+ go_terms:GO:0005515,GO:0031386 functional_description:"Ubiquitin (AHRD V1 ***- C0SFI2_PARBP); contains Interpro domain(s) IPR019956 Ubiquitin subgroup " | 8E-68 |
| 44 | Contig 44 | 692 | 1 | GH203768 | Solyc11g005670.1.1 evidence_code:10F0H1E1IEG genomic_reference:SL2.40ch11 gene_region:496333-497937 transcript_region:SL2.40ch11:496333..497937+ functional_description:"Ubiquitin (AHRD V1 ***- Q2UZT4_MUSAC); contains Interpro domain(s) IPR019956 Ubiquitin subgroup " | 1E-111 |
| 45 | Contig 45 | 248 | 1 | GH203850 | Solyc08g008590.2.1 genomic_reference:SL2.40ch08 gene_region:2962192-2966912 transcript_region:SL2.40ch08:2962192..2966912+ go_terms:GO:0043008 functional_description:"Ubiquitin (AHRD V1 ***- C0SFI2_PARBP); contains Interpro domain(s) IPR019956 Ubiquitin subgroup " | 5E-19 |
| 46 | Contig 46 | 950 | 1 | GH205118 | ********* |  |
| 47 | Contig 47 | 517 | 1 | GH204793 | Solyc06g065710.2.1 genomic_reference:SL2.40ch06 gene_region:37530153-37541354 transcript_region:SL2.40ch06:37530153..37541354- go_terms:GO:0005488 functional_description:"Sister chromatid cohesion protein PDS5 homolog B-B (AHRD V1 *--- PD5BB_XENLA); contains Interpro domain(s) IPR016024 Armadillo-type fold " | 2E-33 |
| 48 | Contig 48 | 653 | 1 | GH203530 | Solyc05g023740.2.1 genomic_reference:SL2.40ch05 gene_region:29171601-29178176 transcript_region:SL2.40ch05:29171601..29178176+ go_terms:GO:0005515,GO:0003723 functional_description:"MEI2-like RNA binding protein (AHRD V1 **-* Q64M78_ORYSJ); contains Interpro domain(s) IPR007201 RNA recognition motif 2 " | 5E-99 |
| 49 | Contig 49 | 1116 | 15 | GH205338 | Solyc02g085950.2.1 genomic_reference:SL2.40ch02 gene_region:43293286-43294312 transcript_region:SL2.40ch02:43293286..43294312+ go_terms:GO:0005515,GO:0005507 functional_description:"Ribulose bisphosphate carboxylase small chain (AHRD V1 ***- A9YTZ7_SOLTU); contains Interpro domain(s) IPR000894 Ribulose bisphosphate carboxylase, small chain " | 1E-85 |
| 50 | Contig 50 | 449 | 4 | GH204760 | Solyc02g085950.2.1 genomic_reference:SL2.40ch02 gene_region:43293286-43294312 transcript_region:SL2.40ch02:43293286..43294312+ go_terms:GO:0005515,GO:0005507 functional_description:"Ribulose bisphosphate carboxylase small chain (AHRD V1 ***- A9YTZ7_SOLTU); contains Interpro domain(s) IPR000894 Ribulose bisphosphate carboxylase, small chain " | 2E-39 |
| 51 | Contig 51 | 597 | 2 | GH203637 | Solyc04g071900.2.1 genomic_reference:SL2.40ch04 gene_region:56513280-56516291 transcript_region:SL2.40ch04:56513280..56516291+ go_terms:GO:0004601 functional_description:"Peroxidase (AHRD V1 **** Q94IQ1_TOBAC); contains Interpro domain(s) IPR002016 Haem peroxidase, plant/fungal/bacterial " | 6E-90 |
| 52 | Contig 52 | 122 | 1 | GH204768 | ********* |  |
| 53 | Contig 53 | 690 | 1 | GH204469 | Solyc06g008260.2.1 genomic_reference:SL2.40ch06 gene_region:2134911-2138172 transcript_region:SL2.40ch06:2134911..2138172+ go_terms:GO:0003735,GO:0003723 functional_description:"60 ribosomal protein L14 (AHRD V1 ***- B6TMP6_MAIZE); contains Interpro domain(s) IPR002784 Ribosomal protein L14 " | 8E-42 |
| 54 | Contig 54 | 615 | 15 | GH203907 | Solyc06g024210.1.1 evidence_code:10F0H0E0IEG genomic_reference:SL2.40ch06 gene_region:14701266-14701628 transcript_region:SL2.40ch06:14701266..14701628+ functional_description:"TO54-2 (Fragment) (AHRD V1 *-*- Q3LVL5_TAROF)" | 8E-29 |
| 55 | Contig 55 | 1082 | 13 | GH204745 | Solyc07g052480.2.1 genomic_reference:SL2.40ch07 gene_region:58294426-58296992 transcript_region:SL2.40ch07:58294426..58296992- go_terms:GO:0046421,GO:0004451 functional_description:"Isocitrate lyase (AHRD V1 **** Q9FQD2_IPOBA); contains Interpro domain(s) IPR006254 Isocitrate lyase " | 0 |
| 56 | Contig 56 | 851 | 1 | GH203784 | Solyc04g078550.2.1 genomic_reference:SL2.40ch04 gene_region:60852591-60854416 transcript_region:SL2.40ch04:60852591..60854416+ go_terms:GO:0005516 functional_description:"WRKY transcription factor 2 (AHRD V1 ***- A7UGC9_SOLTU); contains Interpro domain(s) IPR003657 DNA-binding WRKY " | 1E-110 |
| 57 | Contig 57 | 212 | 14 | GH203733 | Solyc03g120320.1.1 evidence_code:10F0H1E1IEG genomic_reference:SL2.40ch03 gene_region:62801895-62802962 transcript_region:SL2.40ch03:62801895..62802962- functional_description:"Kelch-like protein (AHRD V1 *-*- C4Q722_SCHMA); contains Interpro domain(s) IPR015915 Kelch-type beta propeller " | 4E-37 |
| 58 | Contig 58 | 879 | 5 | GH204942 | Solyc06g009970.2.1 genomic_reference:SL2.40ch06 gene_region:4379727-4382219 transcript_region:SL2.40ch06:4379727..4382219+ go_terms:GO:0051015 functional_description:"Elongation factor 1-alpha (AHRD V1 ***- Q8H9C0_SOLTU); contains Interpro domain(s) IPR004539 Translation elongation factor EF1A, eukaryotic and archaeal IPR000795 Protein synthesis factor, GTP-binding " | 1E-125 |
| 59 | Contig 59 | 746 | 2 | GH203444 | Solyc03g026280.2.1 genomic_reference:SL2.40ch03 gene_region:8094816-8095712 transcript_region:SL2.40ch03:8094816..8095712+ go_terms:GO:0016563 functional_description:"CRT binding factor 2 (AHRD V1 *-*- B3TPN7_SOLHA); contains Interpro domain(s) IPR001471 Pathogenesis-related transcriptional factor and ERF, DNA-binding " | 2E-32 |
| 60 | Contig 60 | 953 | 3 | GH204106 | Solyc04g082680.2.1 genomic_reference:SL2.40ch04 gene_region:63870914-63876134 transcript_region:SL2.40ch04:63870914..63876134+ functional_description:"Chloroplast channel forming outer membrane protein (AHRD V1 ***- Q9SM57_PEA)" | 2E-51 |
| 61 | Contig 61 | 820 | 1 | GH204857 | Solyc03g083620.1.1 evidence_code:10F1H0E1IEG genomic_reference:SL2.40ch03 gene_region:47126438-47126803 transcript_region:SL2.40ch03:47126438..47126803- functional_description:"Unknown Protein (AHRD V1)" | 4E-22 |
| 62 | Contig 62 | 112 | 1 | GH204778 | ********* |  |
| 63 | Contig 63 | 582 | 1 | GH204665 | Solyc01g056780.2.1 genomic_reference:SL2.40ch01 gene_region:49444478-49445701 transcript_region:SL2.40ch01:49444478..49445701+ go_terms:GO:0005840 functional_description:"50S ribosomal protein L34 (AHRD V1 *-*- D7KDQ0_ARALY); contains Interpro domain(s) IPR000271 Ribosomal protein L34 " | 1E-13 |
| 64 | Contig 64 | 713 | 5 | GH205009 | Solyc01g094690.2.1 genomic_reference:SL2.40ch01 gene_region:77897514-77900268 transcript_region:SL2.40ch01:77897514..77900268+ go_terms:GO:0016020,GO:0016021 functional_description:"Aquaporin (AHRD V1 ***- Q40266_MESCR); contains Interpro domain(s) IPR012269 Aquaporin " | 1E-116 |
| 65 | Contig 65 | 892 | 2 | GH205224 | Solyc08g081190.2.1 genomic_reference:SL2.40ch08 gene_region:61427665-61431015 transcript_region:SL2.40ch08:61427665..61431015+ go_terms:GO:0015250 functional_description:"Aquaporin 1 (AHRD V1 ***- O24662_TOBAC); contains Interpro domain(s) IPR012269 Aquaporin " | 2E-97 |
| 66 | Contig 66 | 521 | 2 | GH205350 | Solyc01g111660.2.1 genomic_reference:SL2.40ch01 gene_region:89627217-89628682 transcript_region:SL2.40ch01:89627217..89628682+ go_terms:GO:0015250 functional_description:"Aquaporin-like protein (AHRD V1 ***- Q8W1A8_PETHY); contains Interpro domain(s) IPR012269 Aquaporin " | 9E-57 |
| 67 | Contig 67 | 449 | 1 | GH204919 | Solyc10g055630.1.1 evidence_code:10F1H1E1IEG genomic_reference:SL2.40ch10 gene_region:52582972-52584379 transcript_region:SL2.40ch10:52582972..52584379+ go_terms:GO:0015250 functional_description:"Aquaporin (AHRD V1 ***- Q8W506_TOBAC); contains Interpro domain(s) IPR012269 Aquaporin " | 5E-60 |
| 68 | Contig 68 | 687 | 6 | GH204406 | Solyc11g006460.1.1 evidence_code:10F1H1E1IEG genomic_reference:SL2.40ch11 gene_region:1149792-1152899 transcript_region:SL2.40ch11:1149792..1152899- go_terms:GO:0006457,GO:0031072 functional_description:"DNAJ chaperone (AHRD V1 ***- B0W7V8_CULQU); contains Interpro domain(s) IPR003095 Heat shock protein DnaJ " | 2E-71 |
| 69 | Contig 69 | 607 | 1 | GH205242 | Solyc01g107870.2.1 genomic_reference:SL2.40ch01 gene_region:87021438-87028206 transcript_region:SL2.40ch01:87021438..87028206+ go_terms:GO:0008143 functional_description:"Poly(A) RNA binding protein (AHRD V1 **** B2CJ74_9HYPO); contains Interpro domain(s) IPR006515 Polyadenylate binding protein, human types 1, 2, 3, 4 " | 1E-111 |
| 70 | Contig 70 | 238 | 1 | GH203618 | ********* |  |
| 71 | Contig 71 | 446 | 1 | GH203791 | Solyc07g008560.2.1 genomic_reference:SL2.40ch07 gene_region:3500707-3507834 transcript_region:SL2.40ch07:3500707..3507834+ go_terms:GO:0004722,GO:0005515 functional_description:"Purple acid phosphatase (AHRD V1 **-* B0WRM8_CULQU); contains Interpro domain(s) IPR008963 Purple acid phosphatase-like, N-terminal " | 3E-85 |
| 72 | Contig 72 | 756 | 1 | GH204193 | Solyc06g072430.1.1 evidence_code:10F0H0E1IEG genomic_reference:SL2.40ch06 gene_region:41073324-41074445 transcript_region:SL2.40ch06:41073324..41074445+ go_terms:GO:0006915 functional_description:"Bcl-2-associated athanogene-like protein (AHRD V1 *-*- D1MIX4_VITVI); contains Interpro domain(s) IPR003103 Apoptosis regulator Bcl-2 protein, BAG " | 1E-121 |
| 73 | Contig 73 | 666 | 9 | GH204712 | Solyc10g075110.1.1 evidence_code:10F1H0E1IEG genomic_reference:SL2.40ch10 gene_region:58157020-58157628 transcript_region:SL2.40ch10:58157020..58157628- go_terms:GO:0006869 functional_description:"Non-specific lipid-transfer protein (AHRD V1 ***- A7UGG9_SOLTU); contains Interpro domain(s) IPR000528 Plant lipid transfer protein/Par allergen " | 2E-27 |
| 74 | Contig 74 | 468 | 8 | GH203937 | Solyc01g104370.2.1 genomic_reference:SL2.40ch01 gene_region:84525541-84526490 transcript_region:SL2.40ch01:84525541..84526490- go_terms:GO:0005515 functional_description:"60S acidic ribosomal protein P1 (AHRD V1 ***- B6T3C1_MAIZE); contains Interpro domain(s) IPR001813 Ribosomal protein 60S " | 4E-25 |
| 75 | Contig 75 | 546 | 1 | GH204528 | Solyc11g067100.1.1 evidence_code:10F1H0E1IEG genomic_reference:SL2.40ch11 gene_region:49928225-49929780 transcript_region:SL2.40ch11:49928225..49929780- go_terms:GO:0005840 functional_description:"60s acidic ribosomal protein-like protein (AHRD V1 ***- Q3HVP0_SOLTU); contains Interpro domain(s) IPR001813 Ribosomal protein 60S " | 1E-20 |
| 76 | Contig 76 | 692 | 4 | GH203606 | Solyc01g099630.2.1 genomic_reference:SL2.40ch01 gene_region:81570892-81573619 transcript_region:SL2.40ch01:81570892..81573619- go_terms:GO:0016798 functional_description:"Xyloglucan endotransglucosylase/hydrolase 5 (AHRD V1 **** C0IRG4_ACTDE); contains Interpro domain(s) IPR016455 Xyloglucan endotransglucosylase/hydrolase IPR008263 Glycoside hydrolase, family 16, active site " | 1E-138 |
| 77 | Contig 77 | 706 | 2 | GH205301 | Solyc07g055990.2.1 genomic_reference:SL2.40ch07 gene_region:61215070-61217037 transcript_region:SL2.40ch07:61215070..61217037+ go_terms:GO:0016798 functional_description:"Xyloglucan endotransglucosylase/hydrolase 7 (AHRD V1 **** C0IRG6_ACTDE); contains Interpro domain(s) IPR016455 Xyloglucan endotransglucosylase/hydrolase " | 1E-115 |
| 78 | Contig 78 | 785 | 1 | GH205029 | Solyc03g093120.2.1 genomic_reference:SL2.40ch03 gene_region:47736430-47737672 transcript_region:SL2.40ch03:47736430..47737672- go_terms:GO:0016798,GO:0016762 functional_description:"Xyloglucan endotransglucosylase/hydrolase 9 (AHRD V1 **** C0IRG8_9ERIC); contains Interpro domain(s) IPR016455 Xyloglucan endotransglucosylase/hydrolase " | 1E-128 |
| 79 | Contig 79 | 567 | 1 | GH203550 | Solyc07g056000.2.1 genomic_reference:SL2.40ch07 gene_region:61225049-61226324 transcript_region:SL2.40ch07:61225049..61226324+ go_terms:GO:0016798 functional_description:"Xyloglucan endotransglucosylase/hydrolase 7 (AHRD V1 **** C0IRG6_ACTDE); contains Interpro domain(s) IPR016455 Xyloglucan endotransglucosylase/hydrolase IPR008263 Glycoside hydrolase, family 16, active site " | 1E-103 |
| 80 | Contig 80 | 387 | 1 | GH203511 | Solyc07g052980.2.1 genomic_reference:SL2.40ch07 gene_region:58724756-58726704 transcript_region:SL2.40ch07:58724756..58726704+ go_terms:GO:0016798 functional_description:"Xyloglucan endotransglucosylase/hydrolase 5 (AHRD V1 **** C0IRH8_MALDO); contains Interpro domain(s) IPR016455 Xyloglucan endotransglucosylase/hydrolase " | 4E-73 |
| 81 | Contig 81 | 438 | 8 | GH204446 | Solyc09g097770.2.1 genomic_reference:SL2.40ch09 gene_region:67062889-67064180 transcript_region:SL2.40ch09:67062889..67064180+ functional_description:"Cell wall protein (AHRD V1 ***- Q40142_SOLLC); contains Interpro domain(s) IPR010800 Glycine rich " | 2E-11 |
| 82 | Contig 82 | 789 | 7 | GH204181 | Solyc08g081230.1.1 evidence_code:10F0H1E1IEG genomic_reference:SL2.40ch08 gene_region:61456635-61458026 transcript_region:SL2.40ch08:61456635..61458026- go_terms:GO:0005515,GO:0016020 functional_description:"Potassium channel tetramerization domain-containing protein (AHRD V1 ***- D7MIW8_ARALY); contains Interpro domain(s) IPR003131 Potassium channel, voltage dependent, Kv, tetramerisation " | 1E-140 |
| 83 | Contig 83 | 662 | 5 | GH203522 | Solyc02g067180.2.1 genomic_reference:SL2.40ch02 gene_region:31941535-31947091 transcript_region:SL2.40ch02:31941535..31947091- go_terms:GO:0006520,GO:0030170 functional_description:"Cystathionine gamma synthase (AHRD V1 ***- Q6R8F6_SOLLC); contains Interpro domain(s) IPR000277 Cys/Met metabolism, pyridoxal phosphate-dependent enzyme " | 5E-72 |
| 84 | Contig 84 | 699 | 2 | GH204646 | Solyc01g090770.2.1 genomic_reference:SL2.40ch01 gene_region:76182282-76188354 transcript_region:SL2.40ch01:76182282..76188354- functional_description:"Unknown Protein (AHRD V1); contains Interpro domain(s) IPR016803 Uncharacterised conserved protein UCP022280 " | 2E-62 |
| 85 | Contig 85 | 805 | 6 | GH203742 | Solyc08g006040.2.1 genomic_reference:SL2.40ch08 gene_region:798845-801415 transcript_region:SL2.40ch08:798845..801415- go_terms:GO:0003735,GO:0005515 functional_description:"40S ribosomal protein S6 (AHRD V1 ***- B6T1H0_MAIZE); contains Interpro domain(s) IPR014401 Ribosomal protein S6, eukaryotic " | 8E-66 |
| 86 | Contig 86 | 654 | 1 | GH203772 | Solyc02g086460.2.1 genomic_reference:SL2.40ch02 gene_region:43702622-43720134 transcript_region:SL2.40ch02:43702622..43720134+ functional_description:"Unknown Protein (AHRD V1)" | 1E-122 |
| 87 | Contig 87 | 429 | 3 | GH203902 | Solyc02g086730.1.1 evidence_code:10F1H1E1IEG genomic_reference:SL2.40ch02 gene_region:43937531-43938103 transcript_region:SL2.40ch02:43937531..43938103+ go_terms:GO:0005515,GO:0003682 functional_description:"50S ribosomal protein L12-C (AHRD V1 ***- Q8LBJ7_ARATH); contains Interpro domain(s) IPR015608 Ribosomal protein L12, chloroplast " | 1E-33 |
| 88 | Contig 88 | 470 | 2 | GH204378 | Solyc02g086820.2.1 genomic_reference:SL2.40ch02 gene_region:44012475-44015926 transcript_region:SL2.40ch02:44012475..44015926+ go_terms:GO:0005515 functional_description:"Carbonic anhydrase (AHRD V1 ***- Q5NE20_SOLLC); contains Interpro domain(s) IPR015892 Carbonic anhydrase, prokaryotic-like, conserved site " | 4E-64 |
| 89 | Contig 89 | 665 | 1 | GH204272 | Solyc12g056830.1.1 evidence_code:10F0H1E1IEG genomic_reference:SL2.40ch12 gene_region:48264644-48265396 transcript_region:SL2.40ch12:48264644..48265396+ go_terms:GO:0045261 functional_description:"ATP synthase delta subunit (AHRD V1 ***- Q7XYM8_BIGNA); contains Interpro domain(s) IPR000711 ATPase, F1 complex, OSCP/delta subunit " | 1E-100 |
| 90 | Contig 90 | 813 | 1 | GH204547 | Solyc09g020190.2.1 genomic_reference:SL2.40ch09 gene_region:19185577-19189409 transcript_region:SL2.40ch09:19185577..19189409+ go_terms:GO:0016788 functional_description:"Phosphoesterase family protein (AHRD V1 ***- D7KH02_ARALY); contains Interpro domain(s) IPR007312 Phosphoesterase " | 4E-94 |
| 91 | Contig 91 | 536 | 2 | GH205062 | Solyc01g111500.2.1 genomic_reference:SL2.40ch01 gene_region:89480965-89482050 transcript_region:SL2.40ch01:89480965..89482050- go_terms:GO:0003700 functional_description:"MYB transcription factor (AHRD V1 **** B9H191_POPTR); contains Interpro domain(s) IPR015495 Myb transcription factor " | 1E-64 |
| 92 | Contig 92 | 234 | 3 | GH204873 | Solyc04g080590.2.1 genomic_reference:SL2.40ch04 gene_region:62295025-62299348 transcript_region:SL2.40ch04:62295025..62299348- go_terms:GO:0019773,GO:0005839 functional_description:"Proteasome subunit alpha type (AHRD V1 ***- Q8H1Y2_NICBE); contains Interpro domain(s) IPR001353 Proteasome, subunit alpha/beta " | 6E-39 |
| 93 | Contig 93 | 694 | 1 | GH204244 | Solyc08g006720.2.1 genomic_reference:SL2.40ch08 gene_region:1274923-1279455 transcript_region:SL2.40ch08:1274923..1279455- go_terms:GO:0055114 functional_description:"Glutathione peroxidase (AHRD V1 ***- D6BR59_9ROSI); contains Interpro domain(s) IPR000889 Glutathione peroxidase " | 1E-113 |
| 94 | Contig 94 | 683 | 1 | GH205212 | Solyc06g075690.2.1 genomic_reference:SL2.40ch06 gene_region:43427533-43430452 transcript_region:SL2.40ch06:43427533..43430452+ functional_description:"Auxin-regulated protein (AHRD V1 ***- Q945F5_SOLLC); contains Interpro domain(s) IPR010369 Protein of unknown function DUF966 " | 1E-102 |
| 95 | Contig 95 | 714 | 6 | GH203918 | Solyc08g076220.2.1 genomic_reference:SL2.40ch08 gene_region:57405726-57410850 transcript_region:SL2.40ch08:57405726..57410850+ go_terms:GO:0005515 functional_description:"Phosphoribulokinase/uridine kinase (AHRD V1 ***- B7KI62_CYAP7); contains Interpro domain(s) IPR006083 Phosphoribulokinase/uridine kinase " | 1E-119 |
| 96 | Contig 96 | 839 | 6 | GH204846 | Solyc02g070360.2.1 genomic_reference:SL2.40ch02 gene_region:34691346-34693734 transcript_region:SL2.40ch02:34691346..34693734+ go_terms:GO:0032183,GO:0003735 functional_description:"40S ribosomal protein S4-like protein (AHRD V1 ***- Q2XPX5_SOLTU); contains Interpro domain(s) IPR000876 Ribosomal protein S4e " | 1E-103 |
| 97 | Contig 97 | 530 | 4 | GH205139 | Solyc05g055230.1.1 evidence_code:10F1H1E1IEG genomic_reference:SL2.40ch05 gene_region:64111952-64112386 transcript_region:SL2.40ch05:64111952..64112386- go_terms:GO:0003735,GO:0003723 functional_description:"40S ribosomal protein S17-like protein (AHRD V1 ***- Q3HRX4_SOLTU); contains Interpro domain(s) IPR018273 Ribosomal protein S17e, conserved site " | 2E-58 |
| 98 | Contig 98 | 340 | 2 | GH203591 | Solyc05g055230.1.1 evidence_code:10F1H1E1IEG genomic_reference:SL2.40ch05 gene_region:64111952-64112386 transcript_region:SL2.40ch05:64111952..64112386- go_terms:GO:0003735,GO:0003723 functional_description:"40S ribosomal protein S17-like protein (AHRD V1 ***- Q3HRX4_SOLTU); contains Interpro domain(s) IPR018273 Ribosomal protein S17e, conserved site " | 5E-54 |
| 99 | Contig 99 | 476 | 3 | GH204301 | ********* |  |
| 100 | Contig 100 | 783 | 2 | GH204422 | ********* |  |
| 101 | Contig 101 | 352 | 1 | GH204489 | ********* |  |
| 102 | Contig 102 | 689 | 4 | GH204747 | Solyc09g090610.2.1 genomic_reference:SL2.40ch09 gene_region:65421245-65423825 transcript_region:SL2.40ch09:65421245..65423825- go_terms:GO:0005840 functional_description:"50S ribosomal protein L14 (AHRD V1 ***- RL14_DEHE1); contains Interpro domain(s) IPR000218 Ribosomal protein L14b/L23e " | 8E-77 |
| 103 | Contig 103 | 593 | 2 | GH204726 | Solyc06g083780.2.1 genomic_reference:SL2.40ch06 gene_region:45395413-45397278 transcript_region:SL2.40ch06:45395413..45397278+ go_terms:GO:0005840 functional_description:"50S ribosomal protein L14 (AHRD V1 ***- RL14_DEHE1); contains Interpro domain(s) IPR000218 Ribosomal protein L14b/L23e " | 5E-54 |
| 104 | Contig 104 | 626 | 4 | GH204325 | Solyc10g086020.1.1 evidence_code:10F1H1E1IEG genomic_reference:SL2.40ch10 gene_region:64331344-64333142 transcript_region:SL2.40ch10:64331344..64333142- go_terms:GO:0005840,GO:0015935 functional_description:"30S ribosomal protein S12 (AHRD V1 ***- RS12_MYCPU); contains Interpro domain(s) IPR005680 Ribosomal protein S23, eukaryotic/archaeal " | 5E-78 |
| 105 | Contig 105 | 684 | 1 | GH205100 | Solyc10g084310.1.1 evidence_code:10F1H1E1IEG genomic_reference:SL2.40ch10 gene_region:63236088-63237567 transcript_region:SL2.40ch10:63236088..63237567+ go_terms:GO:0005840,GO:0015935 functional_description:"30S ribosomal protein S12 (AHRD V1 ***- RS12_MYCPU); contains Interpro domain(s) IPR005680 Ribosomal protein S23, eukaryotic/archaeal " | 7E-78 |
| 106 | Contig 106 | 854 | 1 | GH204795 | Solyc01g008550.2.1 genomic_reference:SL2.40ch01 gene_region:2646612-2650332 transcript_region:SL2.40ch01:2646612..2650332- go_terms:GO:0044237,GO:0008152 functional_description:"Cinnamoyl CoA reductase-like protein (AHRD V1 ***- B9HNY0_POPTR); contains Interpro domain(s) IPR016040 NAD(P)-binding domain " | 1E-149 |
| 107 | Contig 107 | 739 | 2 | GH203834 | Solyc03g120430.2.1 genomic_reference:SL2.40ch03 gene_region:62875286-62878923 transcript_region:SL2.40ch03:62875286..62878923- go_terms:GO:0008887 functional_description:"Glycerate kinase (AHRD V1 **** C1DYY5_9CHLO)" | 1E-113 |
| 108 | Contig 108 | 1110 | 3 | GH204253 | Solyc06g073090.2.1 genomic_reference:SL2.40ch06 gene_region:41417554-41421363 transcript_region:SL2.40ch06:41417554..41421363+ go_terms:GO:0003735,GO:0003824 functional_description:"Ribosomal subunit interface protein (AHRD V1 **-- Q2JIS4_SYNJB); contains Interpro domain(s) IPR003489 Ribosomal protein S30Ae/sigma 54 modulation protein " | 1E-144 |
| 109 | Contig 109 | 798 | 1 | GH204417 | Solyc03g112900.2.1 genomic_reference:SL2.40ch03 gene_region:57238077-57241465 transcript_region:SL2.40ch03:57238077..57241465+ go_terms:GO:0008198 functional_description:"Iron-sulfur cluster assembly scaffold protein IscU (AHRD V1 **** D0I7Q6_VIBHO); contains Interpro domain(s) IPR002871 NIF system FeS cluster assembly, NifU, N-terminal " | 3E-56 |
| 110 | Contig 110 | 716 | 2 | GH204838 | Solyc05g054810.2.1 genomic_reference:SL2.40ch05 gene_region:63775298-63777437 transcript_region:SL2.40ch05:63775298..63777437+ go_terms:GO:0003735 functional_description:"60S ribosomal protein L35 (AHRD V1 ***- D7M7V2_ARALY); contains Interpro domain(s) IPR001854 Ribosomal protein L29 " | 7E-40 |
| 111 | Contig 111 | 571 | 1 | GH204907 | Solyc05g052800.2.1 genomic_reference:SL2.40ch05 gene_region:62130017-62132095 transcript_region:SL2.40ch05:62130017..62132095- go_terms:GO:0003735 functional_description:"60S ribosomal protein L35a (AHRD V1 ***- B6VC50_VERFO); contains Interpro domain(s) IPR001780 Ribosomal protein L35Ae " | 2E-49 |
| 112 | Contig 112 | 461 | 1 | GH204175 | ********* |  |
| 113 | Contig 113 | 141 | 1 | GH204418 | ********* |  |
| 114 | Contig 114 | 531 | 1 | GH204108 | Solyc05g052810.2.1 genomic_reference:SL2.40ch05 gene_region:62132991-62135193 transcript_region:SL2.40ch05:62132991..62135193- go_terms:GO:0005840 functional_description:"60S ribosomal protein L33-B (AHRD V1 *-*- B6T1L8_MAIZE); contains Interpro domain(s) IPR001780 Ribosomal protein L35Ae " | 6E-36 |
| 115 | Contig 115 | 330 | 2 | GH205152 | Solyc01g105050.2.1 genomic_reference:SL2.40ch01 gene_region:85093521-85094689 transcript_region:SL2.40ch01:85093521..85094689+ go_terms:GO:0016168 functional_description:"Chlorophyll a-b binding protein, chloroplastic (AHRD V1 **** CB12_PETHY); contains Interpro domain(s) IPR001344 Chlorophyll A-B binding protein " | 6E-63 |
| 116 | Contig 116 | 785 | 1 | GH204833 | Solyc09g014520.2.1 genomic_reference:SL2.40ch09 gene_region:6145784-6147813 transcript_region:SL2.40ch09:6145784..6147813- go_terms:GO:0005515 functional_description:"Chlorophyll a-b binding protein 6A, chloroplastic (AHRD V1 **** CB11_SOLLC); contains Interpro domain(s) IPR001344 Chlorophyll A-B binding protein " | 2E-76 |
| 117 | Contig 117 | 723 | 1 | GH204826 | Solyc03g005780.1.1 evidence_code:10F1H1E1IEG genomic_reference:SL2.40ch03 gene_region:543035-543838 transcript_region:SL2.40ch03:543035..543838+ go_terms:GO:0016168 functional_description:"Chlorophyll a-b binding protein 3C-like (AHRD V1 **** Q2XTE0_SOLTU); contains Interpro domain(s) IPR001344 Chlorophyll A-B binding protein " | 1E-121 |
| 118 | Contig 118 | 661 | 1 | GH204401 | Solyc12g011450.1.1 evidence_code:10F1H1E1IEG genomic_reference:SL2.40ch12 gene_region:4262292-4263398 transcript_region:SL2.40ch12:4262292..4263398+ go_terms:GO:0016020 functional_description:"Chlorophyll a-b binding protein 13, chloroplastic (AHRD V1 ***- CB23_SOLLC); contains Interpro domain(s) IPR001344 Chlorophyll A-B binding protein " | 1E-121 |
| 119 | Contig 119 | 488 | 1 | GH204086 | Solyc10g006230.2.1 genomic_reference:SL2.40ch10 gene_region:920027-923670 transcript_region:SL2.40ch10:920027..923670+ go_terms:GO:0016020 functional_description:"Chlorophyll a-b binding protein 7, chloroplastic (AHRD V1 ***- CB12_SOLLC); contains Interpro domain(s) IPR001344 Chlorophyll A-B binding protein " | 2E-64 |
| 120 | Contig 120 | 515 | 2 | GH204510 | Solyc01g094560.2.1 genomic_reference:SL2.40ch01 gene_region:77748472-77751106 transcript_region:SL2.40ch01:77748472..77751106- go_terms:GO:0003735 functional_description:"60S ribosomal protein L36 (AHRD V1 ***- B9I1L9_POPTR); contains Interpro domain(s) IPR000509 Ribosomal protein L36e " | 7E-52 |
| 121 | Contig 121 | 273 | 3 | GH203488 | Solyc03g025520.2.1 genomic_reference:SL2.40ch03 gene_region:7288938-7290880 transcript_region:SL2.40ch03:7288938..7290880+ go_terms:GO:0003735,GO:0003723 functional_description:"60S ribosomal protein L36 (AHRD V1 ***- B9I1L9_POPTR); contains Interpro domain(s) IPR000509 Ribosomal protein L36e " | 3E-45 |
| 122 | Contig 122 | 244 | 1 | GH203906 | ********* |  |
| 123 | Contig 123 | 739 | 5 | GH203564 | Solyc12g095960.1.1 evidence_code:10F0H1E1IEG genomic_reference:SL2.40ch12 gene_region:63401514-63405993 transcript_region:SL2.40ch12:63401514..63405993- go_terms:GO:0051010 functional_description:"Insulin-like growth factor 2 mRNA-binding protein 2 (AHRD V1 *-** IF2B2_PONAB); contains Interpro domain(s) IPR018111 K Homology, type 1, subgroup " | 5E-56 |
| 124 | Contig 124 | 806 | 4 | GH204596 | Solyc05g054070.2.1 genomic_reference:SL2.40ch05 gene_region:63196547-63198365 transcript_region:SL2.40ch05:63196547..63198365- go_terms:GO:0003735,GO:0003723 functional_description:"60S ribosomal protein L6 (AHRD V1 ***- B9RHH3_RICCO); contains Interpro domain(s) IPR000915 Ribosomal protein L6E " | 2E-89 |
| 125 | Contig 125 | 419 | 1 | GH204713 | Solyc04g014720.2.1 genomic_reference:SL2.40ch04 gene_region:4982850-4985110 transcript_region:SL2.40ch04:4982850..4985110+ go_terms:GO:0003735,GO:0003723 functional_description:"60S ribosomal protein L6 (AHRD V1 ***- B9RHH3_RICCO); contains Interpro domain(s) IPR000915 Ribosomal protein L6E " | 3E-19 |
| 126 | Contig 126 | 570 | 5 | GH204807 | Solyc11g005330.1.1 evidence_code:10F1H1E1IEG genomic_reference:SL2.40ch11 gene_region:263971-265417 transcript_region:SL2.40ch11:263971..265417- go_terms:GO:0005515,GO:0005524 functional_description:"Actin (AHRD V1 ***- Q7XZK0_GOSHI); contains Interpro domain(s) IPR004000 Actin/actin-like " | 1E-104 |
| 127 | Contig 127 | 783 | 5 | GH204764 | Solyc07g065340.1.1 evidence_code:10F0H1E1IEG genomic_reference:SL2.40ch07 gene_region:64367108-64368103 transcript_region:SL2.40ch07:64367108..64368103- go_terms:GO:0009001 functional_description:"Serine acetyltransferase (AHRD V1 **** Q39533_CITLA); contains Interpro domain(s) IPR005881 Serine O-acetyltransferase " | 5E-99 |
| 128 | Contig 128 | 327 | 5 | GH203611 | Solyc11g066630.1.1 evidence_code:10F1H1E1IEG genomic_reference:SL2.40ch11 gene_region:49482826-49486050 transcript_region:SL2.40ch11:49482826..49486050- go_terms:GO:0005515,GO:0003723 functional_description:"MKI67 FHA domain-interacting nucleolar phosphoprotein-like (AHRD V1 **-- B4FL92_MAIZE); contains Interpro domain(s) IPR012677 Nucleotide-binding, alpha-beta plait " | 8E-23 |
| 129 | Contig 129 | 571 | 3 | GH203604 | Solyc07g016150.2.1 genomic_reference:SL2.40ch07 gene_region:6357299-6359427 transcript_region:SL2.40ch07:6357299..6359427+ go_terms:GO:0003785 functional_description:"Elongation factor 1-beta (AHRD V1 ***- D7KES3_ARALY); contains Interpro domain(s) IPR014038 Translation elongation factor EF1B, beta and delta chains, guanine nucleotide exchange " | 3E-63 |
| 130 | Contig 130 | 703 | 1 | GH203823 | Solyc11g012770.1.1 evidence_code:10F0H1E1IEG genomic_reference:SL2.40ch11 gene_region:5526597-5549066 transcript_region:SL2.40ch11:5526597..5549066+ go_terms:GO:0005488 functional_description:"Sister chromatid cohesion protein PDS5 homolog B (AHRD V1 **-- PDS5B_HUMAN); contains Interpro domain(s) IPR016024 Armadillo-type fold " | 1E-92 |
| 131 | Contig 131 | 383 | 1 | GH204484 | Solyc01g111500.2.1 genomic_reference:SL2.40ch01 gene_region:89480965-89482050 transcript_region:SL2.40ch01:89480965..89482050- go_terms:GO:0003700 functional_description:"MYB transcription factor (AHRD V1 **** B9H191_POPTR); contains Interpro domain(s) IPR015495 Myb transcription factor " | 5E-75 |
| 132 | Contig 132 | 716 | 3 | GH204318 | Solyc01g109250.2.1 genomic_reference:SL2.40ch01 gene_region:88009744-88010619 transcript_region:SL2.40ch01:88009744..88010619+ go_terms:GO:0005515 functional_description:"TMV response-related protein (AHRD V1 **-- B6SLK0_MAIZE)" | 3E-82 |
| 133 | Contig 133 | 431 | 2 | GH203479 | Solyc04g077980.1.1 evidence_code:10F1H1E1IEG genomic_reference:SL2.40ch04 gene_region:60443326-60444108 transcript_region:SL2.40ch04:60443326..60444108+ go_terms:GO:0003700,GO:0016564 functional_description:"Zinc-finger protein (AHRD V1 ***- Q40899_PETHY); contains Interpro domain(s) IPR007087 Zinc finger, C2H2-type " | 2E-39 |
| 134 | Contig 134 | 690 | 3 | GH204129 | Solyc08g062910.2.1 genomic_reference:SL2.40ch08 gene_region:49531008-49535044 transcript_region:SL2.40ch08:49531008..49535044- go_terms:GO:0003746 functional_description:"Elongation factor EF-2 (AHRD V1 **** Q9SGT4_ARATH); contains Interpro domain(s) IPR000795 Protein synthesis factor, GTP-binding " | 1E-105 |
| 135 | Contig 135 | 402 | 1 | GH205185 | Solyc07g062700.2.1 genomic_reference:SL2.40ch07 gene_region:62603490-62608714 transcript_region:SL2.40ch07:62603490..62608714- go_terms:GO:0016021,GO:0005509 functional_description:"Sodium/calcium exchanger family protein (AHRD V1 ***- D7KKF3_ARALY); contains Interpro domain(s) IPR004837 Sodium/calcium exchanger membrane region " | 1E-55 |
| 136 | Contig 136 | 318 | 1 | GH204213 | Solyc06g005060.2.1 genomic_reference:SL2.40ch06 gene_region:36352-38132 transcript_region:SL2.40ch06:36352..38132+ go_terms:GO:0051015 functional_description:"Elongation factor 1-alpha (AHRD V1 ***- Q8H9C0_SOLTU); contains Interpro domain(s) IPR004539 Translation elongation factor EF1A, eukaryotic and archaeal IPR000795 Protein synthesis factor, GTP-binding " | 5E-43 |
| 137 | Contig 137 | 1037 | 5 | GH203671 | Solyc10g008980.2.1 genomic_reference:SL2.40ch10 gene_region:3003367-3007677 transcript_region:SL2.40ch10:3003367..3007677+ go_terms:GO:0008524 functional_description:"Triose phosphate/phosphate translocator (AHRD V1 **** Q9MSB6_MESCR); contains Interpro domain(s) IPR004696 Tpt phosphate/phosphoenolpyruvate translocator " | 7E-63 |
| 138 | Contig 138 | 454 | 4 | GH203863 | Solyc04g008460.2.1 genomic_reference:SL2.40ch04 gene_region:2077830-2080111 transcript_region:SL2.40ch04:2077830..2080111+ go_terms:GO:0005840 functional_description:"Ribosomal protein L15 (AHRD V1 ***- B9GPA1_POPTR); contains Interpro domain(s) IPR000439 Ribosomal protein L15e " | 6E-72 |
| 139 | Contig 139 | 333 | 1 | GH204057 | Solyc01g080470.2.1 genomic_reference:SL2.40ch01 gene_region:72218392-72222088 transcript_region:SL2.40ch01:72218392..72222088+ go_terms:GO:0006396 functional_description:"tRNA (Guanosine-2&apos-O)-methyltransferase (AHRD V1 ***- D2TIV2_CITRI); contains Interpro domain(s) IPR001537 tRNA/rRNA methyltransferase, SpoU " | 1E-46 |
| 140 | Contig 140 | 797 | 2 | GH204487 | Solyc10g085300.1.1 evidence_code:10F1H1E1IEG genomic_reference:SL2.40ch10 gene_region:63850620-63853872 transcript_region:SL2.40ch10:63850620..63853872- functional_description:"Os03g0210500 protein (Fragment) (AHRD V1 *-*- Q0DU33_ORYSJ); contains Interpro domain(s) IPR007493 Protein of unknown function DUF538 " | 9E-94 |
| 141 | Contig 141 | 517 | 2 | GH204943 | Solyc08g075690.2.1 genomic_reference:SL2.40ch08 gene_region:56986351-56988984 transcript_region:SL2.40ch08:56986351..56988984- go_terms:GO:0004860 functional_description:"Acyl-CoA-binding protein (AHRD V1 ***- Q8RVT5_PANGI); contains Interpro domain(s) IPR000582 Acyl-CoA-binding protein, ACBP " | 5E-34 |
| 142 | Contig 142 | 826 | 1 | GH204686 | Solyc06g073390.2.1 genomic_reference:SL2.40ch06 gene_region:41616510-41621899 transcript_region:SL2.40ch06:41616510..41621899+ go_terms:GO:0004660 functional_description:"Farnesyltransferase/type I geranylgeranyltransferase alpha subunit (AHRD V1 **** Q6EZ49_CATRO); contains Interpro domain(s) IPR008940 Protein prenyltransferase " | 1E-102 |
| 143 | Contig 143 | 558 | 5 | GH205282 | Solyc02g062340.2.1 genomic_reference:SL2.40ch02 gene_region:28530062-28532470 transcript_region:SL2.40ch02:28530062..28532470- go_terms:GO:0005507 functional_description:"Fructose-bisphosphate aldolase (AHRD V1 ***- Q9SXX4_NICPA); contains Interpro domain(s) IPR000741 Fructose-bisphosphate aldolase, class-I " | 7E-95 |
| 144 | Contig 144 | 640 | 2 | GH203931 | Solyc04g072000.2.1 genomic_reference:SL2.40ch04 gene_region:56659743-56661631 transcript_region:SL2.40ch04:56659743..56661631- go_terms:GO:0008843 functional_description:"Chitinase (AHRD V1 ***- D0QU15_LACSA); contains Interpro domain(s) IPR016283 Glycoside hydrolase, family 19 " | 1E-100 |
| 145 | Contig 145 | 729 | 2 | GH203779 | Solyc10g074440.1.1 evidence_code:10F0H1E1IEG genomic_reference:SL2.40ch10 gene_region:57233453-57234581 transcript_region:SL2.40ch10:57233453..57234581- go_terms:GO:0008843 functional_description:"Endochitinase (Chitinase) (AHRD V1 **** Q43184_SOLTU); contains Interpro domain(s) IPR016283 Glycoside hydrolase, family 19 " | 1E-124 |
| 146 | Contig 146 | 292 | 1 | GH204780 | Solyc04g025540.2.1 genomic_reference:SL2.40ch04 gene_region:21409249-21419957 transcript_region:SL2.40ch04:21409249..21419957+ go_terms:GO:0004048 functional_description:"Anthranilate phosphoribosyltransferase-like protein (AHRD V1 *-** Q3SM27_THIDA); contains Interpro domain(s) IPR000312 Glycosyl transferase, family 3 " | 1E-24 |
| 147 | Contig 147 | 744 | 4 | GH203415 | Solyc01g111510.2.1 genomic_reference:SL2.40ch01 gene_region:89495534-89500705 transcript_region:SL2.40ch01:89495534..89500705- go_terms:GO:0016688 functional_description:"Ascorbate peroxidase (AHRD V1 **** Q8W4V7_CAPAN); contains Interpro domain(s) IPR002207 Plant ascorbate peroxidase " | 1E-133 |
| 148 | Contig 148 | 681 | 4 | GH204226 | Solyc10g084140.1.1 evidence_code:10F0H1E1IEG genomic_reference:SL2.40ch10 gene_region:63108257-63114427 transcript_region:SL2.40ch10:63108257..63114427- go_terms:GO:0006783 functional_description:"Ferrochelatase (AHRD V1 ***- B9RRU8_RICCO); contains Interpro domain(s) IPR001015 Ferrochelatase " | 1E-110 |
| 149 | Contig 149 | 647 | 4 | GH204312 | Solyc02g087150.2.1 genomic_reference:SL2.40ch02 gene_region:44221975-44225097 transcript_region:SL2.40ch02:44221975..44225097+ go_terms:GO:0030001 functional_description:"Heavy-metal-associated domain-containing protein (AHRD V1 *-*- D7M6P1_ARALY); contains Interpro domain(s) IPR006121 Heavy metal transport/detoxification protein " | 3E-51 |
| 150 | Contig 150 | 1025 | 4 | GH204054 | Solyc01g100200.2.1 genomic_reference:SL2.40ch01 gene_region:82020197-82022661 transcript_region:SL2.40ch01:82020197..82022661- go_terms:GO:0003700 functional_description:"GRAS family transcription factor (AHRD V1 **-* B9GJL6_POPTR); contains Interpro domain(s) IPR005202 GRAS transcription factor " | 0 |
| 151 | Contig 151 | 753 | 4 | GH204844 | Solyc04g074450.1.1 evidence_code:10F0H1E1IEG genomic_reference:SL2.40ch04 gene_region:58031412-58032356 transcript_region:SL2.40ch04:58031412..58032356- functional_description:"Phi-1 protein (Fragment) (AHRD V1 ***- Q4LAX0_CAPCH); contains Interpro domain(s) IPR006766 Phosphate-induced protein 1 conserved region " | 1E-145 |
| 152 | Contig 152 | 403 | 4 | GH203787 | Solyc08g078510.2.1 genomic_reference:SL2.40ch08 gene_region:59454948-59456978 transcript_region:SL2.40ch08:59454948..59456978- functional_description:"GRAM-containing/ABA-responsive protein (Fragment) (AHRD V1 *--- C6FBP1_PSEMZ); contains Interpro domain(s) IPR004182 GRAM " | 5E-78 |
| 153 | Contig 153 | 381 | 4 | GH204697 | Solyc02g088690.2.1 genomic_reference:SL2.40ch02 gene_region:45257865-45259203 transcript_region:SL2.40ch02:45257865..45259203- go_terms:GO:0003979 functional_description:"UDP-glucose 6-dehydrogenase (AHRD V1 **** B6TBY8_MAIZE); contains Interpro domain(s) IPR001732 UDP-glucose/GDP-mannose dehydrogenase, N-terminal " | 3E-60 |
| 154 | Contig 154 | 776 | 3 | GH204297 | Solyc08g062910.2.1 genomic_reference:SL2.40ch08 gene_region:49531008-49535044 transcript_region:SL2.40ch08:49531008..49535044- go_terms:GO:0003746 functional_description:"Elongation factor EF-2 (AHRD V1 **** Q9SGT4_ARATH); contains Interpro domain(s) IPR000795 Protein synthesis factor, GTP-binding " | 1E-136 |
| 155 | Contig 155 | 387 | 1 | GH204716 | Solyc12g098640.1.1 evidence_code:10F0H1E1IEG genomic_reference:SL2.40ch12 gene_region:64422393-64426637 transcript_region:SL2.40ch12:64422393..64426637- go_terms:GO:0047793 functional_description:"Cycloeucalenol cycloisomerase (AHRD V1 **-* B6TK44_MAIZE)" | 2E-49 |
| 156 | Contig 156 | 882 | 4 | GH204750 | Solyc01g099770.2.1 genomic_reference:SL2.40ch01 gene_region:81675491-81677536 transcript_region:SL2.40ch01:81675491..81677536+ go_terms:GO:0005515 functional_description:"Translationally-controlled tumor protein homolog (AHRD V1 ***- B5XDL0_SALSA); contains Interpro domain(s) IPR001983 Translationally controlled tumour-associated TCTP " | 1E-80 |
| 157 | Contig 157 | 350 | 4 | GH204368 | Solyc07g054850.2.1 genomic_reference:SL2.40ch07 gene_region:60355806-60357323 transcript_region:SL2.40ch07:60355806..60357323+ functional_description:"Uncharacterized GPI-anchored protein At4g28100 (AHRD V1 ***- UGPI7_ARATH)" | 5E-12 |
| 158 | Contig 158 | 341 | 4 | GH204628 | Solyc01g099840.2.1 genomic_reference:SL2.40ch01 gene_region:81745463-81747550 transcript_region:SL2.40ch01:81745463..81747550+ functional_description:"Auxin-repressed protein (AHRD V1 ***- B4FA62_MAIZE); contains Interpro domain(s) IPR008406 Dormancyauxin associated " | 2E-42 |
| 159 | Contig 159 | 827 | 3 | GH204858 | Solyc01g107640.2.1 genomic_reference:SL2.40ch01 gene_region:86881312-86885702 transcript_region:SL2.40ch01:86881312..86885702- go_terms:GO:0042803,GO:0015088,GO:0005381 functional_description:"Tic21 (AHRD V1 **-- Q09S70_PEA)" | 1E-66 |
| 160 | Contig 160 | 283 | 1 | GH205230 | Solyc01g107640.2.1 genomic_reference:SL2.40ch01 gene_region:86881312-86885702 transcript_region:SL2.40ch01:86881312..86885702- go_terms:GO:0042803,GO:0015088,GO:0005381 functional_description:"Tic21 (AHRD V1 **-- Q09S70_PEA)" | 1E-46 |
| 161 | Contig 161 | 334 | 4 | GH203848 | Solyc01g095620.2.1 genomic_reference:SL2.40ch01 gene_region:78589232-78591027 transcript_region:SL2.40ch01:78589232..78591027- go_terms:GO:0080043,GO:0080044 functional_description:"UDP-glucosyltransferase (AHRD V1 **-* B8QI32_9MAGN); contains Interpro domain(s) IPR002213 UDP-glucuronosyl/UDP-glucosyltransferase " | 4E-52 |
| 162 | Contig 162 | 292 | 4 | GH204600 | Solyc09g007640.2.1 genomic_reference:SL2.40ch09 gene_region:1208268-1210947 transcript_region:SL2.40ch09:1208268..1210947+ go_terms:GO:0042802 functional_description:"Serine carboxypeptidase (AHRD V1 ***- A8HP98_CHLRE); contains Interpro domain(s) IPR001563 Peptidase S10, serine carboxypeptidase " | 7E-49 |
| 163 | Contig 163 | 580 | 3 | GH205111 | Solyc01g095430.2.1 genomic_reference:SL2.40ch01 gene_region:78441004-78444296 transcript_region:SL2.40ch01:78441004..78444296- functional_description:"Threonine endopeptidase (AHRD V1 **-- B6TCN7_MAIZE)" | 1E-68 |
| 164 | Contig 164 | 533 | 1 | GH204387 | Solyc05g012570.2.1 genomic_reference:SL2.40ch05 gene_region:5796042-5797877 transcript_region:SL2.40ch05:5796042..5797877- functional_description:"Unknown Protein (AHRD V1)" | 5E-15 |
| 165 | Contig 165 | 763 | 2 | GH204373 | Solyc06g063240.2.1 genomic_reference:SL2.40ch06 gene_region:36331992-36336504 transcript_region:SL2.40ch06:36331992..36336504+ functional_description:"Os03g0169000 protein (Fragment) (AHRD V1 **-- Q0DUS7_ORYSJ); contains Interpro domain(s) IPR004348 Protein of unknown function DUF246, plant " | 1E-138 |
| 166 | Contig 166 | 321 | 2 | GH204601 | Solyc11g005450.1.1 evidence_code:10F1H1E1IEG genomic_reference:SL2.40ch11 gene_region:359146-361493 transcript_region:SL2.40ch11:359146..361493- go_terms:GO:0005634 functional_description:"Transcription elongation factor A protein 3 (AHRD V1 *--- TCEA3_BOVIN); contains Interpro domain(s) IPR010990 Transcription elongation factor, TFIIS/elongin A/CRSP70, N-terminal " | 5E-36 |
| 167 | Contig 167 | 653 | 2 | GH203938 | Solyc06g071790.2.1 genomic_reference:SL2.40ch06 gene_region:40601620-40603444 transcript_region:SL2.40ch06:40601620..40603444- go_terms:GO:0003746 functional_description:"Elongation factor Tu (AHRD V1 **** D7MFK2_ARALY); contains Interpro domain(s) IPR004541 Translation elongation factor EFTu/EF1A, bacterial and organelle " | 3E-72 |
| 168 | Contig 168 | 317 | 1 | GH204138 | Solyc09g075150.2.1 genomic_reference:SL2.40ch09 gene_region:62444486-62447482 transcript_region:SL2.40ch09:62444486..62447482+ go_terms:GO:0003735,GO:0008201 functional_description:"60S ribosomal protein L22-2 (AHRD V1 ***- D7L3W0_ARALY); contains Interpro domain(s) IPR002671 Ribosomal protein L22e " | 2E-28 |
| 169 | Contig 169 | 546 | 1 | GH204953 | ********* |  |
| 170 | Contig 170 | 696 | 2 | GH204286 | Solyc07g053280.2.1 genomic_reference:SL2.40ch07 gene_region:59072715-59077834 transcript_region:SL2.40ch07:59072715..59077834- go_terms:GO:0000287,GO:0070402 functional_description:"Ketol-acid reductoisomerase (AHRD V1 ***- D6QSY0_CATRO); contains Interpro domain(s) IPR016206 Ketol-acid reductoisomerase, plant " | 1E-119 |
| 171 | Contig 171 | 323 | 2 | GH204069 | Solyc01g096990.2.1 genomic_reference:SL2.40ch01 gene_region:79733061-79738750 transcript_region:SL2.40ch01:79733061..79738750+ go_terms:GO:0005089 functional_description:"Guanine nucleotide exchange factor Vps9 (AHRD V1 **-* C5JGG6_AJEDS); contains Interpro domain(s) IPR013995 Vacuolar sorting protein 9, subgroup " | 9E-57 |
| 172 | Contig 172 | 433 | 4 | GH203561 | Solyc06g071870.2.1 genomic_reference:SL2.40ch06 gene_region:40668511-40671232 transcript_region:SL2.40ch06:40668511..40671232- go_terms:GO:0003735 functional_description:"60S ribosomal protein L17 (AHRD V1 ***- D7KV33_ARALY); contains Interpro domain(s) IPR005721 Ribosomal protein L22/L17, eukaryotic/archaeal " | 3E-70 |
| 173 | Contig 173 | 479 | 2 | GH203542 | Solyc11g007920.1.1 evidence_code:10F1H1E1IEG genomic_reference:SL2.40ch11 gene_region:2160950-2161384 transcript_region:SL2.40ch11:2160950..2161384- go_terms:GO:0005515,GO:0003677 functional_description:"Histone H2B (AHRD V1 ***- A5BCC0_VITVI); contains Interpro domain(s) IPR000558 Histone H2B " | 3E-44 |
| 174 | Contig 174 | 778 | 2 | GH204906 | Solyc05g055440.1.1 evidence_code:10F1H1E1IEG genomic_reference:SL2.40ch05 gene_region:64237122-64237565 transcript_region:SL2.40ch05:64237122..64237565- go_terms:GO:0005515,GO:0003677 functional_description:"Histone H2B (AHRD V1 ***- Q2XPW1_SOLTU); contains Interpro domain(s) IPR000558 Histone H2B " | 3E-44 |
| 175 | Contig 175 | 161 | 4 | GH203645 | Solyc07g062700.2.1 genomic_reference:SL2.40ch07 gene_region:62603490-62608714 transcript_region:SL2.40ch07:62603490..62608714- go_terms:GO:0016021,GO:0005509 functional_description:"Sodium/calcium exchanger family protein (AHRD V1 ***- D7KKF3_ARALY); contains Interpro domain(s) IPR004837 Sodium/calcium exchanger membrane region " | 5E-25 |
| 176 | Contig 176 | 324 | 3 | GH204482 | Solyc09g057650.2.1 genomic_reference:SL2.40ch09 gene_region:45675783-45678653 transcript_region:SL2.40ch09:45675783..45678653- go_terms:GO:0003735,GO:0005515 functional_description:"40S ribosomal protein S8 (AHRD V1 ***- Q3HRZ6_SOLTU); contains Interpro domain(s) IPR001047 Ribosomal protein S8e " | 3E-42 |
| 177 | Contig 177 | 126 | 1 | GH203465 | ********* |  |
| 178 | Contig 178 | 402 | 3 | GH203956 | Solyc11g071760.1.1 evidence_code:10F1H1E1IEG genomic_reference:SL2.40ch11 gene_region:52225377-52225973 transcript_region:SL2.40ch11:52225377..52225973- go_terms:GO:0005509 functional_description:"Calmodulin-like protein (AHRD V1 ***- Q0VJ70_DATME); contains Interpro domain(s) IPR018248 EF hand " | 8E-69 |
| 179 | Contig 179 | 403 | 1 | GH204152 | Solyc02g094000.1.1 evidence_code:10F1H1E1IEG genomic_reference:SL2.40ch02 gene_region:49243310-49243735 transcript_region:SL2.40ch02:49243310..49243735+ go_terms:GO:0005509 functional_description:"Calmodulin-like protein (AHRD V1 ***- Q0VJ70_DATME); contains Interpro domain(s) IPR011992 EF-Hand type " | 8E-64 |
| 180 | Contig 180 | 626 | 2 | GH204693 | Solyc04g008810.2.1 genomic_reference:SL2.40ch04 gene_region:2432009-2434241 transcript_region:SL2.40ch04:2432009..2434241+ go_terms:GO:0005840 functional_description:"40S ribosomal protein S26 (AHRD V1 ***- B6TXN6_MAIZE); contains Interpro domain(s) IPR000892 Ribosomal protein S26e " | 1E-33 |
| 181 | Contig 181 | 501 | 2 | GH204059 | Solyc06g007470.2.1 genomic_reference:SL2.40ch06 gene_region:1479427-1482088 transcript_region:SL2.40ch06:1479427..1482088- go_terms:GO:0005840 functional_description:"40S ribosomal protein S26 (AHRD V1 ***- B6TXN6_MAIZE); contains Interpro domain(s) IPR000892 Ribosomal protein S26e " | 5E-34 |
| 182 | Contig 182 | 305 | 1 | GH204955 | ********* |  |
| 183 | Contig 183 | 576 | 1 | GH204988 | Solyc10g083460.1.1 evidence_code:10F1H1E1IEG genomic_reference:SL2.40ch10 gene_region:62577522-62578001 transcript_region:SL2.40ch10:62577522..62578001+ go_terms:GO:0008270 functional_description:"Zinc finger A20 and AN1 domain-containing stress-associated protein 6 (AHRD V1 *-*- C1BNQ4_9MAXI); contains Interpro domain(s) IPR000058 Zinc finger, AN1-type " | 1E-15 |
| 184 | Contig 184 | 316 | 1 | GH203965 | Solyc12g010860.1.1 evidence_code:10F1H1E1IEG genomic_reference:SL2.40ch12 gene_region:3781557-3784182 transcript_region:SL2.40ch12:3781557..3784182- go_terms:GO:0019904 functional_description:"14-3-3 protein beta/alpha (AHRD V1 **-- 1433B_XENTR); contains Interpro domain(s) IPR000308 14-3-3 protein " | 1E-50 |
| 185 | Contig 185 | 714 | 1 | GH204276 | Solyc03g026220.2.1 genomic_reference:SL2.40ch03 gene_region:8010313-8012274 transcript_region:SL2.40ch03:8010313..8012274+ go_terms:GO:0005515 functional_description:"BCL-2 binding anthanogene-1 (AHRD V1 **** B4FV61_MAIZE); contains Interpro domain(s) IPR003103 Apoptosis regulator Bcl-2 protein, BAG " | 3E-70 |
| 186 | Contig 186 | 920 | 3 | GH203748 | Solyc10g083830.1.1 evidence_code:10F0H1E1IEG genomic_reference:SL2.40ch10 gene_region:62894379-62902328 transcript_region:SL2.40ch10:62894379..62902328- go_terms:GO:0008152,GO:0005524,GO:0006807,GO:0003824 functional_description:"Glutamine synthetase I (AHRD V1 ***- Q9SC91_MEDTR); contains Interpro domain(s) IPR008146 Glutamine synthetase, catalytic region " | 1E-158 |
| 187 | Contig 187 | 829 | 3 | GH205112 | Solyc08g066210.2.1 genomic_reference:SL2.40ch08 gene_region:51851957-51855494 transcript_region:SL2.40ch08:51851957..51855494- go_terms:GO:0004675 functional_description:"Receptor like kinase, RLK" | 1E-141 |
| 188 | Contig 188 | 710 | 3 | GH204569 | Solyc12g006700.1.1 evidence_code:10F1H1E1IEG genomic_reference:SL2.40ch12 gene_region:1165391-1166395 transcript_region:SL2.40ch12:1165391..1166395- functional_description:"Os07g0419800 protein (Fragment) (AHRD V1 *--- Q0D6Y1_ORYSJ); contains Interpro domain(s) IPR012866 Protein of unknown function DUF1644 " | 1E-102 |
| 189 | Contig 189 | 765 | 3 | GH205050 | Solyc07g047670.2.1 genomic_reference:SL2.40ch07 gene_region:56216949-56224432 transcript_region:SL2.40ch07:56216949..56224432+ go_terms:GO:0003713 functional_description:"Pescadillo homolog 1 (AHRD V1 ***- B2RDF2_HUMAN); contains Interpro domain(s) IPR010613 Pescadillo, N-terminal " | 1E-136 |
| 190 | Contig 190 | 716 | 3 | GH203446 | Solyc02g067920.2.1 genomic_reference:SL2.40ch02 gene_region:32585049-32590884 transcript_region:SL2.40ch02:32585049..32590884+ functional_description:"Abhydrolase domain-containing protein FAM108B1 (AHRD V1 *--- C0HAS5_SALSA)" | 1E-132 |
| 191 | Contig 191 | 884 | 3 | GH204951 | Solyc09g091250.2.1 genomic_reference:SL2.40ch09 gene_region:65895466-65898011 transcript_region:SL2.40ch09:65895466..65898011+ go_terms:GO:0006355 functional_description:"GATA transcription factor 1 (AHRD V1 *-*- Q0WTQ5_ARATH); contains Interpro domain(s) IPR000679 Zinc finger, GATA-type " | 3E-95 |
| 192 | Contig 192 | 712 | 3 | GH204225 | Solyc12g009650.1.1 evidence_code:10F0H1E1IEG genomic_reference:SL2.40ch12 gene_region:2922153-2923552 transcript_region:SL2.40ch12:2922153..2923552- go_terms:GO:0005199,GO:0006869 functional_description:"Proline rich protein (Fragment) (AHRD V1 *--- P93274_MALDO); contains Interpro domain(s) IPR013770 Plant lipid transfer protein and hydrophobic protein, helical " | 2E-28 |
| 193 | Contig 193 | 612 | 3 | GH205012 | Solyc03g096780.1.1 evidence_code:10F1H1E1IEG genomic_reference:SL2.40ch03 gene_region:52568046-52568387 transcript_region:SL2.40ch03:52568046..52568387- functional_description:"Unknown Protein (AHRD V1)" | 2E-32 |
| 194 | Contig 194 | 847 | 3 | GH203691 | Solyc12g006320.1.1 evidence_code:10F0H1E1IEG genomic_reference:SL2.40ch12 gene_region:841803-847655 transcript_region:SL2.40ch12:841803..847655- go_terms:GO:0008026,GO:0005524 functional_description:"ATP-dependent RNA helicase (AHRD V1 *-*- Q6L724_HORVU); contains Interpro domain(s) IPR011545 DNA/RNA helicase, DEAD/DEAH box type, N-terminal " | 1E-41 |
| 195 | Contig 195 | 587 | 3 | GH204282 | Solyc12g013810.1.1 evidence_code:10F1H1E1IEG genomic_reference:SL2.40ch12 gene_region:4597746-4599971 transcript_region:SL2.40ch12:4597746..4599971- go_terms:GO:0045454 functional_description:"Thioredoxin (AHRD V1 ***- A7LNX7_9CARY); contains Interpro domain(s) IPR005746 Thioredoxin " | 9E-76 |
| 196 | Contig 196 | 570 | 3 | GH203560 | Solyc11g012700.1.1 evidence_code:10F0H1E1IEG genomic_reference:SL2.40ch11 gene_region:5469691-5473409 transcript_region:SL2.40ch11:5469691..5473409+ go_terms:GO:0055085 functional_description:"Oligopeptide transporter 9 (AHRD V1 ***- B6SVF4_MAIZE); contains Interpro domain(s) IPR004648 Tetrapeptide transporter, OPT1/isp4 " | 1E-115 |
| 197 | Contig 197 | 570 | 3 | GH203668 | Solyc01g108240.2.1 genomic_reference:SL2.40ch01 gene_region:87368287-87369574 transcript_region:SL2.40ch01:87368287..87369574+ go_terms:GO:0003677 functional_description:"Ethylene responsive transcription factor 2b (AHRD V1 *-*- C0J9I6_9ROSA); contains Interpro domain(s) IPR001471 Pathogenesis-related transcriptional factor and ERF, DNA-binding " | 9E-54 |
| 198 | Contig 198 | 630 | 3 | GH204445 | Solyc04g082140.2.1 genomic_reference:SL2.40ch04 gene_region:63511091-63515964 transcript_region:SL2.40ch04:63511091..63515964- go_terms:GO:0055114 functional_description:"Laccase-22 (AHRD V1 **-- LAC22_ORYSJ); contains Interpro domain(s) IPR001117 Multicopper oxidase, type 1 " | 1E-109 |
| 199 | Contig 199 | 606 | 3 | GH203915 | Solyc01g105560.2.1 genomic_reference:SL2.40ch01 gene_region:85458577-85463185 transcript_region:SL2.40ch01:85458577..85463185+ go_terms:GO:0009231 functional_description:"3 4-dihydroxy-2-butanone 4-phosphate synthase (AHRD V1 ***- D7CLR6_SYNLT); contains Interpro domain(s) IPR000422 DHBP synthase RibB " | 2E-96 |
| 200 | Contig 200 | 542 | 3 | GH203623 | Solyc08g078010.2.1 genomic_reference:SL2.40ch08 gene_region:59052085-59055703 transcript_region:SL2.40ch08:59052085..59055703- go_terms:GO:0005840 functional_description:"50S ribosomal protein L19 (AHRD V1 *-*- B6T8M1_MAIZE); contains Interpro domain(s) IPR001857 Ribosomal protein L19 " | 2E-57 |
| 201 | Contig 201 | 545 | 3 | GH203427 | Solyc02g079950.2.1 genomic_reference:SL2.40ch02 gene_region:38907616-38909519 transcript_region:SL2.40ch02:38907616..38909519- go_terms:GO:0019898 functional_description:"Oxygen-evolving enhancer protein 3 (AHRD V1 ***- Q7Y1T5_PEA); contains Interpro domain(s) IPR008797 Photosystem II oxygen evolving complex protein PsbQ " | 2E-76 |
| 202 | Contig 202 | 546 | 3 | GH204053 | Solyc04g081440.2.1 genomic_reference:SL2.40ch04 gene_region:63008173-63012837 transcript_region:SL2.40ch04:63008173..63012837- go_terms:GO:0005515,GO:0004564 functional_description:"Neutral invertase like protein (AHRD V1 ***- Q67XD9_ARATH); contains Interpro domain(s) IPR006937 Plant neutral invertase " | 1E-96 |
| 203 | Contig 203 | 514 | 3 | GH204330 | Solyc02g090200.2.1 genomic_reference:SL2.40ch02 gene_region:46403055-46408254 transcript_region:SL2.40ch02:46403055..46408254- functional_description:"Cell division cycle associated 7 (AHRD V1 *-*- Q5M7M4_XENTR); contains Interpro domain(s) IPR018866 Cell division cycle-associated protein " | 9E-92 |
| 204 | Contig 204 | 472 | 3 | GH204808 | Solyc07g065120.2.1 genomic_reference:SL2.40ch07 gene_region:64242568-64249740 transcript_region:SL2.40ch07:64242568..64249740+ go_terms:GO:0016301 functional_description:"Glycerophosphoryl diester phosphodiesterase family protein (AHRD V1 ***- D7MKI0_ARALY); contains Interpro domain(s) IPR017946 PLC-like phosphodiesterase, TIM beta/alpha-barrel domain " | 2E-80 |
| 205 | Contig 205 | 530 | 3 | GH204413 | Solyc02g069850.2.1 genomic_reference:SL2.40ch02 gene_region:34251255-34253551 transcript_region:SL2.40ch02:34251255..34253551+ go_terms:GO:0003735 functional_description:"40S ribosomal protein S25-1 (AHRD V1 ***- B4FG22_MAIZE); contains Interpro domain(s) IPR004977 Ribosomal protein S25 " | 2E-26 |
| 206 | Contig 206 | 554 | 3 | GH205317 | Solyc04g014720.2.1 genomic_reference:SL2.40ch04 gene_region:4982850-4985110 transcript_region:SL2.40ch04:4982850..4985110+ go_terms:GO:0003735,GO:0003723 functional_description:"60S ribosomal protein L6 (AHRD V1 ***- B9RHH3_RICCO); contains Interpro domain(s) IPR000915 Ribosomal protein L6E " | 2E-23 |
| 207 | Contig 207 | 417 | 3 | GH204034 | Solyc09g019980.2.1 genomic_reference:SL2.40ch09 gene_region:17885037-17890116 transcript_region:SL2.40ch09:17885037..17890116+ functional_description:"Ferric-chelate reductase 1 (AHRD V1 *--- C0HB47_SALSA); contains Interpro domain(s) IPR006593 Cytochrome b561/ferric reductase transmembrane " | 6E-78 |
| 208 | Contig 208 | 406 | 3 | GH204770 | Solyc01g080280.2.1 genomic_reference:SL2.40ch01 gene_region:72039739-72045026 transcript_region:SL2.40ch01:72039739..72045026+ go_terms:GO:0004356 functional_description:"Glutamine synthetase (AHRD V1 ***- B5LAU9_CAPAN); contains Interpro domain(s) IPR008146 Glutamine synthetase, catalytic region " | 5E-64 |
| 209 | Contig 209 | 459 | 3 | GH203537 | ********* |  |
| 210 | Contig 210 | 495 | 3 | GH205016 | Solyc07g062190.2.1 genomic_reference:SL2.40ch07 gene_region:62214361-62218516 transcript_region:SL2.40ch07:62214361..62218516+ go_terms:GO:0005840 functional_description:"50S ribosomal protein L13 (AHRD V1 ***- B6SVH3_MAIZE); contains Interpro domain(s) IPR005823 Ribosomal protein L13, bacterial-type " | 2E-74 |
| 211 | Contig 211 | 781 | 3 | GH204124 | Solyc01g010580.2.1 genomic_reference:SL2.40ch01 gene_region:5590470-5593394 transcript_region:SL2.40ch01:5590470..5593394+ go_terms:GO:0003735 functional_description:"Ribosomal protein (AHRD V1 ***- B3TM27_ELAGV); contains Interpro domain(s) IPR002942 RNA-binding S4 IPR018079 Ribosomal protein S4, conserved site IPR001912 Ribosomal protein S4 " | 2E-99 |
| 212 | Contig 212 | 462 | 3 | GH204701 | Solyc12g014010.1.1 evidence_code:10F0H1E1IEG genomic_reference:SL2.40ch12 gene_region:4844617-4847217 transcript_region:SL2.40ch12:4844617..4847217+ go_terms:GO:0008152 functional_description:"Glucosyltransferase (AHRD V1 ***- Q8RU72_TOBAC); contains Interpro domain(s) IPR002213 UDP-glucuronosyl/UDP-glucosyltransferase " | 2E-44 |
| 213 | Contig 213 | 748 | 2 | GH205218 | Solyc04g076980.2.1 genomic_reference:SL2.40ch04 gene_region:59539119-59540374 transcript_region:SL2.40ch04:59539119..59540374+ go_terms:GO:0004675 functional_description:"LRR receptor-like serine/threonine-protein kinase, RLP" | 1E-103 |
| 214 | Contig 214 | 630 | 1 | GH203908 | Solyc08g066210.2.1 genomic_reference:SL2.40ch08 gene_region:51851957-51855494 transcript_region:SL2.40ch08:51851957..51855494- go_terms:GO:0004675 functional_description:"Receptor like kinase, RLK" | 1E-117 |
| 215 | Contig 215 | 402 | 3 | GH203730 | Solyc04g073990.2.1 genomic_reference:SL2.40ch04 gene_region:57610227-57612650 transcript_region:SL2.40ch04:57610227..57612650- go_terms:GO:0005544 functional_description:"Annexin (AHRD V1 ***- Q42657_CAPAN); contains Interpro domain(s) IPR009118 Annexin, type plant " | 6E-59 |
| 216 | Contig 216 | 386 | 3 | GH203945 | Solyc05g055160.2.1 genomic_reference:SL2.40ch05 gene_region:64069121-64073511 transcript_region:SL2.40ch05:64069121..64073511- go_terms:GO:0050750 functional_description:"DNAJ chaperone (AHRD V1 ***- B0W7V8_CULQU); contains Interpro domain(s) IPR003095 Heat shock protein DnaJ " | 2E-60 |
| 217 | Contig 217 | 600 | 3 | GH203960 | Solyc02g086810.1.1 evidence_code:10F0H0E1IEG genomic_reference:SL2.40ch02 gene_region:44004622-44005443 transcript_region:SL2.40ch02:44004622..44005443+ functional_description:"Unknown Protein (AHRD V1); contains Interpro domain(s) IPR012442 Protein of unknown function DUF1645 " | 3E-55 |
| 218 | Contig 218 | 677 | 2 | GH203528 | Solyc09g075290.2.1 genomic_reference:SL2.40ch09 gene_region:62517630-62519911 transcript_region:SL2.40ch09:62517630..62519911+ go_terms:GO:0003735,GO:0003723 functional_description:"Ribosomal protein L18 (AHRD V1 ***- C5X0Q5_SORBI); contains Interpro domain(s) IPR000039 Ribosomal protein L18e " | 2E-83 |
| 219 | Contig 219 | 479 | 1 | GH204522 | Solyc01g099900.2.1 genomic_reference:SL2.40ch01 gene_region:81786875-81789836 transcript_region:SL2.40ch01:81786875..81789836+ go_terms:GO:0003735,GO:0003723 functional_description:"Ribosomal protein L18 (AHRD V1 ***- C5X0Q5_SORBI); contains Interpro domain(s) IPR000039 Ribosomal protein L18e " | 5E-64 |
| 220 | Contig 220 | 439 | 3 | GH205026 | Solyc10g018300.1.1 evidence_code:10F0H1E1IEG genomic_reference:SL2.40ch10 gene_region:7264174-7267205 transcript_region:SL2.40ch10:7264174..7267205- go_terms:GO:0004802,GO:0005515 functional_description:"Transketolase 1 (AHRD V1 **** O78327_CAPAN); contains Interpro domain(s) IPR005478 Bacterial transketolase " | 3E-49 |
| 221 | Contig 221 | 390 | 3 | GH205046 | Solyc01g097270.2.1 genomic_reference:SL2.40ch01 gene_region:79940651-79942014 transcript_region:SL2.40ch01:79940651..79942014- go_terms:GO:0005515 functional_description:"Chitinase (Fragment) (AHRD V1 *--- Q38777_ALLSA); contains Interpro domain(s) IPR000726 Glycoside hydrolase, family 19, catalytic IPR001153 Barwin " | 5E-32 |
| 222 | Contig 222 | 495 | 3 | GH204352 | Solyc11g039650.1.1 evidence_code:10F0H1E1IEG genomic_reference:SL2.40ch11 gene_region:25081015-25107234 transcript_region:SL2.40ch11:25081015..25107234+ go_terms:GO:0005525 functional_description:"Dynamin-2A (AHRD V1 ***- B6UEQ3_MAIZE); contains Interpro domain(s) IPR001401 Dynamin, GTPase region " | 2E-37 |
| 223 | Contig 223 | 761 | 2 | GH203816 | Solyc01g057830.2.1 genomic_reference:SL2.40ch01 gene_region:56934925-56939761 transcript_region:SL2.40ch01:56934925..56939761+ go_terms:GO:0005840,GO:0003723 functional_description:"30S ribosomal protein S1 (AHRD V1 **-- B4FUZ5_MAIZE); contains Interpro domain(s) IPR003029 Ribosomal protein S1, RNA binding domain " | 1E-108 |
| 224 | Contig 224 | 637 | 1 | GH203762 | Solyc09g014400.2.1 genomic_reference:SL2.40ch09 gene_region:6028037-6031223 transcript_region:SL2.40ch09:6028037..6031223+ go_terms:GO:0030246,GO:0005975 functional_description:"Alpha-amylase (AHRD V1 *--- B9LEH8_CHLSY); contains Interpro domain(s) IPR002044 Glycoside hydrolase, carbohydrate-binding " | 2E-41 |
| 225 | Contig 225 | 630 | 3 | GH203437 | Solyc06g076520.1.1 evidence_code:10F1H1E1IEG genomic_reference:SL2.40ch06 gene_region:43936690-43937154 transcript_region:SL2.40ch06:43936690..43937154- go_terms:GO:0051082 functional_description:"class I heat shock protein (AHRD V1 ***- Q69BI7_CARPA); contains Interpro domain(s) IPR002068 Heat shock protein Hsp20 " | 2E-64 |
| 226 | Contig 226 | 541 | 3 | GH204888 | Solyc04g009030.2.1 genomic_reference:SL2.40ch04 gene_region:2597412-2600773 transcript_region:SL2.40ch04:2597412..2600773+ go_terms:GO:0004365 functional_description:"Glyceraldehyde-3-phosphate dehydrogenase (AHRD V1 **** D7UNZ5_BRARC); contains Interpro domain(s) IPR000173 Glyceraldehyde 3-phosphate dehydrogenase " | 5E-59 |
| 227 | Contig 227 | 893 | 2 | GH203954 | Solyc05g006650.2.1 genomic_reference:SL2.40ch05 gene_region:1311134-1316254 transcript_region:SL2.40ch05:1311134..1316254- go_terms:GO:0003700 functional_description:"Transcription factor (AHRD V1 **** D6MKM4_9ASPA); contains Interpro domain(s) IPR011598 Helix-loop-helix DNA-binding " | 1E-128 |
| 228 | Contig 228 | 535 | 1 | GH203525 | Solyc06g069190.2.1 genomic_reference:SL2.40ch06 gene_region:39347730-39352677 transcript_region:SL2.40ch06:39347730..39352677- go_terms:GO:0006508 functional_description:"Nucellin-like aspartic protease (Fragment) (AHRD V1 **-- Q8L886_MAIZE); contains Interpro domain(s) IPR001461 Peptidase A1 " | 7E-93 |
| 229 | Contig 229 | 315 | 3 | GH203516 | Solyc06g074720.2.1 genomic_reference:SL2.40ch06 gene_region:42639045-42643117 transcript_region:SL2.40ch06:42639045..42643117+ go_terms:GO:0005515,GO:0003723 functional_description:"MKI67 FHA domain-interacting nucleolar phosphoprotein-like (AHRD V1 **-- B6THB2_MAIZE); contains Interpro domain(s) IPR012677 Nucleotide-binding, alpha-beta plait " | 2E-26 |
| 230 | Contig 230 | 340 | 3 | GH203892 | Solyc08g016420.2.1 genomic_reference:SL2.40ch08 gene_region:7447438-7455889 transcript_region:SL2.40ch08:7447438..7455889- go_terms:GO:0015631,GO:0051087 functional_description:"Prefoldin subunit 6 (AHRD V1 ***- B6T5S0_MAIZE); contains Interpro domain(s) IPR002777 Prefoldin beta-like " | 1E-42 |
| 231 | Contig 231 | 552 | 2 | GH204913 | Solyc04g011510.2.1 genomic_reference:SL2.40ch04 gene_region:3944572-3949105 transcript_region:SL2.40ch04:3944572..3949105+ go_terms:GO:0004807 functional_description:"Triosephosphate isomerase (AHRD V1 **** Q38JI4_SOLTU); contains Interpro domain(s) IPR000652 Triosephosphate isomerase " | 3E-71 |
| 232 | Contig 232 | 419 | 1 | GH203702 | Solyc01g111120.2.1 genomic_reference:SL2.40ch01 gene_region:89253979-89260194 transcript_region:SL2.40ch01:89253979..89260194- go_terms:GO:0005515,GO:0004807 functional_description:"Triosephosphate isomerase (AHRD V1 **** D6N3G7_GOSHI); contains Interpro domain(s) IPR000652 Triosephosphate isomerase " | 3E-61 |
| 233 | Contig 233 | 962 | 3 | GH204979 | Solyc04g077020.2.1 genomic_reference:SL2.40ch04 gene_region:59583881-59586708 transcript_region:SL2.40ch04:59583881..59586708+ go_terms:GO:0046982,GO:0005200 functional_description:"Tubulin alpha-3 chain (AHRD V1 ***- B6SPX4_MAIZE); contains Interpro domain(s) IPR002452 Alpha tubulin " | 1E-161 |
| 234 | Contig 234 | 283 | 3 | GH203576 | Solyc08g079700.1.1 evidence_code:10F1H1E1IEG genomic_reference:SL2.40ch08 gene_region:60343880-60344377 transcript_region:SL2.40ch08:60343880..60344377+ go_terms:GO:0005515 functional_description:"Zinc finger A20 and AN1 domain-containing stress-associated protein 7 (AHRD V1 ***- SAP7_ARATH); contains Interpro domain(s) IPR000058 Zinc finger, AN1-type " | 2E-50 |
| 235 | Contig 235 | 273 | 3 | GH204424 | ********* |  |
| 236 | Contig 236 | 556 | 2 | GH203727 | Solyc05g014470.2.1 genomic_reference:SL2.40ch05 gene_region:8322381-8324881 transcript_region:SL2.40ch05:8322381..8324881+ go_terms:GO:0004365 functional_description:"Glyceraldehyde 3-phosphate dehydrogenase (AHRD V1 **** Q8LK04_SOLTU); contains Interpro domain(s) IPR000173 Glyceraldehyde 3-phosphate dehydrogenase " | 3E-92 |
| 237 | Contig 237 | 441 | 1 | GH203666 | Solyc03g006140.2.1 genomic_reference:SL2.40ch03 gene_region:791650-799108 transcript_region:SL2.40ch03:791650..799108- go_terms:GO:0017040 functional_description:"Neutral ceramidase (AHRD V1 **** A9YFM2_WHEAT); contains Interpro domain(s) IPR006823 Neutral/alkaline nonlysosomal ceramidase " | 1E-73 |
| 238 | Contig 238 | 269 | 3 | GH203861 | Solyc06g064500.2.1 genomic_reference:SL2.40ch06 gene_region:36549724-36552243 transcript_region:SL2.40ch06:36549724..36552243- go_terms:GO:0030755,GO:0033799 functional_description:"O-methyltransferase (AHRD V1 **** A5HJZ9_VITVI); contains Interpro domain(s) IPR016461 O-methyltransferase, COMT, eukaryota " | 9E-51 |
| 239 | Contig 239 | 269 | 3 | GH203789 | Solyc06g083340.2.1 genomic_reference:SL2.40ch06 gene_region:45068054-45069921 transcript_region:SL2.40ch06:45068054..45069921- functional_description:"Wound-induced basic protein (AHRD V1 ***- PR4_PHAVU); contains Interpro domain(s) IPR012643 Wound-inducible basic " | 1E-21 |
| 240 | Contig 240 | 315 | 3 | GH205253 | ********* |  |
| 241 | Contig 241 | 257 | 3 | GH203441 | Solyc01g060180.2.1 genomic_reference:SL2.40ch01 gene_region:62560881-62565105 transcript_region:SL2.40ch01:62560881..62565105- go_terms:GO:0008152 functional_description:"AMP-dependent synthetase and ligase (AHRD V1 ***- A1U2F4_MARAV); contains Interpro domain(s) IPR000873 AMP-dependent synthetase and ligase " | 1E-15 |
| 242 | Contig 242 | 442 | 3 | GH205206 | Solyc02g063070.2.1 genomic_reference:SL2.40ch02 gene_region:29692418-29699297 transcript_region:SL2.40ch02:29692418..29699297- go_terms:GO:0050815 functional_description:"14-3-3 protein beta/alpha-1 (AHRD V1 **-- 143B1_DANRE); contains Interpro domain(s) IPR000308 14-3-3 protein " | 5E-70 |
| 243 | Contig 243 | 600 | 3 | GH204612 | Solyc02g087300.1.1 evidence_code:10F0H1E1IEG genomic_reference:SL2.40ch02 gene_region:44325520-44326428 transcript_region:SL2.40ch02:44325520..44326428+ go_terms:GO:0005515 functional_description:"Protein transport SEC13-like protein (AHRD V1 **-- Q2PYY1_SOLTU); contains Interpro domain(s) IPR020472 G-protein beta WD-40 repeat, region " | 6E-89 |
| 244 | Contig 244 | 482 | 2 | GH205060 | Solyc03g120710.2.1 genomic_reference:SL2.40ch03 gene_region:63062243-63065253 transcript_region:SL2.40ch03:63062243..63065253+ functional_description:"Harpin-induced protein (AHRD V1 **-- B6TDG9_MAIZE); contains Interpro domain(s) IPR010847 Harpin-induced 1 " | 4E-40 |
| 245 | Contig 245 | 443 | 1 | GH204355 | Solyc01g091530.2.1 genomic_reference:SL2.40ch01 gene_region:76909578-76911211 transcript_region:SL2.40ch01:76909578..76911211- go_terms:GO:0005516 functional_description:"Fasciclin-like arabinogalactan protein 13 (AHRD V1 ***- A9XTL8_GOSHI); contains Interpro domain(s) IPR000782 FAS1 domain " | 6E-63 |
| 246 | Contig 246 | 407 | 2 | GH205222 | Solyc01g098560.2.1 genomic_reference:SL2.40ch01 gene_region:80808104-80812097 transcript_region:SL2.40ch01:80808104..80812097+ go_terms:GO:0016020,GO:0016021 functional_description:"Glucose transporter 8 (AHRD V1 ***- Q2KKJ3_SOLIN); contains Interpro domain(s) IPR003663 Sugar/inositol transporter " | 2E-74 |
| 247 | Contig 247 | 729 | 1 | GH204464 | Solyc11g006970.1.1 evidence_code:10F1H1E1IEG genomic_reference:SL2.40ch11 gene_region:1477794-1483280 transcript_region:SL2.40ch11:1477794..1483280+ functional_description:"Unknown protein DS12 from 2D-PAGE of leaf, chloroplastic (AHRD V1 **-- UP12_ORYSJ)" | 1E-129 |
| 248 | Contig 248 | 220 | 3 | GH203971 | Solyc09g074930.2.1 genomic_reference:SL2.40ch09 gene_region:62225793-62227794 transcript_region:SL2.40ch09:62225793..62227794+ functional_description:"REF-like stress related protein 1 (AHRD V1 *--- Q6XNP4_HEVBR); contains Interpro domain(s) IPR008802 Rubber elongation factor " | 2E-34 |
| 249 | Contig 249 | 220 | 3 | GH203628 | Solyc11g011130.1.1 evidence_code:10F0H1E1IEG genomic_reference:SL2.40ch11 gene_region:4210363-4214352 transcript_region:SL2.40ch11:4210363..4214352- go_terms:GO:0003676,GO:0000166 functional_description:"THO complex subunit 4 (AHRD V1 *--- B6TD71_MAIZE); contains Interpro domain(s) IPR012677 Nucleotide-binding, alpha-beta plait " | 2E-11 |
| 250 | Contig 250 | 399 | 3 | GH205170 | Solyc10g086280.1.1 evidence_code:10F0H1E1IEG genomic_reference:SL2.40ch10 gene_region:64482821-64484140 transcript_region:SL2.40ch10:64482821..64484140- go_terms:GO:0030001 functional_description:"Heavy metal-associated domain containing protein expressed (AHRD V1 *-*- Q10RN8_ORYSJ); contains Interpro domain(s) IPR006121 Heavy metal transport/detoxification protein " | 1E-35 |
| 251 | Contig 251 | 386 | 2 | GH204111 | Solyc06g051810.2.1 genomic_reference:SL2.40ch06 gene_region:31973284-31982663 transcript_region:SL2.40ch06:31973284..31982663- functional_description:"X1 (Fragment) (AHRD V1 **-- Q3T7E6_ZEAMM); contains Interpro domain(s) IPR005380 Region of unknown function XS " | 2E-70 |
| 252 | Contig 252 | 467 | 1 | GH205198 | ********* |  |
| 253 | Contig 253 | 189 | 3 | GH203751 | Solyc03g120320.1.1 evidence_code:10F0H1E1IEG genomic_reference:SL2.40ch03 gene_region:62801895-62802962 transcript_region:SL2.40ch03:62801895..62802962- functional_description:"Kelch-like protein (AHRD V1 *-*- C4Q722_SCHMA); contains Interpro domain(s) IPR015915 Kelch-type beta propeller " | 5E-33 |
| 254 | Contig 254 | 508 | 2 | GH204170 | Solyc12g043110.1.1 evidence_code:10F0H1E1IEG genomic_reference:SL2.40ch12 gene_region:44210946-44216059 transcript_region:SL2.40ch12:44210946..44216059- go_terms:GO:0005524 functional_description:"Heat shock protein 4 (AHRD V1 ***- B6U237_MAIZE); contains Interpro domain(s) IPR013126 Heat shock protein 70 " | 1E-70 |
| 255 | Contig 255 | 758 | 1 | GH205055 | ********* |  |
| 256 | Contig 256 | 636 | 3 | GH204462 | Solyc04g005650.1.1 evidence_code:10F0H1E1IEG genomic_reference:SL2.40ch04 gene_region:419666-420718 transcript_region:SL2.40ch04:419666..420718- go_terms:GO:0005515,GO:0051724 functional_description:"Mitochondrial carrier family (AHRD V1 ***- C1MWU5_MICPS); contains Interpro domain(s) IPR001993 Mitochondrial substrate carrier " | 9E-64 |
| 257 | Contig 257 | 166 | 3 | GH203450 | Solyc03g025990.1.1 evidence_code:10F1H1E1IEG genomic_reference:SL2.40ch03 gene_region:7788120-7788731 transcript_region:SL2.40ch03:7788120..7788731+ functional_description:"Hexulose-6-phosphate isomerase (AHRD V1 **-- Q2RUM7_RHORT)" | 2E-17 |
| 258 | Contig 258 | 466 | 2 | GH204199 | Solyc06g071720.1.1 evidence_code:10F0H1E1IEG genomic_reference:SL2.40ch06 gene_region:40574165-40574611 transcript_region:SL2.40ch06:40574165..40574611- go_terms:GO:0003735 functional_description:"60S ribosomal protein L27A (AHRD V1 ***- B6VC54_VERFO); contains Interpro domain(s) IPR001196 Ribosomal protein L15 " | 2E-75 |
| 259 | Contig 259 | 559 | 1 | GH204658 | Solyc03g112360.1.1 evidence_code:10F0H1E1IEG genomic_reference:SL2.40ch03 gene_region:56836935-56837381 transcript_region:SL2.40ch03:56836935..56837381+ go_terms:GO:0003735 functional_description:"60S ribosomal protein L27A (AHRD V1 ***- B6VC54_VERFO); contains Interpro domain(s) IPR001196 Ribosomal protein L15 " | 3E-63 |
| 260 | Contig 260 | 503 | 2 | GH203796 | Solyc01g005000.2.1 genomic_reference:SL2.40ch01 gene_region:12963-15149 transcript_region:SL2.40ch01:12963..15149+ go_terms:GO:0006536,GO:0030170,GO:0019752 functional_description:"Glutamate decarboxylase (AHRD V1 ***- Q1I1D8_CITSI); contains Interpro domain(s) IPR010107 Glutamate decarboxylase " | 1E-78 |
| 261 | Contig 261 | 772 | 1 | GH204509 | Solyc11g011920.1.1 evidence_code:10F1H1E1IEG genomic_reference:SL2.40ch11 gene_region:4848830-4855228 transcript_region:SL2.40ch11:4848830..4855228- go_terms:GO:0006536,GO:0030170,GO:0019752 functional_description:"Glutamate decarboxylase (AHRD V1 ***- Q8LKR4_TOBAC); contains Interpro domain(s) IPR010107 Glutamate decarboxylase " | 1E-151 |
| 262 | Contig 262 | 267 | 2 | GH203761 | Solyc06g066660.2.1 genomic_reference:SL2.40ch06 gene_region:38274359-38276478 transcript_region:SL2.40ch06:38274359..38276478- go_terms:GO:0003735 functional_description:"Ribosomal protein L37 (AHRD V1 ***- B9RQN1_RICCO); contains Interpro domain(s) IPR001569 Ribosomal protein L37e " | 2E-24 |
| 263 | Contig 263 | 413 | 1 | GH204856 | Solyc03g120780.2.1 genomic_reference:SL2.40ch03 gene_region:63113010-63114120 transcript_region:SL2.40ch03:63113010..63114120- go_terms:GO:0005840 functional_description:"Ribosomal protein L37 (AHRD V1 ***- B9RQN1_RICCO); contains Interpro domain(s) IPR001569 Ribosomal protein L37e " | 2E-46 |
| 264 | Contig 264 | 818 | 2 | GH204918 | Solyc10g078620.1.1 evidence_code:10F1H1E1IEG genomic_reference:SL2.40ch10 gene_region:59701774-59703879 transcript_region:SL2.40ch10:59701774..59703879- go_terms:GO:0003735 functional_description:"Ribosomal protein S5 (AHRD V1 ***- A8J2I5_CHLRE); contains Interpro domain(s) IPR005716 Ribosomal protein S7, eukaryotic/archaeal " | 1E-106 |
| 265 | Contig 265 | 786 | 1 | GH204588 | Solyc10g086150.1.1 evidence_code:10F1H1E1IEG genomic_reference:SL2.40ch10 gene_region:64403180-64404803 transcript_region:SL2.40ch10:64403180..64404803- go_terms:GO:0043047 functional_description:"Single-stranded DNA binding protein (AHRD V1 **-* Q8W214_SOLTU); contains Interpro domain(s) IPR000504 RNA recognition motif, RNP-1 " | 1E-112 |
| 266 | Contig 266 | 767 | 2 | GH205117 | Solyc01g110360.2.1 genomic_reference:SL2.40ch01 gene_region:88844597-88847246 transcript_region:SL2.40ch01:88844597..88847246+ go_terms:GO:0005507 functional_description:"Fructose-bisphosphate aldolase (AHRD V1 ***- B0FPD8_SOLTU); contains Interpro domain(s) IPR000741 Fructose-bisphosphate aldolase, class-I " | 1E-100 |
| 267 | Contig 267 | 269 | 1 | GH204342 | ********* |  |
| 268 | Contig 268 | 480 | 1 | GH204369 | Solyc10g083280.1.1 evidence_code:10F0H1E1IEG genomic_reference:SL2.40ch10 gene_region:62430835-62433599 transcript_region:SL2.40ch10:62430835..62433599+ go_terms:GO:0005840 functional_description:"30S ribosomal protein S11 (AHRD V1 ***- RS11_CAUSK); contains Interpro domain(s) IPR018102 Ribosomal S11, conserved site " | 8E-65 |
| 269 | Contig 269 | 683 | 1 | GH204260 | Solyc09g010100.2.1 genomic_reference:SL2.40ch09 gene_region:3488735-3491402 transcript_region:SL2.40ch09:3488735..3491402- go_terms:GO:0003735,GO:0048027 functional_description:"30S ribosomal protein S11 (AHRD V1 ***- RS11_CAUSK); contains Interpro domain(s) IPR018102 Ribosomal S11, conserved site " | 2E-55 |
| 270 | Contig 270 | 552 | 1 | GH204752 | Solyc06g084360.2.1 genomic_reference:SL2.40ch06 gene_region:45789645-45797146 transcript_region:SL2.40ch06:45789645..45797146+ go_terms:GO:0008270 functional_description:"Baculoviral IAP repeat-containing protein 3 (AHRD V1 *--- B0XJ37_CULQU); contains Interpro domain(s) IPR001841 Zinc finger, RING-type " | 4E-59 |
| 271 | Contig 271 | 897 | 2 | GH204817 | Solyc12g098820.1.1 evidence_code:10F0H1E1IEG genomic_reference:SL2.40ch12 gene_region:64535368-64540452 transcript_region:SL2.40ch12:64535368..64540452+ go_terms:GO:0006468 functional_description:"Receptor-like kinase (AHRD V1 ***- A7VM24_MARPO); contains Interpro domain(s) IPR002290 Serine/threonine protein kinase " | 9E-95 |
| 272 | Contig 272 | 914 | 2 | GH203900 | Solyc06g069120.2.1 genomic_reference:SL2.40ch06 gene_region:39292001-39293674 transcript_region:SL2.40ch06:39292001..39293674+ functional_description:"Unknown Protein (AHRD V1)" | 7E-90 |
| 273 | Contig 273 | 875 | 2 | GH204271 | Solyc10g077130.1.1 evidence_code:10F0H0E1IEG genomic_reference:SL2.40ch10 gene_region:59327643-59328260 transcript_region:SL2.40ch10:59327643..59328260+ functional_description:"Atcambp25-binding protein OF (AHRD V1 ***- D7LGI4_ARALY); contains Interpro domain(s) IPR008889 VQ " | 2E-32 |
| 274 | Contig 274 | 880 | 2 | GH204134 | Solyc09g092510.2.1 genomic_reference:SL2.40ch09 gene_region:66920991-66924952 transcript_region:SL2.40ch09:66920991..66924952+ functional_description:"Unknown Protein (AHRD V1)" | 1E-165 |
| 275 | Contig 275 | 872 | 2 | GH203829 | Solyc01g108500.2.1 genomic_reference:SL2.40ch01 gene_region:87565501-87571455 transcript_region:SL2.40ch01:87565501..87571455- go_terms:GO:0005515 functional_description:"Polyadenylate-binding protein (AHRD V1 **-* PABP_DROME); contains Interpro domain(s) IPR012677 Nucleotide-binding, alpha-beta plait " | 1E-109 |
| 276 | Contig 276 | 812 | 2 | GH203983 | Solyc01g104460.2.1 genomic_reference:SL2.40ch01 gene_region:84688779-84693304 transcript_region:SL2.40ch01:84688779..84693304+ go_terms:GO:0005515 functional_description:"Partner of Y14 and mago (AHRD V1 *-*- WIBG_CULQU); contains Interpro domain(s) IPR015362 Exon junction complex, Pym " | 2E-93 |
| 277 | Contig 277 | 802 | 2 | GH205146 | Solyc11g008360.1.1 evidence_code:10F1H1E1IEG genomic_reference:SL2.40ch11 gene_region:2570463-2575364 transcript_region:SL2.40ch11:2570463..2575364- go_terms:GO:0009451,GO:0003723 functional_description:"Pseudouridine synthase family protein (AHRD V1 ***- D7LCF4_ARALY); contains Interpro domain(s) IPR006145 Pseudouridine synthase, RsuA and RluB/C/D/E/F " | 2E-94 |
| 278 | Contig 278 | 792 | 2 | GH204706 | Solyc03g112200.1.1 evidence_code:10F0H0E1IEG genomic_reference:SL2.40ch03 gene_region:56739563-56740318 transcript_region:SL2.40ch03:56739563..56740318- functional_description:"Unknown Protein (AHRD V1)" | 1E-119 |
| 279 | Contig 279 | 949 | 2 | GH203774 | Solyc02g064960.2.1 genomic_reference:SL2.40ch02 gene_region:30651561-30655131 transcript_region:SL2.40ch02:30651561..30655131+ go_terms:GO:0003677 functional_description:"AP2-like ethylene-responsive transcription factor At1g16060 (AHRD V1 *-*- AP2L1_ARATH); contains Interpro domain(s) IPR001471 Pathogenesis-related transcriptional factor and ERF, DNA-binding " | 5E-31 |
| 280 | Contig 280 | 767 | 2 | GH205159 | Solyc08g075090.2.1 genomic_reference:SL2.40ch08 gene_region:56410976-56415969 transcript_region:SL2.40ch08:56410976..56415969- go_terms:GO:0005840,GO:0015934,GO:0005634,GO:0045449 functional_description:"60S ribosomal protein L7 (AHRD V1 *-*- D7MY49_ARALY); contains Interpro domain(s) IPR005998 Ribosomal protein L7, eukaryotic " | 1E-105 |
| 281 | Contig 281 | 858 | 2 | GH205151 | Solyc12g042060.1.1 evidence_code:10F0H1E1IEG genomic_reference:SL2.40ch12 gene_region:42533149-42537576 transcript_region:SL2.40ch12:42533149..42537576+ go_terms:GO:0019538,GO:0005524,GO:0017111,GO:0006289 functional_description:"ATP-dependent clp protease ATP-binding subunit (AHRD V1 **-- Q85G08_CYAME); contains Interpro domain(s) IPR013093 ATPase associated with various cellular activities, AAA-2 " | 1E-147 |
| 282 | Contig 282 | 883 | 2 | GH203507 | Solyc04g007910.2.1 genomic_reference:SL2.40ch04 gene_region:1574818-1580749 transcript_region:SL2.40ch04:1574818..1580749- go_terms:GO:0005975 functional_description:"Glucan endo-1 3-beta-glucosidase 3 (AHRD V1 ***- B6TLN1_MAIZE); contains Interpro domain(s) IPR013781 Glycoside hydrolase, subgroup, catalytic core " | 1E-48 |
| 283 | Contig 283 | 791 | 2 | GH204811 | Solyc06g035720.2.1 genomic_reference:SL2.40ch06 gene_region:21506013-21509381 transcript_region:SL2.40ch06:21506013..21509381- go_terms:GO:0005515 functional_description:"BCL-2 binding anthanogene-1 (AHRD V1 **** B4FV61_MAIZE); contains Interpro domain(s) IPR003103 Apoptosis regulator Bcl-2 protein, BAG " | 2E-85 |
| 284 | Contig 284 | 670 | 2 | GH203655 | Solyc03g043660.2.1 genomic_reference:SL2.40ch03 gene_region:11111754-11138015 transcript_region:SL2.40ch03:11111754..11138015- go_terms:GO:0004252 functional_description:"Pro-apoptotic serine protease nma111-like protein (AHRD V1 *-** D0NE02_PHYIN); contains Interpro domain(s) IPR009003 Peptidase, trypsin-like serine and cysteine " | 1E-122 |
| 285 | Contig 285 | 740 | 2 | GH204534 | Solyc06g068870.2.1 genomic_reference:SL2.40ch06 gene_region:39083842-39089281 transcript_region:SL2.40ch06:39083842..39089281- go_terms:GO:0016563,GO:0003700 functional_description:"BHLH transcription factor (AHRD V1 *-** A9YWR2_MEDTR); contains Interpro domain(s) IPR011598 Helix-loop-helix DNA-binding " | 1E-110 |
| 286 | Contig 286 | 770 | 2 | GH203579 | Solyc08g081250.2.1 genomic_reference:SL2.40ch08 gene_region:61469375-61478079 transcript_region:SL2.40ch08:61469375..61478079- go_terms:GO:0004222 functional_description:"Aminopeptidase N (AHRD V1 ***- D5C0C8_NITHN); contains Interpro domain(s) IPR012779 Peptidase M1, alanyl aminopeptidase " | 1E-131 |
| 287 | Contig 287 | 806 | 2 | GH204608 | Solyc09g009390.2.1 genomic_reference:SL2.40ch09 gene_region:2835367-2840425 transcript_region:SL2.40ch09:2835367..2840425+ go_terms:GO:0016656 functional_description:"Monodehydroascorbate reductase (NADH)-like protein (AHRD V1 **** Q0WUJ1_ARATH); contains Interpro domain(s) IPR013027 FAD-dependent pyridine nucleotide-disulphide oxidoreductase " | 1E-133 |
| 288 | Contig 288 | 852 | 2 | GH204836 | Solyc07g066650.2.1 genomic_reference:SL2.40ch07 gene_region:65228371-65238555 transcript_region:SL2.40ch07:65228371..65238555- go_terms:GO:0043130 functional_description:"DCN1-like protein 2 (AHRD V1 ***- B6TM27_MAIZE); contains Interpro domain(s) IPR014764 Defective in cullin neddylation " | 1E-117 |
| 289 | Contig 289 | 635 | 2 | GH204499 | Solyc03g071710.1.1 evidence_code:10F1H0E1IEG genomic_reference:SL2.40ch03 gene_region:41328764-41329111 transcript_region:SL2.40ch03:41328764..41329111- functional_description:"Self-pruning interacting protein 1 (AHRD V1 ***- Q9FR57_SOLLC)" | 3E-62 |
| 290 | Contig 290 | 635 | 2 | GH204205 | Solyc03g115020.2.1 genomic_reference:SL2.40ch03 gene_region:58930557-58932425 transcript_region:SL2.40ch03:58930557..58932425- functional_description:"Unknown Protein (AHRD V1); contains Interpro domain(s) IPR009500 Protein of unknown function DUF1118 " | 6E-65 |
| 291 | Contig 291 | 772 | 2 | GH205155 | Solyc09g011210.2.1 genomic_reference:SL2.40ch09 gene_region:4553223-4555661 transcript_region:SL2.40ch09:4553223..4555661- go_terms:GO:0008143 functional_description:"RNA binding protein (AHRD V1 **** B6U487_MAIZE); contains Interpro domain(s) IPR012677 Nucleotide-binding, alpha-beta plait " | 9E-61 |
| 292 | Contig 292 | 754 | 2 | GH203613 | Solyc11g005680.1.1 evidence_code:10F1H1E1IEG genomic_reference:SL2.40ch11 gene_region:502496-504749 transcript_region:SL2.40ch11:502496..504749- go_terms:GO:0003735,GO:0005515 functional_description:"40S ribosomal protein S18 (AHRD V1 ***- B4G286_MAIZE); contains Interpro domain(s) IPR001892 Ribosomal protein S13 " | 5E-75 |
| 293 | Contig 293 | 800 | 2 | GH204267 | Solyc10g076990.1.1 evidence_code:10F0H1E1IEG genomic_reference:SL2.40ch10 gene_region:59242945-59251017 transcript_region:SL2.40ch10:59242945..59251017+ go_terms:GO:0032312 functional_description:"Arf-GAP domain and FG repeats-containing protein 1 (AHRD V1 *--- AGFG1_MOUSE); contains Interpro domain(s) IPR001164 Arf GTPase activating protein " | 1E-129 |
| 294 | Contig 294 | 653 | 2 | GH204258 | Solyc11g069000.1.1 evidence_code:10F1H1E1IEG genomic_reference:SL2.40ch11 gene_region:50652125-50657452 transcript_region:SL2.40ch11:50652125..50657452- go_terms:GO:0044267,GO:0006457 functional_description:"T-complex protein 1 subunit beta (AHRD V1 ***- B6T8X5_MAIZE); contains Interpro domain(s) IPR012716 T-complex protein 1, beta subunit " | 2E-69 |
| 295 | Contig 295 | 677 | 2 | GH205087 | Solyc03g112230.2.1 genomic_reference:SL2.40ch03 gene_region:56747879-56752208 transcript_region:SL2.40ch03:56747879..56752208- go_terms:GO:0008270 functional_description:"ZZ type zinc finger domain-containing protein (Fragment) (AHRD V1 *--- C1GFD2_PARBD); contains Interpro domain(s) IPR000270 Octicosapeptide/Phox/Bem1p " | 1E-119 |
| 296 | Contig 296 | 788 | 2 | GH205071 | Solyc10g081590.1.1 evidence_code:10F1H0E1IEG genomic_reference:SL2.40ch10 gene_region:61948462-61951703 transcript_region:SL2.40ch10:61948462..61951703+ go_terms:GO:0032259 functional_description:"N6-adenine-specific DNA methyltransferase 2 (AHRD V1 ***- C1C3V5_RANCA); contains Interpro domain(s) IPR019369 N-6 adenine-specific DNA methylase-related, eukaryotic " | 1E-118 |
| 297 | Contig 297 | 683 | 2 | GH204440 | Solyc11g039840.1.1 evidence_code:10F1H1E1IEG genomic_reference:SL2.40ch11 gene_region:26506797-26509759 transcript_region:SL2.40ch11:26506797..26509759+ go_terms:GO:0008121,GO:0046872 functional_description:"Ubiquinol-cytochrome c reductase iron-sulfur subunit (AHRD V1 **** Q38M54_SOLTU); contains Interpro domain(s) IPR005805 Rieske iron-sulphur protein, C-terminal " | 1E-88 |
| 298 | Contig 298 | 703 | 2 | GH204125 | Solyc10g005360.2.1 genomic_reference:SL2.40ch10 gene_region:261366-264169 transcript_region:SL2.40ch10:261366..264169- go_terms:GO:0045543 functional_description:"Gibberellin 2-beta-dioxygenase 7 (AHRD V1 **-* B6SZM8_MAIZE); contains Interpro domain(s) IPR005123 Oxoglutarate and iron-dependent oxygenase " | 2E-64 |
| 299 | Contig 299 | 638 | 2 | GH204380 | Solyc03g123630.2.1 genomic_reference:SL2.40ch03 gene_region:64478109-64481386 transcript_region:SL2.40ch03:64478109..64481386- go_terms:GO:0005618,GO:0030599 functional_description:"Pectinesterase (AHRD V1 ***- Q564D7_SOLLC); contains Interpro domain(s) IPR000070 Pectinesterase, catalytic " | 9E-96 |
| 300 | Contig 300 | 714 | 2 | GH204055 | Solyc06g074210.2.1 genomic_reference:SL2.40ch06 gene_region:42268413-42276417 transcript_region:SL2.40ch06:42268413..42276417- functional_description:"MRNA decapping enzyme (AHRD V1 *-*- Q45NB8_XENLA); contains Interpro domain(s) IPR010334 Dcp1-like decapping " | 4E-34 |
| 301 | Contig 301 | 661 | 2 | GH204334 | Solyc11g069090.1.1 evidence_code:10F0H1E1IEG genomic_reference:SL2.40ch11 gene_region:50759436-50762771 transcript_region:SL2.40ch11:50759436..50762771+ go_terms:GO:0017111,GO:0016887 functional_description:"ATP-binding cassette protein (AHRD V1 ***- C0NDN3_AJECG); contains Interpro domain(s) IPR003439 ABC transporter-like " | 1E-103 |
| 302 | Contig 302 | 591 | 2 | GH204779 | Solyc00g007270.2.1 genomic_reference:SL2.40ch00 gene_region:6900991-6904901 transcript_region:SL2.40ch00:6900991..6904901- go_terms:GO:0005515,GO:0043023 functional_description:"Translation initiation factor (AHRD V1 ***- A6ZWQ9_YEAS7); contains Interpro domain(s) IPR002769 Translation initiation factor IF6 " | 7E-89 |
| 303 | Contig 303 | 602 | 2 | GH204700 | Solyc01g109940.2.1 genomic_reference:SL2.40ch01 gene_region:88527503-88532712 transcript_region:SL2.40ch01:88527503..88532712- go_terms:GO:0005524,GO:0017111,GO:0005737 functional_description:"26S protease regulatory subunit (AHRD V1 ***- C0NAB1_AJECG); contains Interpro domain(s) IPR005937 26S proteasome subunit P45 " | 1E-107 |
| 304 | Contig 304 | 769 | 2 | GH203535 | Solyc12g056580.1.1 evidence_code:10F0H1E1IEG genomic_reference:SL2.40ch12 gene_region:47881791-47888295 transcript_region:SL2.40ch12:47881791..47888295+ go_terms:GO:0016020,GO:0008270 functional_description:"Cellulose synthase (AHRD V1 ***- B9GTH4_POPTR); contains Interpro domain(s) IPR005150 Cellulose synthase " | 1E-116 |
| 305 | Contig 305 | 699 | 2 | GH205180 | Solyc09g009840.1.1 evidence_code:10F0H0E1IEG genomic_reference:SL2.40ch09 gene_region:3332891-3333757 transcript_region:SL2.40ch09:3332891..3333757- functional_description:"Calcium/calmodulin protein kinase (AHRD V1 *-*- Q84ZT8_TOBAC)" | 4E-91 |
| 306 | Contig 306 | 606 | 2 | GH204800 | Solyc02g090310.1.1 evidence_code:10F0H1E1IEG genomic_reference:SL2.40ch02 gene_region:46465985-46466767 transcript_region:SL2.40ch02:46465985..46466767- go_terms:GO:0045449 functional_description:"Dof zinc finger protein 4 (AHRD V1 *-*- A5HWF3_HORVD); contains Interpro domain(s) IPR003851 Zinc finger, Dof-type " | 2E-81 |
| 307 | Contig 307 | 599 | 2 | GH204118 | Solyc07g007590.1.1 evidence_code:10F1H1E1IEG genomic_reference:SL2.40ch07 gene_region:2239957-2241090 transcript_region:SL2.40ch07:2239957..2241090- go_terms:GO:0008152,GO:0006571 functional_description:"Prephenate dehydrogenase family protein (AHRD V1 ***- D7KDI5_ARALY); contains Interpro domain(s) IPR012070 Arogenate/prephenate dehydrogenase, plant " | 2E-34 |
| 308 | Contig 308 | 566 | 2 | GH205132 | Solyc07g047970.2.1 genomic_reference:SL2.40ch07 gene_region:56506647-56514306 transcript_region:SL2.40ch07:56506647..56514306- go_terms:GO:0000166 functional_description:"Prolactin regulatory element-binding protein (AHRD V1 *--* C0H9F8_SALSA); contains Interpro domain(s) IPR017986 WD40 repeat, region " | 3E-36 |
| 309 | Contig 309 | 756 | 2 | GH204698 | Solyc03g095190.2.1 genomic_reference:SL2.40ch03 gene_region:49598390-49602085 transcript_region:SL2.40ch03:49598390..49602085- go_terms:GO:0008270,GO:0016887 functional_description:"ATP synthase epsilon chain (AHRD V1 ***- A3UHR9_9RHOB); contains Interpro domain(s) IPR001469 ATPase, F1 complex, delta/epsilon subunit " | 1E-64 |
| 310 | Contig 310 | 547 | 2 | GH204314 | Solyc01g079950.2.1 genomic_reference:SL2.40ch01 gene_region:71609788-71611611 transcript_region:SL2.40ch01:71609788..71611611- go_terms:GO:0006508 functional_description:"Xylanase inhibitor (Fragment) (AHRD V1 **-- Q53IQ3_WHEAT); contains Interpro domain(s) IPR001461 Peptidase A1 " | 1E-106 |
| 311 | Contig 311 | 917 | 2 | GH203925 | Solyc09g075460.2.1 genomic_reference:SL2.40ch09 gene_region:62664675-62668645 transcript_region:SL2.40ch09:62664675..62668645+ go_terms:GO:0005975 functional_description:"Polygalacturonase-like protein-like (AHRD V1 ***- Q2XTD7_SOLTU); contains Interpro domain(s) IPR012334 Pectin lyase fold " | 1E-94 |
| 312 | Contig 312 | 543 | 2 | GH204320 | Solyc03g114810.2.1 genomic_reference:SL2.40ch03 gene_region:58741832-58746719 transcript_region:SL2.40ch03:58741832..58746719- go_terms:GO:0016758 functional_description:"Glycosyltransferase (AHRD V1 ***- B9N4D7_POPTR); contains Interpro domain(s) IPR002495 Glycosyl transferase, family 8 " | 1E-99 |
| 313 | Contig 313 | 572 | 2 | GH204548 | Solyc09g010800.2.1 genomic_reference:SL2.40ch09 gene_region:4094901-4096611 transcript_region:SL2.40ch09:4094901..4096611+ go_terms:GO:0046872 functional_description:"Type 2 metallothionein (AHRD V1 ***- B3VKV4_SOLNI); contains Interpro domain(s) IPR000347 Plant metallothionein, family 15 " | 3E-15 |
| 314 | Contig 314 | 632 | 2 | GH204819 | Solyc05g006240.2.1 genomic_reference:SL2.40ch05 gene_region:898981-903747 transcript_region:SL2.40ch05:898981..903747- go_terms:GO:0006457,GO:0031072 functional_description:"Chaperone protein dnaJ 16 (AHRD V1 ***- B6THE8_MAIZE); contains Interpro domain(s) IPR003095 Heat shock protein DnaJ " | 9E-40 |
| 315 | Contig 315 | 529 | 2 | GH205179 | Solyc01g091800.2.1 genomic_reference:SL2.40ch01 gene_region:77119410-77125807 transcript_region:SL2.40ch01:77119410..77125807- go_terms:GO:0000154,GO:0006364 functional_description:"Ribosomal RNA small subunit methyltransferase A (AHRD V1 ***- D6YS21_WADCW); contains Interpro domain(s) IPR011530 rRNA adenine dimethylase " | 5E-82 |
| 316 | Contig 316 | 601 | 2 | GH204156 | Solyc01g096700.2.1 genomic_reference:SL2.40ch01 gene_region:79480993-79483332 transcript_region:SL2.40ch01:79480993..79483332- go_terms:GO:0051082 functional_description:"Chaperone protein dnaJ 1 (AHRD V1 *-*- DNAJ1_THET8); contains Interpro domain(s) IPR015609 Molecular chaperone, heat shock protein, Hsp40, DnaJ " | 1E-74 |
| 317 | Contig 317 | 556 | 2 | GH203876 | Solyc05g051200.1.1 evidence_code:10F0H1E1IEG genomic_reference:SL2.40ch05 gene_region:60588307-60588981 transcript_region:SL2.40ch05:60588307..60588981- go_terms:GO:0016563,GO:0003700 functional_description:"Ethylene-responsive transcription factor 1A (AHRD V1 *-** A9P6A4_MEDTR); contains Interpro domain(s) IPR001471 Pathogenesis-related transcriptional factor and ERF, DNA-binding " | 6E-57 |
| 318 | Contig 318 | 513 | 2 | GH204946 | Solyc01g105560.2.1 genomic_reference:SL2.40ch01 gene_region:85458577-85463185 transcript_region:SL2.40ch01:85458577..85463185+ go_terms:GO:0009231 functional_description:"3 4-dihydroxy-2-butanone 4-phosphate synthase (AHRD V1 ***- D7CLR6_SYNLT); contains Interpro domain(s) IPR000422 DHBP synthase RibB " | 2E-95 |
| 319 | Contig 319 | 600 | 2 | GH204924 | Solyc06g054260.1.1 evidence_code:10F1H1E1IEG genomic_reference:SL2.40ch06 gene_region:33565231-33565857 transcript_region:SL2.40ch06:33565231..33565857- go_terms:GO:0009538 functional_description:"Photosystem I reaction center subunit II (AHRD V1 ***- B4FAW3_MAIZE); contains Interpro domain(s) IPR003685 Photosystem I protein PsaD " | 1E-59 |
| 320 | Contig 320 | 506 | 2 | GH205361 | Solyc03g117270.1.1 evidence_code:10F0H0E1IEG genomic_reference:SL2.40ch03 gene_region:60500607-60501878 transcript_region:SL2.40ch03:60500607..60501878+ functional_description:"F-box family protein (AHRD V1 ***- B9HV09_POPTR); contains Interpro domain(s) IPR001810 Cyclin-like F-box " | 8E-85 |
| 321 | Contig 321 | 548 | 2 | GH203888 | Solyc01g105290.2.1 genomic_reference:SL2.40ch01 gene_region:85274367-85276805 transcript_region:SL2.40ch01:85274367..85276805+ go_terms:GO:0004867 functional_description:"Genomic DNA chromosome 5 P1 clone MSJ1 (AHRD V1 ***- Q9FMG2_ARATH)" | 8E-52 |
| 322 | Contig 322 | 502 | 2 | GH204718 | Solyc02g021440.2.1 genomic_reference:SL2.40ch02 gene_region:13422223-13429172 transcript_region:SL2.40ch02:13422223..13429172- go_terms:GO:0004691 functional_description:"CBL-interacting protein kinase 04 (AHRD V1 **** A5BQ82_VITVI); contains Interpro domain(s) IPR002290 Serine/threonine protein kinase " | 8E-45 |
| 323 | Contig 323 | 503 | 2 | GH204715 | Solyc01g103540.2.1 genomic_reference:SL2.40ch01 gene_region:83887917-83893923 transcript_region:SL2.40ch01:83887917..83893923+ functional_description:"YTH domain family 2 (Predicted) (AHRD V1 *-*- B2GUU1_RAT); contains Interpro domain(s) IPR007275 YT521-B-like protein " | 5E-97 |
| 324 | Contig 324 | 525 | 2 | GH203590 | Solyc06g073050.2.1 genomic_reference:SL2.40ch06 gene_region:41395982-41399877 transcript_region:SL2.40ch06:41395982..41399877+ go_terms:GO:0045449 functional_description:"NAC domain protein IPR003441 (AHRD V1 *-*- B9IDH0_POPTR); contains Interpro domain(s) IPR003441 No apical meristem (NAM) protein " | 3E-99 |
| 325 | Contig 325 | 587 | 2 | GH203544 | Solyc11g011920.1.1 evidence_code:10F1H1E1IEG genomic_reference:SL2.40ch11 gene_region:4848830-4855228 transcript_region:SL2.40ch11:4848830..4855228- go_terms:GO:0006536,GO:0030170,GO:0019752 functional_description:"Glutamate decarboxylase (AHRD V1 ***- Q8LKR4_TOBAC); contains Interpro domain(s) IPR010107 Glutamate decarboxylase " | 1E-70 |
| 326 | Contig 326 | 485 | 2 | GH204938 | Solyc08g005470.2.1 genomic_reference:SL2.40ch08 gene_region:347999-354065 transcript_region:SL2.40ch08:347999..354065- go_terms:GO:0005524 functional_description:"Cell division protein kinase 7 (AHRD V1 ***- CDK7_HUMAN); contains Interpro domain(s) IPR002290 Serine/threonine protein kinase " | 4E-58 |
| 327 | Contig 327 | 824 | 2 | GH203703 | Solyc04g076850.2.1 genomic_reference:SL2.40ch04 gene_region:59354651-59358834 transcript_region:SL2.40ch04:59354651..59358834+ go_terms:GO:0003700 functional_description:"Auxin responsive protein (AHRD V1 *-*- D9IQE6_CATRO); contains Interpro domain(s) IPR003311 AUX/IAA protein " | 7E-99 |
| 328 | Contig 328 | 534 | 2 | GH205247 | Solyc12g099100.1.1 evidence_code:10F1H1E1IEG genomic_reference:SL2.40ch12 gene_region:64711180-64716181 transcript_region:SL2.40ch12:64711180..64716181+ go_terms:GO:0005737,GO:0055114 functional_description:"Dihydrolipoyl dehydrogenase (AHRD V1 ***- Q9FEN7_SOLTU); contains Interpro domain(s) IPR006258 Dihydrolipoamide dehydrogenase " | 2E-34 |
| 329 | Contig 329 | 520 | 2 | GH204306 | Solyc03g007810.2.1 genomic_reference:SL2.40ch03 gene_region:2363561-2372504 transcript_region:SL2.40ch03:2363561..2372504- go_terms:GO:0006096,GO:0003824 functional_description:"Pyruvate kinase (AHRD V1 ***- A5BTB0_VITVI); contains Interpro domain(s) IPR001697 Pyruvate kinase " | 1E-29 |
| 330 | Contig 330 | 718 | 2 | GH204761 | Solyc01g101060.2.1 genomic_reference:SL2.40ch01 gene_region:82678819-82681422 transcript_region:SL2.40ch01:82678819..82681422+ go_terms:GO:0006730 functional_description:"S-adenosylmethionine synthase (AHRD V1 ***- B8LFH4_IPOBA); contains Interpro domain(s) IPR002133 S-adenosylmethionine synthetase " | 2E-86 |
| 331 | Contig 331 | 467 | 2 | GH203712 | Solyc07g041490.1.1 evidence_code:10F0H1E1IEG genomic_reference:SL2.40ch07 gene_region:50632738-50633541 transcript_region:SL2.40ch07:50632738..50633541+ functional_description:"Stress responsive alpha-beta barrel domain protein (AHRD V1 *-*- C7QRA6_CYAP0); contains Interpro domain(s) IPR013097 Stress responsive alpha-beta barrel " | 1E-69 |
| 332 | Contig 332 | 703 | 2 | GH203704 | Solyc05g009370.2.1 genomic_reference:SL2.40ch05 gene_region:3518447-3524365 transcript_region:SL2.40ch05:3518447..3524365- go_terms:GO:0005840,GO:0015934 functional_description:"50S ribosomal protein L15 (AHRD V1 *-*- B4B3P1_9CHRO); contains Interpro domain(s) IPR005749 Ribosomal protein L15, bacterial-type " | 1E-82 |
| 333 | Contig 333 | 497 | 2 | GH203669 | Solyc09g063070.2.1 genomic_reference:SL2.40ch09 gene_region:56657761-56663158 transcript_region:SL2.40ch09:56657761..56663158+ functional_description:"Senescence-associated protein-like (ISS) (AHRD V1 ***- Q00X72_OSTTA)" | 3E-75 |
| 334 | Contig 334 | 472 | 2 | GH203711 | Solyc04g008710.2.1 genomic_reference:SL2.40ch04 gene_region:2378787-2381066 transcript_region:SL2.40ch04:2378787..2381066- functional_description:"Unknown Protein (AHRD V1)" | 2E-44 |
| 335 | Contig 335 | 476 | 2 | GH205133 | Solyc10g047650.1.1 evidence_code:10F1H0E1IEG genomic_reference:SL2.40ch10 gene_region:37017583-37019644 transcript_region:SL2.40ch10:37017583..37019644+ go_terms:GO:0005515 functional_description:"p8MTCP1 (AHRD V1 ***- B6TKB9_MAIZE); contains Interpro domain(s) IPR010625 CHCH " | 2E-29 |
| 336 | Contig 336 | 453 | 2 | GH205066 | Solyc06g076880.2.1 genomic_reference:SL2.40ch06 gene_region:44165771-44168095 transcript_region:SL2.40ch06:44165771..44168095+ go_terms:GO:0042802 functional_description:"Flavoprotein wrbA (AHRD V1 ***- B6T4R2_MAIZE); contains Interpro domain(s) IPR010089 Flavoprotein WrbA " | 1E-63 |
| 337 | Contig 337 | 455 | 2 | GH203967 | Solyc03g082850.2.1 genomic_reference:SL2.40ch03 gene_region:46244596-46250538 transcript_region:SL2.40ch03:46244596..46250538- go_terms:GO:0003676,GO:0005622 functional_description:"3&apos-5&apos exonuclease family protein (AHRD V1 *--- Q23B12_TETTH); contains Interpro domain(s) IPR002782 Protein of unknown function DUF82 " | 5E-60 |
| 338 | Contig 338 | 445 | 2 | GH203878 | Solyc01g066840.2.1 genomic_reference:SL2.40ch01 gene_region:67445196-67447049 transcript_region:SL2.40ch01:67445196..67447049+ go_terms:GO:0003735 functional_description:"40S ribosomal protein S21 (AHRD V1 ***- D7M5J1_ARALY); contains Interpro domain(s) IPR001931 Ribosomal protein S21e " | 2E-43 |
| 339 | Contig 339 | 684 | 2 | GH204984 | Solyc01g096450.2.1 genomic_reference:SL2.40ch01 gene_region:79304614-79307810 transcript_region:SL2.40ch01:79304614..79307810+ go_terms:GO:0006508,GO:0031177 functional_description:"Aspartic proteinase nepenthesin-1 (AHRD V1 **-- B6TDX6_MAIZE); contains Interpro domain(s) IPR001461 Peptidase A1 " | 1E-117 |
| 340 | Contig 340 | 796 | 2 | GH204939 | Solyc07g055230.2.1 genomic_reference:SL2.40ch07 gene_region:60660818-60663114 transcript_region:SL2.40ch07:60660818..60663114+ go_terms:GO:0003735 functional_description:"50S ribosomal protein L5 (AHRD V1 ***- RL5_XANP2); contains Interpro domain(s) IPR002132 Ribosomal protein L5 " | 4E-47 |
| 341 | Contig 341 | 434 | 2 | GH204883 | Solyc02g069460.2.1 genomic_reference:SL2.40ch02 gene_region:33917839-33919502 transcript_region:SL2.40ch02:33917839..33919502- go_terms:GO:0009538 functional_description:"Photosystem I reaction center subunit III (AHRD V1 ***- Q9XQB4_PHAAU); contains Interpro domain(s) IPR003666 Photosystem I reaction centre protein PsaF, subunit III " | 4E-59 |
| 342 | Contig 342 | 434 | 2 | GH203952 | Solyc10g007150.2.1 genomic_reference:SL2.40ch10 gene_region:1553509-1565649 transcript_region:SL2.40ch10:1553509..1565649+ go_terms:GO:0017134 functional_description:"Apoptosis inhibitor (AHRD V1 ***- B0WCI8_CULQU); contains Interpro domain(s) IPR008383 Apoptosis inhibitory 5 " | 4E-69 |
| 343 | Contig 343 | 432 | 2 | GH203688 | Solyc01g005250.2.1 genomic_reference:SL2.40ch01 gene_region:191856-195078 transcript_region:SL2.40ch01:191856..195078- go_terms:GO:0008152,GO:0005737,GO:0055114 functional_description:"Aspartate-semialdehyde dehydrogenase (AHRD V1 ***- B6TWW1_MAIZE); contains Interpro domain(s) IPR005986 Aspartate-semialdehyde dehydrogenase, bacterial " | 7E-74 |
| 344 | Contig 344 | 429 | 2 | GH205355 | Solyc02g093680.2.1 genomic_reference:SL2.40ch02 gene_region:49014535-49017784 transcript_region:SL2.40ch02:49014535..49017784+ go_terms:GO:0000104,GO:0008270 functional_description:"Succinate dehydrogenase iron-sulfur protein (AHRD V1 **** C0NQC7_AJECG); contains Interpro domain(s) IPR004489 Succinate dehydrogenase/fumarate reductase iron-sulphur protein " | 2E-79 |
| 345 | Contig 345 | 468 | 2 | GH205019 | Solyc02g087190.1.1 evidence_code:10F0H1E1IEG genomic_reference:SL2.40ch02 gene_region:44243351-44244337 transcript_region:SL2.40ch02:44243351..44244337+ go_terms:GO:0004601,GO:0005515 functional_description:"Peroxidase 65 (AHRD V1 **** B6TYF5_MAIZE); contains Interpro domain(s) IPR002016 Haem peroxidase, plant/fungal/bacterial " | 2E-82 |
| 346 | Contig 346 | 455 | 2 | GH203899 | Solyc11g006540.1.1 evidence_code:10F1H1E1IEG genomic_reference:SL2.40ch11 gene_region:1191915-1194459 transcript_region:SL2.40ch11:1191915..1194459- go_terms:GO:0055114 functional_description:"FAD-dependent pyridine nucleotide-disulphide oxidoreductase (AHRD V1 **-- D3M425_9ACTO); contains Interpro domain(s) IPR001327 Pyridine nucleotide-disulphide oxidoreductase, NAD-binding region " | 5E-72 |
| 347 | Contig 347 | 530 | 2 | GH203753 | ********* |  |
| 348 | Contig 348 | 422 | 2 | GH204495 | Solyc06g083440.2.1 genomic_reference:SL2.40ch06 gene_region:45134673-45136744 transcript_region:SL2.40ch06:45134673..45136744+ go_terms:GO:0045153 functional_description:"Cytochrome b5 reductase (AHRD V1 *-** Q54XV1_DICDI); contains Interpro domain(s) IPR001199 Cytochrome b5 " | 7E-29 |
| 349 | Contig 349 | 415 | 2 | GH204117 | Solyc01g010750.2.1 genomic_reference:SL2.40ch01 gene_region:5783031-5788195 transcript_region:SL2.40ch01:5783031..5788195- functional_description:"Stress responsive protein (AHRD V1 *--- B6STP1_MAIZE); contains Interpro domain(s) IPR008997 Ricin B-related lectin " | 5E-64 |
| 350 | Contig 350 | 410 | 2 | GH205292 | Solyc04g076990.2.1 genomic_reference:SL2.40ch04 gene_region:59540573-59542894 transcript_region:SL2.40ch04:59540573..59542894+ go_terms:GO:0004675 functional_description:"Receptor like kinase, RLK" | 2E-65 |
| 351 | Contig 351 | 452 | 2 | GH204648 | Solyc03g095730.2.1 genomic_reference:SL2.40ch03 gene_region:50349408-50352048 transcript_region:SL2.40ch03:50349408..50352048- functional_description:"Ubiquinol-cytochrome c reductase complex protein (AHRD V1 ***- B6T3C5_MAIZE)" | 3E-14 |
| 352 | Contig 352 | 438 | 2 | GH204333 | Solyc01g104590.2.1 genomic_reference:SL2.40ch01 gene_region:84802263-84805701 transcript_region:SL2.40ch01:84802263..84805701- go_terms:GO:0003735,GO:0005515 functional_description:"Ribosomal protein L3 (AHRD V1 ***- Q6SKP4_SOLLC); contains Interpro domain(s) IPR019926 Ribosomal protein L3, conserved site IPR000597 Ribosomal protein L3 " | 6E-81 |
| 353 | Contig 353 | 420 | 2 | GH203800 | Solyc06g065390.2.1 genomic_reference:SL2.40ch06 gene_region:37196707-37200050 transcript_region:SL2.40ch06:37196707..37200050+ go_terms:GO:0005840 functional_description:"50S ribosomal protein L21 (AHRD V1 *-*- B6U3Z0_MAIZE); contains Interpro domain(s) IPR001787 Ribosomal protein L21 " | 5E-17 |
| 354 | Contig 354 | 572 | 2 | GH203549 | Solyc04g076190.1.1 evidence_code:10F0H1E1IEG genomic_reference:SL2.40ch04 gene_region:58719018-58720325 transcript_region:SL2.40ch04:58719018..58720325- go_terms:GO:0006508 functional_description:"Aspartic proteinase nepenthesin-1 (AHRD V1 **-- B6TDX6_MAIZE); contains Interpro domain(s) IPR001461 Peptidase A1 " | 5E-40 |
| 355 | Contig 355 | 491 | 2 | GH204991 | Solyc06g064810.2.1 genomic_reference:SL2.40ch06 gene_region:36771327-36775760 transcript_region:SL2.40ch06:36771327..36775760+ functional_description:"Chromosome 06 contig 1 DNA sequence (AHRD V1 ***- Q016E2_OSTTA)" | 1E-72 |
| 356 | Contig 356 | 396 | 2 | GH203524 | Solyc09g082780.2.1 genomic_reference:SL2.40ch09 gene_region:63852368-63856111 transcript_region:SL2.40ch09:63852368..63856111+ functional_description:"Asparagine synthetase B (AHRD V1 *-*- A1JQ99_YERE8)" | 6E-18 |
| 357 | Contig 357 | 464 | 2 | GH204385 | Solyc10g083260.1.1 evidence_code:10F1H0E1IEG genomic_reference:SL2.40ch10 gene_region:62423852-62424076 transcript_region:SL2.40ch10:62423852..62424076- functional_description:"Arabidopsis thaliana genomic DNA chromosome 5 P1 clone MOK16 (AHRD V1 **-- Q9LYW2_ARATH); contains Interpro domain(s) IPR007608 Protein of unknown function DUF584 " | 4E-19 |
| 358 | Contig 358 | 407 | 2 | GH204597 | Solyc09g010400.2.1 genomic_reference:SL2.40ch09 gene_region:3777857-3778870 transcript_region:SL2.40ch09:3777857..3778870+ go_terms:GO:0003684 functional_description:"Histone H2A (AHRD V1 ***- B9T4D3_RICCO); contains Interpro domain(s) IPR002119 Histone H2A " | 6E-42 |
| 359 | Contig 359 | 429 | 2 | GH204458 | Solyc08g006320.2.1 genomic_reference:SL2.40ch08 gene_region:985013-986480 transcript_region:SL2.40ch08:985013..986480- go_terms:GO:0005516 functional_description:"WRKY transcription factor 3 (AHRD V1 ***- A7UGD0_SOLTU); contains Interpro domain(s) IPR003657 DNA-binding WRKY " | 2E-42 |
| 360 | Contig 360 | 403 | 2 | GH203475 | Solyc03g058190.2.1 genomic_reference:SL2.40ch03 gene_region:20504510-20518591 transcript_region:SL2.40ch03:20504510..20518591+ go_terms:GO:0005515,GO:0003746 functional_description:"Elongation factor family GTP-binding protein (AHRD V1 **** Q67QM4_SYMTH); contains Interpro domain(s) IPR006298 GTP-binding protein TypA " | 8E-72 |
| 361 | Contig 361 | 385 | 2 | GH204556 | Solyc06g009290.2.1 genomic_reference:SL2.40ch06 gene_region:3249411-3257702 transcript_region:SL2.40ch06:3249411..3257702- go_terms:GO:0010329,GO:0008559 functional_description:"Lipid A export ATP-binding/permease protein msbA (AHRD V1 *--- MSBA_SYNAS); contains Interpro domain(s) IPR003439 ABC transporter-like " | 5E-24 |
| 362 | Contig 362 | 587 | 2 | GH204101 | Solyc08g075360.1.1 evidence_code:10F0H1E0IEG genomic_reference:SL2.40ch08 gene_region:56666411-56671614 transcript_region:SL2.40ch08:56666411..56671614- go_terms:GO:0008428 functional_description:"Zinc import ATP-binding protein ZnuC (AHRD V1 *-*- ZNUC_RICFE); contains Interpro domain(s) IPR013283 ABC transporter, ABCE " | 7E-73 |
| 363 | Contig 363 | 409 | 2 | GH203953 | Solyc05g047390.1.1 evidence_code:10F0H0E1IEG genomic_reference:SL2.40ch05 gene_region:57979251-57979487 transcript_region:SL2.40ch05:57979251..57979487+ go_terms:GO:0005741 functional_description:"Mitochondrial import receptor subunit TOM7-1 (AHRD V1 *-*- B6SJR3_MAIZE); contains Interpro domain(s) IPR012621 Mitochondrial outer membrane translocase complex, subunitt Tom7 " | 5E-11 |
| 364 | Contig 364 | 375 | 2 | GH204172 | Solyc11g069000.1.1 evidence_code:10F1H1E1IEG genomic_reference:SL2.40ch11 gene_region:50652125-50657452 transcript_region:SL2.40ch11:50652125..50657452- go_terms:GO:0044267,GO:0006457 functional_description:"T-complex protein 1 subunit beta (AHRD V1 ***- B6T8X5_MAIZE); contains Interpro domain(s) IPR012716 T-complex protein 1, beta subunit " | 5E-42 |
| 365 | Contig 365 | 652 | 2 | GH203476 | Solyc08g082690.2.1 genomic_reference:SL2.40ch08 gene_region:62557534-62560345 transcript_region:SL2.40ch08:62557534..62560345- go_terms:GO:0005840 functional_description:"30S ribosomal protein S6 (AHRD V1 *-*- D7DYF3_NOSA0); contains Interpro domain(s) IPR000529 Ribosomal protein S6 " | 3E-79 |
| 366 | Contig 366 | 392 | 2 | GH203588 | Solyc01g059980.2.1 genomic_reference:SL2.40ch01 gene_region:62201815-62226187 transcript_region:SL2.40ch01:62201815..62226187+ go_terms:GO:0005975 functional_description:"Beta-glucanase (AHRD V1 ***- Q53X08_NICPL); contains Interpro domain(s) IPR000490 Glycoside hydrolase, family 17 " | 2E-72 |
| 367 | Contig 367 | 370 | 2 | GH204257 | Solyc05g009790.1.1 evidence_code:10F0H1E1IEG genomic_reference:SL2.40ch05 gene_region:3987084-3988271 transcript_region:SL2.40ch05:3987084..3988271- go_terms:GO:0003677,GO:0016564 functional_description:"Transcription factor (AHRD V1 *-** D6MKM9_9ASPA); contains Interpro domain(s) IPR003340 Transcriptional factor B3 " | 1E-59 |
| 368 | Contig 368 | 881 | 2 | GH203624 | Solyc12g005340.1.1 evidence_code:10F1H1E1IEG genomic_reference:SL2.40ch12 gene_region:211757-213433 transcript_region:SL2.40ch12:211757..213433- functional_description:"GRAS family transcription factor (AHRD V1 **-- B9I7E1_POPTR); contains Interpro domain(s) IPR005202 GRAS transcription factor " | 1E-127 |
| 369 | Contig 369 | 365 | 2 | GH205240 | Solyc01g091650.2.1 genomic_reference:SL2.40ch01 gene_region:77004990-77009999 transcript_region:SL2.40ch01:77004990..77009999- go_terms:GO:0031995 functional_description:"COP9 signalosome complex subunit 1 (AHRD V1 ***- B6THB6_MAIZE); contains Interpro domain(s) IPR019585 26S proteasome, regulatory subunit Rpn7 " | 4E-65 |
| 370 | Contig 370 | 794 | 2 | GH203620 | Solyc06g054540.2.1 genomic_reference:SL2.40ch06 gene_region:33776420-33780993 transcript_region:SL2.40ch06:33776420..33780993- go_terms:GO:0005102 functional_description:"CHY zinc finger family protein expressed (AHRD V1 ***- Q2QP88_ORYSJ); contains Interpro domain(s) IPR008913 Zinc finger, CHY-type " | 1E-108 |
| 371 | Contig 371 | 396 | 2 | GH205174 | Solyc09g010630.2.1 genomic_reference:SL2.40ch09 gene_region:3965253-3968837 transcript_region:SL2.40ch09:3965253..3968837+ go_terms:GO:0051082 functional_description:"heat shock protein (AHRD V1 ***- B2D2G5_CAPSN); contains Interpro domain(s) IPR013126 Heat shock protein 70 " | 1E-70 |
| 372 | Contig 372 | 367 | 2 | GH203438 | Solyc03g119040.2.1 genomic_reference:SL2.40ch03 gene_region:61807988-61810035 transcript_region:SL2.40ch03:61807988..61810035- go_terms:GO:0005080,GO:0000166 functional_description:"Guanine nucleotide-binding protein beta subunit-like protein (AHRD V1 **-* B4FKM1_MAIZE); contains Interpro domain(s) IPR020472 G-protein beta WD-40 repeat, region " | 1E-68 |
| 373 | Contig 373 | 680 | 2 | GH204957 | Solyc07g040960.1.1 evidence_code:10F1H1E1IEG genomic_reference:SL2.40ch07 gene_region:48550320-48551819 transcript_region:SL2.40ch07:48550320..48551819+ go_terms:GO:0016042 functional_description:"Os07g0175100 protein (Fragment) (AHRD V1 *--- Q0D898_ORYSJ)" | 1E-126 |
| 374 | Contig 374 | 362 | 2 | GH204950 | Solyc04g081880.2.1 genomic_reference:SL2.40ch04 gene_region:63335224-63339615 transcript_region:SL2.40ch04:63335224..63339615- go_terms:GO:0003676 functional_description:"Ribonuclease P protein subunit p25 (AHRD V1 *--- RPP25_RAT); contains Interpro domain(s) IPR002775 Alba, DNA/RNA-binding protein " | 4E-32 |
| 375 | Contig 375 | 487 | 2 | GH204196 | Solyc01g105710.2.1 genomic_reference:SL2.40ch01 gene_region:85539000-85541482 transcript_region:SL2.40ch01:85539000..85541482- go_terms:GO:0006457 functional_description:"Peptidyl-prolyl cis-trans isomerase (AHRD V1 **-- A6RSK7_BOTFB); contains Interpro domain(s) IPR001179 Peptidyl-prolyl cis-trans isomerase, FKBP-type " | 1E-54 |
| 376 | Contig 376 | 604 | 2 | GH204853 | Solyc01g107820.2.1 genomic_reference:SL2.40ch01 gene_region:86993487-86995355 transcript_region:SL2.40ch01:86993487..86995355- go_terms:GO:0080044 functional_description:"UDP-glucosyltransferase family 1 protein (AHRD V1 **** C6KI43_CITSI); contains Interpro domain(s) IPR002213 UDP-glucuronosyl/UDP-glucosyltransferase " | 1E-116 |
| 377 | Contig 377 | 319 | 2 | GH204064 | Solyc08g078240.2.1 genomic_reference:SL2.40ch08 gene_region:59236252-59238091 transcript_region:SL2.40ch08:59236252..59238091- functional_description:"Unknown Protein (AHRD V1)" | 5E-55 |
| 378 | Contig 378 | 348 | 2 | GH204004 | Solyc09g075710.1.1 evidence_code:10F0H1E1IEG genomic_reference:SL2.40ch09 gene_region:62830815-62831798 transcript_region:SL2.40ch09:62830815..62831798- go_terms:GO:0004091 functional_description:"Gibberellin receptor GID1L2 (AHRD V1 **-- B6TRC0_MAIZE); contains Interpro domain(s) IPR013094 Alpha/beta hydrolase fold-3 " | 1E-50 |
| 379 | Contig 379 | 316 | 2 | GH204478 | Solyc10g086560.1.1 evidence_code:10F1H0E1IEG genomic_reference:SL2.40ch10 gene_region:64674861-64677116 transcript_region:SL2.40ch10:64674861..64677116- functional_description:"Unknown Protein (AHRD V1)" | 7E-57 |
| 380 | Contig 380 | 325 | 2 | GH204453 | Solyc06g083620.2.1 genomic_reference:SL2.40ch06 gene_region:45258804-45262774 transcript_region:SL2.40ch06:45258804..45262774- go_terms:GO:0005524,GO:0017111,GO:0005737 functional_description:"26S protease regulatory subunit 4 (AHRD V1 ***- B6T2S0_MAIZE); contains Interpro domain(s) IPR005937 26S proteasome subunit P45 " | 1E-56 |
| 381 | Contig 381 | 428 | 2 | GH204016 | Solyc02g081430.2.1 genomic_reference:SL2.40ch02 gene_region:39982861-39984643 transcript_region:SL2.40ch02:39982861..39984643+ go_terms:GO:0004601,GO:0004364 functional_description:"Microsomal glutathione S-transferase 3 (AHRD V1 **** B4FLH3_MAIZE); contains Interpro domain(s) IPR001129 Membrane-associated, eicosanoid and glutathione metabolism (MAPEG) " | 2E-57 |
| 382 | Contig 382 | 486 | 2 | GH203559 | ********* |  |
| 383 | Contig 383 | 776 | 2 | GH204317 | Solyc10g052880.1.1 evidence_code:10F0H1E1IEG genomic_reference:SL2.40ch10 gene_region:49565421-49566857 transcript_region:SL2.40ch10:49565421..49566857+ go_terms:GO:0004675 functional_description:"LRR receptor-like serine/threonine-protein kinase, RLP" | 1E-124 |
| 384 | Contig 384 | 315 | 2 | GH203990 | Solyc02g080640.2.1 genomic_reference:SL2.40ch02 gene_region:39406300-39409809 transcript_region:SL2.40ch02:39406300..39409809+ go_terms:GO:0009973 functional_description:"Phosphoadenosine phosphosulfate reductase (AHRD V1 *-** B7I7M3_ACIB5); contains Interpro domain(s) IPR013766 Thioredoxin domain " | 2E-54 |
| 385 | Contig 385 | 363 | 2 | GH204581 | Solyc06g082090.2.1 genomic_reference:SL2.40ch06 gene_region:44317047-44320702 transcript_region:SL2.40ch06:44317047..44320702+ go_terms:GO:0003700 functional_description:"Methionine aminopeptidase (AHRD V1 **-- C5FF46_NANOT); contains Interpro domain(s) IPR004545 Proliferation-associated protein 1 " | 2E-22 |
| 386 | Contig 386 | 333 | 2 | GH204145 | Solyc06g082830.2.1 genomic_reference:SL2.40ch06 gene_region:44784361-44787512 transcript_region:SL2.40ch06:44784361..44787512- functional_description:"Iron-stress related protein (AHRD V1 ***- B4FBU5_MAIZE)" | 5E-44 |
| 387 | Contig 387 | 657 | 2 | GH204587 | Solyc07g005760.2.1 genomic_reference:SL2.40ch07 gene_region:607977-611742 transcript_region:SL2.40ch07:607977..611742- go_terms:GO:0047205,GO:0047172 functional_description:"Hydroxycinnamoyl CoA shikimate/quinate hydroxycinnamoyltransferase (AHRD V1 **-* B2Z6Q6_POPTR); contains Interpro domain(s) IPR003480 Transferase " | 1E-98 |
| 388 | Contig 388 | 581 | 1 | GH203976 | Solyc01g060020.2.1 genomic_reference:SL2.40ch01 gene_region:62224181-62226272 transcript_region:SL2.40ch01:62224181..62226272- go_terms:GO:0005975 functional_description:"Beta-glucanase (AHRD V1 ***- Q53X08_NICPL); contains Interpro domain(s) IPR000490 Glycoside hydrolase, family 17 " | 3E-44 |
| 389 | Contig 389 | 577 | 1 | GH205356 | Solyc01g059980.2.1 genomic_reference:SL2.40ch01 gene_region:62201815-62226187 transcript_region:SL2.40ch01:62201815..62226187+ go_terms:GO:0005975 functional_description:"Beta-glucanase (AHRD V1 ***- Q53X08_NICPL); contains Interpro domain(s) IPR000490 Glycoside hydrolase, family 17 " | 1E-64 |
| 390 | Contig 390 | 596 | 2 | GH204937 | Solyc06g007440.2.1 genomic_reference:SL2.40ch06 gene_region:1453365-1455690 transcript_region:SL2.40ch06:1453365..1455690- go_terms:GO:0004679 functional_description:"CBL-interacting protein kinase 16 (AHRD V1 **** A0MNK3_POPTR); contains Interpro domain(s) IPR002290 Serine/threonine protein kinase " | 9E-38 |
| 391 | Contig 391 | 411 | 2 | GH204166 | Solyc11g068810.1.1 evidence_code:10F1H0E1IEG genomic_reference:SL2.40ch11 gene_region:50510078-50510311 transcript_region:SL2.40ch11:50510078..50510311- functional_description:"Unknown Protein (AHRD V1)" | 1E-39 |
| 392 | Contig 392 | 273 | 2 | GH205075 | Solyc06g051960.2.1 genomic_reference:SL2.40ch06 gene_region:32128328-32131813 transcript_region:SL2.40ch06:32128328..32131813+ go_terms:GO:0005618,GO:0030599 functional_description:"Pectinesterase (AHRD V1 ***- B9IDR8_POPTR); contains Interpro domain(s) IPR000070 Pectinesterase, catalytic " | 9E-51 |
| 393 | Contig 393 | 456 | 2 | GH203812 | Solyc10g081100.1.1 evidence_code:10F1H0E1IEG genomic_reference:SL2.40ch10 gene_region:61573572-61575473 transcript_region:SL2.40ch10:61573572..61575473+ functional_description:"Unknown Protein (AHRD V1)" | 2E-26 |
| 394 | Contig 394 | 283 | 2 | GH203865 | Solyc11g070060.1.1 evidence_code:10F0H1E0IEG genomic_reference:SL2.40ch11 gene_region:51693900-51699534 transcript_region:SL2.40ch11:51693900..51699534- functional_description:"DUF866 domain protein (AHRD V1 **-- A1C7N7_ASPCL); contains Interpro domain(s) IPR008584 Protein of unknown function DUF866, eukaryotic " | 2E-41 |
| 395 | Contig 395 | 626 | 2 | GH204030 | Solyc01g106280.2.1 genomic_reference:SL2.40ch01 gene_region:85985054-85987923 transcript_region:SL2.40ch01:85985054..85987923- go_terms:GO:0003677 functional_description:"DNA-binding bromodomain-containing protein (AHRD V1 *--* D7KG11_ARALY); contains Interpro domain(s) IPR001487 Bromodomain " | 2E-21 |
| 396 | Contig 396 | 265 | 2 | GH204036 | Solyc03g093560.1.1 evidence_code:10F0H1E1IEG genomic_reference:SL2.40ch03 gene_region:48392056-48392790 transcript_region:SL2.40ch03:48392056..48392790- go_terms:GO:0005515,GO:0003677 functional_description:"Ethylene-responsive transcription factor 2 (AHRD V1 ***- B6U860_MAIZE); contains Interpro domain(s) IPR001471 Pathogenesis-related transcriptional factor and ERF, DNA-binding " | 4E-16 |
| 397 | Contig 397 | 498 | 2 | GH204093 | Solyc12g006320.1.1 evidence_code:10F0H1E1IEG genomic_reference:SL2.40ch12 gene_region:841803-847655 transcript_region:SL2.40ch12:841803..847655- go_terms:GO:0008026,GO:0005524 functional_description:"ATP-dependent RNA helicase (AHRD V1 *-*- Q6L724_HORVU); contains Interpro domain(s) IPR011545 DNA/RNA helicase, DEAD/DEAH box type, N-terminal " | 2E-81 |
| 398 | Contig 398 | 315 | 2 | GH203558 | Solyc09g076040.2.1 genomic_reference:SL2.40ch09 gene_region:63216517-63225104 transcript_region:SL2.40ch09:63216517..63225104+ functional_description:"Protein SUPPRESSOR OF GENE SILENCING 3 homolog (AHRD V1 *--- SGS3_ORYSJ); contains Interpro domain(s) IPR005380 Region of unknown function XS " | 8E-29 |
| 399 | Contig 399 | 261 | 2 | GH203752 | Solyc02g063070.2.1 genomic_reference:SL2.40ch02 gene_region:29692418-29699297 transcript_region:SL2.40ch02:29692418..29699297- go_terms:GO:0050815 functional_description:"14-3-3 protein beta/alpha-1 (AHRD V1 **-- 143B1_DANRE); contains Interpro domain(s) IPR000308 14-3-3 protein " | 1E-43 |
| 400 | Contig 400 | 530 | 2 | GH204434 | Solyc11g071490.1.1 evidence_code:10F0H1E1IEG genomic_reference:SL2.40ch11 gene_region:52029448-52031919 transcript_region:SL2.40ch11:52029448..52031919+ go_terms:GO:0005840 functional_description:"Ribosomal protein L30 (AHRD V1 ***- B3TLP4_ELAGV); contains Interpro domain(s) IPR000231 Ribosomal protein L30e " | 6E-59 |
| 401 | Contig 401 | 365 | 2 | GH203497 | ********* |  |
| 402 | Contig 402 | 979 | 2 | GH203638 | Solyc01g009180.2.1 genomic_reference:SL2.40ch01 gene_region:3229107-3234908 transcript_region:SL2.40ch01:3229107..3234908+ go_terms:GO:0008705 functional_description:"5- methyltetrahydropteroyltriglutamate--homocysteine methyltransferase (AHRD V1 ***- B6UF55_MAIZE); contains Interpro domain(s) IPR006276 5-methyltetrahydropteroyltriglutamate--homocysteine S-methyltransferase " | 1E-139 |
| 403 | Contig 403 | 261 | 2 | GH203435 | ********* |  |
| 404 | Contig 404 | 265 | 2 | GH204215 | Solyc03g120320.1.1 evidence_code:10F0H1E1IEG genomic_reference:SL2.40ch03 gene_region:62801895-62802962 transcript_region:SL2.40ch03:62801895..62802962- functional_description:"Kelch-like protein (AHRD V1 *-*- C4Q722_SCHMA); contains Interpro domain(s) IPR015915 Kelch-type beta propeller " | 1E-40 |
| 405 | Contig 405 | 289 | 2 | GH203425 | Solyc03g083560.1.1 evidence_code:10F0H1E1IEG genomic_reference:SL2.40ch03 gene_region:47058250-47059299 transcript_region:SL2.40ch03:47058250..47059299+ functional_description:"Expressed protein (Fragment) (AHRD V1 ***- B3VJP0_POPTN); contains Interpro domain(s) IPR006766 Phosphate-induced protein 1 conserved region " | 8E-26 |
| 406 | Contig 406 | 239 | 2 | GH204958 | Solyc12g099970.1.1 evidence_code:10F0H1E1IEG genomic_reference:SL2.40ch12 gene_region:65242252-65245386 transcript_region:SL2.40ch12:65242252..65245386+ go_terms:GO:0019901 functional_description:"5-amp-activated protein kinase beta subunit (AHRD V1 **** Q17IU7_AEDAE); contains Interpro domain(s) IPR006828 5-AMP-activated protein kinase, beta subunit, complex-interacting region " | 7E-17 |
| 407 | Contig 407 | 551 | 2 | GH205396 | Solyc08g077440.2.1 genomic_reference:SL2.40ch08 gene_region:58492655-58502566 transcript_region:SL2.40ch08:58492655..58502566- go_terms:GO:0004176,GO:0070182 functional_description:"ATP-dependent protease La (AHRD V1 **** A7HC55_ANADF); contains Interpro domain(s) IPR004815 Peptidase S16, ATP-dependent protease La " | 3E-83 |
| 408 | Contig 408 | 340 | 2 | GH204956 | ********* |  |
| 409 | Contig 409 | 232 | 2 | GH203555 | ********* |  |
| 410 | Contig 410 | 276 | 2 | GH204809 | Solyc02g092430.1.1 evidence_code:10F1H1E1IEG genomic_reference:SL2.40ch02 gene_region:48070364-48070804 transcript_region:SL2.40ch02:48070364..48070804+ go_terms:GO:0003735,GO:0003723 functional_description:"Ribosomal protein L26 (AHRD V1 ***- A8HMG7_CHLRE); contains Interpro domain(s) IPR005756 Ribosomal protein L26, eukaryotic/archaeal " | 2E-29 |
| 411 | Contig 411 | 231 | 2 | GH204654 | Solyc09g014780.2.1 genomic_reference:SL2.40ch09 gene_region:6917876-6935366 transcript_region:SL2.40ch09:6917876..6935366+ go_terms:GO:0032403 functional_description:"WD-repeat protein mip1 (AHRD V1 *--- C5FLG0_NANOT); contains Interpro domain(s) IPR004083 Regulatory associated protein of TOR " | 1E-38 |
| 412 | Contig 412 | 376 | 2 | GH205041 | Solyc12g006010.1.1 evidence_code:10F1H1E1IEG genomic_reference:SL2.40ch12 gene_region:619162-621481 transcript_region:SL2.40ch12:619162..621481+ go_terms:GO:0005737 functional_description:"Small ubiquitin-related modifier (AHRD V1 ***- A6BMG7_COPCI); contains Interpro domain(s) IPR000626 Ubiquitin " | 7E-20 |
| 413 | Contig 413 | 303 | 2 | GH204235 | Solyc12g008630.1.1 evidence_code:10F1H1E1IEG genomic_reference:SL2.40ch12 gene_region:2007849-2015328 transcript_region:SL2.40ch12:2007849..2015328- go_terms:GO:0006508,GO:0046872 functional_description:"Mitochondrial processing peptidase alpha subunit (AHRD V1 ***- Q948V5_MORAL); contains Interpro domain(s) IPR011237 Peptidase M16, core " | 5E-27 |
| 414 | Contig 414 | 421 | 2 | GH204788 | Solyc01g095150.2.1 genomic_reference:SL2.40ch01 gene_region:78285184-78287680 transcript_region:SL2.40ch01:78285184..78287680+ go_terms:GO:0009269 functional_description:"Late embryogenesis abundant protein (Fragment) (AHRD V1 **-- B7TGE2_PINSY); contains Interpro domain(s) IPR013990 Water Stress and Hypersensitive response " | 3E-64 |
| 415 | Contig 415 | 511 | 2 | GH204787 | Solyc10g078960.1.1 evidence_code:10F1H1E1IEG genomic_reference:SL2.40ch10 gene_region:59923974-59925902 transcript_region:SL2.40ch10:59923974..59925902- go_terms:GO:0005840 functional_description:"60S ribosomal protein L21-like protein (AHRD V1 ***- Q3HVK5_SOLTU); contains Interpro domain(s) IPR001147 Ribosomal protein L21e " | 2E-55 |
| 416 | Contig 416 | 639 | 2 | GH205177 | Solyc02g070890.2.1 genomic_reference:SL2.40ch02 gene_region:35066723-35070830 transcript_region:SL2.40ch02:35066723..35070830+ go_terms:GO:0004675 functional_description:"Receptor like kinase, RLK" | 1E-117 |
| 417 | Contig 417 | 217 | 2 | GH205149 | Solyc06g075780.1.1 evidence_code:10F1H1E1IEG genomic_reference:SL2.40ch06 gene_region:43485594-43486058 transcript_region:SL2.40ch06:43485594..43486058+ go_terms:GO:0003700 functional_description:"Cys2/His2 zinc-finger transcription factor (AHRD V1 **** Q4U318_SILLA); contains Interpro domain(s) IPR007087 Zinc finger, C2H2-type " | 3E-40 |
| 418 | Contig 418 | 362 | 2 | GH204696 | Solyc03g044330.1.1 evidence_code:10F0H1E1IEG genomic_reference:SL2.40ch03 gene_region:12823169-12825148 transcript_region:SL2.40ch03:12823169..12825148- go_terms:GO:0003984 functional_description:"Acetolactate synthase (AHRD V1 **** D7RPJ7_SOLTU); contains Interpro domain(s) IPR012846 Acetolactate synthase, large subunit, biosynthetic " | 2E-63 |
| 419 | Contig 419 | 213 | 2 | GH204521 | Solyc01g110870.2.1 genomic_reference:SL2.40ch01 gene_region:89139334-89139841 transcript_region:SL2.40ch01:89139334..89139841- go_terms:GO:0005516 functional_description:"Auxin-induced SAUR-like protein (AHRD V1 ***- Q8S351_CAPAN); contains Interpro domain(s) IPR003676 Auxin responsive SAUR protein " | 2E-17 |
| 420 | Contig 420 | 656 | 2 | GH203662 | Solyc12g100160.1.1 evidence_code:10F0H1E1IEG genomic_reference:SL2.40ch12 gene_region:65330773-65334942 transcript_region:SL2.40ch12:65330773..65334942+ go_terms:GO:0005840 functional_description:"50S ribosomal protein L6 (AHRD V1 ***- B6SL57_MAIZE); contains Interpro domain(s) IPR019906 Ribosomal protein L6, subgroup " | 4E-80 |
| 421 | Contig 421 | 387 | 2 | GH203936 | Solyc06g082940.2.1 genomic_reference:SL2.40ch06 gene_region:44850630-44851884 transcript_region:SL2.40ch06:44850630..44851884+ go_terms:GO:0009538 functional_description:"Photosystem I reaction center subunit XI (AHRD V1 ***- B6SHL1_MAIZE); contains Interpro domain(s) IPR003757 Photosystem I reaction centre, subunit XI PsaL " | 2E-37 |
| 422 | Contig 422 | 198 | 2 | GH205054 | Solyc01g086970.2.1 genomic_reference:SL2.40ch01 gene_region:73647298-73648116 transcript_region:SL2.40ch01:73647298..73648116- go_terms:GO:0005515 functional_description:"Zinc finger A20 and AN1 domain-containing stress-associated protein 6 (AHRD V1 ***- C1C2A2_9MAXI); contains Interpro domain(s) IPR000058 Zinc finger, AN1-type " | 1E-28 |
| 423 | Contig 423 | 678 | 2 | GH205371 | Solyc08g067430.2.1 genomic_reference:SL2.40ch08 gene_region:53686130-53690841 transcript_region:SL2.40ch08:53686130..53690841+ functional_description:"cDNA clone 002-182-C01 full insert sequence (AHRD V1 *-*- B7F3P2_ORYSJ)" | 6E-93 |
| 424 | Contig 424 | 196 | 2 | GH204891 | Solyc01g080280.2.1 genomic_reference:SL2.40ch01 gene_region:72039739-72045026 transcript_region:SL2.40ch01:72039739..72045026+ go_terms:GO:0004356 functional_description:"Glutamine synthetase (AHRD V1 ***- B5LAU9_CAPAN); contains Interpro domain(s) IPR008146 Glutamine synthetase, catalytic region " | 1E-35 |
| 425 | Contig 425 | 195 | 2 | GH203684 | ********* |  |
| 426 | Contig 426 | 353 | 2 | GH204763 | Solyc01g107870.2.1 genomic_reference:SL2.40ch01 gene_region:87021438-87028206 transcript_region:SL2.40ch01:87021438..87028206+ go_terms:GO:0008143 functional_description:"Poly(A) RNA binding protein (AHRD V1 **** B2CJ74_9HYPO); contains Interpro domain(s) IPR006515 Polyadenylate binding protein, human types 1, 2, 3, 4 " | 3E-48 |
| 427 | Contig 427 | 479 | 2 | GH203864 | Solyc03g019880.2.1 genomic_reference:SL2.40ch03 gene_region:6774310-6777201 transcript_region:SL2.40ch03:6774310..6777201+ functional_description:"UPF0426 protein At1g28150, chloroplastic (AHRD V1 ***- Y1815_ARATH)" | 1E-66 |
| 428 | Contig 428 | 790 | 2 | GH205184 | Solyc06g076360.2.1 genomic_reference:SL2.40ch06 gene_region:43831465-43835828 transcript_region:SL2.40ch06:43831465..43835828- go_terms:GO:0006886,GO:0019867 functional_description:"Outer envelope membrane protein (AHRD V1 **-- Q31PR1_SYNE7); contains Interpro domain(s) IPR005689 Chloroplast envelope protein translocase, IAP75 " | 1E-123 |
| 429 | Contig 429 | 177 | 2 | GH203527 | Solyc07g052980.2.1 genomic_reference:SL2.40ch07 gene_region:58724756-58726704 transcript_region:SL2.40ch07:58724756..58726704+ go_terms:GO:0016798 functional_description:"Xyloglucan endotransglucosylase/hydrolase 5 (AHRD V1 **** C0IRH8_MALDO); contains Interpro domain(s) IPR016455 Xyloglucan endotransglucosylase/hydrolase " | 1E-29 |
| 430 | Contig 430 | 173 | 2 | GH203582 | ********* |  |
| 431 | Contig 431 | 353 | 2 | GH203746 | Solyc10g086570.2.1 genomic_reference:SL2.40ch10 gene_region:64684179-64684888 transcript_region:SL2.40ch10:64684179..64684888- go_terms:GO:0008270 functional_description:"Palmitoyltransferase PFA4 (AHRD V1 **-- B6TA04_MAIZE); contains Interpro domain(s) IPR001594 Zinc finger, DHHC-type " | 5E-23 |
| 432 | Contig 432 | 633 | 2 | GH205196 | Solyc01g101140.2.1 genomic_reference:SL2.40ch01 gene_region:82748479-82753118 transcript_region:SL2.40ch01:82748479..82753118+ functional_description:"LOC100005105 protein (Fragment) (AHRD V1 *-*- A4QNR9_DANRE); contains Interpro domain(s) IPR007491 Protein of unknown function DUF537 " | 1E-100 |
| 433 | Contig 433 | 179 | 2 | GH204304 | Solyc12g056830.1.1 evidence_code:10F0H1E1IEG genomic_reference:SL2.40ch12 gene_region:48264644-48265396 transcript_region:SL2.40ch12:48264644..48265396+ go_terms:GO:0045261 functional_description:"ATP synthase delta subunit (AHRD V1 ***- Q7XYM8_BIGNA); contains Interpro domain(s) IPR000711 ATPase, F1 complex, OSCP/delta subunit " | 5E-11 |
| 434 | Contig 434 | 214 | 2 | GH204305 | ********* |  |
| 435 | Contig 435 | 234 | 2 | GH203832 | ********* |  |
| 436 | Contig 436 | 905 | 2 | GH203500 | Solyc11g071640.1.1 evidence_code:10F0H1E1IEG genomic_reference:SL2.40ch11 gene_region:52141513-52144602 transcript_region:SL2.40ch11:52141513..52144602+ go_terms:GO:0008810 functional_description:"Beta-D-glucosidase (AHRD V1 ***- O82074_TROMA); contains Interpro domain(s) IPR001764 Glycoside hydrolase, family 3, N-terminal " | 1E-167 |
| 437 | Contig 437 | 343 | 2 | GH205085 | Solyc06g007510.2.1 genomic_reference:SL2.40ch06 gene_region:1501286-1506397 transcript_region:SL2.40ch06:1501286..1506397- go_terms:GO:0004842 functional_description:"Ubiquitin-conjugating enzyme E2 8 (AHRD V1 **** UBC8_ARATH); contains Interpro domain(s) IPR000608 Ubiquitin-conjugating enzyme, E2 " | 6E-32 |
| 438 | Contig 438 | 421 | 2 | GH204562 | Solyc05g054620.2.1 genomic_reference:SL2.40ch05 gene_region:63640937-63645239 transcript_region:SL2.40ch05:63640937..63645239+ go_terms:GO:0008152 functional_description:"Amino acid binding protein (AHRD V1 **-- B6TDN9_MAIZE); contains Interpro domain(s) IPR002912 Amino acid-binding ACT " | 3E-79 |
| 439 | Contig 439 | 651 | 2 | GH204691 | ********* |  |
| 440 | Contig 440 | 182 | 2 | GH205015 | ********* |  |
| 441 | Contig 441 | 543 | 2 | GH204751 | Solyc06g066640.2.1 genomic_reference:SL2.40ch06 gene_region:38268035-38269441 transcript_region:SL2.40ch06:38268035..38269441+ go_terms:GO:0009538 functional_description:"Photosystem I reaction center subunit VI-1, chloroplastic (AHRD V1 ***- PSAH1_ARATH); contains Interpro domain(s) IPR004928 Photosystem I reaction centre subunit VI " | 4E-51 |
| 442 | Contig 442 | 144 | 2 | GH203517 | ********* |  |
| 443 | Contig 443 | 631 | 2 | GH205115 | Solyc05g008450.2.1 genomic_reference:SL2.40ch05 gene_region:2800495-2805716 transcript_region:SL2.40ch05:2800495..2805716- go_terms:GO:0005507 functional_description:"Oxidoreductase FAD/NAD(P)-binding domain protein (AHRD V1 **-* B8JDQ6_ANAD2); contains Interpro domain(s) IPR001433 Oxidoreductase FAD/NAD(P)-binding " | 7E-82 |
| 444 | Contig 444 | 169 | 2 | GH203538 | Solyc06g051960.2.1 genomic_reference:SL2.40ch06 gene_region:32128328-32131813 transcript_region:SL2.40ch06:32128328..32131813+ go_terms:GO:0005618,GO:0030599 functional_description:"Pectinesterase (AHRD V1 ***- B9IDR8_POPTR); contains Interpro domain(s) IPR000070 Pectinesterase, catalytic " | 4E-26 |
| 445 | Contig 445 | 670 | 2 | GH203678 | Solyc10g079420.1.1 evidence_code:10F0H1E1IEG genomic_reference:SL2.40ch10 gene_region:60282482-60283099 transcript_region:SL2.40ch10:60282482..60283099+ go_terms:GO:0005509 functional_description:"Calmodulin (AHRD V1 ***- B6TKX0_MAIZE); contains Interpro domain(s) IPR011992 EF-Hand type " | 1E-36 |
| 446 | Contig 446 | 224 | 2 | GH203636 | Solyc03g119040.2.1 genomic_reference:SL2.40ch03 gene_region:61807988-61810035 transcript_region:SL2.40ch03:61807988..61810035- go_terms:GO:0005080,GO:0000166 functional_description:"Guanine nucleotide-binding protein beta subunit-like protein (AHRD V1 **-* B4FKM1_MAIZE); contains Interpro domain(s) IPR020472 G-protein beta WD-40 repeat, region " | 5E-22 |
| 447 | Contig 447 | 122 | 2 | GH204849 | Solyc06g009210.2.1 genomic_reference:SL2.40ch06 gene_region:3150991-3153904 transcript_region:SL2.40ch06:3150991..3153904+ go_terms:GO:0003735 functional_description:"Ribosomal protein L19 (AHRD V1 ***- Q2PYW9_SOLTU); contains Interpro domain(s) IPR000196 Ribosomal protein L19/L19e " | 9E-11 |
| 448 | Contig 448 | 569 | 2 | GH204515 | ********* |  |
| 449 | Contig 449 | 1123 | 2 | GH203881 | Solyc05g050120.2.1 genomic_reference:SL2.40ch05 gene_region:59250938-59255250 transcript_region:SL2.40ch05:59250938..59255250+ go_terms:GO:0004473 functional_description:"Malic enzyme (AHRD V1 ***- O04936_SOLLC); contains Interpro domain(s) IPR012302 Malic enzyme, NAD-binding " | 1E-160 |
| 450 | Contig 450 | 556 | 2 | GH205182 | Solyc02g082000.2.1 genomic_reference:SL2.40ch02 gene_region:40322179-40324715 transcript_region:SL2.40ch02:40322179..40324715- go_terms:GO:0003735,GO:0005515 functional_description:"30S ribosomal protein S19 (AHRD V1 *-*- RS19_BRAHW); contains Interpro domain(s) IPR005713 Ribosomal protein S15, eukaryotic/archaeal " | 5E-83 |
| 451 | Contig 451 | 322 | 2 | GH205143 | Solyc03g006490.2.1 genomic_reference:SL2.40ch03 gene_region:1074627-1077380 transcript_region:SL2.40ch03:1074627..1077380+ functional_description:"Aluminum-induced protein-like (AHRD V1 ***- Q9FG81_ARATH)" | 2E-59 |
| 452 | Contig 452 | 600 | 2 | GH205285 | Solyc11g006880.1.1 evidence_code:10F1H0E1IEG genomic_reference:SL2.40ch11 gene_region:1417654-1420489 transcript_region:SL2.40ch11:1417654..1420489- functional_description:"Genomic DNA chromosome 5 TAC clone K17N15 (AHRD V1 **-- Q9FHN3_ARATH); contains Interpro domain(s) IPR009787 Protein of unknown function DUF1352 " | 7E-73 |
| 453 | Contig 453 | 133 | 2 | GH204653 | ********* |  |
| 454 | Contig 454 | 116 | 2 | GH205286 | ********* |  |
| 455 | Contig 455 | 288 | 2 | GH203509 | ********* |  |
| 456 | Contig 456 | 335 | 2 | GH203601 | Solyc06g035450.2.1 genomic_reference:SL2.40ch06 gene_region:21040469-21044581 transcript_region:SL2.40ch06:21040469..21044581+ go_terms:GO:0004004 functional_description:"ATP-dependent RNA helicase (AHRD V1 **** Q6L724_HORVU); contains Interpro domain(s) IPR011545 DNA/RNA helicase, DEAD/DEAH box type, N-terminal " | 2E-55 |
| 457 | Contig 457 | 429 | 1 | GH204327 | Solyc09g007920.2.1 genomic_reference:SL2.40ch09 gene_region:1435451-1440492 transcript_region:SL2.40ch09:1435451..1440492+ go_terms:GO:0004397,GO:0045548 functional_description:"Phenylalanine ammonia-lyase (AHRD V1 **** B5LAW0_CAPAN); contains Interpro domain(s) IPR005922 Phenylalanine ammonia-lyase " | 6E-54 |
| 458 | Contig 458 | 485 | 1 | GH204829 | Solyc09g007900.2.1 genomic_reference:SL2.40ch09 gene_region:1419041-1422226 transcript_region:SL2.40ch09:1419041..1422226- go_terms:GO:0004397,GO:0045548 functional_description:"Phenylalanine ammonia-lyase (AHRD V1 **** B5LAW0_CAPAN); contains Interpro domain(s) IPR005922 Phenylalanine ammonia-lyase " | 3E-70 |
| 459 | Contig 459 | 662 | 2 | GH205265 | Solyc10g054870.1.1 evidence_code:10F0H1E1IEG genomic_reference:SL2.40ch10 gene_region:51507074-51512787 transcript_region:SL2.40ch10:51507074..51512787- go_terms:GO:0008152 functional_description:"Triosephosphate isomerase (AHRD V1 ***- A9PH17_POPTR); contains Interpro domain(s) IPR000652 Triosephosphate isomerase " | 1E-103 |
| 460 | Contig 460 | 784 | 2 | GH203916 | Solyc12g013710.1.1 evidence_code:10F1H1E1IEG genomic_reference:SL2.40ch12 gene_region:4544984-4547459 transcript_region:SL2.40ch12:4544984..4547459- go_terms:GO:0004745 functional_description:"Protochlorophyllide reductase (AHRD V1 ***- Q8LAV9_ARATH); contains Interpro domain(s) IPR005979 Light-dependent protochlorophyllide reductase " | 2E-78 |
| 461 | Contig 461 | 347 | 1 | GH204503 | ********* |  |
| 462 | Contig 462 | 163 | 1 | GH204593 | Solyc11g062130.1.1 evidence_code:10F0H1E1IEG genomic_reference:SL2.40ch11 gene_region:46084210-46085851 transcript_region:SL2.40ch11:46084210..46085851+ go_terms:GO:0016020,GO:0005743 functional_description:"Mitochondrial ADP/ATP carrier proteins (AHRD V1 ***- Q2UU95_ASPOR); contains Interpro domain(s) IPR002113 Adenine nucleotide translocator 1 " | 3E-24 |
| 463 | Contig 463 | 600 | 1 | GH204365 | Solyc08g078860.2.1 genomic_reference:SL2.40ch08 gene_region:59718819-59724122 transcript_region:SL2.40ch08:59718819..59724122+ functional_description:"Homology to unknown gene (AHRD V1 ***- Q010L3_OSTTA)" | 1E-53 |
| 464 | Contig 464 | 489 | 1 | GH204150 | Solyc08g078860.2.1 genomic_reference:SL2.40ch08 gene_region:59718819-59724122 transcript_region:SL2.40ch08:59718819..59724122+ functional_description:"Homology to unknown gene (AHRD V1 ***- Q010L3_OSTTA)" | 2E-36 |
| 465 | Contig 465 | 365 | 2 | GH203489 | Solyc12g099080.1.1 evidence_code:10F0H1E1IEG genomic_reference:SL2.40ch12 gene_region:64696729-64701008 transcript_region:SL2.40ch12:64696729..64701008- go_terms:GO:0005525,GO:0005622 functional_description:"ADP ribosylation factor (AHRD V1 *-*- Q08IJ1_TOBAC); contains Interpro domain(s) IPR006688 ADP-ribosylation factor " | 4E-30 |
| 466 | Contig 466 | 780 | 1 | GH204537 | Solyc06g063000.2.1 genomic_reference:SL2.40ch06 gene_region:36157647-36163138 transcript_region:SL2.40ch06:36157647..36163138+ go_terms:GO:0005515 functional_description:"Polyadenylate-binding protein (AHRD V1 **** A8N215_COPC7); contains Interpro domain(s) IPR012677 Nucleotide-binding, alpha-beta plait " | 6E-72 |
| 467 | Contig 467 | 730 | 1 | GH204082 | Solyc10g047130.1.1 evidence_code:10F0H1E0IEG genomic_reference:SL2.40ch10 gene_region:35339896-35342026 transcript_region:SL2.40ch10:35339896..35342026+ go_terms:GO:0003690,GO:0043047 functional_description:"RNA-binding protein RZ-1 (AHRD V1 **** D0V0M9_CAPAN); contains Interpro domain(s) IPR015465 RNA recognition motif, glycine rich protein " | 2E-86 |
| 468 | Contig 468 | 682 | 1 | GH204676 | Solyc09g005720.2.1 genomic_reference:SL2.40ch09 gene_region:498274-500367 transcript_region:SL2.40ch09:498274..500367- go_terms:GO:0003735,GO:0019843 functional_description:"60S ribosomal protein L23a (AHRD V1 ***- B6TN30_MAIZE); contains Interpro domain(s) IPR001014 Ribosomal protein L23/L25, conserved site " | 2E-61 |
| 469 | Contig 469 | 381 | 1 | GH204813 | Solyc05g054580.2.1 genomic_reference:SL2.40ch05 gene_region:63611603-63614235 transcript_region:SL2.40ch05:63611603..63614235- go_terms:GO:0005515,GO:0005507 functional_description:"60S acidic ribosomal protein P0 (AHRD V1 ***- D7L9V8_ARALY); contains Interpro domain(s) IPR001790 Ribosomal protein L10 " | 7E-55 |
| 470 | Contig 470 | 712 | 2 | GH205178 | Solyc10g018300.1.1 evidence_code:10F0H1E1IEG genomic_reference:SL2.40ch10 gene_region:7264174-7267205 transcript_region:SL2.40ch10:7264174..7267205- go_terms:GO:0004802,GO:0005515 functional_description:"Transketolase 1 (AHRD V1 **** O78327_CAPAN); contains Interpro domain(s) IPR005478 Bacterial transketolase " | 1E-110 |
| 471 | Contig 471 | 709 | 1 | GH203718 | Solyc10g080710.1.1 evidence_code:10F1H1E1IEG genomic_reference:SL2.40ch10 gene_region:61247846-61251817 transcript_region:SL2.40ch10:61247846..61251817- go_terms:GO:0005515,GO:0004816 functional_description:"Asparaginyl-tRNA synthetase 2 (AHRD V1 **** D7L6K3_ARALY); contains Interpro domain(s) IPR004522 Asparaginyl-tRNA synthetase, class IIb " | 1E-106 |
| 472 | Contig 472 | 569 | 1 | GH204890 | Solyc02g083290.2.1 genomic_reference:SL2.40ch02 gene_region:41311201-41321957 transcript_region:SL2.40ch02:41311201..41321957- go_terms:GO:0004712 functional_description:"Serine/threonine/tyrosine kinase (AHRD V1 *-** Q9AWA6_ARAHY); contains Interpro domain(s) IPR015783 ATMRK serine/threonine protein kinase-like " | 1E-107 |
| 473 | Contig 473 | 190 | 1 | GH203658 | Solyc05g009790.1.1 evidence_code:10F0H1E1IEG genomic_reference:SL2.40ch05 gene_region:3987084-3988271 transcript_region:SL2.40ch05:3987084..3988271- go_terms:GO:0003677,GO:0016564 functional_description:"Transcription factor (AHRD V1 *-** D6MKM9_9ASPA); contains Interpro domain(s) IPR003340 Transcriptional factor B3 " | 4E-13 |
| 474 | Contig 474 | 246 | 1 | GH203877 | ********* |  |
| 475 | Contig 475 | 529 | 2 | GH204613 | Solyc03g033310.2.1 genomic_reference:SL2.40ch03 gene_region:9164130-9168788 transcript_region:SL2.40ch03:9164130..9168788- go_terms:GO:0003676,GO:0000166 functional_description:"RNA-binding protein (AHRD V1 *--- B6JZH8_SCHJY); contains Interpro domain(s) IPR012677 Nucleotide-binding, alpha-beta plait " | 6E-54 |
| 476 | Contig 476 | 613 | 1 | GH204275 | Solyc04g082200.2.1 genomic_reference:SL2.40ch04 gene_region:63550865-63552237 transcript_region:SL2.40ch04:63550865..63552237- go_terms:GO:0009415 functional_description:"Dehydrin (AHRD V1 *-*- Q1A4H3_COFCA); contains Interpro domain(s) IPR000167 Dehydrin " | 6E-30 |
| 477 | Contig 477 | 584 | 1 | GH204981 | ********* |  |
| 478 | Contig 478 | 908 | 1 | GH203659 | Solyc09g075180.2.1 genomic_reference:SL2.40ch09 gene_region:62453539-62456934 transcript_region:SL2.40ch09:62453539..62456934- go_terms:GO:0006281 functional_description:"Cryptochrome DASH family (AHRD V1 **-- B5W072_SPIMA); contains Interpro domain(s) IPR006050 DNA photolyase, N-terminal " | 1E-102 |
| 479 | Contig 479 | 254 | 1 | GH204692 | Solyc11g067100.1.1 evidence_code:10F1H0E1IEG genomic_reference:SL2.40ch11 gene_region:49928225-49929780 transcript_region:SL2.40ch11:49928225..49929780- go_terms:GO:0005840 functional_description:"60s acidic ribosomal protein-like protein (AHRD V1 ***- Q3HVP0_SOLTU); contains Interpro domain(s) IPR001813 Ribosomal protein 60S " | 2E-28 |
| 480 | Contig 480 | 628 | 1 | GH205059 | Solyc12g055750.1.1 evidence_code:10F0H1E1IEG genomic_reference:SL2.40ch12 gene_region:47093237-47100681 transcript_region:SL2.40ch12:47093237..47100681- go_terms:GO:0055085,GO:0016021 functional_description:"Vacuolar cation/proton exchanger 2 (AHRD V1 ***- B6UEI1_MAIZE); contains Interpro domain(s) IPR004713 Calcium/proton exchanger " | 3E-86 |
| 481 | Contig 481 | 808 | 1 | GH204688 | Solyc06g011280.2.1 genomic_reference:SL2.40ch06 gene_region:6257892-6260968 transcript_region:SL2.40ch06:6257892..6260968- go_terms:GO:0003713,GO:0005507 functional_description:"Elongation factor 1-gamma (AHRD V1 ***- B7SDI2_ORYSJ); contains Interpro domain(s) IPR001662 Translation elongation factor EF1B, gamma chain, conserved " | 1E-118 |
| 482 | Contig 482 | 104 | 1 | GH203523 | ********* |  |
| 483 | Contig 483 | 344 | 1 | GH204618 | Solyc03g095770.2.1 genomic_reference:SL2.40ch03 gene_region:50421318-50423492 transcript_region:SL2.40ch03:50421318..50423492+ go_terms:GO:0003700 functional_description:"WRKY transcription factor 6 (AHRD V1 **** A7UGD3_SOLTU); contains Interpro domain(s) IPR003657 DNA-binding WRKY " | 1E-24 |
| 484 | Contig 484 | 445 | 1 | GH204194 | Solyc11g011470.1.1 evidence_code:10F0H1E1IEG genomic_reference:SL2.40ch11 gene_region:4520459-4526103 transcript_region:SL2.40ch11:4520459..4526103- go_terms:GO:0016020,GO:0051536,GO:0016491,GO:0055114 functional_description:"NADH-ubiquinone oxidoreductase subunit (AHRD V1 ***- B9T118_RICCO); contains Interpro domain(s) IPR010228 NADH:ubiquinone oxidoreductase, subunit G " | 2E-15 |
| 485 | Contig 485 | 641 | 1 | GH203617 | Solyc06g073790.2.1 genomic_reference:SL2.40ch06 gene_region:41938647-41940686 transcript_region:SL2.40ch06:41938647..41940686- go_terms:GO:0003735,GO:0005515 functional_description:"40S ribosomal protein S11 (AHRD V1 ***- D7MBM0_ARALY); contains Interpro domain(s) IPR000266 Ribosomal protein S17 " | 9E-77 |
| 486 | Contig 486 | 749 | 1 | GH204289 | Solyc11g069490.1.1 evidence_code:10F0H1E1IEG genomic_reference:SL2.40ch11 gene_region:51168203-51179394 transcript_region:SL2.40ch11:51168203..51179394+ functional_description:"Serine/threonine-protein phosphatase 6 regulatory subunit 3 (AHRD V1 *-*- SAPS3_CHICK); contains Interpro domain(s) IPR007587 SIT4 phosphatase-associated protein " | 1E-124 |
| 487 | Contig 487 | 298 | 1 | GH204240 | Solyc06g083190.2.1 genomic_reference:SL2.40ch06 gene_region:45004847-45008910 transcript_region:SL2.40ch06:45004847..45008910- go_terms:GO:0005528 functional_description:"Peptidyl-prolyl cis-trans isomerase (AHRD V1 ***- Q86M29_BRUMA); contains Interpro domain(s) IPR001179 Peptidyl-prolyl cis-trans isomerase, FKBP-type " | 8E-23 |
| 488 | Contig 488 | 688 | 1 | GH204741 | Solyc03g113910.2.1 genomic_reference:SL2.40ch03 gene_region:58001941-58004317 transcript_region:SL2.40ch03:58001941..58004317+ functional_description:"Gibberellin-regulated protein 2 (AHRD V1 *--- B6SKV6_MAIZE); contains Interpro domain(s) IPR003854 Gibberellin regulated protein " | 2E-32 |
| 489 | Contig 489 | 329 | 1 | GH204912 | Solyc08g067910.2.1 genomic_reference:SL2.40ch08 gene_region:54158711-54167363 transcript_region:SL2.40ch08:54158711..54167363- go_terms:GO:0005484 functional_description:"Syntaxin 32 (AHRD V1 ***- B6TET0_MAIZE); contains Interpro domain(s) IPR010989 t-SNARE " | 8E-39 |
| 490 | Contig 490 | 678 | 1 | GH204407 | Solyc07g066470.2.1 genomic_reference:SL2.40ch07 gene_region:65112777-65116118 transcript_region:SL2.40ch07:65112777..65116118- go_terms:GO:0004418 functional_description:"Porphobilinogen deaminase (AHRD V1 ***- B6TVP0_MAIZE); contains Interpro domain(s) IPR000860 Tetrapyrrole biosynthesis, hydroxymethylbilane synthase " | 1E-101 |
| 491 | Contig 491 | 545 | 1 | GH204042 | Solyc07g005700.2.1 genomic_reference:SL2.40ch07 gene_region:577484-584465 transcript_region:SL2.40ch07:577484..584465- go_terms:GO:0008270 functional_description:"Unknown Protein (AHRD V1); contains Interpro domain(s) IPR003604 Zinc finger, U1-type " | 7E-24 |
| 492 | Contig 492 | 295 | 1 | GH204265 | Solyc06g048510.2.1 genomic_reference:SL2.40ch06 gene_region:27661455-27665948 transcript_region:SL2.40ch06:27661455..27665948+ go_terms:GO:0032266 functional_description:"WD-repeat domain phosphoinositide-interacting protein 3 (AHRD V1 **-- B6SUF7_MAIZE); contains Interpro domain(s) IPR011046 WD40 repeat-like " | 7E-51 |
| 493 | Contig 493 | 549 | 1 | GH204409 | Solyc11g033280.1.1 evidence_code:10F1H1E1IEG genomic_reference:SL2.40ch11 gene_region:22986368-22991947 transcript_region:SL2.40ch11:22986368..22991947- go_terms:GO:0008026,GO:0005524 functional_description:"ATP-dependent RNA helicase FAL1 (AHRD V1 ***- A8NX16_COPC7); contains Interpro domain(s) IPR011545 DNA/RNA helicase, DEAD/DEAH box type, N-terminal " | 1E-97 |
| 494 | Contig 494 | 841 | 1 | GH204259 | Solyc08g005150.2.1 genomic_reference:SL2.40ch08 gene_region:97549-104036 transcript_region:SL2.40ch08:97549..104036+ go_terms:GO:0003713,GO:0004842 functional_description:"Ubiquitin ligase (AHRD V1 **-* B3G3Y7_ADIVA); contains Interpro domain(s) IPR006575 RWD " | 7E-36 |
| 495 | Contig 495 | 677 | 1 | GH205014 | Solyc01g111240.2.1 genomic_reference:SL2.40ch01 gene_region:89317519-89321842 transcript_region:SL2.40ch01:89317519..89321842- go_terms:GO:0005525 functional_description:"Translocase of chloroplast 90, chloroplastic (AHRD V1 *--- TOC90_ARATH); contains Interpro domain(s) IPR005690 Chloroplast protein import component Toc86/159 " | 6E-72 |
| 496 | Contig 496 | 420 | 1 | GH203695 | Solyc04g079550.2.1 genomic_reference:SL2.40ch04 gene_region:61565562-61568797 transcript_region:SL2.40ch04:61565562..61568797- go_terms:GO:0004175 functional_description:"Signal peptidase I (AHRD V1 *-*- B4W1T4_9CYAN); contains Interpro domain(s) IPR000223 Peptidase S26A, signal peptidase I " | 8E-14 |
| 497 | Contig 497 | 385 | 1 | GH204513 | Solyc07g056480.2.1 genomic_reference:SL2.40ch07 gene_region:61623166-61624189 transcript_region:SL2.40ch07:61623166..61624189+ go_terms:GO:0004364,GO:0043295 functional_description:"Glutathione S-transferase-like protein (AHRD V1 **** Q8GVD1_SOLLC); contains Interpro domain(s) IPR004046 Glutathione S-transferase, C-terminal " | 1E-72 |
| 498 | Contig 498 | 212 | 1 | GH204755 | Solyc12g094480.1.1 evidence_code:10F0H1E0IEG genomic_reference:SL2.40ch12 gene_region:63040511-63043542 transcript_region:SL2.40ch12:63040511..63043542- functional_description:"Unknown Protein (AHRD V1)" | 1E-14 |
| 499 | Contig 499 | 714 | 1 | GH203423 | Solyc02g078040.2.1 genomic_reference:SL2.40ch02 gene_region:37435419-37436560 transcript_region:SL2.40ch02:37435419..37436560- go_terms:GO:0005199 functional_description:"Pistil extensin like protein (Fragment) (AHRD V1 *-*- Q40552_TOBAC); contains Interpro domain(s) IPR006041 Pollen Ole e 1 allergen and extensin " | 3E-56 |
| 500 | Contig 500 | 533 | 1 | GH205129 | Solyc10g054330.1.1 evidence_code:10F0H1E1IEG genomic_reference:SL2.40ch10 gene_region:50729498-50732695 transcript_region:SL2.40ch10:50729498..50732695+ go_terms:GO:0008270 functional_description:"Cellular nucleic acid-binding protein (AHRD V1 **-- B6T3W5_MAIZE); contains Interpro domain(s) IPR013084 Zinc finger, CCHC retroviral-type " | 1E-101 |
| 501 | Contig 501 | 740 | 1 | GH203743 | Solyc04g079310.2.1 genomic_reference:SL2.40ch04 gene_region:61412534-61417774 transcript_region:SL2.40ch04:61412534..61417774+ go_terms:GO:0042802 functional_description:"RNA Binding Protein 47 (AHRD V1 **-* Q9LEB3_NICPL); contains Interpro domain(s) IPR012677 Nucleotide-binding, alpha-beta plait " | 7E-90 |
| 502 | Contig 502 | 789 | 1 | GH204165 | Solyc07g066610.2.1 genomic_reference:SL2.40ch07 gene_region:65209792-65212260 transcript_region:SL2.40ch07:65209792..65212260- go_terms:GO:0005524 functional_description:"Phosphoglycerate kinase (AHRD V1 ***- O81394_SOLTU); contains Interpro domain(s) IPR001576 Phosphoglycerate kinase " | 1E-138 |
| 503 | Contig 503 | 514 | 1 | GH203432 | Solyc02g091740.2.1 genomic_reference:SL2.40ch02 gene_region:47555499-47557404 transcript_region:SL2.40ch02:47555499..47557404+ functional_description:"Unknown Protein (AHRD V1); contains Interpro domain(s) IPR013256 Chromatin SPT2 " | 4E-23 |
| 504 | Contig 504 | 228 | 1 | GH204877 | ********* |  |
| 505 | Contig 505 | 235 | 1 | GH204884 | ********* |  |
| 506 | Contig 506 | 187 | 1 | GH204363 | ********* |  |
| 507 | Contig 507 | 273 | 1 | GH204477 | Solyc06g065970.1.1 evidence_code:10F1H1E1IEG genomic_reference:SL2.40ch06 gene_region:37743284-37743664 transcript_region:SL2.40ch06:37743284..37743664+ go_terms:GO:0008289 functional_description:"Cortical cell-delineating protein (AHRD V1 **-- B6UGA2_MAIZE); contains Interpro domain(s) IPR013770 Plant lipid transfer protein and hydrophobic protein, helical " | 3E-35 |
| 508 | Contig 508 | 807 | 1 | GH205001 | Solyc05g024290.2.1 genomic_reference:SL2.40ch05 gene_region:31012552-31022465 transcript_region:SL2.40ch05:31012552..31022465- go_terms:GO:0005515,GO:0004674 functional_description:"Serine/threonine-protein kinase (AHRD V1 **** C6ZRV0_SOYBN); contains Interpro domain(s) IPR002290 Serine/threonine protein kinase " | 1E-147 |
| 509 | Contig 509 | 107 | 1 | GH204266 | ********* |  |
| 510 | Contig 510 | 815 | 1 | GH204880 | Solyc12g013690.1.1 evidence_code:10F1H1E1IEG genomic_reference:SL2.40ch12 gene_region:4532510-4535243 transcript_region:SL2.40ch12:4532510..4535243- go_terms:GO:0008152,GO:0016491 functional_description:"Monooxygenase FAD-binding protein (AHRD V1 **-- Q1B616_MYCSS); contains Interpro domain(s) IPR003042 Aromatic-ring hydroxylase-like " | 2E-44 |
| 511 | Contig 511 | 313 | 1 | GH204610 | Solyc04g054810.2.1 genomic_reference:SL2.40ch04 gene_region:52432873-52434340 transcript_region:SL2.40ch04:52432873..52434340+ functional_description:"Pollen allergen Phl p 11 (AHRD V1 ***- B6T2Z8_MAIZE); contains Interpro domain(s) IPR006041 Pollen Ole e 1 allergen and extensin " | 2E-19 |
| 512 | Contig 512 | 463 | 1 | GH204887 | Solyc11g017470.1.1 evidence_code:10F1H1E1IEG genomic_reference:SL2.40ch11 gene_region:8422146-8423170 transcript_region:SL2.40ch11:8422146..8423170- go_terms:GO:0003700,GO:0016563 functional_description:"NAC domain protein IPR003441 (AHRD V1 ***- B9GU14_POPTR); contains Interpro domain(s) IPR003441 No apical meristem (NAM) protein " | 1E-30 |
| 513 | Contig 513 | 321 | 1 | GH204246 | Solyc02g070650.2.1 genomic_reference:SL2.40ch02 gene_region:34912165-34914546 transcript_region:SL2.40ch02:34912165..34914546+ go_terms:GO:0003735,GO:0005515 functional_description:"60S ribosomal protein L18a (AHRD V1 ***- D2D962_9ROSI); contains Interpro domain(s) IPR002670 Ribosomal protein L18ae " | 1E-34 |
| 514 | Contig 514 | 728 | 1 | GH204249 | Solyc06g083920.2.1 genomic_reference:SL2.40ch06 gene_region:45505640-45511030 transcript_region:SL2.40ch06:45505640..45511030+ go_terms:GO:0006629 functional_description:"Lipase family protein (AHRD V1 *-*- Q2R051_ORYSJ); contains Interpro domain(s) IPR002921 Lipase, class 3 " | 5E-22 |
| 515 | Contig 515 | 835 | 1 | GH204870 | ********* |  |
| 516 | Contig 516 | 309 | 1 | GH204228 | Solyc01g096200.2.1 genomic_reference:SL2.40ch01 gene_region:79051800-79061053 transcript_region:SL2.40ch01:79051800..79061053- go_terms:GO:0005488,GO:0000151 functional_description:"Pentatricopeptide repeat-containing protein (AHRD V1 *-*- D7M1E8_ARALY); contains Interpro domain(s) IPR011989 Armadillo-like helical " | 4E-51 |
| 517 | Contig 517 | 217 | 1 | GH204872 | Solyc04g074510.2.1 genomic_reference:SL2.40ch04 gene_region:58094139-58096300 transcript_region:SL2.40ch04:58094139..58096300+ go_terms:GO:0050815 functional_description:"14-3-3 protein beta/alpha-B (AHRD V1 **-- 143BB_XENLA); contains Interpro domain(s) IPR000308 14-3-3 protein " | 2E-23 |
| 518 | Contig 518 | 558 | 1 | GH204217 | Solyc03g078080.2.1 genomic_reference:SL2.40ch03 gene_region:43589225-43595462 transcript_region:SL2.40ch03:43589225..43595462+ go_terms:GO:0005515,GO:0009041 functional_description:"Uridylate kinase (AHRD V1 **** B6TC37_MAIZE); contains Interpro domain(s) IPR015963 Uridylate kinase, bacteria " | 4E-99 |
| 519 | Contig 519 | 546 | 1 | GH204211 | Solyc02g091280.2.1 genomic_reference:SL2.40ch02 gene_region:47199858-47204562 transcript_region:SL2.40ch02:47199858..47204562- go_terms:GO:0005515,GO:0004176 functional_description:"ATP-dependent Clp protease proteolytic subunit (AHRD V1 **** B9H362_POPTR); contains Interpro domain(s) IPR001907 Peptidase S14, ClpP " | 5E-93 |
| 520 | Contig 520 | 367 | 1 | GH204605 | Solyc08g062910.2.1 genomic_reference:SL2.40ch08 gene_region:49531008-49535044 transcript_region:SL2.40ch08:49531008..49535044- go_terms:GO:0003746 functional_description:"Elongation factor EF-2 (AHRD V1 **** Q9SGT4_ARATH); contains Interpro domain(s) IPR000795 Protein synthesis factor, GTP-binding " | 3E-70 |
| 521 | Contig 521 | 386 | 1 | GH204236 | Solyc07g062570.2.1 genomic_reference:SL2.40ch07 gene_region:62472541-62478080 transcript_region:SL2.40ch07:62472541..62478080+ go_terms:GO:0043130 functional_description:"Ubiquitin-conjugating enzyme E2 N (AHRD V1 **** B5XC59_SALSA); contains Interpro domain(s) IPR000608 Ubiquitin-conjugating enzyme, E2 " | 2E-34 |
| 522 | Contig 522 | 791 | 1 | GH204232 | Solyc09g082860.2.1 genomic_reference:SL2.40ch09 gene_region:63941988-63946072 transcript_region:SL2.40ch09:63941988..63946072+ go_terms:GO:0004781 functional_description:"Sulfate adenylyltransferase (AHRD V1 **** Q43183_SOLTU); contains Interpro domain(s) IPR002650 ATP-sulfurylase " | 1E-43 |
| 523 | Contig 523 | 159 | 1 | GH204230 | ********* |  |
| 524 | Contig 524 | 715 | 1 | GH204207 | Solyc03g046470.2.1 genomic_reference:SL2.40ch03 gene_region:16871256-16881175 transcript_region:SL2.40ch03:16871256..16881175- functional_description:"Auxin-independent growth promoter protein-like (AHRD V1 **-- Q9LI68_ARATH); contains Interpro domain(s) IPR004348 Protein of unknown function DUF246, plant " | 1E-115 |
| 525 | Contig 525 | 173 | 1 | GH204864 | Solyc10g086280.1.1 evidence_code:10F0H1E1IEG genomic_reference:SL2.40ch10 gene_region:64482821-64484140 transcript_region:SL2.40ch10:64482821..64484140- go_terms:GO:0030001 functional_description:"Heavy metal-associated domain containing protein expressed (AHRD V1 *-*- Q10RN8_ORYSJ); contains Interpro domain(s) IPR006121 Heavy metal transport/detoxification protein " | 1E-26 |
| 526 | Contig 526 | 386 | 1 | GH204519 | Solyc02g093900.2.1 genomic_reference:SL2.40ch02 gene_region:49178107-49182974 transcript_region:SL2.40ch02:49178107..49182974- go_terms:GO:0045153 functional_description:"Cytochrome c1 (AHRD V1 **** Q9FKS5_ARATH); contains Interpro domain(s) IPR002326 Cytochrome c1 " | 1E-41 |
| 527 | Contig 527 | 816 | 1 | GH204494 | Solyc02g021700.2.1 genomic_reference:SL2.40ch02 gene_region:14281494-14294175 transcript_region:SL2.40ch02:14281494..14294175- go_terms:GO:0005515,GO:0008276 functional_description:"Alpha N-terminal protein methyltransferase 1 (AHRD V1 **** NTM1_ARATH); contains Interpro domain(s) IPR008576 Protein of unknown function DUF858, methyltransferase-like " | 3E-68 |
| 528 | Contig 528 | 561 | 1 | GH205251 | Solyc03g123620.2.1 genomic_reference:SL2.40ch03 gene_region:64472559-64475713 transcript_region:SL2.40ch03:64472559..64475713- go_terms:GO:0005618,GO:0030599 functional_description:"Pectinesterase (AHRD V1 ***- B9GXZ7_POPTR); contains Interpro domain(s) IPR000070 Pectinesterase, catalytic " | 1E-103 |
| 529 | Contig 529 | 613 | 1 | GH204242 | Solyc12g015780.1.1 evidence_code:10F0H1E1IEG genomic_reference:SL2.40ch12 gene_region:5775862-5780527 transcript_region:SL2.40ch12:5775862..5780527- go_terms:GO:0006350 functional_description:"DNA-directed RNA polymerase subunit D (AHRD V1 ***- A8B9C8_GIALA); contains Interpro domain(s) IPR011263 DNA-directed RNA polymerase, RpoA/D/Rpb3-type " | 8E-59 |
| 530 | Contig 530 | 380 | 1 | GH204241 | Solyc04g077970.2.1 genomic_reference:SL2.40ch04 gene_region:60430505-60435397 transcript_region:SL2.40ch04:60430505..60435397- go_terms:GO:0016208,GO:0003999 functional_description:"Adenine phosphoribosyltransferase-like (AHRD V1 **** Q2V997_SOLTU); contains Interpro domain(s) IPR005764 Adenine phosphoribosyl transferase " | 2E-67 |
| 531 | Contig 531 | 718 | 1 | GH204364 | Solyc06g074030.1.1 evidence_code:10F0H1E1IEG genomic_reference:SL2.40ch06 gene_region:42156677-42157471 transcript_region:SL2.40ch06:42156677..42157471- go_terms:GO:0000175 functional_description:"CCR4-NOT transcription complex subunit 7 (AHRD V1 ***- B4FG48_MAIZE); contains Interpro domain(s) IPR006941 Ribonuclease CAF1 " | 1E-120 |
| 532 | Contig 532 | 344 | 1 | GH204895 | ********* |  |
| 533 | Contig 533 | 370 | 1 | GH204516 | Solyc05g054310.2.1 genomic_reference:SL2.40ch05 gene_region:63392299-63395734 transcript_region:SL2.40ch05:63392299..63395734+ go_terms:GO:0003743 functional_description:"Translation initiation factor 2 gamma subunit (AHRD V1 **** Q684L1_9INSE); contains Interpro domain(s) IPR015256 Initiation factor eIF2 gamma, C-terminal " | 6E-62 |
| 534 | Contig 534 | 538 | 1 | GH204210 | Solyc04g077270.2.1 genomic_reference:SL2.40ch04 gene_region:59836299-59839484 transcript_region:SL2.40ch04:59836299..59839484- go_terms:GO:0019199,GO:0004674 functional_description:"Serine/threonine kinase receptor (AHRD V1 **** Q7DMS5_BRANA); contains Interpro domain(s) IPR002290 Serine/threonine protein kinase " | 1E-108 |
| 535 | Contig 535 | 757 | 1 | GH204886 | Solyc02g070770.2.1 genomic_reference:SL2.40ch02 gene_region:34960477-34964329 transcript_region:SL2.40ch02:34960477..34964329- go_terms:GO:0044237,GO:0008152 functional_description:"NAD-dependent epimerase/dehydratase (AHRD V1 **-- B8HW76_CYAP4); contains Interpro domain(s) IPR016040 NAD(P)-binding domain " | 1E-124 |
| 536 | Contig 536 | 624 | 1 | GH204218 | Solyc02g080640.2.1 genomic_reference:SL2.40ch02 gene_region:39406300-39409809 transcript_region:SL2.40ch02:39406300..39409809+ go_terms:GO:0009973 functional_description:"Phosphoadenosine phosphosulfate reductase (AHRD V1 *-** B7I7M3_ACIB5); contains Interpro domain(s) IPR013766 Thioredoxin domain " | 1E-124 |
| 537 | Contig 537 | 574 | 1 | GH204714 | Solyc08g076540.2.1 genomic_reference:SL2.40ch08 gene_region:57691305-57695575 transcript_region:SL2.40ch08:57691305..57695575+ go_terms:GO:0005484 functional_description:"Syntaxin-like protein (AHRD V1 ***- Q3HRZ4_SOLTU); contains Interpro domain(s) IPR010989 t-SNARE " | 3E-82 |
| 538 | Contig 538 | 205 | 1 | GH204255 | Solyc02g064680.2.1 genomic_reference:SL2.40ch02 gene_region:30333568-30339094 transcript_region:SL2.40ch02:30333568..30339094+ go_terms:GO:0016020,GO:0008152 functional_description:"Calcium-transporting ATPase 1 (AHRD V1 ***- Q7XBH9_CERRI); contains Interpro domain(s) IPR006408 ATPase, P-type, calcium-transporting, PMCA-type " | 3E-33 |
| 539 | Contig 539 | 229 | 1 | GH204412 | ********* |  |
| 540 | Contig 540 | 597 | 1 | GH204444 | Solyc06g082930.2.1 genomic_reference:SL2.40ch06 gene_region:44846738-44850138 transcript_region:SL2.40ch06:44846738..44850138+ functional_description:"FRIGIDA (Fragment) (AHRD V1 *-*- Q58T23_ARATH); contains Interpro domain(s) IPR012474 Frigida-like " | 1E-108 |
| 541 | Contig 541 | 268 | 1 | GH204474 | Solyc05g056310.2.1 genomic_reference:SL2.40ch05 gene_region:64792047-64799309 transcript_region:SL2.40ch05:64792047..64799309+ go_terms:GO:0051082 functional_description:"T-complex protein 1 subunit gamma (AHRD V1 ***- B6UCD0_MAIZE); contains Interpro domain(s) IPR012719 T-complex protein 1, gamma subunit " | 1E-41 |
| 542 | Contig 542 | 556 | 1 | GH204411 | Solyc02g087350.1.1 evidence_code:10F0H1E1IEG genomic_reference:SL2.40ch02 gene_region:44354006-44355106 transcript_region:SL2.40ch02:44354006..44355106- go_terms:GO:0016758 functional_description:"Glycosyltransferase (AHRD V1 ***- B9N4D7_POPTR); contains Interpro domain(s) IPR002495 Glycosyl transferase, family 8 " | 8E-92 |
| 543 | Contig 543 | 413 | 1 | GH204749 | Solyc04g049690.2.1 genomic_reference:SL2.40ch04 gene_region:42201867-42210091 transcript_region:SL2.40ch04:42201867..42210091- go_terms:GO:0004017 functional_description:"Adenylate kinase (AHRD V1 **** B6SLP1_MAIZE); contains Interpro domain(s) IPR006259 Adenylate kinase, subfamily " | 2E-43 |
| 544 | Contig 544 | 103 | 1 | GH204431 | ********* |  |
| 545 | Contig 545 | 388 | 1 | GH204602 | Solyc04g047690.2.1 genomic_reference:SL2.40ch04 gene_region:34743223-34744156 transcript_region:SL2.40ch04:34743223..34744156+ go_terms:GO:0016021 functional_description:"SFT2 domain containing 2 (AHRD V1 **-- B6TGT8_MAIZE); contains Interpro domain(s) IPR011691 SFT2-like " | 4E-26 |
| 546 | Contig 546 | 412 | 1 | GH204408 | Solyc08g029000.2.1 genomic_reference:SL2.40ch08 gene_region:21980663-21984456 transcript_region:SL2.40ch08:21980663..21984456+ go_terms:GO:0016165 functional_description:"Lipoxygenase (AHRD V1 **** Q43191_SOLTU); contains Interpro domain(s) IPR001246 Lipoxygenase, plant " | 1E-74 |
| 547 | Contig 547 | 411 | 1 | GH204438 | Solyc07g007050.1.1 evidence_code:10F1H0E1IEG genomic_reference:SL2.40ch07 gene_region:1832990-1833349 transcript_region:SL2.40ch07:1832990..1833349+ functional_description:"Unknown Protein (AHRD V1)" | 2E-20 |
| 548 | Contig 548 | 758 | 1 | GH204730 | Solyc02g087300.1.1 evidence_code:10F0H1E1IEG genomic_reference:SL2.40ch02 gene_region:44325520-44326428 transcript_region:SL2.40ch02:44325520..44326428+ go_terms:GO:0005515 functional_description:"Protein transport SEC13-like protein (AHRD V1 **-- Q2PYY1_SOLTU); contains Interpro domain(s) IPR020472 G-protein beta WD-40 repeat, region " | 1E-108 |
| 549 | Contig 549 | 357 | 1 | GH204592 | Solyc05g014470.2.1 genomic_reference:SL2.40ch05 gene_region:8322381-8324881 transcript_region:SL2.40ch05:8322381..8324881+ go_terms:GO:0004365 functional_description:"Glyceraldehyde 3-phosphate dehydrogenase (AHRD V1 **** Q8LK04_SOLTU); contains Interpro domain(s) IPR000173 Glyceraldehyde 3-phosphate dehydrogenase " | 3E-51 |
| 550 | Contig 550 | 469 | 1 | GH204375 | Solyc02g079250.2.1 genomic_reference:SL2.40ch02 gene_region:38432003-38443209 transcript_region:SL2.40ch02:38432003..38443209+ go_terms:GO:0008441 functional_description:"3&apos(2&apos) 5&apos-bisphosphate nucleotidase-like protein (AHRD V1 **** Q682R6_ARATH); contains Interpro domain(s) IPR000760 Inositol monophosphatase " | 3E-83 |
| 551 | Contig 551 | 518 | 1 | GH204465 | Solyc11g008580.1.1 evidence_code:10F0H1E1IEG genomic_reference:SL2.40ch11 gene_region:2764823-2775320 transcript_region:SL2.40ch11:2764823..2775320- go_terms:GO:0008270,GO:0005622 functional_description:"Ariadne-like ubiquitin ligase (AHRD V1 **-- D3AZ84_POLPA); contains Interpro domain(s) IPR002867 Zinc finger, C6HC-type " | 1E-75 |
| 552 | Contig 552 | 311 | 1 | GH204442 | Solyc11g069000.1.1 evidence_code:10F1H1E1IEG genomic_reference:SL2.40ch11 gene_region:50652125-50657452 transcript_region:SL2.40ch11:50652125..50657452- go_terms:GO:0044267,GO:0006457 functional_description:"T-complex protein 1 subunit beta (AHRD V1 ***- B6T8X5_MAIZE); contains Interpro domain(s) IPR012716 T-complex protein 1, beta subunit " | 6E-52 |
| 553 | Contig 553 | 265 | 1 | GH204905 | Solyc04g080940.2.1 genomic_reference:SL2.40ch04 gene_region:62579308-62582332 transcript_region:SL2.40ch04:62579308..62582332- go_terms:GO:0016020 functional_description:"Nodulin-like protein (AHRD V1 ***- Q94AP3_ARATH); contains Interpro domain(s) IPR000620 Protein of unknown function DUF6, transmembrane " | 1E-47 |
| 554 | Contig 554 | 125 | 1 | GH204227 | ********* |  |
| 555 | Contig 555 | 718 | 1 | GH204759 | Solyc01g080750.2.1 genomic_reference:SL2.40ch01 gene_region:72488752-72490161 transcript_region:SL2.40ch01:72488752..72490161+ go_terms:GO:0005488 functional_description:"HEAT repeat family protein (AHRD V1 **-- B4FX70_MAIZE); contains Interpro domain(s) IPR016024 Armadillo-type fold " | 1E-102 |
| 556 | Contig 556 | 309 | 1 | GH204756 | ********* |  |
| 557 | Contig 557 | 529 | 1 | GH204803 | Solyc08g014130.2.1 genomic_reference:SL2.40ch08 gene_region:3734998-3744536 transcript_region:SL2.40ch08:3734998..3744536+ go_terms:GO:0003852 functional_description:"2-isopropylmalate synthase 1 (AHRD V1 **** Q30DX9_9BRAS); contains Interpro domain(s) IPR005671 Bacterial 2-isopropylmalate synthase " | 5E-71 |
| 558 | Contig 558 | 499 | 1 | GH204425 | ********* |  |
| 559 | Contig 559 | 441 | 1 | GH204421 | ********* |  |
| 560 | Contig 560 | 433 | 1 | GH204881 | Solyc09g005980.2.1 genomic_reference:SL2.40ch09 gene_region:670381-673543 transcript_region:SL2.40ch09:670381..673543+ go_terms:GO:0003676,GO:0008270,GO:0000166 functional_description:"Splicing factor arginine/serine-rich 6 (AHRD V1 *--- B6T453_MAIZE); contains Interpro domain(s) IPR012677 Nucleotide-binding, alpha-beta plait " | 9E-16 |
| 561 | Contig 561 | 455 | 1 | GH204372 | ********* |  |
| 562 | Contig 562 | 468 | 1 | GH204359 | Solyc11g008510.1.1 evidence_code:10F1H1E1IEG genomic_reference:SL2.40ch11 gene_region:2684326-2686166 transcript_region:SL2.40ch11:2684326..2686166+ go_terms:GO:0005840 functional_description:"60S ribosomal protein L38 (AHRD V1 ***- D7LK18_ARALY); contains Interpro domain(s) IPR002675 Ribosomal protein L38e " | 2E-21 |
| 563 | Contig 563 | 381 | 1 | GH204733 | Solyc02g080630.2.1 genomic_reference:SL2.40ch02 gene_region:39392539-39397791 transcript_region:SL2.40ch02:39392539..39397791- go_terms:GO:0046872 functional_description:"Lactoylglutathione lyase (AHRD V1 ***- D2D330_GOSHI); contains Interpro domain(s) IPR004361 Glyoxalase I " | 3E-61 |
| 564 | Contig 564 | 703 | 1 | GH204224 | Solyc04g058090.2.1 genomic_reference:SL2.40ch04 gene_region:54348345-54353968 transcript_region:SL2.40ch04:54348345..54353968- go_terms:GO:0008716 functional_description:"Phosphoribosylformylglycinamidin e cyclo-ligase (AHRD V1 **** Q6T7F2_SOLTU); contains Interpro domain(s) IPR004733 Phosphoribosylformylglycinamidine cyclo-ligase " | 1E-130 |
| 565 | Contig 565 | 443 | 1 | GH204420 | ********* |  |
| 566 | Contig 566 | 767 | 1 | GH204737 | Solyc01g060180.2.1 genomic_reference:SL2.40ch01 gene_region:62560881-62565105 transcript_region:SL2.40ch01:62560881..62565105- go_terms:GO:0008152 functional_description:"AMP-dependent synthetase and ligase (AHRD V1 ***- A1U2F4_MARAV); contains Interpro domain(s) IPR000873 AMP-dependent synthetase and ligase " | 1E-118 |
| 567 | Contig 567 | 512 | 1 | GH204514 | Solyc01g109110.2.1 genomic_reference:SL2.40ch01 gene_region:87904937-87909600 transcript_region:SL2.40ch01:87904937..87909600- go_terms:GO:0007186,GO:0007165 functional_description:"Guanine nucleotide-binding protein alpha-1 subunit (AHRD V1 *-*- B6TWS6_MAIZE); contains Interpro domain(s) IPR001019 Guanine nucleotide binding protein (G-protein), alpha subunit " | 2E-81 |
| 568 | Contig 568 | 271 | 1 | GH204738 | ********* |  |
| 569 | Contig 569 | 770 | 1 | GH204731 | Solyc04g079200.2.1 genomic_reference:SL2.40ch04 gene_region:61339502-61344701 transcript_region:SL2.40ch04:61339502..61344701- go_terms:GO:0070628 functional_description:"26S proteasome regulatory subunit (AHRD V1 **** A8HPA0_CHLRE); contains Interpro domain(s) IPR000555 Mov34/MPN/PAD-1 " | 1E-130 |
| 570 | Contig 570 | 815 | 1 | GH204450 | Solyc01g103410.2.1 genomic_reference:SL2.40ch01 gene_region:83770913-83781702 transcript_region:SL2.40ch01:83770913..83781702- go_terms:GO:0003676 functional_description:"Nucleic acid binding protein (AHRD V1 **-* B6T8Q7_MAIZE); contains Interpro domain(s) IPR004087 K Homology " | 1E-123 |
| 571 | Contig 571 | 498 | 1 | GH204472 | Solyc07g053260.2.1 genomic_reference:SL2.40ch07 gene_region:59058945-59063496 transcript_region:SL2.40ch07:59058945..59063496+ go_terms:GO:0050815 functional_description:"14-3-3 protein sigma gamma zeta beta/alpha (AHRD V1 **-- Q16QZ7_AEDAE); contains Interpro domain(s) IPR000308 14-3-3 protein " | 8E-13 |
| 572 | Contig 572 | 760 | 1 | GH204739 | Solyc09g075890.2.1 genomic_reference:SL2.40ch09 gene_region:63027202-63029749 transcript_region:SL2.40ch09:63027202..63029749- go_terms:GO:0047405,GO:0008252 functional_description:"Pyrimidine 5&apos-nucleotidase (AHRD V1 **** B4WF69_9CAUL); contains Interpro domain(s) IPR010237 Pyrimidine 5-nucleotidase " | 3E-81 |
| 573 | Contig 573 | 203 | 1 | GH204769 | ********* |  |
| 574 | Contig 574 | 736 | 1 | GH204451 | Solyc09g015040.1.1 evidence_code:10F0H1E1IEG genomic_reference:SL2.40ch09 gene_region:7854568-7856100 transcript_region:SL2.40ch09:7854568..7856100+ functional_description:"Os08g0119500 protein (Fragment) (AHRD V1 *-*- Q0J8C9_ORYSJ)" | 1E-118 |
| 575 | Contig 575 | 652 | 1 | GH204349 | Solyc09g010940.2.1 genomic_reference:SL2.40ch09 gene_region:4278272-4282087 transcript_region:SL2.40ch09:4278272..4282087- go_terms:GO:0016787,GO:0008152 functional_description:"Pyrimidine 5&apos-nucleotidase (AHRD V1 ***- D5QKK7_METTR); contains Interpro domain(s) IPR010237 Pyrimidine 5-nucleotidase " | 1E-120 |
| 576 | Contig 576 | 301 | 1 | GH204449 | Solyc03g115360.2.1 genomic_reference:SL2.40ch03 gene_region:59143720-59146034 transcript_region:SL2.40ch03:59143720..59146034+ go_terms:GO:0005840 functional_description:"40S ribosomal protein S19-like (AHRD V1 ***- Q2VCJ6_SOLTU); contains Interpro domain(s) IPR001266 Ribosomal protein S19e " | 1E-31 |
| 577 | Contig 577 | 184 | 1 | GH204427 | Solyc02g083880.2.1 genomic_reference:SL2.40ch02 gene_region:41715236-41716114 transcript_region:SL2.40ch02:41715236..41716114+ go_terms:GO:0005515 functional_description:"Gibberellin-regulated protein 2 (AHRD V1 *--- B6SKV6_MAIZE); contains Interpro domain(s) IPR003854 Gibberellin regulated protein " | 3E-11 |
| 578 | Contig 578 | 834 | 1 | GH204362 | Solyc04g070980.2.1 genomic_reference:SL2.40ch04 gene_region:55417859-55433814 transcript_region:SL2.40ch04:55417859..55433814+ go_terms:GO:0000250 functional_description:"Lanosterol synthase (AHRD V1 **** Q1ERD3_LOTJA); contains Interpro domain(s) IPR018333 Squalene cyclase " | 1E-139 |
| 579 | Contig 579 | 134 | 1 | GH204454 | ********* |  |
| 580 | Contig 580 | 385 | 1 | GH204754 | ********* |  |
| 581 | Contig 581 | 219 | 1 | GH205289 | ********* |  |
| 582 | Contig 582 | 788 | 1 | GH204435 | Solyc03g111850.2.1 genomic_reference:SL2.40ch03 gene_region:56510892-56516492 transcript_region:SL2.40ch03:56510892..56516492- go_terms:GO:0004425,GO:0004635 functional_description:"Indole-3-glycerol phosphate synthase-like (AHRD V1 **** C8TFG9_ORYSI); contains Interpro domain(s) IPR013798 Indole-3-glycerol phosphate synthase " | 3E-62 |
| 583 | Contig 583 | 189 | 1 | GH203597 | Solyc07g017220.2.1 genomic_reference:SL2.40ch07 gene_region:6515317-6518394 transcript_region:SL2.40ch07:6515317..6518394- functional_description:"Os10g0422600 protein (Fragment) (AHRD V1 *-*- Q0IXM0_ORYSJ); contains Interpro domain(s) IPR007650 Protein of unknown function DUF581 " | 5E-28 |
| 584 | Contig 584 | 743 | 1 | GH205269 | Solyc11g056680.1.1 evidence_code:10F0H1E1IEG genomic_reference:SL2.40ch11 gene_region:45139882-45140976 transcript_region:SL2.40ch11:45139882..45140976+ go_terms:GO:0004675 functional_description:"LRR receptor-like serine/threonine-protein kinase, RLP" | 1E-75 |
| 585 | Contig 585 | 479 | 1 | GH204863 | Solyc02g021400.1.1 evidence_code:10F1H0E1IEG genomic_reference:SL2.40ch02 gene_region:13315741-13315938 transcript_region:SL2.40ch02:13315741..13315938+ go_terms:GO:0003735 functional_description:"40S ribosomal protein S28 (AHRD V1 ***- B6T1V2_MAIZE); contains Interpro domain(s) IPR000289 Ribosomal protein S28e " | 2E-20 |
| 586 | Contig 586 | 303 | 1 | GH203545 | Solyc04g009740.2.1 genomic_reference:SL2.40ch04 gene_region:3062113-3072311 transcript_region:SL2.40ch04:3062113..3072311- go_terms:GO:0005515 functional_description:"Exocyst complex protein EXO70 (AHRD V1 *--- EXO70_YARLI); contains Interpro domain(s) IPR004140 Exo70 exocyst complex subunit " | 4E-38 |
| 587 | Contig 587 | 377 | 1 | GH205287 | Solyc01g100570.2.1 genomic_reference:SL2.40ch01 gene_region:82320225-82324027 transcript_region:SL2.40ch01:82320225..82324027- go_terms:GO:0005515,GO:0030515 functional_description:"Nucleolar protein (AHRD V1 ***- Q9LTV0_ARATH); contains Interpro domain(s) IPR002687 Pre-mRNA processing ribonucleoprotein, binding region " | 3E-32 |
| 588 | Contig 588 | 644 | 1 | GH204441 | Solyc06g073730.1.1 evidence_code:10F1H1E1IEG genomic_reference:SL2.40ch06 gene_region:41879453-41881270 transcript_region:SL2.40ch06:41879453..41881270- go_terms:GO:0005515,GO:0003700 functional_description:"Ethylene insensitive 3 class transcription factor (AHRD V1 **** D5L146_MALDO); contains Interpro domain(s) IPR006957 Ethylene insensitive 3 " | 1E-123 |
| 589 | Contig 589 | 593 | 1 | GH204437 | Solyc03g117060.2.1 genomic_reference:SL2.40ch03 gene_region:60374016-60378796 transcript_region:SL2.40ch03:60374016..60378796- go_terms:GO:0042803 functional_description:"60S ribosomal protein L7-like protein (AHRD V1 ***- B3TM16_ELAGV); contains Interpro domain(s) IPR005998 Ribosomal protein L7, eukaryotic " | 6E-72 |
| 590 | Contig 590 | 590 | 1 | GH203643 | Solyc07g006890.1.1 evidence_code:10F0H1E1IEG genomic_reference:SL2.40ch07 gene_region:1747021-1748529 transcript_region:SL2.40ch07:1747021..1748529+ go_terms:GO:0019825 functional_description:"Cytochrome P450" | 1E-67 |
| 591 | Contig 591 | 596 | 1 | GH205290 | Solyc06g053480.2.1 genomic_reference:SL2.40ch06 gene_region:32770333-32774663 transcript_region:SL2.40ch06:32770333..32774663+ go_terms:GO:0004768,GO:0045300 functional_description:"Stearoyl-acyl carrier protein desaturase (AHRD V1 **** B7TZ44_SPIOL); contains Interpro domain(s) IPR005067 Fatty acid desaturase, type 2 " | 1E-48 |
| 592 | Contig 592 | 395 | 1 | GH205291 | Solyc11g005080.1.1 evidence_code:10F1H1E1IEG genomic_reference:SL2.40ch11 gene_region:67045-69072 transcript_region:SL2.40ch11:67045..69072+ functional_description:"Protein tolB (AHRD V1 *-*- D5MJZ0_9BACT); contains Interpro domain(s) IPR011042 Six-bladed beta-propeller, TolB-like " | 1E-77 |
| 593 | Contig 593 | 738 | 1 | GH203539 | Solyc03g115110.2.1 genomic_reference:SL2.40ch03 gene_region:58968179-58972603 transcript_region:SL2.40ch03:58968179..58972603- go_terms:GO:0005515 functional_description:"ATP synthase gamma chain (AHRD V1 ***- B9SZS3_RICCO); contains Interpro domain(s) IPR000131 ATPase, F1 complex, gamma subunit " | 1E-113 |
| 594 | Contig 594 | 634 | 1 | GH205257 | Solyc02g081500.2.1 genomic_reference:SL2.40ch02 gene_region:40016588-40018642 transcript_region:SL2.40ch02:40016588..40018642+ go_terms:GO:0006468 functional_description:"Receptor-like kinase (AHRD V1 ***- Q9LKY7_ORYSA); contains Interpro domain(s) IPR002290 Serine/threonine protein kinase " | 1E-111 |
| 595 | Contig 595 | 349 | 1 | GH205262 | Solyc08g008210.2.1 genomic_reference:SL2.40ch08 gene_region:2662300-2667303 transcript_region:SL2.40ch08:2662300..2667303- go_terms:GO:0005515,GO:0046961 functional_description:"V-type proton ATPase subunit E (AHRD V1 **** D3PGM0_9MAXI); contains Interpro domain(s) IPR002842 ATPase, V1/A1 complex, subunit E " | 5E-41 |
| 596 | Contig 596 | 287 | 1 | GH205228 | Solyc12g096650.1.1 evidence_code:10F1H1E1IEG genomic_reference:SL2.40ch12 gene_region:63815334-63820210 transcript_region:SL2.40ch12:63815334..63820210+ go_terms:GO:0015031 functional_description:"Charged multivesicular body protein 6 (AHRD V1 **-- B6TFV6_MAIZE); contains Interpro domain(s) IPR005024 Snf7 " | 3E-13 |
| 597 | Contig 597 | 265 | 1 | GH205256 | Solyc03g115950.2.1 genomic_reference:SL2.40ch03 gene_region:59579725-59580473 transcript_region:SL2.40ch03:59579725..59580473+ functional_description:"Unknown Protein (AHRD V1)" | 2E-14 |
| 598 | Contig 598 | 228 | 1 | GH203575 | Solyc07g044860.2.1 genomic_reference:SL2.40ch07 gene_region:55244985-55247025 transcript_region:SL2.40ch07:55244985..55247025- go_terms:GO:0008266 functional_description:"Oxygen-evolving enhancer protein 2, chloroplastic (AHRD V1 ***- PSBP_SOLLC); contains Interpro domain(s) IPR002683 Photosystem II oxygen evolving complex protein PsbP " | 3E-29 |
| 599 | Contig 599 | 332 | 1 | GH205225 | Solyc03g007740.2.1 genomic_reference:SL2.40ch03 gene_region:2271270-2273817 transcript_region:SL2.40ch03:2271270..2273817+ go_terms:GO:0005783 functional_description:"Reticulon family protein (AHRD V1 ***- B2WS91_9BRAS); contains Interpro domain(s) IPR003388 Reticulon " | 4E-43 |
| 600 | Contig 600 | 378 | 1 | GH203546 | ********* |  |
| 601 | Contig 601 | 192 | 1 | GH203647 | ********* |  |
| 602 | Contig 602 | 163 | 1 | GH205274 | Solyc07g044860.2.1 genomic_reference:SL2.40ch07 gene_region:55244985-55247025 transcript_region:SL2.40ch07:55244985..55247025- go_terms:GO:0008266 functional_description:"Oxygen-evolving enhancer protein 2, chloroplastic (AHRD V1 ***- PSBP_SOLLC); contains Interpro domain(s) IPR002683 Photosystem II oxygen evolving complex protein PsbP " | 2E-26 |
| 603 | Contig 603 | 296 | 1 | GH203599 | Solyc08g068070.2.1 genomic_reference:SL2.40ch08 gene_region:54369971-54375358 transcript_region:SL2.40ch08:54369971..54375358+ go_terms:GO:0005344 functional_description:"Globin (AHRD V1 ***- C8WRW6_ALIAD); contains Interpro domain(s) IPR001486 Globin, truncated bacterial-like " | 2E-46 |
| 604 | Contig 604 | 589 | 1 | GH203673 | Solyc01g100040.2.1 genomic_reference:SL2.40ch01 gene_region:81872312-81875388 transcript_region:SL2.40ch01:81872312..81875388- go_terms:GO:0004722 functional_description:"Integrin-linked kinase-associated serine/threonine phosphatase 2C (AHRD V1 **** ILKAP_HUMAN); contains Interpro domain(s) IPR015655 Protein phosphatase 2C " | 2E-87 |
| 605 | Contig 605 | 575 | 1 | GH205277 | Solyc02g081310.2.1 genomic_reference:SL2.40ch02 gene_region:39901020-39904709 transcript_region:SL2.40ch02:39901020..39904709- go_terms:GO:0005840,GO:0015934 functional_description:"50S ribosomal protein L15 (AHRD V1 ***- D0P1Y0_PHYIN); contains Interpro domain(s) IPR005749 Ribosomal protein L15, bacterial-type " | 3E-92 |
| 606 | Contig 606 | 182 | 1 | GH203567 | Solyc02g094470.2.1 genomic_reference:SL2.40ch02 gene_region:49555143-49558213 transcript_region:SL2.40ch02:49555143..49558213- go_terms:GO:0005315,GO:0015320 functional_description:"Mitochondrial phosphate carrier protein (AHRD V1 **** B9GFH9_POPTR); contains Interpro domain(s) IPR001993 Mitochondrial substrate carrier " | 3E-27 |
| 607 | Contig 607 | 577 | 1 | GH203657 | Solyc01g095610.2.1 genomic_reference:SL2.40ch01 gene_region:78581914-78585268 transcript_region:SL2.40ch01:78581914..78585268+ functional_description:"Leaf senescence protein-like (AHRD V1 ***- Q654T8_ORYSJ); contains Interpro domain(s) IPR004253 Protein of unknown function DUF231, plant " | 3E-90 |
| 608 | Contig 608 | 554 | 1 | GH203557 | Solyc12g013700.1.1 evidence_code:10F1H1E1IEG genomic_reference:SL2.40ch12 gene_region:4542236-4544315 transcript_region:SL2.40ch12:4542236..4544315+ functional_description:"Aluminum-induced protein-like protein (AHRD V1 ***- Q8S2R9_THEHA)" | 8E-86 |
| 609 | Contig 609 | 772 | 1 | GH203572 | Solyc01g090190.2.1 genomic_reference:SL2.40ch01 gene_region:75571683-75577506 transcript_region:SL2.40ch01:75571683..75577506- functional_description:"Nuclear RNA binding protein (AHRD V1 ***- C7TPG6_SOLTU); contains Interpro domain(s) IPR006861 Hyaluronan/mRNA binding protein " | 6E-58 |
| 610 | Contig 610 | 473 | 1 | GH205284 | Solyc06g049080.2.1 genomic_reference:SL2.40ch06 gene_region:28852597-28855492 transcript_region:SL2.40ch06:28852597..28855492+ go_terms:GO:0004784 functional_description:"Superoxide dismutase (AHRD V1 **** A5BR41_VITVI); contains Interpro domain(s) IPR001189 Manganese/iron superoxide dismutase " | 2E-88 |
| 611 | Contig 611 | 346 | 1 | GH203581 | ********* |  |
| 612 | Contig 612 | 133 | 1 | GH205227 | Solyc08g066750.2.1 genomic_reference:SL2.40ch08 gene_region:52771035-52775069 transcript_region:SL2.40ch08:52771035..52775069- functional_description:"Major facilitator superfamily domain containing protein 5 (AHRD V1 ***- B2W8V3_PYRTR); contains Interpro domain(s) IPR008509 Protein of unknown function DUF791 " | 1E-20 |
| 613 | Contig 613 | 513 | 1 | GH203553 | Solyc07g043420.2.1 genomic_reference:SL2.40ch07 gene_region:54503345-54505070 transcript_region:SL2.40ch07:54503345..54505070+ go_terms:GO:0010302,GO:0045431 functional_description:"2-oxoglutarate-dependent dioxygenase (AHRD V1 **-* Q9ZSH4_SOLCH); contains Interpro domain(s) IPR005123 Oxoglutarate and iron-dependent oxygenase " | 1E-98 |
| 614 | Contig 614 | 206 | 1 | GH203585 | Solyc07g063960.2.1 genomic_reference:SL2.40ch07 gene_region:63515688-63518536 transcript_region:SL2.40ch07:63515688..63518536+ go_terms:GO:0005840 functional_description:"50S ribosomal protein L24 (AHRD V1 ***- B4FTJ3_MAIZE); contains Interpro domain(s) IPR003256 Ribosomal protein L24 " | 9E-19 |
| 615 | Contig 615 | 646 | 1 | GH203649 | Solyc01g028810.2.1 genomic_reference:SL2.40ch01 gene_region:33327242-33332361 transcript_region:SL2.40ch01:33327242..33332361- go_terms:GO:0044267,GO:0005737 functional_description:"chaperonin (AHRD V1 ***- B4VMY1_9CYAN); contains Interpro domain(s) IPR001844 Chaperonin Cpn60 " | 2E-75 |
| 616 | Contig 616 | 599 | 1 | GH203600 | Solyc03g005050.2.1 genomic_reference:SL2.40ch03 gene_region:44287-46883 transcript_region:SL2.40ch03:44287..46883+ go_terms:GO:0006139 functional_description:"Adenylate kinase family-like protein (AHRD V1 ***- Q38JG0_SOLTU); contains Interpro domain(s) IPR000850 Adenylate kinase " | 1E-101 |
| 617 | Contig 617 | 665 | 1 | GH203580 | Solyc02g065580.2.1 genomic_reference:SL2.40ch02 gene_region:31319010-31323703 transcript_region:SL2.40ch02:31319010..31323703- go_terms:GO:0000293 functional_description:"Cytochrome b561 (AHRD V1 ***- Q3LGX4_CITLA); contains Interpro domain(s) IPR004877 Cytochrome b561, eukaryote " | 2E-78 |
| 618 | Contig 618 | 586 | 1 | GH203651 | Solyc04g071970.2.1 genomic_reference:SL2.40ch04 gene_region:56571411-56574425 transcript_region:SL2.40ch04:56571411..56574425- functional_description:"Light stress-responsive one-helix protein-like (AHRD V1 ***- Q5ZC72_ORYSJ)" | 2E-44 |
| 619 | Contig 619 | 772 | 1 | GH203650 | Solyc01g103450.2.1 genomic_reference:SL2.40ch01 gene_region:83821528-83826037 transcript_region:SL2.40ch01:83821528..83826037+ go_terms:GO:0005524,GO:0006457 functional_description:"Chaperone DnaK (AHRD V1 ***- Q1SKX2_MEDTR); contains Interpro domain(s) IPR012725 Chaperone DnaK " | 1E-133 |
| 620 | Contig 620 | 646 | 1 | GH203562 | Solyc09g008130.2.1 genomic_reference:SL2.40ch09 gene_region:1600648-1607197 transcript_region:SL2.40ch09:1600648..1607197- go_terms:GO:0006096,GO:0006400 functional_description:"Phosphoglycerate kinase (AHRD V1 ***- B9IFP7_POPTR); contains Interpro domain(s) IPR001576 Phosphoglycerate kinase " | 1E-117 |
| 621 | Contig 621 | 640 | 1 | GH205283 | Solyc04g063290.2.1 genomic_reference:SL2.40ch04 gene_region:54541016-54543059 transcript_region:SL2.40ch04:54541016..54543059- go_terms:GO:0003735 functional_description:"30S ribosomal protein S5 (AHRD V1 ***- C5K1T7_AJEDS); contains Interpro domain(s) IPR005711 Ribosomal protein S5, eukaryotic/archaeal " | 8E-94 |
| 622 | Contig 622 | 367 | 1 | GH204309 | Solyc06g073280.2.1 genomic_reference:SL2.40ch06 gene_region:41539950-41545003 transcript_region:SL2.40ch06:41539950..41545003+ go_terms:GO:0010285,GO:0005507 functional_description:"LL-diaminopimelate aminotransferase (AHRD V1 **** A8IW39_CHLRE); contains Interpro domain(s) IPR019942 LL-diaminopimelate aminotransferase, plant-related " | 1E-67 |
| 623 | Contig 623 | 251 | 1 | GH204313 | ********* |  |
| 624 | Contig 624 | 901 | 1 | GH203605 | Solyc09g075140.2.1 genomic_reference:SL2.40ch09 gene_region:62435204-62439921 transcript_region:SL2.40ch09:62435204..62439921- go_terms:GO:0008289,GO:0004622 functional_description:"Lipase-like protein (AHRD V1 **-- Q9M3D1_ARATH)" | 3E-23 |
| 625 | Contig 625 | 764 | 1 | GH204319 | Solyc07g032380.2.1 genomic_reference:SL2.40ch07 gene_region:37904500-37908632 transcript_region:SL2.40ch07:37904500..37908632- functional_description:"Pentatricopeptide repeat-containing protein (AHRD V1 ***- D7M4J8_ARALY); contains Interpro domain(s) IPR002885 Pentatricopeptide repeat " | 1E-115 |
| 626 | Contig 626 | 472 | 1 | GH204341 | Solyc11g065670.1.1 evidence_code:10F1H1E1IEG genomic_reference:SL2.40ch11 gene_region:48297370-48297870 transcript_region:SL2.40ch11:48297370..48297870- go_terms:GO:0005840 functional_description:"Ribosomal protein L12 (AHRD V1 ***- A8J597_CHLRE); contains Interpro domain(s) IPR000911 Ribosomal protein L11 " | 3E-46 |
| 627 | Contig 627 | 185 | 1 | GH204329 | Solyc06g073280.2.1 genomic_reference:SL2.40ch06 gene_region:41539950-41545003 transcript_region:SL2.40ch06:41539950..41545003+ go_terms:GO:0010285,GO:0005507 functional_description:"LL-diaminopimelate aminotransferase (AHRD V1 **** A8IW39_CHLRE); contains Interpro domain(s) IPR019942 LL-diaminopimelate aminotransferase, plant-related " | 1E-31 |
| 628 | Contig 628 | 191 | 1 | GH204270 | ********* |  |
| 629 | Contig 629 | 286 | 1 | GH204723 | Solyc07g066310.2.1 genomic_reference:SL2.40ch07 gene_region:65009418-65011119 transcript_region:SL2.40ch07:65009418..65011119+ go_terms:GO:0042651 functional_description:"photosystem II polypeptide (AHRD V1 ***- Q6V7X5_TRIPR); contains Interpro domain(s) IPR006814 Photosystem II protein PsbR " | 5E-36 |
| 630 | Contig 630 | 414 | 1 | GH203566 | Solyc03g098220.2.1 genomic_reference:SL2.40ch03 gene_region:54022399-54028329 transcript_region:SL2.40ch03:54022399..54028329- go_terms:GO:0004758 functional_description:"Serine palmitoyltransferase (AHRD V1 **-* B3Y000_NICBE); contains Interpro domain(s) IPR004839 Aminotransferase, class I and II " | 9E-68 |
| 631 | Contig 631 | 408 | 1 | GH204281 | ********* |  |
| 632 | Contig 632 | 444 | 1 | GH204302 | Solyc03g044330.1.1 evidence_code:10F0H1E1IEG genomic_reference:SL2.40ch03 gene_region:12823169-12825148 transcript_region:SL2.40ch03:12823169..12825148- go_terms:GO:0003984 functional_description:"Acetolactate synthase (AHRD V1 **** D7RPJ7_SOLTU); contains Interpro domain(s) IPR012846 Acetolactate synthase, large subunit, biosynthetic " | 9E-81 |
| 633 | Contig 633 | 317 | 1 | GH204831 | Solyc09g091660.2.1 genomic_reference:SL2.40ch09 gene_region:66255162-66263653 transcript_region:SL2.40ch09:66255162..66263653+ go_terms:GO:0042626 functional_description:"ABC transporter G family member 40 (AHRD V1 ***- AB40G_ARATH); contains Interpro domain(s) IPR013525 ABC-2 type transporter " | 3E-56 |
| 634 | Contig 634 | 707 | 1 | GH204827 | Solyc08g079090.2.1 genomic_reference:SL2.40ch08 gene_region:59901779-59905784 transcript_region:SL2.40ch08:59901779..59905784+ go_terms:GO:0055114 functional_description:"Laccase-22 (AHRD V1 **-- LAC22_ORYSJ); contains Interpro domain(s) IPR001117 Multicopper oxidase, type 1 " | 1E-126 |
| 635 | Contig 635 | 502 | 1 | GH204290 | Solyc07g065840.2.1 genomic_reference:SL2.40ch07 gene_region:64658676-64662396 transcript_region:SL2.40ch07:64658676..64662396- go_terms:GO:0042623 functional_description:"Heat shock protein 90 (AHRD V1 ***- Q14TB1_TOBAC); contains Interpro domain(s) IPR003594 ATP-binding region, ATPase-like IPR001404 Heat shock protein Hsp90 " | 1E-87 |
| 636 | Contig 636 | 602 | 1 | GH204296 | Solyc05g056140.2.1 genomic_reference:SL2.40ch05 gene_region:64680558-64685370 transcript_region:SL2.40ch05:64680558..64685370- go_terms:GO:0004842 functional_description:"Inter-alpha-trypsin inhibitor heavy chain H3 (AHRD V1 *--- ITIH3_MESAU); contains Interpro domain(s) IPR002035 von Willebrand factor, type A " | 6E-69 |
| 637 | Contig 637 | 677 | 1 | GH204268 | Solyc07g064910.2.1 genomic_reference:SL2.40ch07 gene_region:64106940-64112207 transcript_region:SL2.40ch07:64106940..64112207+ go_terms:GO:0019904 functional_description:"EH-domain-containing protein 1 (AHRD V1 **** B6U193_MAIZE); contains Interpro domain(s) IPR001401 Dynamin, GTPase region " | 7E-91 |
| 638 | Contig 638 | 498 | 1 | GH204292 | Solyc02g077730.2.1 genomic_reference:SL2.40ch02 gene_region:37185838-37188452 transcript_region:SL2.40ch02:37185838..37188452+ functional_description:"Unknown Protein (AHRD V1)" | 4E-14 |
| 639 | Contig 639 | 273 | 1 | GH204834 | Solyc08g080660.1.1 evidence_code:10F1H1E1IEG genomic_reference:SL2.40ch08 gene_region:61053978-61054730 transcript_region:SL2.40ch08:61053978..61054730- go_terms:GO:0005515 functional_description:"Osmotin-like protein (Fragment) (AHRD V1 **-- Q8S4L1_SOLNI); contains Interpro domain(s) IPR001938 Thaumatin, pathogenesis-related " | 3E-55 |
| 640 | Contig 640 | 438 | 1 | GH204851 | Solyc03g117480.2.1 genomic_reference:SL2.40ch03 gene_region:60677249-60682108 transcript_region:SL2.40ch03:60677249..60682108- go_terms:GO:0009678 functional_description:"Pyrophosphate-energized proton pump (Pyrophosphate-energized inorganic pyrophosphatase) (H(+)-PPase) (AHRD V1 **** C7HW54_9FIRM); contains Interpro domain(s) IPR004131 Inorganic H+ pyrophosphatase " | 5E-33 |
| 641 | Contig 641 | 565 | 1 | GH204295 | Solyc10g083520.1.1 evidence_code:10F1H1E1IEG genomic_reference:SL2.40ch10 gene_region:62639481-62640898 transcript_region:SL2.40ch10:62639481..62640898+ functional_description:"cDNA clone J033118E13 full insert sequence (AHRD V1 **-- B7EU28_ORYSJ); contains Interpro domain(s) IPR007033 Protein of unknown function DUF662 " | 3E-29 |
| 642 | Contig 642 | 525 | 1 | GH204679 | Solyc03g120090.1.1 evidence_code:10F1H1E1IEG genomic_reference:SL2.40ch03 gene_region:62641034-62641951 transcript_region:SL2.40ch03:62641034..62641951+ go_terms:GO:0046982,GO:0042803 functional_description:"Pyridoxal biosynthesis lyase pdxS (AHRD V1 ***- Q2RMJ0_MOOTA); contains Interpro domain(s) IPR001852 Vitamin B6 biosynthesis protein " | 8E-59 |
| 643 | Contig 643 | 327 | 1 | GH204719 | ********* |  |
| 644 | Contig 644 | 669 | 1 | GH204721 | Solyc06g065470.2.1 genomic_reference:SL2.40ch06 gene_region:37255674-37260503 transcript_region:SL2.40ch06:37255674..37260503- go_terms:GO:0005515 functional_description:"RNA-binding protein 8A (AHRD V1 *--* B6TR37_MAIZE); contains Interpro domain(s) IPR012677 Nucleotide-binding, alpha-beta plait " | 7E-75 |
| 645 | Contig 645 | 658 | 1 | GH205266 | Solyc03g112150.1.1 evidence_code:10F1H1E1IEG genomic_reference:SL2.40ch03 gene_region:56698575-56700008 transcript_region:SL2.40ch03:56698575..56700008+ go_terms:GO:0003746 functional_description:"Elongation factor Tu (AHRD V1 **** D7MFK2_ARALY); contains Interpro domain(s) IPR004541 Translation elongation factor EFTu/EF1A, bacterial and organelle " | 3E-94 |
| 646 | Contig 646 | 336 | 1 | GH205013 | Solyc04g082140.2.1 genomic_reference:SL2.40ch04 gene_region:63511091-63515964 transcript_region:SL2.40ch04:63511091..63515964- go_terms:GO:0055114 functional_description:"Laccase-22 (AHRD V1 **-- LAC22_ORYSJ); contains Interpro domain(s) IPR001117 Multicopper oxidase, type 1 " | 1E-59 |
| 647 | Contig 647 | 302 | 1 | GH204659 | Solyc08g077530.2.1 genomic_reference:SL2.40ch08 gene_region:58684261-58687653 transcript_region:SL2.40ch08:58684261..58687653+ go_terms:GO:0016161 functional_description:"Beta-amylase (AHRD V1 **** Q5F305_SOYBN); contains Interpro domain(s) IPR001371 Glycoside hydrolase, family 14B, plant " | 1E-57 |
| 648 | Contig 648 | 677 | 1 | GH203573 | Solyc03g115200.2.1 genomic_reference:SL2.40ch03 gene_region:59049950-59054055 transcript_region:SL2.40ch03:59049950..59054055- functional_description:"Glucan endo-1 3-beta-glucosidase 1 (AHRD V1 *-*- B6TW10_MAIZE); contains Interpro domain(s) IPR012946 X8 " | 1E-64 |
| 649 | Contig 649 | 526 | 1 | GH204274 | Solyc10g079930.1.1 evidence_code:10F1H1E1IEG genomic_reference:SL2.40ch10 gene_region:60702629-60703942 transcript_region:SL2.40ch10:60702629..60703942- go_terms:GO:0008152 functional_description:"UDP-glucosyltransferase HvUGT5876 (AHRD V1 ***- D3WYW1_HORVD); contains Interpro domain(s) IPR002213 UDP-glucuronosyl/UDP-glucosyltransferase " | 1E-101 |
| 650 | Contig 650 | 696 | 1 | GH204273 | Solyc08g081410.2.1 genomic_reference:SL2.40ch08 gene_region:61641833-61651949 transcript_region:SL2.40ch08:61641833..61651949+ go_terms:GO:0016020,GO:0005488 functional_description:"General vesicular transport factor p115 (AHRD V1 *--- B0WXD3_CULQU); contains Interpro domain(s) IPR006955 Uso1/p115 like vesicle tethering protein, C-terminal " | 1E-108 |
| 651 | Contig 651 | 453 | 1 | GH205264 | Solyc01g088020.2.1 genomic_reference:SL2.40ch01 gene_region:74630101-74643687 transcript_region:SL2.40ch01:74630101..74643687+ go_terms:GO:0048306 functional_description:"Protein transport protein sec31 (AHRD V1 **-- C8V1I6_EMENI); contains Interpro domain(s) IPR017986 WD40 repeat, region " | 4E-65 |
| 652 | Contig 652 | 569 | 1 | GH204859 | Solyc03g121590.2.1 genomic_reference:SL2.40ch03 gene_region:63727316-63737352 transcript_region:SL2.40ch03:63727316..63737352+ go_terms:GO:0031177 functional_description:"Protein SEY1 (AHRD V1 ***- B6U4D9_MAIZE); contains Interpro domain(s) IPR008803 Root hair defective 3 GTP-binding " | 1E-105 |
| 653 | Contig 653 | 460 | 1 | GH204862 | ********* |  |
| 654 | Contig 654 | 147 | 1 | GH203578 | Solyc09g074930.2.1 genomic_reference:SL2.40ch09 gene_region:62225793-62227794 transcript_region:SL2.40ch09:62225793..62227794+ functional_description:"REF-like stress related protein 1 (AHRD V1 *--- Q6XNP4_HEVBR); contains Interpro domain(s) IPR008802 Rubber elongation factor " | 7E-22 |
| 655 | Contig 655 | 404 | 1 | GH204860 | ********* |  |
| 656 | Contig 656 | 351 | 1 | GH204861 | Solyc02g091560.2.1 genomic_reference:SL2.40ch02 gene_region:47400296-47406791 transcript_region:SL2.40ch02:47400296..47406791+ go_terms:GO:0004372 functional_description:"Serine hydroxymethyltransferase (AHRD V1 **** C6ZJZ0_SOYBN); contains Interpro domain(s) IPR001085 Serine hydroxymethyltransferase " | 2E-47 |
| 657 | Contig 657 | 166 | 1 | GH205273 | ********* |  |
| 658 | Contig 658 | 251 | 1 | GH204280 | Solyc06g063370.2.1 genomic_reference:SL2.40ch06 gene_region:36437291-36439451 transcript_region:SL2.40ch06:36437291..36439451- go_terms:GO:0016020 functional_description:"Chlorophyll a-b binding protein 1A, chloroplastic (AHRD V1 ***- CB2A_PYRPY); contains Interpro domain(s) IPR001344 Chlorophyll A-B binding protein " | 1E-43 |
| 659 | Contig 659 | 179 | 1 | GH204609 | ********* |  |
| 660 | Contig 660 | 419 | 1 | GH204744 | Solyc02g080530.2.1 genomic_reference:SL2.40ch02 gene_region:39317455-39319147 transcript_region:SL2.40ch02:39317455..39319147- go_terms:GO:0004601,GO:0005515 functional_description:"Peroxidase (AHRD V1 **** C7DYB2_9ERIC); contains Interpro domain(s) IPR002016 Haem peroxidase, plant/fungal/bacterial " | 6E-46 |
| 661 | Contig 661 | 666 | 1 | GH204279 | Solyc11g069090.1.1 evidence_code:10F0H1E1IEG genomic_reference:SL2.40ch11 gene_region:50759436-50762771 transcript_region:SL2.40ch11:50759436..50762771+ go_terms:GO:0017111,GO:0016887 functional_description:"ATP-binding cassette protein (AHRD V1 ***- C0NDN3_AJECG); contains Interpro domain(s) IPR003439 ABC transporter-like " | 4E-38 |
| 662 | Contig 662 | 499 | 1 | GH204278 | Solyc02g087930.2.1 genomic_reference:SL2.40ch02 gene_region:44752863-44756574 transcript_region:SL2.40ch02:44752863..44756574- go_terms:GO:0003735,GO:0005515 functional_description:"60S ribosomal protein L34 (AHRD V1 ***- B6T098_MAIZE); contains Interpro domain(s) IPR008195 Ribosomal protein L34e " | 6E-50 |
| 663 | Contig 663 | 494 | 1 | GH204847 | Solyc04g081440.2.1 genomic_reference:SL2.40ch04 gene_region:63008173-63012837 transcript_region:SL2.40ch04:63008173..63012837- go_terms:GO:0005515,GO:0004564 functional_description:"Neutral invertase like protein (AHRD V1 ***- Q67XD9_ARATH); contains Interpro domain(s) IPR006937 Plant neutral invertase " | 5E-69 |
| 664 | Contig 664 | 481 | 1 | GH204652 | Solyc11g066160.1.1 evidence_code:10F1H1E1IEG genomic_reference:SL2.40ch11 gene_region:48939641-48939952 transcript_region:SL2.40ch11:48939641..48939952+ go_terms:GO:0043565 functional_description:"Histone H4 (AHRD V1 ***- B6T0P4_MAIZE); contains Interpro domain(s) IPR001951 Histone H4 " | 3E-40 |
| 665 | Contig 665 | 208 | 1 | GH204952 | Solyc07g042250.2.1 genomic_reference:SL2.40ch07 gene_region:52684617-52687267 transcript_region:SL2.40ch07:52684617..52687267+ go_terms:GO:0051087 functional_description:"chaperonin (AHRD V1 *-*- B4WMS9_9SYNE); contains Interpro domain(s) IPR017416 Chaperonin 21, chloroplast IPR018369 Chaperonin Cpn10, conserved site " | 3E-32 |
| 666 | Contig 666 | 653 | 1 | GH204916 | Solyc01g111520.2.1 genomic_reference:SL2.40ch01 gene_region:89504374-89512877 transcript_region:SL2.40ch01:89504374..89512877- go_terms:GO:0005509 functional_description:"Synaptotagmin (AHRD V1 *--- B0W3L1_CULQU); contains Interpro domain(s) IPR018029 C2 membrane targeting protein " | 1E-115 |
| 667 | Contig 667 | 432 | 1 | GH204184 | Solyc09g072770.1.1 evidence_code:10F0H0E1IEG genomic_reference:SL2.40ch09 gene_region:60943399-60944010 transcript_region:SL2.40ch09:60943399..60944010+ functional_description:"mRNA clone RAFL22-93-M12 (Fragment) (AHRD V1 *-*- Q67ZI7_ARATH)" | 4E-28 |
| 668 | Contig 668 | 440 | 1 | GH204159 | Solyc09g092260.2.1 genomic_reference:SL2.40ch09 gene_region:66746507-66748076 transcript_region:SL2.40ch09:66746507..66748076+ go_terms:GO:0031072 functional_description:"Chaperone protein dnaJ 20 (AHRD V1 ***- B6U349_MAIZE); contains Interpro domain(s) IPR001623 Heat shock protein DnaJ, N-terminal " | 1E-59 |
| 669 | Contig 669 | 568 | 1 | GH204131 | Solyc03g079940.2.1 genomic_reference:SL2.40ch03 gene_region:45400646-45404642 transcript_region:SL2.40ch03:45400646..45404642- go_terms:GO:0015450 functional_description:"Mitochondrial import inner membrane translocase (AHRD V1 **-- C1FD57_9CHLO); contains Interpro domain(s) IPR001660 Sterile alpha motif SAM " | 1E-86 |
| 670 | Contig 670 | 355 | 1 | GH204151 | Solyc02g089610.1.1 evidence_code:10F0H1E1IEG genomic_reference:SL2.40ch02 gene_region:45946477-45947565 transcript_region:SL2.40ch02:45946477..45947565- go_terms:GO:0004014 functional_description:"S-adenosylmethionine decarboxylase proenzyme (AHRD V1 **** Q7XZQ9_VITVI); contains Interpro domain(s) IPR001985 S-adenosylmethionine decarboxylase " | 1E-60 |
| 671 | Contig 671 | 481 | 1 | GH204153 | Solyc12g011340.1.1 evidence_code:10F0H1E1IEG genomic_reference:SL2.40ch12 gene_region:4183346-4190442 transcript_region:SL2.40ch12:4183346..4190442+ go_terms:GO:0003676,GO:0000166 functional_description:"Polyadenylate-binding protein (AHRD V1 *-*- D3TN92_GLOMM); contains Interpro domain(s) IPR000504 RNA recognition motif, RNP-1 " | 6E-43 |
| 672 | Contig 672 | 566 | 1 | GH204141 | Solyc09g090430.2.1 genomic_reference:SL2.40ch09 gene_region:65298327-65302769 transcript_region:SL2.40ch09:65298327..65302769- go_terms:GO:0008152,GO:0003677,GO:0009439 functional_description:"Cyanate hydratase (AHRD V1 ***- B6TTW1_MAIZE); contains Interpro domain(s) IPR008076 Cyanase " | 7E-74 |
| 673 | Contig 673 | 573 | 1 | GH204201 | Solyc06g063310.1.1 evidence_code:10F1H1E1IEG genomic_reference:SL2.40ch06 gene_region:36402097-36402360 transcript_region:SL2.40ch06:36402097..36402360- functional_description:"Fiber protein Fb15 (Fragment) (AHRD V1 ***- B4UW72_ARAHY)" | 1E-30 |
| 674 | Contig 674 | 245 | 1 | GH204190 | Solyc11g068450.1.1 evidence_code:10F0H1E0IEG genomic_reference:SL2.40ch11 gene_region:50260524-50262870 transcript_region:SL2.40ch11:50260524..50262870+ functional_description:"Biogenesis of lysosome-related organelles complex-1 subunit 1 (AHRD V1 ***- C3KJU5_ANOFI); contains Interpro domain(s) IPR009395 GCN5-like 1 " | 2E-31 |
| 675 | Contig 675 | 797 | 1 | GH204203 | Solyc10g076290.1.1 evidence_code:10F0H1E1IEG genomic_reference:SL2.40ch10 gene_region:58549205-58554411 transcript_region:SL2.40ch10:58549205..58554411+ go_terms:GO:0005515,GO:0004842 functional_description:"F-box/LRR-repeat protein 3 (AHRD V1 ***- FBL3_ARATH); contains Interpro domain(s) IPR006553 Leucine-rich repeat, cysteine-containing subtype " | 1E-123 |
| 676 | Contig 676 | 618 | 1 | GH204185 | Solyc03g044200.2.1 genomic_reference:SL2.40ch03 gene_region:12420327-12423488 transcript_region:SL2.40ch03:12420327..12423488+ go_terms:GO:0004024 functional_description:"Alcohol dehydrogenase (AHRD V1 **** Q43169_SOLTU); contains Interpro domain(s) IPR002085 Alcohol dehydrogenase superfamily, zinc-containing " | 1E-117 |
| 677 | Contig 677 | 292 | 1 | GH204191 | ********* |  |
| 678 | Contig 678 | 567 | 1 | GH204711 | Solyc03g111720.2.1 genomic_reference:SL2.40ch03 gene_region:56431489-56432545 transcript_region:SL2.40ch03:56431489..56432545+ go_terms:GO:0008113 functional_description:"Peptide methionine sulfoxide reductase msrA (AHRD V1 **** D7E9W5_METEZ); contains Interpro domain(s) IPR002569 Methionine sulphoxide reductase A " | 2E-80 |
| 679 | Contig 679 | 672 | 1 | GH204655 | Solyc05g006870.2.1 genomic_reference:SL2.40ch05 gene_region:1484874-1486270 transcript_region:SL2.40ch05:1484874..1486270- go_terms:GO:0032403 functional_description:"Thioredoxin H (AHRD V1 ***- Q4U0W0_NICAL); contains Interpro domain(s) IPR015467 Thioredoxin, core " | 3E-37 |
| 680 | Contig 680 | 578 | 1 | GH204524 | Solyc01g009850.2.1 genomic_reference:SL2.40ch01 gene_region:4369328-4375850 transcript_region:SL2.40ch01:4369328..4375850+ functional_description:"Unknown Protein (AHRD V1)" | 1E-16 |
| 681 | Contig 681 | 205 | 1 | GH204206 | Solyc02g083340.2.1 genomic_reference:SL2.40ch02 gene_region:41357264-41365434 transcript_region:SL2.40ch02:41357264..41365434- go_terms:GO:0004630 functional_description:"Phospholipase D (AHRD V1 **** Q6AVR2_ORYSJ); contains Interpro domain(s) IPR011402 Phospholipase D, plant " | 3E-23 |
| 682 | Contig 682 | 456 | 1 | GH204523 | Solyc10g078630.1.1 evidence_code:10F1H0E1IEG genomic_reference:SL2.40ch10 gene_region:59705341-59705538 transcript_region:SL2.40ch10:59705341..59705538- go_terms:GO:0005840 functional_description:"40S ribosomal protein S28 (AHRD V1 ***- B6T1V2_MAIZE); contains Interpro domain(s) IPR000289 Ribosomal protein S28e " | 1E-14 |
| 683 | Contig 683 | 617 | 1 | GH204147 | Solyc08g042050.2.1 genomic_reference:SL2.40ch08 gene_region:28924706-28934844 transcript_region:SL2.40ch08:28924706..28934844+ go_terms:GO:0004004,GO:0017151 functional_description:"DEAD-box ATP-dependent RNA helicase 3 (AHRD V1 **** RH3_ORYSJ); contains Interpro domain(s) IPR011545 DNA/RNA helicase, DEAD/DEAH box type, N-terminal " | 3E-66 |
| 684 | Contig 684 | 395 | 1 | GH203977 | Solyc02g071120.2.1 genomic_reference:SL2.40ch02 gene_region:35176956-35184955 transcript_region:SL2.40ch02:35176956..35184955+ go_terms:GO:0016413 functional_description:"CAS1 domain containing 1 (AHRD V1 **-- B0W8N6_CULQU); contains Interpro domain(s) IPR012419 Cas1p-like " | 6E-13 |
| 685 | Contig 685 | 693 | 1 | GH205063 | Solyc12g009810.1.1 evidence_code:10F0H1E1IEG genomic_reference:SL2.40ch12 gene_region:3011434-3014084 transcript_region:SL2.40ch12:3011434..3014084- go_terms:GO:0030259 functional_description:"UDP-N-acetylglucosamine transferase subunit alg13 (AHRD V1 **-- C1BGJ4_ONCMY); contains Interpro domain(s) IPR007235 Glycosyl transferase, family 28, C-terminal " | 6E-98 |
| 686 | Contig 686 | 466 | 1 | GH203987 | Solyc12g089310.1.1 evidence_code:10F0H1E0IEG genomic_reference:SL2.40ch12 gene_region:62884778-62887198 transcript_region:SL2.40ch12:62884778..62887198- go_terms:GO:0005200 functional_description:"Tubulin beta-1 chain (AHRD V1 ***- D7KT68_ARALY); contains Interpro domain(s) IPR002453 Beta tubulin " | 2E-52 |
| 687 | Contig 687 | 228 | 1 | GH204541 | Solyc01g100200.2.1 genomic_reference:SL2.40ch01 gene_region:82020197-82022661 transcript_region:SL2.40ch01:82020197..82022661- go_terms:GO:0003700 functional_description:"GRAS family transcription factor (AHRD V1 **-* B9GJL6_POPTR); contains Interpro domain(s) IPR005202 GRAS transcription factor " | 9E-19 |
| 688 | Contig 688 | 143 | 1 | GH203985 | ********* |  |
| 689 | Contig 689 | 283 | 1 | GH204002 | ********* |  |
| 690 | Contig 690 | 677 | 1 | GH204586 | Solyc02g085780.2.1 genomic_reference:SL2.40ch02 gene_region:43134602-43139924 transcript_region:SL2.40ch02:43134602..43139924- go_terms:GO:0008536 functional_description:"Regulator of chromosome condensation (RCC1)-like protein (AHRD V1 **-- D0NEM9_PHYIN); contains Interpro domain(s) IPR009091 Regulator of chromosome condensation/beta-lactamase-inhibitor protein II " | 3E-73 |
| 691 | Contig 691 | 438 | 1 | GH203979 | Solyc09g075360.2.1 genomic_reference:SL2.40ch09 gene_region:62584484-62587919 transcript_region:SL2.40ch09:62584484..62587919+ go_terms:GO:0008810 functional_description:"Endoglucanase 1 (AHRD V1 ***- B6U0P7_MAIZE); contains Interpro domain(s) IPR008928 Six-hairpin glycosidase-like IPR012341 Six-hairpin glycosidase IPR018221 Glycoside hydrolase, family 9, active site IPR001701 Glycoside hydrolase, family 9 " | 9E-83 |
| 692 | Contig 692 | 813 | 1 | GH205044 | Solyc09g075370.2.1 genomic_reference:SL2.40ch09 gene_region:62589136-62593044 transcript_region:SL2.40ch09:62589136..62593044+ functional_description:"Mitochondrial ribosomal protein L46 (AHRD V1 ***- B0X635_CULQU)" | 8E-71 |
| 693 | Contig 693 | 493 | 1 | GH204625 | Solyc02g064730.2.1 genomic_reference:SL2.40ch02 gene_region:30412691-30418734 transcript_region:SL2.40ch02:30412691..30418734- functional_description:"Expressed protein having alternate splicing products (AHRD V1 *--- Q75KB8_ORYSJ); contains Interpro domain(s) IPR015023 Protein of unknown function DUF1909 " | 2E-41 |
| 694 | Contig 694 | 135 | 1 | GH204707 | Solyc10g085000.1.1 evidence_code:10F0H1E1IEG genomic_reference:SL2.40ch10 gene_region:63669940-63674284 transcript_region:SL2.40ch10:63669940..63674284+ go_terms:GO:0006468,GO:0005488 functional_description:"Receptor-like kinase (AHRD V1 ***- C0SW32_SOYBN); contains Interpro domain(s) IPR002290 Serine/threonine protein kinase " | 4E-20 |
| 695 | Contig 695 | 278 | 1 | GH204935 | Solyc05g051290.2.1 genomic_reference:SL2.40ch05 gene_region:60727691-60729288 transcript_region:SL2.40ch05:60727691..60729288- go_terms:GO:0005634,GO:0003677 functional_description:"High mobility group family (AHRD V1 *--- B9H3A3_POPTR); contains Interpro domain(s) IPR017956 AT hook, DNA-binding, conserved site " | 3E-12 |
| 696 | Contig 696 | 227 | 1 | GH204176 | Solyc12g009800.1.1 evidence_code:10F0H1E1IEG genomic_reference:SL2.40ch12 gene_region:3005117-3008886 transcript_region:SL2.40ch12:3005117..3008886- go_terms:GO:0016787,GO:0046872 functional_description:"Purple acid phosphatase 3 (AHRD V1 ***- Q6J5M8_SOLTU); contains Interpro domain(s) IPR015914 Purple acid phosphatase, N-terminal " | 4E-41 |
| 697 | Contig 697 | 226 | 1 | GH204174 | ********* |  |
| 698 | Contig 698 | 232 | 1 | GH204169 | Solyc02g030170.2.1 genomic_reference:SL2.40ch02 gene_region:15953224-15955598 transcript_region:SL2.40ch02:15953224..15955598- go_terms:GO:0042605,GO:0019899 functional_description:"FAD linked oxidase domain protein (AHRD V1 **-- D1VPW7_9ACTO); contains Interpro domain(s) IPR006094 FAD linked oxidase, N-terminal " | 1E-40 |
| 699 | Contig 699 | 192 | 1 | GH204171 | Solyc09g011880.2.1 genomic_reference:SL2.40ch09 gene_region:5155740-5160598 transcript_region:SL2.40ch09:5155740..5160598- functional_description:"LOC556397 protein (Fragment) (AHRD V1 **-- Q05AJ0_DANRE)" | 9E-33 |
| 700 | Contig 700 | 762 | 1 | GH205057 | Solyc02g069090.2.1 genomic_reference:SL2.40ch02 gene_region:33631872-33638685 transcript_region:SL2.40ch02:33631872..33638685- go_terms:GO:0030984 functional_description:"Cathepsin B (AHRD V1 ***- Q1HER6_NICBE); contains Interpro domain(s) IPR015643 Peptidase C1A, cathepsin B " | 1E-121 |
| 701 | Contig 701 | 147 | 1 | GH204947 | Solyc11g017200.1.1 evidence_code:10F0H1E1IEG genomic_reference:SL2.40ch11 gene_region:8016371-8020103 transcript_region:SL2.40ch11:8016371..8020103- go_terms:GO:0005488 functional_description:"TPR repeat (AHRD V1 *--- Q31NR5_SYNE7); contains Interpro domain(s) IPR011990 Tetratricopeptide-like helical " | 1E-22 |
| 702 | Contig 702 | 705 | 1 | GH204940 | Solyc01g111980.2.1 genomic_reference:SL2.40ch01 gene_region:89876788-89879583 transcript_region:SL2.40ch01:89876788..89879583- go_terms:GO:0015171 functional_description:"Lysine/histidine transporter (AHRD V1 **** B9HR02_POPTR); contains Interpro domain(s) IPR013057 Amino acid transporter, transmembrane " | 1E-128 |
| 703 | Contig 703 | 326 | 1 | GH204120 | Solyc07g008880.2.1 genomic_reference:SL2.40ch07 gene_region:3845794-3858565 transcript_region:SL2.40ch07:3845794..3858565+ go_terms:GO:0005681 functional_description:"Pre-mRNA-processing-splicing factor 8 (AHRD V1 ***- D0NFV2_PHYIN); contains Interpro domain(s) IPR012592 PROCN " | 3E-53 |
| 704 | Contig 704 | 495 | 1 | GH204119 | ********* |  |
| 705 | Contig 705 | 572 | 1 | GH204128 | Solyc01g105410.2.1 genomic_reference:SL2.40ch01 gene_region:85348461-85349698 transcript_region:SL2.40ch01:85348461..85349698+ functional_description:"Os06g0220000 protein (Fragment) (AHRD V1 ***- Q0DDJ2_ORYSJ); contains Interpro domain(s) IPR006766 Phosphate-induced protein 1 conserved region " | 1E-110 |
| 706 | Contig 706 | 506 | 1 | GH204960 | Solyc11g069410.1.1 evidence_code:10F1H0E1IEG genomic_reference:SL2.40ch11 gene_region:51081439-51084930 transcript_region:SL2.40ch11:51081439..51084930+ functional_description:"Zinc finger C3HC4 type family protein expressed (AHRD V1 **-- Q7XDA5_ORYSJ)" | 2E-17 |
| 707 | Contig 707 | 134 | 1 | GH204122 | ********* |  |
| 708 | Contig 708 | 370 | 1 | GH204990 | Solyc04g007790.2.1 genomic_reference:SL2.40ch04 gene_region:1474832-1476779 transcript_region:SL2.40ch04:1474832..1476779+ go_terms:GO:0009607 functional_description:"Major latex-like protein (AHRD V1 **-- B5THI3_PANGI); contains Interpro domain(s) IPR000916 Bet v I allergen " | 3E-25 |
| 709 | Contig 709 | 409 | 1 | GH204994 | Solyc03g121330.2.1 genomic_reference:SL2.40ch03 gene_region:63514413-63516687 transcript_region:SL2.40ch03:63514413..63516687+ go_terms:GO:0005515 functional_description:"60S ribosomal protein L28 (AHRD V1 ***- B3TLS3_ELAGV); contains Interpro domain(s) IPR002672 Ribosomal protein L28e " | 6E-61 |
| 710 | Contig 710 | 194 | 1 | GH204121 | Solyc06g009290.2.1 genomic_reference:SL2.40ch06 gene_region:3249411-3257702 transcript_region:SL2.40ch06:3249411..3257702- go_terms:GO:0010329,GO:0008559 functional_description:"Lipid A export ATP-binding/permease protein msbA (AHRD V1 *--- MSBA_SYNAS); contains Interpro domain(s) IPR003439 ABC transporter-like " | 8E-13 |
| 711 | Contig 711 | 311 | 1 | GH204977 | Solyc03g083530.2.1 genomic_reference:SL2.40ch03 gene_region:47018628-47020981 transcript_region:SL2.40ch03:47018628..47020981+ go_terms:GO:0003735,GO:0070181 functional_description:"40S ribosomal protein S13 (AHRD V1 ***- D7LRU2_ARALY); contains Interpro domain(s) IPR000589 Ribosomal protein S15 " | 5E-46 |
| 712 | Contig 712 | 192 | 1 | GH204962 | ********* |  |
| 713 | Contig 713 | 378 | 1 | GH204967 | Solyc03g115630.2.1 genomic_reference:SL2.40ch03 gene_region:59330578-59335551 transcript_region:SL2.40ch03:59330578..59335551+ go_terms:GO:0005515,GO:0016301 functional_description:"Carbamoyl-phosphate synthase small chain (AHRD V1 ***- B6TJA4_MAIZE); contains Interpro domain(s) IPR006274 Carbamoyl phosphate synthase, small subunit " | 6E-67 |
| 714 | Contig 714 | 594 | 1 | GH204968 | Solyc03g034190.2.1 genomic_reference:SL2.40ch03 gene_region:10158743-10161172 transcript_region:SL2.40ch03:10158743..10161172+ go_terms:GO:0009536 functional_description:"Ribosomal protein PSRP-3/Ycf65 (AHRD V1 *-*- B5W261_SPIMA); contains Interpro domain(s) IPR006924 Ribosomal protein, PSRP-3/Ycf65 " | 2E-58 |
| 715 | Contig 715 | 692 | 1 | GH204115 | Solyc10g005110.2.1 genomic_reference:SL2.40ch10 gene_region:96242-103626 transcript_region:SL2.40ch10:96242..103626- go_terms:GO:0042803 functional_description:"Coproporphyrinogen III oxidase aerobic (AHRD V1 ***- A9DTF4_9FLAO); contains Interpro domain(s) IPR001260 Coproporphyrinogen III oxidase " | 7E-14 |
| 716 | Contig 716 | 614 | 1 | GH204114 | Solyc07g041750.2.1 genomic_reference:SL2.40ch07 gene_region:51404606-51417208 transcript_region:SL2.40ch07:51404606..51417208- functional_description:"Os03g0169000 protein (Fragment) (AHRD V1 ***- Q0DUS7_ORYSJ); contains Interpro domain(s) IPR004348 Protein of unknown function DUF246, plant " | 1E-119 |
| 717 | Contig 717 | 185 | 1 | GH204104 | Solyc11g005630.1.1 evidence_code:10F0H1E1IEG genomic_reference:SL2.40ch11 gene_region:483096-485429 transcript_region:SL2.40ch11:483096..485429+ go_terms:GO:0005529,GO:0006468 functional_description:"Receptor-like protein kinase (AHRD V1 ***- Q39202_ARATH); contains Interpro domain(s) IPR002290 Serine/threonine protein kinase " | 2E-27 |
| 718 | Contig 718 | 699 | 1 | GH204973 | Solyc03g116600.2.1 genomic_reference:SL2.40ch03 gene_region:59993979-59998304 transcript_region:SL2.40ch03:59993979..59998304- go_terms:GO:0006810 functional_description:"Zeta2-COP (AHRD V1 **-- A8JHN4_CHLRE); contains Interpro domain(s) IPR011012 Longin-like " | 1E-55 |
| 719 | Contig 719 | 605 | 1 | GH204127 | Solyc04g009440.2.1 genomic_reference:SL2.40ch04 gene_region:2856469-2858639 transcript_region:SL2.40ch04:2856469..2858639+ go_terms:GO:0003700,GO:0016563 functional_description:"NAC domain protein (AHRD V1 ***- Q6RH27_SOLLC); contains Interpro domain(s) IPR003441 No apical meristem (NAM) protein " | 7E-86 |
| 720 | Contig 720 | 663 | 1 | GH204103 | Solyc03g080160.2.1 genomic_reference:SL2.40ch03 gene_region:45598367-45600275 transcript_region:SL2.40ch03:45598367..45600275- go_terms:GO:0051082 functional_description:"Nascent polypeptide-associated complex alpha subunit-like protein (AHRD V1 ***- B6SU89_MAIZE); contains Interpro domain(s) IPR016641 Nascent polypeptide-associated complex, alpha subunit " | 2E-52 |
| 721 | Contig 721 | 270 | 1 | GH204107 | Solyc10g044470.1.1 evidence_code:10F0H1E1IEG genomic_reference:SL2.40ch10 gene_region:21959526-21966277 transcript_region:SL2.40ch10:21959526..21966277+ go_terms:GO:0005247 functional_description:"Voltage-gated chloride channel (AHRD V1 **** Q96325_ARATH); contains Interpro domain(s) IPR002251 Chloride channel plant CLC " | 1E-32 |
| 722 | Contig 722 | 722 | 1 | GH204094 | Solyc10g084400.1.1 evidence_code:10F1H1E1IEG genomic_reference:SL2.40ch10 gene_region:63281886-63284225 transcript_region:SL2.40ch10:63281886..63284225- functional_description:"Glutathione S-transferase (AHRD V1 ***- Q76KW1_PEA); contains Interpro domain(s) IPR017933 Glutathione S-transferase/chloride channel, C-terminal " | 3E-94 |
| 723 | Contig 723 | 543 | 1 | GH204985 | Solyc03g031940.2.1 genomic_reference:SL2.40ch03 gene_region:8685384-8688719 transcript_region:SL2.40ch03:8685384..8688719- go_terms:GO:0018858,GO:0015645 functional_description:"Acyl-CoA synthetase/AMP-acid ligase II (AHRD V1 **** D0C359_9GAMM); contains Interpro domain(s) IPR000873 AMP-dependent synthetase and ligase " | 1E-104 |
| 724 | Contig 724 | 338 | 1 | GH204130 | Solyc06g065970.1.1 evidence_code:10F1H1E1IEG genomic_reference:SL2.40ch06 gene_region:37743284-37743664 transcript_region:SL2.40ch06:37743284..37743664+ go_terms:GO:0008289 functional_description:"Cortical cell-delineating protein (AHRD V1 **-- B6UGA2_MAIZE); contains Interpro domain(s) IPR013770 Plant lipid transfer protein and hydrophobic protein, helical " | 4E-11 |
| 725 | Contig 725 | 235 | 1 | GH204987 | Solyc03g065340.2.1 genomic_reference:SL2.40ch03 gene_region:38799475-38808273 transcript_region:SL2.40ch03:38799475..38808273+ go_terms:GO:0008184 functional_description:"Phosphorylase (AHRD V1 **** B9SJB6_RICCO); contains Interpro domain(s) IPR011833 Glycogen/starch/alpha-glucan phosphorylase " | 2E-31 |
| 726 | Contig 726 | 675 | 1 | GH204097 | Solyc03g082590.2.1 genomic_reference:SL2.40ch03 gene_region:46063003-46070268 transcript_region:SL2.40ch03:46063003..46070268+ go_terms:GO:0005622 functional_description:"TBC1 domain family member 15 (AHRD V1 *--- Q6P4X9_XENTR); contains Interpro domain(s) IPR000195 RabGAP/TBC " | 1E-113 |
| 727 | Contig 727 | 120 | 1 | GH204921 | Solyc03g117630.1.1 evidence_code:10F0H1E1IEG genomic_reference:SL2.40ch03 gene_region:60777610-60779574 transcript_region:SL2.40ch03:60777610..60779574- go_terms:GO:0051082,GO:0005524 functional_description:"heat shock protein (AHRD V1 ***- B2D2G5_CAPSN); contains Interpro domain(s) IPR013126 Heat shock protein 70 " | 9E-17 |
| 728 | Contig 728 | 301 | 1 | GH204920 | ********* |  |
| 729 | Contig 729 | 585 | 1 | GH204188 | Solyc02g070480.2.1 genomic_reference:SL2.40ch02 gene_region:34799332-34800890 transcript_region:SL2.40ch02:34799332..34800890+ functional_description:"F-box family protein (AHRD V1 ***- B9H2A8_POPTR); contains Interpro domain(s) IPR001810 Cyclin-like F-box " | 5E-28 |
| 730 | Contig 730 | 291 | 1 | GH204526 | ********* |  |
| 731 | Contig 731 | 643 | 1 | GH204709 | Solyc08g083330.2.1 genomic_reference:SL2.40ch08 gene_region:62986084-62991290 transcript_region:SL2.40ch08:62986084..62991290- functional_description:"Membrane related protein (AHRD V1 **-- Q6ZXK5_ORYSJ); contains Interpro domain(s) IPR002913 Lipid-binding START " | 7E-60 |
| 732 | Contig 732 | 426 | 1 | GH204986 | Solyc03g094010.2.1 genomic_reference:SL2.40ch03 gene_region:49163343-49166873 transcript_region:SL2.40ch03:49163343..49166873- go_terms:GO:0004353 functional_description:"Glutamate dehydrogenase (AHRD V1 **** Q94IA5_BRANA); contains Interpro domain(s) IPR014362 Glutamate dehydrogenase " | 1E-26 |
| 733 | Contig 733 | 473 | 1 | GH205000 | Solyc12g094620.1.1 evidence_code:10F0H1E1IEG genomic_reference:SL2.40ch12 gene_region:63142846-63144894 transcript_region:SL2.40ch12:63142846..63144894+ go_terms:GO:0055114 functional_description:"Catalase (AHRD V1 ***- Q2PYW5_SOLTU); contains Interpro domain(s) IPR018028 Catalase related subgroup " | 2E-90 |
| 734 | Contig 734 | 485 | 1 | GH204651 | Solyc06g065560.1.1 evidence_code:10F1H1E1IEG genomic_reference:SL2.40ch06 gene_region:37340923-37342464 transcript_region:SL2.40ch06:37340923..37342464- go_terms:GO:0009922 functional_description:"Fatty acid elongase 3-ketoacyl-CoA synthase (AHRD V1 **** Q6DUV6_BRANA); contains Interpro domain(s) IPR012392 Very-long-chain 3-ketoacyl-CoA synthase " | 3E-31 |
| 735 | Contig 735 | 460 | 1 | GH204989 | ********* |  |
| 736 | Contig 736 | 121 | 1 | GH204530 | ********* |  |
| 737 | Contig 737 | 325 | 1 | GH204085 | Solyc01g096360.2.1 genomic_reference:SL2.40ch01 gene_region:79201448-79205523 transcript_region:SL2.40ch01:79201448..79205523+ go_terms:GO:0005622 functional_description:"Rho GTPase activating protein 2 (AHRD V1 ***- Q6UQ72_ORYSJ); contains Interpro domain(s) IPR000198 RhoGAP " | 1E-43 |
| 738 | Contig 738 | 191 | 1 | GH204102 | Solyc08g061850.2.1 genomic_reference:SL2.40ch08 gene_region:46828543-46831672 transcript_region:SL2.40ch08:46828543..46831672- go_terms:GO:0003735 functional_description:"Ribosomal protein (AHRD V1 ***- B3TLR8_ELAGV); contains Interpro domain(s) IPR005703 Ribosomal protein S3, eukaryotic/archaeal " | 9E-25 |
| 739 | Contig 739 | 233 | 1 | GH204980 | Solyc01g103540.2.1 genomic_reference:SL2.40ch01 gene_region:83887917-83893923 transcript_region:SL2.40ch01:83887917..83893923+ functional_description:"YTH domain family 2 (Predicted) (AHRD V1 *-*- B2GUU1_RAT); contains Interpro domain(s) IPR007275 YT521-B-like protein " | 4E-42 |
| 740 | Contig 740 | 377 | 1 | GH204702 | Solyc01g081510.2.1 genomic_reference:SL2.40ch01 gene_region:73219130-73220362 transcript_region:SL2.40ch01:73219130..73220362+ go_terms:GO:0050662,GO:0008152,GO:0016491,GO:0055114 functional_description:"3-hydroxyisobutyrate dehydrogenase (AHRD V1 ***- D8MAS0_BLAHO); contains Interpro domain(s) IPR015815 3-hydroxyacid dehydrogenase/reductase " | 1E-16 |
| 741 | Contig 741 | 568 | 1 | GH204098 | Solyc12g095760.1.1 evidence_code:10F1H1E1IEG genomic_reference:SL2.40ch12 gene_region:63231944-63238575 transcript_region:SL2.40ch12:63231944..63238575- go_terms:GO:0005576,GO:0005945 functional_description:"Diphosphate--fructose-6- phosphate 1-phosphotransferase (AHRD V1 ***- C8PT93_9SPIO); contains Interpro domain(s) IPR011183 Pyrophosphate-dependent phosphofructokinase PfpB " | 1E-105 |
| 742 | Contig 742 | 364 | 1 | GH204011 | Solyc01g060470.2.1 genomic_reference:SL2.40ch01 gene_region:63867613-63872933 transcript_region:SL2.40ch01:63867613..63872933+ go_terms:GO:0008320 functional_description:"Importin alpha-1b subunit (AHRD V1 ***- B6T451_MAIZE); contains Interpro domain(s) IPR011989 Armadillo-like helical " | 3E-46 |
| 743 | Contig 743 | 397 | 1 | GH204039 | Solyc02g082570.1.1 evidence_code:10F0H0E1IEG genomic_reference:SL2.40ch02 gene_region:40814597-40815712 transcript_region:SL2.40ch02:40814597..40815712+ functional_description:"Unknown Protein (AHRD V1)" | 1E-62 |
| 744 | Contig 744 | 535 | 1 | GH205028 | Solyc10g005110.2.1 genomic_reference:SL2.40ch10 gene_region:96242-103626 transcript_region:SL2.40ch10:96242..103626- go_terms:GO:0042803 functional_description:"Coproporphyrinogen III oxidase aerobic (AHRD V1 ***- A9DTF4_9FLAO); contains Interpro domain(s) IPR001260 Coproporphyrinogen III oxidase " | 1E-101 |
| 745 | Contig 745 | 477 | 1 | GH204048 | Solyc01g111780.2.1 genomic_reference:SL2.40ch01 gene_region:89713800-89731648 transcript_region:SL2.40ch01:89713800..89731648+ go_terms:GO:0008320 functional_description:"Importin beta-2 subunit (AHRD V1 **-- C5JY88_AJEDS); contains Interpro domain(s) IPR011989 Armadillo-like helical " | 2E-65 |
| 746 | Contig 746 | 246 | 1 | GH204046 | ********* |  |
| 747 | Contig 747 | 353 | 1 | GH204536 | ********* |  |
| 748 | Contig 748 | 611 | 1 | GH204391 | Solyc01g095050.2.1 genomic_reference:SL2.40ch01 gene_region:78179288-78180925 transcript_region:SL2.40ch01:78179288..78180925- functional_description:"Negatively light-regulated protein (AHRD V1 ***- B4FAY8_MAIZE); contains Interpro domain(s) IPR012482 Lg106-like " | 5E-36 |
| 749 | Contig 749 | 681 | 1 | GH204589 | Solyc02g087650.2.1 genomic_reference:SL2.40ch02 gene_region:44594705-44595462 transcript_region:SL2.40ch02:44594705..44595462+ functional_description:"Unknown Protein (AHRD V1)" | 2E-59 |
| 750 | Contig 750 | 658 | 1 | GH204040 | Solyc06g053310.2.1 genomic_reference:SL2.40ch06 gene_region:32541547-32550340 transcript_region:SL2.40ch06:32541547..32550340+ go_terms:GO:0004416 functional_description:"Hydroxyacylglutathione hydrolase 2 (AHRD V1 **** B6T9Z9_MAIZE); contains Interpro domain(s) IPR017782 Hydroxyacylglutathione hydrolase " | 1E-113 |
| 751 | Contig 751 | 556 | 1 | GH204668 | Solyc10g081370.1.1 evidence_code:10F1H0E1IEG genomic_reference:SL2.40ch10 gene_region:61786900-61789542 transcript_region:SL2.40ch10:61786900..61789542+ go_terms:GO:0005634 functional_description:"Small nuclear ribonucleoprotein F (AHRD V1 **-- B6TPP2_MAIZE); contains Interpro domain(s) IPR016487 Small nuclear ribonucleoprotein SmF " | 9E-35 |
| 752 | Contig 752 | 746 | 1 | GH204043 | Solyc02g030170.2.1 genomic_reference:SL2.40ch02 gene_region:15953224-15955598 transcript_region:SL2.40ch02:15953224..15955598- go_terms:GO:0042605,GO:0019899 functional_description:"FAD linked oxidase domain protein (AHRD V1 **-- D1VPW7_9ACTO); contains Interpro domain(s) IPR006094 FAD linked oxidase, N-terminal " | 1E-127 |
| 753 | Contig 753 | 656 | 1 | GH204037 | Solyc01g110120.2.1 genomic_reference:SL2.40ch01 gene_region:88653085-88663519 transcript_region:SL2.40ch01:88653085..88663519- go_terms:GO:0046961,GO:0009678 functional_description:"V-type proton ATPase subunit a (AHRD V1 **** VPH1_NEUCR); contains Interpro domain(s) IPR002490 ATPase, V0/A0 complex, 116-kDa subunit " | 1E-127 |
| 754 | Contig 754 | 241 | 1 | GH204031 | Solyc02g070340.2.1 genomic_reference:SL2.40ch02 gene_region:34684400-34688917 transcript_region:SL2.40ch02:34684400..34688917+ go_terms:GO:0032183,GO:0003735 functional_description:"40S ribosomal protein S4-like protein (AHRD V1 ***- Q2XPX5_SOLTU); contains Interpro domain(s) IPR000876 Ribosomal protein S4e " | 1E-28 |
| 755 | Contig 755 | 597 | 1 | GH204026 | Solyc01g108150.2.1 genomic_reference:SL2.40ch01 gene_region:87266478-87282799 transcript_region:SL2.40ch01:87266478..87282799- go_terms:GO:0008152,GO:0016491,GO:0055114 functional_description:"Oxidoreductase zinc-binding dehydrogenase family protein (AHRD V1 *--- Q22RF9_TETTH); contains Interpro domain(s) IPR002085 Alcohol dehydrogenase superfamily, zinc-containing " | 1E-103 |
| 756 | Contig 756 | 733 | 1 | GH205034 | Solyc08g062420.2.1 genomic_reference:SL2.40ch08 gene_region:48280886-48282192 transcript_region:SL2.40ch08:48280886..48282192- functional_description:"Genomic DNA chromosome 5 P1 clone MJJ3 (AHRD V1 ***- Q9FFJ8_ARATH)" | 1E-100 |
| 757 | Contig 757 | 119 | 1 | GH204022 | Solyc11g069180.1.1 evidence_code:10F0H1E1IEG genomic_reference:SL2.40ch11 gene_region:50892679-50899807 transcript_region:SL2.40ch11:50892679..50899807+ go_terms:GO:0055114 functional_description:"Isovaleryl-CoA dehydrogenase (AHRD V1 ***- Q0MX57_BETVU); contains Interpro domain(s) IPR009100 Acyl-CoA dehydrogenase/oxidase, middle and N-terminal " | 2E-13 |
| 758 | Contig 758 | 804 | 1 | GH204024 | Solyc02g086710.2.1 genomic_reference:SL2.40ch02 gene_region:43930486-43935980 transcript_region:SL2.40ch02:43930486..43935980+ go_terms:GO:0005524,GO:0016656 functional_description:"Monodehydroascorbate reductase (NADH)-like protein (AHRD V1 **** Q0WUJ1_ARATH); contains Interpro domain(s) IPR013027 FAD-dependent pyridine nucleotide-disulphide oxidoreductase " | 1E-125 |
| 759 | Contig 759 | 620 | 1 | GH204045 | Solyc12g013890.1.1 evidence_code:10F0H1E1IEG genomic_reference:SL2.40ch12 gene_region:4682111-4693552 transcript_region:SL2.40ch12:4682111..4693552+ functional_description:"Genomic DNA chromosome 5 TAC clone K19P17 (AHRD V1 ***- Q9FN40_ARATH)" | 6E-94 |
| 760 | Contig 760 | 150 | 1 | GH204017 | ********* |  |
| 761 | Contig 761 | 649 | 1 | GH204025 | Solyc11g069790.1.1 evidence_code:10F1H1E1IEG genomic_reference:SL2.40ch11 gene_region:51502451-51507359 transcript_region:SL2.40ch11:51502451..51507359- go_terms:GO:0044267,GO:0005737 functional_description:"chaperonin (AHRD V1 ***- B2IXD2_NOSP7); contains Interpro domain(s) IPR001844 Chaperonin Cpn60 " | 6E-47 |
| 762 | Contig 762 | 108 | 1 | GH204790 | Solyc05g046390.2.1 genomic_reference:SL2.40ch05 gene_region:57965282-57966565 transcript_region:SL2.40ch05:57965282..57966565- functional_description:"Unknown Protein (AHRD V1)" | 1E-14 |
| 763 | Contig 763 | 359 | 1 | GH204396 | ********* |  |
| 764 | Contig 764 | 103 | 1 | GH204801 | ********* |  |
| 765 | Contig 765 | 430 | 1 | GH204393 | ********* |  |
| 766 | Contig 766 | 716 | 1 | GH204398 | Solyc05g056090.2.1 genomic_reference:SL2.40ch05 gene_region:64613030-64615907 transcript_region:SL2.40ch05:64613030..64615907+ functional_description:"Unknown Protein (AHRD V1); contains Interpro domain(s) IPR009724 Protein of unknown function DUF1301 " | 2E-47 |
| 767 | Contig 767 | 833 | 1 | GH204354 | Solyc08g075860.2.1 genomic_reference:SL2.40ch08 gene_region:57100097-57105604 transcript_region:SL2.40ch08:57100097..57105604- functional_description:"Os06g0115800 protein (Fragment) (AHRD V1 *-*- Q0DF43_ORYSJ)" | 1E-124 |
| 768 | Contig 768 | 673 | 1 | GH204599 | Solyc05g015980.2.1 genomic_reference:SL2.40ch05 gene_region:13141497-13144413 transcript_region:SL2.40ch05:13141497..13144413- functional_description:"Unknown Protein (AHRD V1)" | 5E-71 |
| 769 | Contig 769 | 577 | 1 | GH204343 | ********* |  |
| 770 | Contig 770 | 524 | 1 | GH204351 | Solyc06g069520.2.1 genomic_reference:SL2.40ch06 gene_region:39682005-39684690 transcript_region:SL2.40ch06:39682005..39684690+ go_terms:GO:0005515 functional_description:"UPF0139 membrane protein At5g07960 (AHRD V1 ***- U139_ARATH); contains Interpro domain(s) IPR005351 Uncharacterised protein family UPF0139 " | 2E-36 |
| 771 | Contig 771 | 620 | 1 | GH204358 | ********* |  |
| 772 | Contig 772 | 343 | 1 | GH204394 | Solyc07g044840.2.1 genomic_reference:SL2.40ch07 gene_region:55217173-55222925 transcript_region:SL2.40ch07:55217173..55222925+ go_terms:GO:0008830,GO:0004619 functional_description:"2 3-bisphosphoglycerate-independent phosphoglycerate mutase (AHRD V1 **** B9S1V6_RICCO); contains Interpro domain(s) IPR005995 Phosphoglycerate mutase, 2,3-bisphosphoglycerate-independent " | 4E-62 |
| 773 | Contig 773 | 478 | 1 | GH204799 | Solyc01g103430.2.1 genomic_reference:SL2.40ch01 gene_region:83804323-83813569 transcript_region:SL2.40ch01:83804323..83813569+ go_terms:GO:0004696 functional_description:"Glycogen synthase kinase (AHRD V1 **** C7AE95_SOYBN); contains Interpro domain(s) IPR002290 Serine/threonine protein kinase " | 3E-86 |
| 774 | Contig 774 | 190 | 1 | GH204390 | ********* |  |
| 775 | Contig 775 | 189 | 1 | GH204791 | Solyc11g068400.1.1 evidence_code:10F1H1E1IEG genomic_reference:SL2.40ch11 gene_region:50223989-50227276 transcript_region:SL2.40ch11:50223989..50227276- go_terms:GO:0005740 functional_description:"Ubiquinol-cytochrome C reductase (AHRD V1 ***- D6BQN0_9ROSI); contains Interpro domain(s) IPR008027 Ubiquinol-cytochrome C reductase, UQCRX/QCR9-like " | 1E-22 |
| 776 | Contig 776 | 406 | 1 | GH204481 | Solyc02g078870.1.1 evidence_code:10F1H0E1IEG genomic_reference:SL2.40ch02 gene_region:38092358-38092510 transcript_region:SL2.40ch02:38092358..38092510+ functional_description:"Unknown Protein (AHRD V1)" | 2E-22 |
| 777 | Contig 777 | 227 | 1 | GH204480 | ********* |  |
| 778 | Contig 778 | 228 | 1 | GH204728 | Solyc07g063960.2.1 genomic_reference:SL2.40ch07 gene_region:63515688-63518536 transcript_region:SL2.40ch07:63515688..63518536+ go_terms:GO:0005840 functional_description:"50S ribosomal protein L24 (AHRD V1 ***- B4FTJ3_MAIZE); contains Interpro domain(s) IPR003256 Ribosomal protein L24 " | 2E-12 |
| 779 | Contig 779 | 254 | 1 | GH204402 | Solyc08g048550.2.1 genomic_reference:SL2.40ch08 gene_region:40724238-40730507 transcript_region:SL2.40ch08:40724238..40730507- go_terms:GO:0004252 functional_description:"Protease Do-like (S2 serine-type protease) (AHRD V1 **** A6CGY1_9PLAN); contains Interpro domain(s) IPR001254 Peptidase S1 and S6, chymotrypsin/Hap " | 2E-43 |
| 780 | Contig 780 | 351 | 1 | GH204666 | ********* |  |
| 781 | Contig 781 | 450 | 1 | GH204399 | Solyc05g013910.2.1 genomic_reference:SL2.40ch05 gene_region:7349028-7353433 transcript_region:SL2.40ch05:7349028..7353433+ functional_description:"Unknown Protein (AHRD V1)" | 2E-19 |
| 782 | Contig 782 | 755 | 1 | GH205081 | Solyc06g073320.2.1 genomic_reference:SL2.40ch06 gene_region:41566941-41570177 transcript_region:SL2.40ch06:41566941..41570177+ go_terms:GO:0080046,GO:0010475,GO:0080048 functional_description:"GDP-L-galactose phosphorylase 1" | 1E-115 |
| 783 | Contig 783 | 401 | 1 | GH203951 | Solyc05g013380.2.1 genomic_reference:SL2.40ch05 gene_region:6451782-6457884 transcript_region:SL2.40ch05:6451782..6457884+ go_terms:GO:0004021 functional_description:"Alanine aminotransferase 2 (AHRD V1 **** A8IKE5_SOYBN); contains Interpro domain(s) IPR004839 Aminotransferase, class I and II " | 2E-71 |
| 784 | Contig 784 | 587 | 1 | GH203928 | Solyc08g068600.2.1 genomic_reference:SL2.40ch08 gene_region:54940071-54942182 transcript_region:SL2.40ch08:54940071..54942182- go_terms:GO:0030170,GO:0019752 functional_description:"Decarboxylase family protein (AHRD V1 ***- B1ILJ6_CLOBK); contains Interpro domain(s) IPR002129 Pyridoxal phosphate-dependent decarboxylase " | 1E-109 |
| 785 | Contig 785 | 481 | 1 | GH205080 | ********* |  |
| 786 | Contig 786 | 395 | 1 | GH203948 | Solyc04g007550.2.1 genomic_reference:SL2.40ch04 gene_region:1237504-1241655 transcript_region:SL2.40ch04:1237504..1241655+ go_terms:GO:0005507 functional_description:"ATP synthase subunit beta (AHRD V1 ***- O82722_NICSY); contains Interpro domain(s) IPR005722 ATPase, F1 complex, beta subunit " | 2E-70 |
| 787 | Contig 787 | 219 | 1 | GH203934 | ********* |  |
| 788 | Contig 788 | 419 | 1 | GH205074 | Solyc11g005170.1.1 evidence_code:10F0H1E1IEG genomic_reference:SL2.40ch11 gene_region:147568-156637 transcript_region:SL2.40ch11:147568..156637+ go_terms:GO:0003676,GO:0005634,GO:0000166 functional_description:"RNA splicing factor (AHRD V1 **-- C5JY77_AJEDS); contains Interpro domain(s) IPR006509 Splicing factor, CC1-like " | 9E-63 |
| 789 | Contig 789 | 682 | 1 | GH203969 | Solyc09g090140.2.1 genomic_reference:SL2.40ch09 gene_region:65031288-65034684 transcript_region:SL2.40ch09:65031288..65034684- go_terms:GO:0005515,GO:0030060 functional_description:"Malate dehydrogenase (AHRD V1 **** Q2PYY8_SOLTU); contains Interpro domain(s) IPR011274 Malate dehydrogenase, NAD-dependent, cytosolic " | 1E-125 |
| 790 | Contig 790 | 416 | 1 | GH204542 | Solyc06g007670.2.1 genomic_reference:SL2.40ch06 gene_region:1672485-1676176 transcript_region:SL2.40ch06:1672485..1676176- go_terms:GO:0003735,GO:0008097 functional_description:"60S ribosomal protein L5-1 (AHRD V1 ***- B6THG9_MAIZE); contains Interpro domain(s) IPR005485 Ribosomal protein L5, eukaryotic " | 4E-76 |
| 791 | Contig 791 | 391 | 1 | GH205082 | Solyc02g079770.2.1 genomic_reference:SL2.40ch02 gene_region:38805497-38807567 transcript_region:SL2.40ch02:38805497..38807567+ functional_description:"DAG protein (AHRD V1 ***- B6TYI4_MAIZE)" | 5E-62 |
| 792 | Contig 792 | 247 | 1 | GH204001 | Solyc06g005560.2.1 genomic_reference:SL2.40ch06 gene_region:592513-595666 transcript_region:SL2.40ch06:592513..595666- go_terms:GO:0005576,GO:0009664 functional_description:"Expansin-1 (AHRD V1 ***- Q6RX69_PETHY); contains Interpro domain(s) IPR007112 Expansin 45, endoglucanase-like IPR007117 Pollen allergen/expansin, C-terminal " | 4E-49 |
| 793 | Contig 793 | 143 | 1 | GH205048 | ********* |  |
| 794 | Contig 794 | 263 | 1 | GH203998 | Solyc07g041970.2.1 genomic_reference:SL2.40ch07 gene_region:52037727-52040636 transcript_region:SL2.40ch07:52037727..52040636+ go_terms:GO:0004252 functional_description:"Subtilisin-like protease (AHRD V1 **-- B6SZ82_MAIZE); contains Interpro domain(s) IPR015500 Peptidase S8, subtilisin-related " | 2E-38 |
| 795 | Contig 795 | 811 | 1 | GH203992 | Solyc01g079480.2.1 genomic_reference:SL2.40ch01 gene_region:71029166-71030328 transcript_region:SL2.40ch01:71029166..71030328- go_terms:GO:0043565,GO:0003700,GO:0046982 functional_description:"BZIP transcription factor (AHRD V1 **-* Q1HW69_CAPAN); contains Interpro domain(s) IPR011616 bZIP transcription factor, bZIP-1 " | 1E-78 |
| 796 | Contig 796 | 523 | 1 | GH205065 | Solyc04g082820.2.1 genomic_reference:SL2.40ch04 gene_region:63943159-63954735 transcript_region:SL2.40ch04:63943159..63954735- go_terms:GO:0005622 functional_description:"ARID/BRIGHT DNA-binding domain-containing protein (AHRD V1 *-*- D7KTU4_ARALY); contains Interpro domain(s) IPR001606 AT-rich interaction region " | 3E-70 |
| 797 | Contig 797 | 667 | 1 | GH203933 | Solyc07g016210.1.1 evidence_code:10F0H0E0IEG genomic_reference:SL2.40ch07 gene_region:6483974-6485166 transcript_region:SL2.40ch07:6483974..6485166+ functional_description:"Organ-specific protein S2 (AHRD V1 *-*- OSS2_PEA)" | 6E-74 |
| 798 | Contig 798 | 174 | 1 | GH203932 | ********* |  |
| 799 | Contig 799 | 640 | 1 | GH205077 | Solyc11g012900.1.1 evidence_code:10F1H1E1IEG genomic_reference:SL2.40ch11 gene_region:5682714-5696581 transcript_region:SL2.40ch11:5682714..5696581- go_terms:GO:0005488 functional_description:"Small glutamine-rich tetratricopeptide repeat-containing protein A (AHRD V1 *--- C1BM93_OSMMO); contains Interpro domain(s) IPR011990 Tetratricopeptide-like helical " | 3E-84 |
| 800 | Contig 800 | 242 | 1 | GH203930 | Solyc11g068830.1.1 evidence_code:10F0H1E1IEG genomic_reference:SL2.40ch11 gene_region:50519655-50524370 transcript_region:SL2.40ch11:50519655..50524370+ go_terms:GO:0004588,GO:0004590 functional_description:"Orotidine 5&apos-phosphate decarboxylase (Orotate phosphoribosyltransferase) (AHRD V1 **** C4QAV6_SCHMA); contains Interpro domain(s) IPR001754 Orotidine 5'-phosphate decarboxylase, core " | 2E-33 |
| 801 | Contig 801 | 258 | 1 | GH203941 | Solyc09g011070.1.1 evidence_code:10F0H1E1IEG genomic_reference:SL2.40ch09 gene_region:4400701-4402746 transcript_region:SL2.40ch09:4400701..4402746- go_terms:GO:0005515,GO:0016301 functional_description:"Receptor like protein kinase (AHRD V1 **** Q39139_ARATH); contains Interpro domain(s) IPR001220 Legume lectin, beta chain " | 1E-11 |
| 802 | Contig 802 | 495 | 1 | GH204621 | Solyc04g007200.2.1 genomic_reference:SL2.40ch04 gene_region:896645-898572 transcript_region:SL2.40ch04:896645..898572+ go_terms:GO:0016291 functional_description:"Tol-pal system-associated acyl-CoA thioesterase (AHRD V1 **** C6E6Z6_GEOSM); contains Interpro domain(s) IPR006683 Thioesterase superfamily " | 3E-83 |
| 803 | Contig 803 | 137 | 1 | GH204081 | ********* |  |
| 804 | Contig 804 | 756 | 1 | GH204535 | Solyc01g103220.2.1 genomic_reference:SL2.40ch01 gene_region:83625084-83629542 transcript_region:SL2.40ch01:83625084..83629542+ go_terms:GO:0020037 functional_description:"Cytochrome c (AHRD V1 ***- B6SKR4_MAIZE); contains Interpro domain(s) IPR002327 Cytochrome c, class IA/ IB " | 5E-57 |
| 805 | Contig 805 | 563 | 1 | GH205004 | Solyc01g095640.1.1 evidence_code:10F0H0E0IEG genomic_reference:SL2.40ch01 gene_region:78616587-78617431 transcript_region:SL2.40ch01:78616587..78617431- go_terms:GO:0005515,GO:0003700 functional_description:"Myb family transcription factor (AHRD V1 **** D7LC50_ARALY); contains Interpro domain(s) IPR015495 Myb transcription factor " | 4E-24 |
| 806 | Contig 806 | 169 | 1 | GH204649 | Solyc01g104590.2.1 genomic_reference:SL2.40ch01 gene_region:84802263-84805701 transcript_region:SL2.40ch01:84802263..84805701- go_terms:GO:0003735,GO:0005515 functional_description:"Ribosomal protein L3 (AHRD V1 ***- Q6SKP4_SOLLC); contains Interpro domain(s) IPR019926 Ribosomal protein L3, conserved site IPR000597 Ribosomal protein L3 " | 1E-28 |
| 807 | Contig 807 | 341 | 1 | GH204077 | Solyc07g049180.2.1 genomic_reference:SL2.40ch07 gene_region:56760988-56770880 transcript_region:SL2.40ch07:56760988..56770880- go_terms:GO:0019199,GO:0005057 functional_description:"Receptor-like protein kinase At5g59670 (AHRD V1 *-** RLK7_ARATH); contains Interpro domain(s) IPR002290 Serine/threonine protein kinase " | 8E-44 |
| 808 | Contig 808 | 401 | 1 | GH204049 | Solyc06g060790.1.1 evidence_code:10F1H1E1IEG genomic_reference:SL2.40ch06 gene_region:35203918-35204688 transcript_region:SL2.40ch06:35203918..35204688- go_terms:GO:0016836 functional_description:"3-isopropylmalate dehydratase small subunit (AHRD V1 ***- Q9ZW85_ARATH); contains Interpro domain(s) IPR011827 3-isopropylmalate dehydratase, small subunit, subgroup " | 6E-50 |
| 809 | Contig 809 | 421 | 1 | GH204074 | Solyc06g051210.2.1 genomic_reference:SL2.40ch06 gene_region:30886606-30892332 transcript_region:SL2.40ch06:30886606..30892332- functional_description:"Bromodomain-containing protein (AHRD V1 ***- B9R8P3_RICCO); contains Interpro domain(s) IPR001487 Bromodomain " | 4E-46 |
| 810 | Contig 810 | 360 | 1 | GH204080 | Solyc05g056000.2.1 genomic_reference:SL2.40ch05 gene_region:64542811-64544762 transcript_region:SL2.40ch05:64542811..64544762+ go_terms:GO:0004622 functional_description:"Lipase-like protein (AHRD V1 **-- Q9M3D1_ARATH)" | 2E-42 |
| 811 | Contig 811 | 262 | 1 | GH204062 | Solyc01g107820.2.1 genomic_reference:SL2.40ch01 gene_region:86993487-86995355 transcript_region:SL2.40ch01:86993487..86995355- go_terms:GO:0080044 functional_description:"UDP-glucosyltransferase family 1 protein (AHRD V1 **** C6KI43_CITSI); contains Interpro domain(s) IPR002213 UDP-glucuronosyl/UDP-glucosyltransferase " | 3E-46 |
| 812 | Contig 812 | 425 | 1 | GH204699 | Solyc03g113460.1.1 evidence_code:10F0H1E1IEG genomic_reference:SL2.40ch03 gene_region:57655307-57656107 transcript_region:SL2.40ch03:57655307..57656107+ go_terms:GO:0005507 functional_description:"Alcohol dehydrogenase (Fragment) (AHRD V1 ***- Q6R4Y6_ZEALU); contains Interpro domain(s) IPR002347 Glucose/ribitol dehydrogenase " | 2E-14 |
| 813 | Contig 813 | 300 | 1 | GH204068 | Solyc10g006490.2.1 genomic_reference:SL2.40ch10 gene_region:1075550-1079615 transcript_region:SL2.40ch10:1075550..1079615+ go_terms:GO:0042802 functional_description:"Trafficking protein particle complex subunit 3 (AHRD V1 ***- B6T6G9_MAIZE); contains Interpro domain(s) IPR016721 TRAPP I complex, Bet3 " | 5E-20 |
| 814 | Contig 814 | 576 | 1 | GH203940 | Solyc03g098110.2.1 genomic_reference:SL2.40ch03 gene_region:53890467-53900661 transcript_region:SL2.40ch03:53890467..53900661- functional_description:"Genome sequencing data contig C315 (AHRD V1 ***- A8YHW9_MICAE)" | 4E-49 |
| 815 | Contig 815 | 342 | 1 | GH204015 | Solyc03g080050.2.1 genomic_reference:SL2.40ch03 gene_region:45473113-45480181 transcript_region:SL2.40ch03:45473113..45480181- go_terms:GO:0005102 functional_description:"Band 7 stomatin family protein (AHRD V1 **-- C1MUP6_MICPS); contains Interpro domain(s) IPR001972 Stomatin " | 1E-31 |
| 816 | Contig 816 | 633 | 1 | GH204073 | Solyc04g008660.2.1 genomic_reference:SL2.40ch04 gene_region:2289220-2305938 transcript_region:SL2.40ch04:2289220..2305938+ go_terms:GO:0032312,GO:0005737 functional_description:"Arf-GAP with GTPase, ANK repeat and PH domain-containing protein 1 (AHRD V1 *--- AGAP1_XENLA); contains Interpro domain(s) IPR001164 Arf GTPase activating protein " | 1E-69 |
| 817 | Contig 817 | 639 | 1 | GH204050 | Solyc08g005610.2.1 genomic_reference:SL2.40ch08 gene_region:477231-479683 transcript_region:SL2.40ch08:477231..479683+ go_terms:GO:0019825,GO:0010295 functional_description:"Cytochrome P450" | 1E-119 |
| 818 | Contig 818 | 216 | 1 | GH204066 | Solyc03g093480.2.1 genomic_reference:SL2.40ch03 gene_region:48226050-48252855 transcript_region:SL2.40ch03:48226050..48252855- go_terms:GO:0005515 functional_description:"U3 small nucleolar RNA-associated protein 10 (AHRD V1 *--- C8VGG5_EMENI); contains Interpro domain(s) IPR012954 BP28, C-terminal " | 4E-33 |
| 819 | Contig 819 | 345 | 1 | GH203674 | Solyc01g111980.2.1 genomic_reference:SL2.40ch01 gene_region:89876788-89879583 transcript_region:SL2.40ch01:89876788..89879583- go_terms:GO:0015171 functional_description:"Lysine/histidine transporter (AHRD V1 **** B9HR02_POPTR); contains Interpro domain(s) IPR013057 Amino acid transporter, transmembrane " | 2E-58 |
| 820 | Contig 820 | 316 | 1 | GH203943 | Solyc03g119080.2.1 genomic_reference:SL2.40ch03 gene_region:61837300-61840225 transcript_region:SL2.40ch03:61837300..61840225- go_terms:GO:0080083 functional_description:"Beta-glucosidase (AHRD V1 **** D7L7Z3_ARALY); contains Interpro domain(s) IPR001360 Glycoside hydrolase, family 1 " | 4E-60 |
| 821 | Contig 821 | 749 | 1 | GH205053 | Solyc11g017250.1.1 evidence_code:10F0H1E1IEG genomic_reference:SL2.40ch11 gene_region:8083498-8089958 transcript_region:SL2.40ch11:8083498..8089958+ go_terms:GO:0008152,GO:0016021 functional_description:"Dihydrolipoyllysine-residue acetyltransferase component of pyruvate dehydrogenase complex (AHRD V1 ***- A5GUY8_SYNR3); contains Interpro domain(s) IPR001078 2-oxoacid dehydrogenase acyltransferase, catalytic domain " | 4E-78 |
| 822 | Contig 822 | 785 | 1 | GH203997 | Solyc06g073430.2.1 genomic_reference:SL2.40ch06 gene_region:41644320-41646138 transcript_region:SL2.40ch06:41644320..41646138+ go_terms:GO:0003735 functional_description:"40S ribosomal protein S29 (AHRD V1 ***- A2ZG71_ORYSI); contains Interpro domain(s) IPR001209 Ribosomal protein S14 " | 8E-31 |
| 823 | Contig 823 | 591 | 1 | GH204704 | ********* |  |
| 824 | Contig 824 | 689 | 1 | GH204466 | Solyc01g065580.2.1 genomic_reference:SL2.40ch01 gene_region:64325048-64330129 transcript_region:SL2.40ch01:64325048..64330129- go_terms:GO:0005488,GO:0003723 functional_description:"Pumilio domain-containing protein KIAA0020 (AHRD V1 ***- B5X3V8_SALSA); contains Interpro domain(s) IPR011989 Armadillo-like helical " | 1E-127 |
| 825 | Contig 825 | 480 | 1 | GH205214 | Solyc12g096590.1.1 evidence_code:10F1H1E1IEG genomic_reference:SL2.40ch12 gene_region:63783509-63787350 transcript_region:SL2.40ch12:63783509..63787350- go_terms:GO:0009349 functional_description:"6 7-dimethyl-8-ribityllumazine synthase (AHRD V1 ***- B2CM14_SOLCH); contains Interpro domain(s) IPR002180 6,7-dimethyl-8-ribityllumazine synthase " | 4E-88 |
| 826 | Contig 826 | 901 | 1 | GH204463 | Solyc09g090660.2.1 genomic_reference:SL2.40ch09 gene_region:65460983-65469080 transcript_region:SL2.40ch09:65460983..65469080+ go_terms:GO:0042802 functional_description:"Guanine nucleotide-binding protein subunit beta-like protein (AHRD V1 *--* GBLP_SCHPO); contains Interpro domain(s) IPR017986 WD40 repeat, region " | 1E-154 |
| 827 | Contig 827 | 701 | 1 | GH203920 | Solyc11g013440.1.1 evidence_code:10F1H1E1IEG genomic_reference:SL2.40ch11 gene_region:6453735-6462143 transcript_region:SL2.40ch11:6453735..6462143+ functional_description:"Eukaryotic translation initiation factor 3 subunit 5 (AHRD V1 ***- B4FRD9_MAIZE); contains Interpro domain(s) IPR000555 Mov34/MPN/PAD-1 " | 3E-71 |
| 828 | Contig 828 | 250 | 1 | GH203430 | Solyc01g104950.2.1 genomic_reference:SL2.40ch01 gene_region:85031292-85035396 transcript_region:SL2.40ch01:85031292..85035396- go_terms:GO:0009044,GO:0046556 functional_description:"Alpha-L- arabinofuranosidase/beta-D-xylosidase (AHRD V1 **** D9D7L0_MALDO); contains Interpro domain(s) IPR001764 Glycoside hydrolase, family 3, N-terminal " | 4E-45 |
| 829 | Contig 829 | 283 | 1 | GH204475 | Solyc10g044470.1.1 evidence_code:10F0H1E1IEG genomic_reference:SL2.40ch10 gene_region:21959526-21966277 transcript_region:SL2.40ch10:21959526..21966277+ go_terms:GO:0005247 functional_description:"Voltage-gated chloride channel (AHRD V1 **** Q96325_ARATH); contains Interpro domain(s) IPR002251 Chloride channel plant CLC " | 6E-49 |
| 830 | Contig 830 | 359 | 1 | GH204177 | Solyc08g007690.1.1 evidence_code:10F0H1E1IEG genomic_reference:SL2.40ch08 gene_region:2183538-2185754 transcript_region:SL2.40ch08:2183538..2185754+ go_terms:GO:0004252 functional_description:"Subtilisin-like protease (AHRD V1 **-- Q9LWA3_SOLLC); contains Interpro domain(s) IPR015500 Peptidase S8, subtilisin-related " | 6E-64 |
| 831 | Contig 831 | 304 | 1 | GH204180 | Solyc04g024500.2.1 genomic_reference:SL2.40ch04 gene_region:12989357-12998283 transcript_region:SL2.40ch04:12989357..12998283+ functional_description:"UPF0760 protein C2orf29 homolog (AHRD V1 ***- CB029_DANRE)" | 2E-57 |
| 832 | Contig 832 | 478 | 1 | GH204930 | Solyc02g070310.2.1 genomic_reference:SL2.40ch02 gene_region:34670896-34672676 transcript_region:SL2.40ch02:34670896..34672676- go_terms:GO:0003735,GO:0005515 functional_description:"Ribosomal protein L32 (AHRD V1 ***- Q45NI6_MEDSA); contains Interpro domain(s) IPR018263 Ribosomal protein L32e, conserved site IPR001515 Ribosomal protein L32e " | 2E-66 |
| 833 | Contig 833 | 574 | 1 | GH203986 | Solyc02g088100.2.1 genomic_reference:SL2.40ch02 gene_region:44867449-44868915 transcript_region:SL2.40ch02:44867449..44868915- go_terms:GO:0005199 functional_description:"Expansin (AHRD V1 ***- Q9ZP31_SOLLC); contains Interpro domain(s) IPR007112 Expansin 45, endoglucanase-like IPR007117 Pollen allergen/expansin, C-terminal " | 1E-36 |
| 834 | Contig 834 | 336 | 1 | GH203414 | ********* |  |
| 835 | Contig 835 | 906 | 1 | GH203887 | Solyc02g081160.2.1 genomic_reference:SL2.40ch02 gene_region:39802521-39809198 transcript_region:SL2.40ch02:39802521..39809198- go_terms:GO:0005945 functional_description:"Diphosphate-fructose-6-phosphate 1-phosphotransferase (AHRD V1 ***- D1JTA2_9BACE); contains Interpro domain(s) IPR011183 Pyrophosphate-dependent phosphofructokinase PfpB " | 1E-167 |
| 836 | Contig 836 | 643 | 1 | GH205208 | Solyc05g053820.2.1 genomic_reference:SL2.40ch05 gene_region:63001106-63003424 transcript_region:SL2.40ch05:63001106..63003424- go_terms:GO:0010294 functional_description:"UDP-glucosyltransferase family 1 protein (AHRD V1 **** C6KI44_CITSI); contains Interpro domain(s) IPR002213 UDP-glucuronosyl/UDP-glucosyltransferase " | 7E-64 |
| 837 | Contig 837 | 765 | 1 | GH204471 | Solyc11g069320.1.1 evidence_code:10F0H1E1IEG genomic_reference:SL2.40ch11 gene_region:51014957-51015613 transcript_region:SL2.40ch11:51014957..51015613+ functional_description:"Kelch repeat-containing F-box family protein (AHRD V1 ***- D7MTV4_ARALY); contains Interpro domain(s) IPR015915 Kelch-type beta propeller " | 3E-78 |
| 838 | Contig 838 | 651 | 1 | GH204936 | Solyc02g082200.2.1 genomic_reference:SL2.40ch02 gene_region:40474471-40478745 transcript_region:SL2.40ch02:40474471..40478745+ go_terms:GO:0045454 functional_description:"Glutaredoxin (AHRD V1 ***- A9LC71_PTEVI); contains Interpro domain(s) IPR004480 Glutaredoxin-related protein " | 2E-86 |
| 839 | Contig 839 | 436 | 1 | GH204162 | Solyc02g020980.2.1 genomic_reference:SL2.40ch02 gene_region:11526882-11568630 transcript_region:SL2.40ch02:11526882..11568630- go_terms:GO:0004134,GO:0010297 functional_description:"4-alpha-glucanotransferase (AHRD V1 **** Q6R608_SOLTU); contains Interpro domain(s) IPR003385 Glycoside hydrolase, family 77 " | 3E-83 |
| 840 | Contig 840 | 468 | 1 | GH203744 | Solyc11g068820.1.1 evidence_code:10F0H1E1IEG genomic_reference:SL2.40ch11 gene_region:50513890-50516507 transcript_region:SL2.40ch11:50513890..50516507- go_terms:GO:0005840 functional_description:"50S ribosomal protein L27 (AHRD V1 ***- Q8LEF5_ARATH); contains Interpro domain(s) IPR001684 Ribosomal protein L27 " | 1E-47 |
| 841 | Contig 841 | 772 | 1 | GH203520 | Solyc04g071140.2.1 genomic_reference:SL2.40ch04 gene_region:55663674-55668653 transcript_region:SL2.40ch04:55663674..55668653+ go_terms:GO:0006468,GO:0019752,GO:0030170 functional_description:"Decarboxylase family protein (AHRD V1 ***- B1ILJ6_CLOBK); contains Interpro domain(s) IPR002129 Pyridoxal phosphate-dependent decarboxylase " | 1E-152 |
| 842 | Contig 842 | 778 | 1 | GH204303 | Solyc06g073050.2.1 genomic_reference:SL2.40ch06 gene_region:41395982-41399877 transcript_region:SL2.40ch06:41395982..41399877+ go_terms:GO:0045449 functional_description:"NAC domain protein IPR003441 (AHRD V1 *-*- B9IDH0_POPTR); contains Interpro domain(s) IPR003441 No apical meristem (NAM) protein " | 1E-134 |
| 843 | Contig 843 | 386 | 1 | GH204316 | Solyc12g056560.1.1 evidence_code:10F0H0E0IEG genomic_reference:SL2.40ch12 gene_region:47864564-47865470 transcript_region:SL2.40ch12:47864564..47865470+ functional_description:"Unknown Protein (AHRD V1); contains Interpro domain(s) IPR008978 HSP20-like chaperone " | 4E-52 |
| 844 | Contig 844 | 461 | 1 | GH205303 | Solyc05g053140.2.1 genomic_reference:SL2.40ch05 gene_region:62417721-62424738 transcript_region:SL2.40ch05:62417721..62424738- go_terms:GO:0005515 functional_description:"26S proteasome non-ATPase regulatory subunit 13 (AHRD V1 ***- B6TA88_MAIZE); contains Interpro domain(s) IPR000717 Proteasome component region PCI " | 5E-72 |
| 845 | Contig 845 | 554 | 1 | GH203735 | Solyc07g066470.2.1 genomic_reference:SL2.40ch07 gene_region:65112777-65116118 transcript_region:SL2.40ch07:65112777..65116118- go_terms:GO:0004418 functional_description:"Porphobilinogen deaminase (AHRD V1 ***- B6TVP0_MAIZE); contains Interpro domain(s) IPR000860 Tetrapyrrole biosynthesis, hydroxymethylbilane synthase " | 5E-88 |
| 846 | Contig 846 | 391 | 1 | GH204575 | ********* |  |
| 847 | Contig 847 | 794 | 1 | GH203587 | Solyc06g067910.2.1 genomic_reference:SL2.40ch06 gene_region:38479523-38482938 transcript_region:SL2.40ch06:38479523..38482938- functional_description:"Os01g0611000 protein (Fragment) (AHRD V1 ***- Q0JLB5_ORYSJ); contains Interpro domain(s) IPR006946 Protein of unknown function DUF642 " | 1E-125 |
| 848 | Contig 848 | 626 | 1 | GH205010 | Solyc12g010230.1.1 evidence_code:10F0H0E1IEG genomic_reference:SL2.40ch12 gene_region:3342181-3346441 transcript_region:SL2.40ch12:3342181..3346441+ functional_description:"Os06g0183700 protein (Fragment) (AHRD V1 *-*- Q0DE22_ORYSJ)" | 2E-43 |
| 849 | Contig 849 | 313 | 1 | GH205011 | Solyc03g114790.2.1 genomic_reference:SL2.40ch03 gene_region:58735379-58738091 transcript_region:SL2.40ch03:58735379..58738091+ go_terms:GO:0003948 functional_description:"N(4)-(Beta-N- acetylglucosaminyl)-L-asparaginase (AHRD V1 **** ASPG_ELIMR); contains Interpro domain(s) IPR000246 Peptidase T2, asparaginase 2 " | 9E-16 |
| 850 | Contig 850 | 530 | 1 | GH203974 | Solyc07g019460.2.1 genomic_reference:SL2.40ch07 gene_region:11523530-11529218 transcript_region:SL2.40ch07:11523530..11529218- go_terms:GO:0003958 functional_description:"Cytochrome P450 NADPH-reductase (AHRD V1 **** B3RFK3_PETHY); contains Interpro domain(s) IPR015702 NADPH Cytochrome P450 Reductase " | 5E-80 |
| 851 | Contig 851 | 515 | 1 | GH205101 | Solyc11g067100.1.1 evidence_code:10F1H0E1IEG genomic_reference:SL2.40ch11 gene_region:49928225-49929780 transcript_region:SL2.40ch11:49928225..49929780- go_terms:GO:0005840 functional_description:"60s acidic ribosomal protein-like protein (AHRD V1 ***- Q3HVP0_SOLTU); contains Interpro domain(s) IPR001813 Ribosomal protein 60S " | 9E-23 |
| 852 | Contig 852 | 899 | 1 | GH204161 | Solyc01g005620.2.1 genomic_reference:SL2.40ch01 gene_region:430522-435101 transcript_region:SL2.40ch01:430522..435101- go_terms:GO:0015367 functional_description:"Mitochondrial 2-oxoglutarate/malate carrier protein (AHRD V1 **** Q9FSF4_TOBAC); contains Interpro domain(s) IPR001993 Mitochondrial substrate carrier " | 1E-148 |
| 853 | Contig 853 | 501 | 1 | GH204630 | Solyc02g032950.2.1 genomic_reference:SL2.40ch02 gene_region:20198127-20200157 transcript_region:SL2.40ch02:20198127..20200157- go_terms:GO:0005515,GO:0010843 functional_description:"Transcription factor WRKY (AHRD V1 ***- C7E5X8_CAPAN); contains Interpro domain(s) IPR003657 DNA-binding WRKY " | 3E-90 |
| 854 | Contig 854 | 382 | 1 | GH204927 | Solyc03g096080.2.1 genomic_reference:SL2.40ch03 gene_region:51376491-51382146 transcript_region:SL2.40ch03:51376491..51382146+ go_terms:GO:0008270 functional_description:"Yippee zinc-binding-like protein (AHRD V1 **** C1FDK9_9CHLO); contains Interpro domain(s) IPR004910 Yippee-like protein " | 4E-41 |
| 855 | Contig 855 | 470 | 1 | GH204099 | Solyc03g083730.1.1 evidence_code:10F0H1E1IEG genomic_reference:SL2.40ch03 gene_region:47216694-47217299 transcript_region:SL2.40ch03:47216694..47217299+ go_terms:GO:0030599 functional_description:"Pectinesterase (AHRD V1 ***- C0PST8_PICSI); contains Interpro domain(s) IPR006501 Pectinesterase inhibitor " | 1E-69 |
| 856 | Contig 856 | 435 | 1 | GH204251 | Solyc03g031920.2.1 genomic_reference:SL2.40ch03 gene_region:8657524-8664052 transcript_region:SL2.40ch03:8657524..8664052- go_terms:GO:0015198 functional_description:"Yellow stripe-like protein 2.1 (Fragment) (AHRD V1 ***- B4ZYE4_BRAJU); contains Interpro domain(s) IPR004813 Oligopeptide transporter OPT superfamily " | 4E-52 |
| 857 | Contig 857 | 178 | 1 | GH205078 | ********* |  |
| 858 | Contig 858 | 299 | 1 | GH204634 | Solyc02g080640.2.1 genomic_reference:SL2.40ch02 gene_region:39406300-39409809 transcript_region:SL2.40ch02:39406300..39409809+ go_terms:GO:0009973 functional_description:"Phosphoadenosine phosphosulfate reductase (AHRD V1 *-** B7I7M3_ACIB5); contains Interpro domain(s) IPR013766 Thioredoxin domain " | 1E-49 |
| 859 | Contig 859 | 238 | 1 | GH205107 | Solyc07g056150.2.1 genomic_reference:SL2.40ch07 gene_region:61318385-61322676 transcript_region:SL2.40ch07:61318385..61322676- go_terms:GO:0015031,GO:0007264,GO:0005525,GO:0007165, GO:0005622 functional_description:"Ras-related protein Rab-2-A (AHRD V1 ***- B6T1U8_MAIZE); contains Interpro domain(s) IPR003579 Ras small GTPase, Rab type " | 5E-35 |
| 860 | Contig 860 | 754 | 1 | GH204629 | Solyc01g094940.2.1 genomic_reference:SL2.40ch01 gene_region:78103755-78107420 transcript_region:SL2.40ch01:78103755..78107420+ go_terms:GO:0006468 functional_description:"Receptor-like protein kinase (AHRD V1 ***- Q9LYS5_ARATH); contains Interpro domain(s) IPR002290 Serine/threonine protein kinase " | 1E-144 |
| 861 | Contig 861 | 485 | 1 | GH204554 | ********* |  |
| 862 | Contig 862 | 202 | 1 | GH203677 | Solyc05g012380.2.1 genomic_reference:SL2.40ch05 gene_region:5639430-5642259 transcript_region:SL2.40ch05:5639430..5642259- functional_description:"Glucan endo-1 3-beta-glucosidase 1 (AHRD V1 *-*- B6TW10_MAIZE); contains Interpro domain(s) IPR012946 X8 " | 9E-25 |
| 863 | Contig 863 | 162 | 1 | GH203750 | Solyc07g049720.2.1 genomic_reference:SL2.40ch07 gene_region:57386210-57396174 transcript_region:SL2.40ch07:57386210..57396174- go_terms:GO:0003917 functional_description:"DNA topoisomerase I (AHRD V1 **** Q9XGL1_DAUCA); contains Interpro domain(s) IPR013499 DNA topoisomerase I, C-terminal, eukaryotic-type " | 1E-25 |
| 864 | Contig 864 | 403 | 1 | GH204642 | ********* |  |
| 865 | Contig 865 | 895 | 1 | GH203707 | Solyc10g008520.2.1 genomic_reference:SL2.40ch10 gene_region:2637742-2643134 transcript_region:SL2.40ch10:2637742..2643134+ go_terms:GO:0080123,GO:0070566 functional_description:"Auxin-responsive GH3-like (AHRD V1 ***- Q0GUM1_ARATH); contains Interpro domain(s) IPR004993 GH3 auxin-responsive promoter " | 1E-172 |
| 866 | Contig 866 | 219 | 1 | GH203698 | Solyc10g081530.1.1 evidence_code:10F1H1E1IEG genomic_reference:SL2.40ch10 gene_region:61897645-61902538 transcript_region:SL2.40ch10:61897645..61902538- go_terms:GO:0033179,GO:0033177 functional_description:"V-type proton ATPase subunit d 1 (AHRD V1 ***- D3PHZ2_9MAXI); contains Interpro domain(s) IPR016727 ATPase, V0 complex, subunit D " | 2E-39 |
| 867 | Contig 867 | 172 | 1 | GH204627 | ********* |  |
| 868 | Contig 868 | 285 | 1 | GH203692 | ********* |  |
| 869 | Contig 869 | 634 | 1 | GH204703 | Solyc10g084320.1.1 evidence_code:10F1H1E1IEG genomic_reference:SL2.40ch10 gene_region:63240593-63242908 transcript_region:SL2.40ch10:63240593..63242908+ go_terms:GO:0006508,GO:0043086 functional_description:"Subtilisin-like protease (AHRD V1 **-- O82777_SOLLC); contains Interpro domain(s) IPR015500 Peptidase S8, subtilisin-related " | 1E-95 |
| 870 | Contig 870 | 212 | 1 | GH204552 | Solyc06g034370.1.1 evidence_code:10F0H1E1IEG genomic_reference:SL2.40ch06 gene_region:20869116-20869694 transcript_region:SL2.40ch06:20869116..20869694- go_terms:GO:0030599 functional_description:"Pectinesterase (AHRD V1 ***- B9T3X5_RICCO); contains Interpro domain(s) IPR006501 Pectinesterase inhibitor " | 7E-11 |
| 871 | Contig 871 | 472 | 1 | GH203722 | ********* |  |
| 872 | Contig 872 | 126 | 1 | GH203732 | Solyc10g005830.2.1 genomic_reference:SL2.40ch10 gene_region:646209-647697 transcript_region:SL2.40ch10:646209..647697+ functional_description:"Homoserine dehydrogenase-like (AHRD V1 ***- Q5QLC2_ORYSJ)" | 8E-21 |
| 873 | Contig 873 | 476 | 1 | GH203891 | Solyc07g007350.1.1 evidence_code:10F0H1E1IEG genomic_reference:SL2.40ch07 gene_region:2091112-2091879 transcript_region:SL2.40ch07:2091112..2091879- functional_description:"Jp18 (AHRD V1 *-*- Q8H6R4_PONTR); contains Interpro domain(s) IPR010993 Sterile alpha motif homology " | 8E-36 |
| 874 | Contig 874 | 174 | 1 | GH205181 | Solyc10g080610.1.1 evidence_code:10F1H1E1IEG genomic_reference:SL2.40ch10 gene_region:61166125-61167159 transcript_region:SL2.40ch10:61166125..61167159+ functional_description:"Kelch-like protein 14 (AHRD V1 ***- B6T7I5_MAIZE); contains Interpro domain(s) IPR015915 Kelch-type beta propeller " | 2E-29 |
| 875 | Contig 875 | 310 | 1 | GH203769 | Solyc06g063370.2.1 genomic_reference:SL2.40ch06 gene_region:36437291-36439451 transcript_region:SL2.40ch06:36437291..36439451- go_terms:GO:0016020 functional_description:"Chlorophyll a-b binding protein 1A, chloroplastic (AHRD V1 ***- CB2A_PYRPY); contains Interpro domain(s) IPR001344 Chlorophyll A-B binding protein " | 2E-49 |
| 876 | Contig 876 | 477 | 1 | GH204058 | Solyc03g121710.2.1 genomic_reference:SL2.40ch03 gene_region:63844015-63848152 transcript_region:SL2.40ch03:63844015..63848152+ go_terms:GO:0043047 functional_description:"RNA-binding motif, single-stranded-interacting protein 1 (AHRD V1 *--* RBMS1_HUMAN); contains Interpro domain(s) IPR000504 RNA recognition motif, RNP-1 " | 1E-58 |
| 877 | Contig 877 | 367 | 1 | GH203724 | Solyc11g066890.1.1 evidence_code:10F0H1E1IEG genomic_reference:SL2.40ch11 gene_region:49772513-49773796 transcript_region:SL2.40ch11:49772513..49773796- go_terms:GO:0009094 functional_description:"Prephenate dehydratase (AHRD V1 ***- Q6JJ29_IPOTF); contains Interpro domain(s) IPR001086 Prephenate dehydratase " | 4E-65 |
| 878 | Contig 878 | 574 | 1 | GH203710 | Solyc06g008880.2.1 genomic_reference:SL2.40ch06 gene_region:2824969-2831802 transcript_region:SL2.40ch06:2824969..2831802+ go_terms:GO:0005112,GO:0042923 functional_description:"WD repeat protein 23 (AHRD V1 **-- B0WXG6_CULQU); contains Interpro domain(s) IPR020472 G-protein beta WD-40 repeat, region " | 1E-111 |
| 879 | Contig 879 | 245 | 1 | GH205189 | Solyc03g025340.1.1 evidence_code:10F1H1E1IEG genomic_reference:SL2.40ch03 gene_region:7153156-7154205 transcript_region:SL2.40ch03:7153156..7154205- functional_description:"C2 domain-containing protein (AHRD V1 *--- Q5DVL6_HORVD); contains Interpro domain(s) IPR018029 C2 membrane targeting protein " | 2E-38 |
| 880 | Contig 880 | 293 | 1 | GH204690 | ********* |  |
| 881 | Contig 881 | 591 | 1 | GH204689 | Solyc04g081330.2.1 genomic_reference:SL2.40ch04 gene_region:62945005-62948114 transcript_region:SL2.40ch04:62945005..62948114- functional_description:"Chromosome 11 contig 1 DNA sequence. (Fragment) (AHRD V1 *-*- Q00Z75_OSTTA)" | 1E-102 |
| 882 | Contig 882 | 300 | 1 | GH203776 | Solyc04g054480.2.1 genomic_reference:SL2.40ch04 gene_region:51734576-51744000 transcript_region:SL2.40ch04:51734576..51744000+ go_terms:GO:0005488 functional_description:"C2 domain-containing protein-like (AHRD V1 *-*- Q67UI5_ORYSJ); contains Interpro domain(s) IPR011989 Armadillo-like helical " | 2E-51 |
| 883 | Contig 883 | 360 | 1 | GH203773 | Solyc03g118670.2.1 genomic_reference:SL2.40ch03 gene_region:61583911-61590920 transcript_region:SL2.40ch03:61583911..61590920+ go_terms:GO:0005515 functional_description:"Protein sel-1 homolog 2 (AHRD V1 **-- SE1L2_HUMAN); contains Interpro domain(s) IPR011990 Tetratricopeptide-like helical " | 2E-65 |
| 884 | Contig 884 | 175 | 1 | GH205166 | ********* |  |
| 885 | Contig 885 | 640 | 1 | GH204348 | Solyc11g018560.1.1 evidence_code:10F1H1E1IEG genomic_reference:SL2.40ch11 gene_region:8721428-8726834 transcript_region:SL2.40ch11:8721428..8726834+ go_terms:GO:0005840,GO:0006412 functional_description:"50S ribosomal protein L25 (AHRD V1 ***- D2LIE4_RHOVA); contains Interpro domain(s) IPR011035 Ribosomal protein L25/Gln-tRNA synthetase, anti-codon-binding domain " | 7E-98 |
| 886 | Contig 886 | 187 | 1 | GH204561 | ********* |  |
| 887 | Contig 887 | 412 | 1 | GH205175 | Solyc01g094080.2.1 genomic_reference:SL2.40ch01 gene_region:77420534-77423172 transcript_region:SL2.40ch01:77420534..77423172- go_terms:GO:0019825 functional_description:"Cytochrome P450" | 4E-74 |
| 888 | Contig 888 | 370 | 1 | GH203682 | Solyc03g112340.1.1 evidence_code:10F0H1E1IEG genomic_reference:SL2.40ch03 gene_region:56820717-56821913 transcript_region:SL2.40ch03:56820717..56821913+ go_terms:GO:0004842 functional_description:"Ring H2 finger protein (AHRD V1 *-*- D9ZHD8_HYPPE); contains Interpro domain(s) IPR018957 Zinc finger, C3HC4 RING-type " | 3E-52 |
| 889 | Contig 889 | 419 | 1 | GH203699 | Solyc06g068870.2.1 genomic_reference:SL2.40ch06 gene_region:39083842-39089281 transcript_region:SL2.40ch06:39083842..39089281- go_terms:GO:0016563,GO:0003700 functional_description:"BHLH transcription factor (AHRD V1 *-** A9YWR2_MEDTR); contains Interpro domain(s) IPR011598 Helix-loop-helix DNA-binding " | 7E-66 |
| 890 | Contig 890 | 435 | 1 | GH205200 | Solyc11g008320.1.1 evidence_code:10F0H1E1IEG genomic_reference:SL2.40ch11 gene_region:2523911-2528684 transcript_region:SL2.40ch11:2523911..2528684- go_terms:GO:0003677 functional_description:"Lysine-specific histone demethylase 1 (AHRD V1 *--- B2WC79_PYRTR); contains Interpro domain(s) IPR002937 Amine oxidase " | 4E-81 |
| 891 | Contig 891 | 103 | 1 | GH204684 | ********* |  |
| 892 | Contig 892 | 728 | 1 | GH205202 | Solyc04g082020.2.1 genomic_reference:SL2.40ch04 gene_region:63421329-63424627 transcript_region:SL2.40ch04:63421329..63424627- functional_description:"DNL zinc finger family protein (AHRD V1 **-- B6TGI7_MAIZE); contains Interpro domain(s) IPR007853 Zinc finger, Zim17-type " | 1E-113 |
| 893 | Contig 893 | 416 | 1 | GH203721 | Solyc07g005510.2.1 genomic_reference:SL2.40ch07 gene_region:400656-406292 transcript_region:SL2.40ch07:400656..406292+ go_terms:GO:0045485 functional_description:"Omega-6 fatty acid desaturase (AHRD V1 **** D7MBK3_ARALY); contains Interpro domain(s) IPR005804 Fatty acid desaturase, type 1 " | 2E-26 |
| 894 | Contig 894 | 336 | 1 | GH203714 | Solyc01g080140.2.1 genomic_reference:SL2.40ch01 gene_region:71805945-71820466 transcript_region:SL2.40ch01:71805945..71820466- functional_description:"Dolichol-phosphate mannosyltransferase (AHRD V1 **-- C0NU33_AJECG); contains Interpro domain(s) IPR013744 Protein of unknown function DUF1749 " | 5E-51 |
| 895 | Contig 895 | 697 | 1 | GH205173 | Solyc01g099400.2.1 genomic_reference:SL2.40ch01 gene_region:81429273-81435009 transcript_region:SL2.40ch01:81429273..81435009+ go_terms:GO:0032266 functional_description:"WD-repeat domain phosphoinositide-interacting protein 3 (AHRD V1 **-- B6SUF7_MAIZE); contains Interpro domain(s) IPR017986 WD40 repeat, region " | 2E-97 |
| 896 | Contig 896 | 572 | 1 | GH203790 | Solyc03g117980.2.1 genomic_reference:SL2.40ch03 gene_region:61025937-61031702 transcript_region:SL2.40ch03:61025937..61031702+ go_terms:GO:0016174 functional_description:"Respiratory burst oxidase-like protein (AHRD V1 **** C1IHQ9_9ROSI); contains Interpro domain(s) IPR013121 Ferric reductase, NAD binding " | 1E-99 |
| 897 | Contig 897 | 516 | 1 | GH203675 | Solyc03g123370.2.1 genomic_reference:SL2.40ch03 gene_region:64308451-64312367 transcript_region:SL2.40ch03:64308451..64312367+ go_terms:GO:0008728 functional_description:"(P)ppGpp synthetase I (GTP pyrophosphokinase) SpoT/RelA (AHRD V1 *--* Q2RHV7_MOOTA); contains Interpro domain(s) IPR007685 RelA/SpoT " | 5E-77 |
| 898 | Contig 898 | 522 | 1 | GH203785 | Solyc06g053200.2.1 genomic_reference:SL2.40ch06 gene_region:32435446-32437176 transcript_region:SL2.40ch06:32435446..32437176- go_terms:GO:0017057 functional_description:"6-phosphogluconolactonase (AHRD V1 **** B6UAK0_MAIZE); contains Interpro domain(s) IPR005900 6-phosphogluconolactonase " | 5E-93 |
| 899 | Contig 899 | 484 | 1 | GH204954 | Solyc02g078560.2.1 genomic_reference:SL2.40ch02 gene_region:37801010-37811610 transcript_region:SL2.40ch02:37801010..37811610+ functional_description:"Wings apart-like CG3707-RB isoform B (Fragment) (AHRD V1 *-*- B5AID2_DROME)" | 4E-37 |
| 900 | Contig 900 | 565 | 1 | GH204277 | Solyc06g007540.2.1 genomic_reference:SL2.40ch06 gene_region:1520884-1525347 transcript_region:SL2.40ch06:1520884..1525347- go_terms:GO:0008121 functional_description:"Cytochrome b-c1 complex subunit 8 (AHRD V1 **** D6BQP2_9ROSI); contains Interpro domain(s) IPR020101 Cytochrome b-c1 complex, subunit 8 " | 9E-37 |
| 901 | Contig 901 | 752 | 1 | GH205040 | Solyc05g018650.2.1 genomic_reference:SL2.40ch05 gene_region:22680872-22699780 transcript_region:SL2.40ch05:22680872..22699780- go_terms:GO:0004325 functional_description:"Ferrochelatase (AHRD V1 **** O64391_SOLTU); contains Interpro domain(s) IPR001015 Ferrochelatase " | 1E-130 |
| 902 | Contig 902 | 543 | 1 | GH205056 | Solyc09g082060.2.1 genomic_reference:SL2.40ch09 gene_region:63298831-63303187 transcript_region:SL2.40ch09:63298831..63303187+ go_terms:GO:0004124 functional_description:"Cysteine synthase (AHRD V1 **** Q3LAG5_TOBAC); contains Interpro domain(s) IPR005859 Cysteine synthase A " | 2E-34 |
| 903 | Contig 903 | 309 | 1 | GH203770 | Solyc07g062570.2.1 genomic_reference:SL2.40ch07 gene_region:62472541-62478080 transcript_region:SL2.40ch07:62472541..62478080+ go_terms:GO:0043130 functional_description:"Ubiquitin-conjugating enzyme E2 N (AHRD V1 **** B5XC59_SALSA); contains Interpro domain(s) IPR000608 Ubiquitin-conjugating enzyme, E2 " | 1E-22 |
| 904 | Contig 904 | 328 | 1 | GH205219 | Solyc06g072280.2.1 genomic_reference:SL2.40ch06 gene_region:40954052-40956307 transcript_region:SL2.40ch06:40954052..40956307+ go_terms:GO:0005515 functional_description:"Small nuclear ribonucleoprotein E (AHRD V1 ***- B6SK95_MAIZE); contains Interpro domain(s) IPR006649 Like-Sm ribonucleoprotein, eukaryotic and archaea-type, core " | 6E-16 |
| 905 | Contig 905 | 833 | 1 | GH204243 | Solyc01g096450.2.1 genomic_reference:SL2.40ch01 gene_region:79304614-79307810 transcript_region:SL2.40ch01:79304614..79307810+ go_terms:GO:0006508,GO:0031177 functional_description:"Aspartic proteinase nepenthesin-1 (AHRD V1 **-- B6TDX6_MAIZE); contains Interpro domain(s) IPR001461 Peptidase A1 " | 3E-77 |
| 906 | Contig 906 | 844 | 1 | GH204381 | Solyc05g053610.2.1 genomic_reference:SL2.40ch05 gene_region:62822197-62829504 transcript_region:SL2.40ch05:62822197..62829504- go_terms:GO:0042626 functional_description:"ATP-binding cassette transporter (AHRD V1 ***- D8RL93_SELML); contains Interpro domain(s) IPR013525 ABC-2 type transporter " | 2E-95 |
| 907 | Contig 907 | 477 | 1 | GH203478 | Solyc12g014470.1.1 evidence_code:10F0H1E1IEG genomic_reference:SL2.40ch12 gene_region:5447836-5450290 transcript_region:SL2.40ch12:5447836..5450290+ go_terms:GO:0005524,GO:0005507 functional_description:"Protein grpE (AHRD V1 *-*- Q1QRU3_NITHX); contains Interpro domain(s) IPR000740 GrpE nucleotide exchange factor " | 1E-62 |
| 908 | Contig 908 | 892 | 1 | GH203498 | Solyc05g051050.2.1 genomic_reference:SL2.40ch05 gene_region:60379685-60383579 transcript_region:SL2.40ch05:60379685..60383579- go_terms:GO:0004696 functional_description:"Glycogen synthase kinase (AHRD V1 **** Q1AMT7_ORYSJ); contains Interpro domain(s) IPR002290 Serine/threonine protein kinase " | 5E-38 |
| 909 | Contig 909 | 646 | 1 | GH205193 | Solyc08g083350.2.1 genomic_reference:SL2.40ch08 gene_region:62993669-62996264 transcript_region:SL2.40ch08:62993669..62996264+ go_terms:GO:0003735,GO:0005515 functional_description:"50S ribosomal protein L11 (AHRD V1 ***- B6U1J2_MAIZE); contains Interpro domain(s) IPR006519 Ribosomal protein L11, bacterial-type " | 4E-87 |
| 910 | Contig 910 | 545 | 1 | GH205204 | Solyc01g009990.2.1 genomic_reference:SL2.40ch01 gene_region:4594127-4598093 transcript_region:SL2.40ch01:4594127..4598093+ go_terms:GO:0006457 functional_description:"Peptidyl-prolyl cis-trans isomerase (AHRD V1 ***- B9RMA5_RICCO); contains Interpro domain(s) IPR002130 Peptidyl-prolyl cis-trans isomerase, cyclophilin-type " | 9E-45 |
| 911 | Contig 911 | 385 | 1 | GH204254 | Solyc08g063040.2.1 genomic_reference:SL2.40ch08 gene_region:49787174-49790203 transcript_region:SL2.40ch08:49787174..49790203+ go_terms:GO:0005622 functional_description:"Zinc finger protein (AHRD V1 *-*- Q9LVQ7_ARATH); contains Interpro domain(s) IPR007087 Zinc finger, C2H2-type " | 2E-59 |
| 912 | Contig 912 | 430 | 1 | GH204405 | Solyc03g111830.2.1 genomic_reference:SL2.40ch03 gene_region:56497562-56502317 transcript_region:SL2.40ch03:56497562..56502317+ go_terms:GO:0004719,GO:0042802 functional_description:"Protein-L-isoaspartate O-methyltransferase (AHRD V1 **** B9I3I9_POPTR); contains Interpro domain(s) IPR000682 Protein-L-isoaspartate(D-aspartate) O-methyltransferase " | 2E-80 |
| 913 | Contig 913 | 124 | 1 | GH204388 | ********* |  |
| 914 | Contig 914 | 338 | 1 | GH203827 | Solyc01g091730.2.1 genomic_reference:SL2.40ch01 gene_region:77079559-77081265 transcript_region:SL2.40ch01:77079559..77081265- go_terms:GO:0005778 functional_description:"Peroxisomal membrane protein 11-5 (AHRD V1 ***- PX115_ORYSJ); contains Interpro domain(s) IPR008733 Peroxisomal biogenesis factor 11 " | 2E-23 |
| 915 | Contig 915 | 276 | 1 | GH204932 | Solyc01g098550.2.1 genomic_reference:SL2.40ch01 gene_region:80797121-80803117 transcript_region:SL2.40ch01:80797121..80803117+ go_terms:GO:0008152,GO:0006568 functional_description:"Tryptophan synthase alpha chain (AHRD V1 ***- Q8LA83_ARATH); contains Interpro domain(s) IPR002028 Tryptophan synthase, alpha chain " | 6E-12 |
| 916 | Contig 916 | 788 | 1 | GH203972 | Solyc01g107630.2.1 genomic_reference:SL2.40ch01 gene_region:86878462-86880092 transcript_region:SL2.40ch01:86878462..86880092+ go_terms:GO:0005515 functional_description:"Cell division cycle protein 123-like protein (AHRD V1 ***- B6S349_9BILA); contains Interpro domain(s) IPR009772 D123 " | 1E-129 |
| 917 | Contig 917 | 638 | 1 | GH204999 | Solyc06g005170.2.1 genomic_reference:SL2.40ch06 gene_region:192235-195321 transcript_region:SL2.40ch06:192235..195321+ go_terms:GO:0016908 functional_description:"Mitogen-activated protein kinase 3 (AHRD V1 **** Q84MI4_SOLLC); contains Interpro domain(s) IPR002290 Serine/threonine protein kinase " | 2E-68 |
| 918 | Contig 918 | 528 | 1 | GH204192 | Solyc12g096700.1.1 evidence_code:10F1H1E1IEG genomic_reference:SL2.40ch12 gene_region:63855944-63857858 transcript_region:SL2.40ch12:63855944..63857858- go_terms:GO:0005840 functional_description:"Ribosomal L9-like protein (AHRD V1 ***- B3TLR1_ELAGV); contains Interpro domain(s) IPR002359 Ribosomal protein L6, conserved site-2 " | 8E-53 |
| 919 | Contig 919 | 680 | 1 | GH205203 | Solyc08g067090.2.1 genomic_reference:SL2.40ch08 gene_region:53210448-53219760 transcript_region:SL2.40ch08:53210448..53219760+ go_terms:GO:0006457 functional_description:"Peptidyl-prolyl cis-trans isomerase (AHRD V1 *-*- B9H8E5_POPTR); contains Interpro domain(s) IPR002130 Peptidyl-prolyl cis-trans isomerase, cyclophilin-type " | 2E-71 |
| 920 | Contig 920 | 310 | 1 | GH204377 | Solyc01g091070.2.1 genomic_reference:SL2.40ch01 gene_region:76550313-76559336 transcript_region:SL2.40ch01:76550313..76559336+ go_terms:GO:0006508,GO:0009987 functional_description:"Methionine aminopeptidase (AHRD V1 ***- B9RD92_RICCO); contains Interpro domain(s) IPR002467 Peptidase M24A, methionine aminopeptidase, subfamily 1 " | 5E-58 |
| 921 | Contig 921 | 749 | 1 | GH204322 | Solyc01g108230.2.1 genomic_reference:SL2.40ch01 gene_region:87342405-87350641 transcript_region:SL2.40ch01:87342405..87350641+ go_terms:GO:0050291 functional_description:"LAG1 longevity assurance homolog 2 (AHRD V1 **-- C1BLI2_OSMMO); contains Interpro domain(s) IPR006634 TRAM, LAG1 and CLN8 homology " | 7E-87 |
| 922 | Contig 922 | 518 | 1 | GH205105 | Solyc01g111710.2.1 genomic_reference:SL2.40ch01 gene_region:89651034-89655488 transcript_region:SL2.40ch01:89651034..89655488- go_terms:GO:0005515 functional_description:"26S proteasome non-ATPase regulatory subunit 3 (AHRD V1 ***- B6TBG8_MAIZE); contains Interpro domain(s) IPR013143 PCI/PINT associated module " | 9E-89 |
| 923 | Contig 923 | 420 | 1 | GH203687 | Solyc02g092670.1.1 evidence_code:10F1H1E1IEG genomic_reference:SL2.40ch02 gene_region:48256556-48258820 transcript_region:SL2.40ch02:48256556..48258820- go_terms:GO:0004252 functional_description:"Subtilisin-like protease (AHRD V1 ***- A9XG40_TOBAC); contains Interpro domain(s) IPR015500 Peptidase S8, subtilisin-related " | 7E-40 |
| 924 | Contig 924 | 442 | 1 | GH204969 | ********* |  |
| 925 | Contig 925 | 555 | 1 | GH204650 | Solyc08g077480.2.1 genomic_reference:SL2.40ch08 gene_region:58599749-58601339 transcript_region:SL2.40ch08:58599749..58601339+ functional_description:"Os04g0585900 protein (Fragment) (AHRD V1 *-*- Q0JAP2_ORYSJ); contains Interpro domain(s) IPR007650 Protein of unknown function DUF581 " | 1E-11 |
| 926 | Contig 926 | 781 | 1 | GH204660 | Solyc06g007980.2.1 genomic_reference:SL2.40ch06 gene_region:1835086-1845250 transcript_region:SL2.40ch06:1835086..1845250+ go_terms:GO:0030259 functional_description:"Sterol 3-beta-glucosyltransferase (AHRD V1 ***- B6U4Q7_MAIZE); contains Interpro domain(s) IPR004276 Glycosyl transferase, family 28 " | 1E-146 |
| 927 | Contig 927 | 373 | 1 | GH203996 | Solyc07g053170.2.1 genomic_reference:SL2.40ch07 gene_region:58941217-58949834 transcript_region:SL2.40ch07:58941217..58949834- go_terms:GO:0019900,GO:0004709 functional_description:"Protein serine/threonine kinase (AHRD V1 *-** D3BP85_POLPA); contains Interpro domain(s) IPR002290 Serine/threonine protein kinase " | 9E-53 |
| 928 | Contig 928 | 615 | 1 | GH205197 | Solyc03g095770.2.1 genomic_reference:SL2.40ch03 gene_region:50421318-50423492 transcript_region:SL2.40ch03:50421318..50423492+ go_terms:GO:0003700 functional_description:"WRKY transcription factor 6 (AHRD V1 **** A7UGD3_SOLTU); contains Interpro domain(s) IPR003657 DNA-binding WRKY " | 1E-113 |
| 929 | Contig 929 | 562 | 1 | GH203639 | Solyc04g005820.2.1 genomic_reference:SL2.40ch04 gene_region:524032-528627 transcript_region:SL2.40ch04:524032..528627- go_terms:GO:0051082 functional_description:"Chaperone protein dnaJ 2 (AHRD V1 ***- DNAJ2_ARATH); contains Interpro domain(s) IPR003095 Heat shock protein DnaJ " | 2E-97 |
| 930 | Contig 930 | 558 | 1 | GH204345 | Solyc02g068380.2.1 genomic_reference:SL2.40ch02 gene_region:32909088-32915018 transcript_region:SL2.40ch02:32909088..32915018- go_terms:GO:0008233,GO:0047652 functional_description:"N-carbamoyl-L-amino-acid amidohydrolase (AHRD V1 **-- Q1LHR1_RALME); contains Interpro domain(s) IPR010158 Amidase, hydantoinase/carbamoylase " | 7E-79 |
| 931 | Contig 931 | 463 | 1 | GH204607 | Solyc02g082130.1.1 evidence_code:10F0H1E1IEG genomic_reference:SL2.40ch02 gene_region:40430556-40431629 transcript_region:SL2.40ch02:40430556..40431629- functional_description:"Unknown Protein (AHRD V1); contains Interpro domain(s) IPR007019 Surfeit locus 6 " | 4E-50 |
| 932 | Contig 932 | 283 | 1 | GH204539 | Solyc03g019890.2.1 genomic_reference:SL2.40ch03 gene_region:6781354-6790955 transcript_region:SL2.40ch03:6781354..6790955+ go_terms:GO:0005515 functional_description:"Beta-galactosidase (AHRD V1 ***- A2JGX1_SOLLC); contains Interpro domain(s) IPR001944 Glycoside hydrolase, family 35 " | 4E-52 |
| 933 | Contig 933 | 673 | 1 | GH205191 | Solyc01g105340.2.1 genomic_reference:SL2.40ch01 gene_region:85301588-85307119 transcript_region:SL2.40ch01:85301588..85307119+ go_terms:GO:0006457,GO:0009408,GO:0031072 functional_description:"Chaperone protein dnaJ (AHRD V1 ***- B6TAJ2_MAIZE); contains Interpro domain(s) IPR012724 Chaperone DnaJ " | 9E-56 |
| 934 | Contig 934 | 736 | 1 | GH203422 | Solyc08g078430.2.1 genomic_reference:SL2.40ch08 gene_region:59406043-59410273 transcript_region:SL2.40ch08:59406043..59410273- go_terms:GO:0005515,GO:0043021 functional_description:"Pre-mRNA processing ribonucleoprotein binding region-containing protein (AHRD V1 **-* A7AUH1_BABBO); contains Interpro domain(s) IPR019175 Prp31 C-terminal " | 4E-68 |
| 935 | Contig 935 | 728 | 1 | GH204197 | ********* |  |
| 936 | Contig 936 | 376 | 1 | GH204076 | Solyc01g104270.2.1 genomic_reference:SL2.40ch01 gene_region:84460019-84465624 transcript_region:SL2.40ch01:84460019..84465624+ go_terms:GO:0005524 functional_description:"Os12g0283800 protein (Fragment) (AHRD V1 *-*- Q0INW6_ORYSJ)" | 1E-21 |
| 937 | Contig 937 | 311 | 1 | GH204583 | Solyc06g050120.2.1 genomic_reference:SL2.40ch06 gene_region:28971861-28974038 transcript_region:SL2.40ch06:28971861..28974038+ go_terms:GO:0003735 functional_description:"Ribosomal protein L7a (AHRD V1 ***- A8J567_CHLRE); contains Interpro domain(s) IPR001921 Ribosomal protein L7A " | 2E-37 |
| 938 | Contig 938 | 584 | 1 | GH204168 | Solyc02g068410.1.1 evidence_code:10F0H1E0IEG genomic_reference:SL2.40ch02 gene_region:32938286-32942722 transcript_region:SL2.40ch02:32938286..32942722+ go_terms:GO:0004650 functional_description:"AT4G20050-like protein (Fragment) (AHRD V1 *-*- D6PQZ2_9BRAS); contains Interpro domain(s) IPR011050 Pectin lyase fold/virulence factor " | 2E-61 |
| 939 | Contig 939 | 479 | 1 | GH203502 | Solyc12g008370.1.1 evidence_code:10F0H1E1IEG genomic_reference:SL2.40ch12 gene_region:1799025-1802970 transcript_region:SL2.40ch12:1799025..1802970+ go_terms:GO:0005681 functional_description:"Pre-mRNA-processing protein 45 (AHRD V1 **-- D6RKF6_COPC7); contains Interpro domain(s) IPR017862 SKI-interacting protein, SKIP " | 8E-67 |
| 940 | Contig 940 | 787 | 1 | GH204237 | Solyc01g109850.2.1 genomic_reference:SL2.40ch01 gene_region:88449072-88454711 transcript_region:SL2.40ch01:88449072..88454711- go_terms:GO:0004040,GO:0008836 functional_description:"Diaminopimelate decarboxylase (AHRD V1 **** D2DKE7_SOYBN); contains Interpro domain(s) IPR002986 Diaminopimelate decarboxylase " | 1E-143 |
| 941 | Contig 941 | 591 | 1 | GH203862 | Solyc07g055950.2.1 genomic_reference:SL2.40ch07 gene_region:61193403-61195377 transcript_region:SL2.40ch07:61193403..61195377- functional_description:"Meiosis 5 (AHRD V1 *-*- B6T5X0_MAIZE)" | 2E-60 |
| 942 | Contig 942 | 547 | 1 | GH203492 | Solyc03g083390.2.1 genomic_reference:SL2.40ch03 gene_region:46783589-46788273 transcript_region:SL2.40ch03:46783589..46788273+ functional_description:"Nuclear movement protein nudc (AHRD V1 *-*- Q17KI6_AEDAE); contains Interpro domain(s) IPR017447 CS " | 1E-100 |
| 943 | Contig 943 | 517 | 1 | GH205006 | Solyc06g050730.2.1 genomic_reference:SL2.40ch06 gene_region:29994029-29995792 transcript_region:SL2.40ch06:29994029..29995792- functional_description:"Genomic DNA chromosome 5 TAC clone K18I23 (AHRD V1 *-*- Q9FLB8_ARATH); contains Interpro domain(s) IPR006597 Sel1-like " | 5E-71 |
| 944 | Contig 944 | 369 | 1 | GH203504 | Solyc04g077850.2.1 genomic_reference:SL2.40ch04 gene_region:60322096-60324505 transcript_region:SL2.40ch04:60322096..60324505+ go_terms:GO:0005488 functional_description:"U-box domain-containing protein 4 (AHRD V1 **-- PUB4_ARATH); contains Interpro domain(s) IPR011989 Armadillo-like helical " | 4E-24 |
| 945 | Contig 945 | 324 | 1 | GH204971 | ********* |  |
| 946 | Contig 946 | 190 | 1 | GH204802 | ********* |  |
| 947 | Contig 947 | 648 | 1 | GH203723 | Solyc07g005700.2.1 genomic_reference:SL2.40ch07 gene_region:577484-584465 transcript_region:SL2.40ch07:577484..584465- go_terms:GO:0008270 functional_description:"Unknown Protein (AHRD V1); contains Interpro domain(s) IPR003604 Zinc finger, U1-type " | 4E-61 |
| 948 | Contig 948 | 803 | 1 | GH204491 | Solyc01g091950.2.1 genomic_reference:SL2.40ch01 gene_region:77242437-77253671 transcript_region:SL2.40ch01:77242437..77253671+ go_terms:GO:0047184 functional_description:"1-acylglycerophosphocholine O-acyltransferase 1 (AHRD V1 **** PCAT1_DROME); contains Interpro domain(s) IPR002123 Phospholipid/glycerol acyltransferase " | 1E-110 |
| 949 | Contig 949 | 589 | 1 | GH204088 | Solyc03g096540.2.1 genomic_reference:SL2.40ch03 gene_region:52147104-52148707 transcript_region:SL2.40ch03:52147104..52148707- functional_description:"Wound/stress protein (AHRD V1 **-- Q672Q3_SOLLC); contains Interpro domain(s) IPR001024 Lipoxygenase, LH2 " | 1E-82 |
| 950 | Contig 950 | 661 | 1 | GH204383 | Solyc06g008620.1.1 evidence_code:10F1H1E1IEG genomic_reference:SL2.40ch06 gene_region:2517512-2519494 transcript_region:SL2.40ch06:2517512..2519494- functional_description:"Protein tolB (AHRD V1 *-*- D3SF14_THISK); contains Interpro domain(s) IPR011042 Six-bladed beta-propeller, TolB-like " | 1E-128 |
| 951 | Contig 951 | 490 | 1 | GH204012 | Solyc01g057770.2.1 genomic_reference:SL2.40ch01 gene_region:56576301-56580082 transcript_region:SL2.40ch01:56576301..56580082+ go_terms:GO:0005452 functional_description:"AE family transporter anion exchange (AHRD V1 *--* A4RY02_OSTLU); contains Interpro domain(s) IPR003020 Bicarbonate transporter, eukaryotic " | 8E-81 |
| 952 | Contig 952 | 628 | 1 | GH203622 | Solyc09g091740.2.1 genomic_reference:SL2.40ch09 gene_region:66314629-66317264 transcript_region:SL2.40ch09:66314629..66317264- go_terms:GO:0003735,GO:0005515 functional_description:"60S ribosomal protein L13a-like protein (AHRD V1 ***- Q3HRW1_SOLTU); contains Interpro domain(s) IPR005755 Ribosomal protein L13, eukaryotic/archaeal " | 1E-113 |
| 953 | Contig 953 | 760 | 1 | GH204900 | Solyc04g010020.2.1 genomic_reference:SL2.40ch04 gene_region:3333448-3338309 transcript_region:SL2.40ch04:3333448..3338309+ go_terms:GO:0003676,GO:0000166 functional_description:"RNA-binding protein (AHRD V1 *--- D3TPM8_GLOMM); contains Interpro domain(s) IPR015464 RNA recognition motif-related " | 3E-74 |
| 954 | Contig 954 | 401 | 1 | GH204765 | ********* |  |
| 955 | Contig 955 | 332 | 1 | GH204798 | Solyc06g084230.2.1 genomic_reference:SL2.40ch06 gene_region:45701450-45704382 transcript_region:SL2.40ch06:45701450..45704382- go_terms:GO:0031369 functional_description:"40S ribosomal protein S24 (AHRD V1 ***- C6TAW2_SOYBN); contains Interpro domain(s) IPR001976 Ribosomal protein S24e " | 3E-44 |
| 956 | Contig 956 | 908 | 1 | GH204070 | Solyc01g109300.2.1 genomic_reference:SL2.40ch01 gene_region:88049244-88054027 transcript_region:SL2.40ch01:88049244..88054027+ go_terms:GO:0055114 functional_description:"4-hydroxy-3-methylbut-2-enyl diphosphate reductase (AHRD V1 ***- A2TGW4_9LAMI); contains Interpro domain(s) IPR003451 LytB protein " | 1E-143 |
| 957 | Contig 957 | 201 | 1 | GH204944 | ********* |  |
| 958 | Contig 958 | 620 | 1 | GH204092 | Solyc10g078920.1.1 evidence_code:10F1H1E1IEG genomic_reference:SL2.40ch10 gene_region:59894275-59896223 transcript_region:SL2.40ch10:59894275..59896223+ go_terms:GO:0045454 functional_description:"Thioredoxin-like 5 (AHRD V1 ***- A6N1I7_ORYSI); contains Interpro domain(s) IPR013766 Thioredoxin domain " | 1E-100 |
| 959 | Contig 959 | 383 | 1 | GH203577 | Solyc03g117980.2.1 genomic_reference:SL2.40ch03 gene_region:61025937-61031702 transcript_region:SL2.40ch03:61025937..61031702+ go_terms:GO:0016174 functional_description:"Respiratory burst oxidase-like protein (AHRD V1 **** C1IHQ9_9ROSI); contains Interpro domain(s) IPR013121 Ferric reductase, NAD binding " | 3E-71 |
| 960 | Contig 960 | 245 | 1 | GH204202 | ********* |  |
| 961 | Contig 961 | 548 | 1 | GH204820 | Solyc01g009020.2.1 genomic_reference:SL2.40ch01 gene_region:3021936-3024369 transcript_region:SL2.40ch01:3021936..3024369- go_terms:GO:0004869,GO:0050897 functional_description:"Cysteine proteinase inhibitor (AHRD V1 **** Q2VY67_9ERIC); contains Interpro domain(s) IPR000010 Proteinase inhibitor I25, cystatin " | 4E-32 |
| 962 | Contig 962 | 587 | 1 | GH205003 | Solyc04g026100.1.1 evidence_code:10F1H1E1IEG genomic_reference:SL2.40ch04 gene_region:24590324-24590767 transcript_region:SL2.40ch04:24590324..24590767+ go_terms:GO:0032183,GO:0003723 functional_description:"30S ribosomal protein S9 (AHRD V1 ***- C5JX04_AJEDS); contains Interpro domain(s) IPR000754 Ribosomal protein S9 " | 4E-72 |
| 963 | Contig 963 | 776 | 1 | GH204529 | Solyc02g077410.2.1 genomic_reference:SL2.40ch02 gene_region:36930997-36935490 transcript_region:SL2.40ch02:36930997..36935490- functional_description:"heat-and acid-stable phosphoprotein (AHRD V1 *-*- B5X267_SALSA); contains Interpro domain(s) IPR019380 Casein kinase substrate, phosphoprotein PP28 " | 3E-47 |
| 964 | Contig 964 | 514 | 1 | GH203456 | Solyc03g118650.2.1 genomic_reference:SL2.40ch03 gene_region:61575132-61577276 transcript_region:SL2.40ch03:61575132..61577276+ go_terms:GO:0005515,GO:0004034 functional_description:"Aldose 1-epimerase-like protein (AHRD V1 **** Q9LVH6_ARATH); contains Interpro domain(s) IPR015443 Aldose-1-epimerase " | 3E-78 |
| 965 | Contig 965 | 481 | 1 | GH203694 | Solyc08g075380.2.1 genomic_reference:SL2.40ch08 gene_region:56680117-56686385 transcript_region:SL2.40ch08:56680117..56686385- functional_description:"F-box protein (AHRD V1 ***- A9Z0N6_PONTR); contains Interpro domain(s) IPR001810 Cyclin-like F-box " | 1E-94 |
| 966 | Contig 966 | 690 | 1 | GH204839 | Solyc06g050770.2.1 genomic_reference:SL2.40ch06 gene_region:30039629-30046725 transcript_region:SL2.40ch06:30039629..30046725- go_terms:GO:0005483 functional_description:"Alpha-soluble NSF attachment protein (AHRD V1 **** D2D2Z7_GOSHI); contains Interpro domain(s) IPR000744 NSF attachment protein " | 2E-85 |
| 967 | Contig 967 | 518 | 1 | GH204910 | Solyc01g110540.2.1 genomic_reference:SL2.40ch01 gene_region:88964018-88966597 transcript_region:SL2.40ch01:88964018..88966597+ functional_description:"Unknown Protein (AHRD V1)" | 4E-19 |
| 968 | Contig 968 | 415 | 1 | GH204508 | Solyc01g008270.2.1 genomic_reference:SL2.40ch01 gene_region:2385892-2400050 transcript_region:SL2.40ch01:2385892..2400050+ go_terms:GO:0015631,GO:0003714 functional_description:"Prefoldin subunit 5 (AHRD V1 ***- B6TVU2_MAIZE); contains Interpro domain(s) IPR011599 Prefoldin alpha subunit " | 2E-37 |
| 969 | Contig 969 | 437 | 1 | GH203814 | Solyc01g091580.2.1 genomic_reference:SL2.40ch01 gene_region:76934096-76939715 transcript_region:SL2.40ch01:76934096..76939715- go_terms:GO:0005525,GO:0017111,GO:0006614 functional_description:"Signal recognition particle-docking protein FtsY (AHRD V1 ***- A7H885_ANADF); contains Interpro domain(s) IPR004390 Cell division transporter substrate-binding protein FtsY " | 7E-38 |
| 970 | Contig 970 | 450 | 1 | GH203815 | Solyc07g047700.2.1 genomic_reference:SL2.40ch07 gene_region:56251359-56254694 transcript_region:SL2.40ch07:56251359..56254694+ functional_description:"UPF0382 membrane protein SE_0353 (AHRD V1 ***- C3KHX6_ANOFI); contains Interpro domain(s) IPR006696 Protein of unknown function DUF423 " | 4E-32 |
| 971 | Contig 971 | 607 | 1 | GH204096 | Solyc10g079740.1.1 evidence_code:10F0H1E1IEG genomic_reference:SL2.40ch10 gene_region:60563881-60566380 transcript_region:SL2.40ch10:60563881..60566380+ functional_description:"HAUS augmin-like complex subunit 1 (AHRD V1 ***- HAUS1_HUMAN)" | 1E-107 |
| 972 | Contig 972 | 517 | 1 | GH203771 | Solyc07g014700.2.1 genomic_reference:SL2.40ch07 gene_region:5107189-5117910 transcript_region:SL2.40ch07:5107189..5117910- go_terms:GO:0008250 functional_description:"Dolichyl- diphosphooligosaccharide--protein glycosyltransferase subunit 2 (AHRD V1 *--- RPN2_HUMAN); contains Interpro domain(s) IPR008814 Ribophorin II " | 2E-90 |
| 973 | Contig 973 | 783 | 1 | GH205309 | Solyc01g110120.2.1 genomic_reference:SL2.40ch01 gene_region:88653085-88663519 transcript_region:SL2.40ch01:88653085..88663519- go_terms:GO:0046961,GO:0009678 functional_description:"V-type proton ATPase subunit a (AHRD V1 **** VPH1_NEUCR); contains Interpro domain(s) IPR002490 ATPase, V0/A0 complex, 116-kDa subunit " | 1E-132 |
| 974 | Contig 974 | 779 | 1 | GH204672 | Solyc09g009190.2.1 genomic_reference:SL2.40ch09 gene_region:2539818-2558047 transcript_region:SL2.40ch09:2539818..2558047+ go_terms:GO:0003844 functional_description:"1 4-alpha-glucan branching enzyme II (AHRD V1 **** P93691_WHEAT); contains Interpro domain(s) IPR006589 Glycosyl hydrolase, family 13, subfamily, catalytic region " | 1E-105 |
| 975 | Contig 975 | 678 | 1 | GH204685 | Solyc03g031720.2.1 genomic_reference:SL2.40ch03 gene_region:8453484-8459499 transcript_region:SL2.40ch03:8453484..8459499- go_terms:GO:0005515 functional_description:"RNA Binding Protein 45 (AHRD V1 **** Q9LEB4_NICPL); contains Interpro domain(s) IPR000504 RNA recognition motif, RNP-1 " | 3E-37 |
| 976 | Contig 976 | 694 | 1 | GH204496 | Solyc11g044270.1.1 evidence_code:10F0H1E0IEG genomic_reference:SL2.40ch11 gene_region:35241307-35251540 transcript_region:SL2.40ch11:35241307..35251540- functional_description:"Sugar transporter (AHRD V1 *-*- D2V7E3_NAEGR); contains Interpro domain(s) IPR016196 Major facilitator superfamily, general substrate transporter " | 2E-28 |
| 977 | Contig 977 | 659 | 1 | GH204083 | ********* |  |
| 978 | Contig 978 | 586 | 1 | GH203763 | Solyc12g095950.1.1 evidence_code:10F0H0E1IEG genomic_reference:SL2.40ch12 gene_region:63400621-63400963 transcript_region:SL2.40ch12:63400621..63400963+ functional_description:"Unknown Protein (AHRD V1)" | 3E-17 |
| 979 | Contig 979 | 380 | 1 | GH203448 | Solyc01g087900.2.1 genomic_reference:SL2.40ch01 gene_region:74528983-74532682 transcript_region:SL2.40ch01:74528983..74532682- go_terms:GO:0004596,GO:0042802 functional_description:"N-alpha-acetyltransferase 20, NatB catalytic subunit (AHRD V1 **** NAA20_DICDI); contains Interpro domain(s) IPR000182 GCN5-related N-acetyltransferase " | 3E-68 |
| 980 | Contig 980 | 383 | 1 | GH203440 | Solyc06g069150.1.1 evidence_code:10F0H0E1IEG genomic_reference:SL2.40ch06 gene_region:39319761-39320498 transcript_region:SL2.40ch06:39319761..39320498+ functional_description:"Genomic DNA chromosome 3 P1 clone MPE11 (AHRD V1 *-*- Q9LUA5_ARATH)" | 5E-54 |
| 981 | Contig 981 | 318 | 1 | GH205375 | Solyc01g009520.2.1 genomic_reference:SL2.40ch01 gene_region:3780910-3784239 transcript_region:SL2.40ch01:3780910..3784239+ go_terms:GO:0032183,GO:0003735 functional_description:"Ribosomal protein (AHRD V1 ***- Q6ER67_ORYSJ); contains Interpro domain(s) IPR002143 Ribosomal protein L1 " | 6E-42 |
| 982 | Contig 982 | 282 | 1 | GH203451 | ********* |  |
| 983 | Contig 983 | 777 | 1 | GH205383 | Solyc09g076050.2.1 genomic_reference:SL2.40ch09 gene_region:63235327-63238938 transcript_region:SL2.40ch09:63235327..63238938+ functional_description:"FRIGIDA (Fragment) (AHRD V1 *--- B0Z034_ARALP); contains Interpro domain(s) IPR012474 Frigida-like " | 1E-138 |
| 984 | Contig 984 | 492 | 1 | GH203470 | Solyc09g064790.2.1 genomic_reference:SL2.40ch09 gene_region:57800521-57806263 transcript_region:SL2.40ch09:57800521..57806263+ functional_description:"PAC (Fragment) (AHRD V1 **-- Q7DLY1_ARATH)" | 9E-43 |
| 985 | Contig 985 | 202 | 1 | GH204570 | Solyc01g111630.2.1 genomic_reference:SL2.40ch01 gene_region:89609244-89612993 transcript_region:SL2.40ch01:89609244..89612993+ go_terms:GO:0008266,GO:0030267 functional_description:"Glyoxylate/hydroxypyruvate reductase B (AHRD V1 **** GHRB_YERPS); contains Interpro domain(s) IPR006140 D-isomer specific 2-hydroxyacid dehydrogenase, NAD-binding " | 9E-33 |
| 986 | Contig 986 | 465 | 1 | GH203445 | Solyc02g069720.2.1 genomic_reference:SL2.40ch02 gene_region:34117564-34122011 transcript_region:SL2.40ch02:34117564..34122011+ go_terms:GO:0003700 functional_description:"Zinc finger protein LSD1 (AHRD V1 **-- Q8W195_BRAOL); contains Interpro domain(s) IPR005735 Zinc finger, LSD1-type " | 2E-55 |
| 987 | Contig 987 | 573 | 1 | GH203442 | Solyc06g082930.2.1 genomic_reference:SL2.40ch06 gene_region:44846738-44850138 transcript_region:SL2.40ch06:44846738..44850138+ functional_description:"FRIGIDA (Fragment) (AHRD V1 *-*- Q58T23_ARATH); contains Interpro domain(s) IPR012474 Frigida-like " | 2E-60 |
| 988 | Contig 988 | 311 | 1 | GH205374 | Solyc02g065570.1.1 evidence_code:10F1H0E1IEG genomic_reference:SL2.40ch02 gene_region:31309646-31309798 transcript_region:SL2.40ch02:31309646..31309798+ functional_description:"DVL1 (AHRD V1 **-- Q6X5V0_ARATH); contains Interpro domain(s) IPR012552 DVL " | 9E-25 |
| 989 | Contig 989 | 288 | 1 | GH205386 | Solyc10g012070.2.1 genomic_reference:SL2.40ch10 gene_region:4403270-4404565 transcript_region:SL2.40ch10:4403270..4404565- functional_description:"Protein BPS1, chloroplastic (AHRD V1 **-- BPS1_ARATH)" | 6E-48 |
| 990 | Contig 990 | 547 | 1 | GH205387 | Solyc06g005060.2.1 genomic_reference:SL2.40ch06 gene_region:36352-38132 transcript_region:SL2.40ch06:36352..38132+ go_terms:GO:0051015 functional_description:"Elongation factor 1-alpha (AHRD V1 ***- Q8H9C0_SOLTU); contains Interpro domain(s) IPR004539 Translation elongation factor EF1A, eukaryotic and archaeal IPR000795 Protein synthesis factor, GTP-binding " | 4E-11 |
| 991 | Contig 991 | 206 | 1 | GH205391 | Solyc02g094220.1.1 evidence_code:10F1H1E1IEG genomic_reference:SL2.40ch02 gene_region:49395545-49395754 transcript_region:SL2.40ch02:49395545..49395754+ functional_description:"Os02g0508100 protein (Fragment) (AHRD V1 ***- Q0E0Z9_ORYSJ)" | 3E-27 |
| 992 | Contig 992 | 531 | 1 | GH203421 | Solyc12g035130.1.1 evidence_code:10F0H1E1IEG genomic_reference:SL2.40ch12 gene_region:23233712-23239726 transcript_region:SL2.40ch12:23233712..23239726+ go_terms:GO:0008026,GO:0005515,GO:0005524 functional_description:"ATP dependent RNA helicase (AHRD V1 ***- B3LNW9_YEAS1); contains Interpro domain(s) IPR011545 DNA/RNA helicase, DEAD/DEAH box type, N-terminal " | 1E-100 |
| 993 | Contig 993 | 588 | 1 | GH203419 | Solyc03g121620.1.1 evidence_code:10F0H1E1IEG genomic_reference:SL2.40ch03 gene_region:63766550-63767353 transcript_region:SL2.40ch03:63766550..63767353+ functional_description:"Harpin-induced protein-like (Fragment) (AHRD V1 **-- D2CFH8_COFAR); contains Interpro domain(s) IPR010847 Harpin-induced 1 " | 6E-85 |
| 994 | Contig 994 | 554 | 1 | GH205394 | Solyc06g007620.2.1 genomic_reference:SL2.40ch06 gene_region:1625363-1627604 transcript_region:SL2.40ch06:1625363..1627604- functional_description:"POT family domain containing protein expressed (AHRD V1 ***- D8L9H8_WHEAT); contains Interpro domain(s) IPR007493 Protein of unknown function DUF538 " | 2E-12 |
| 995 | Contig 995 | 437 | 1 | GH204674 | Solyc06g069020.2.1 genomic_reference:SL2.40ch06 gene_region:39212135-39224003 transcript_region:SL2.40ch06:39212135..39224003+ go_terms:GO:0005525,GO:0006415 functional_description:"Elongation factor 1 alpha (AHRD V1 ***- Q2F6A7_BOMMO); contains Interpro domain(s) IPR000795 Protein synthesis factor, GTP-binding " | 4E-80 |
| 996 | Contig 996 | 691 | 1 | GH203417 | Solyc07g008770.2.1 genomic_reference:SL2.40ch07 gene_region:3751190-3755390 transcript_region:SL2.40ch07:3751190..3755390+ functional_description:"Unknown Protein (AHRD V1)" | 2E-38 |
| 997 | Contig 997 | 336 | 1 | GH205393 | Solyc01g081490.2.1 genomic_reference:SL2.40ch01 gene_region:73199206-73204884 transcript_region:SL2.40ch01:73199206..73204884+ go_terms:GO:0006355 functional_description:"RNA polymerase sigma factor (AHRD V1 *-*- Q59965_SYNP2); contains Interpro domain(s) IPR016262 RNA polymerase sigma factor, SigB/SigC/SigD, plastid " | 9E-48 |
| 998 | Contig 998 | 374 | 1 | GH205310 | Solyc04g074470.1.1 evidence_code:10F0H1E1IEG genomic_reference:SL2.40ch04 gene_region:58047154-58048083 transcript_region:SL2.40ch04:58047154..58048083+ functional_description:"Os06g0220000 protein (Fragment) (AHRD V1 ***- Q0DDJ2_ORYSJ); contains Interpro domain(s) IPR006766 Phosphate-induced protein 1 conserved region " | 5E-69 |
| 999 | Contig 999 | 145 | 1 | GH203529 | ********* |  |
| 1000 | Contig 1000 | 424 | 1 | GH203531 | Solyc01g095400.2.1 genomic_reference:SL2.40ch01 gene_region:78429443-78434370 transcript_region:SL2.40ch01:78429443..78434370+ go_terms:GO:0005515 functional_description:"Kelch-like protein 6 (AHRD V1 *-*- B5X1S7_SALSA); contains Interpro domain(s) IPR013089 Kelch related " | 4E-79 |
| 1001 | Contig 1001 | 407 | 1 | GH205295 | Solyc11g005000.1.1 evidence_code:10F1H0E1IEG genomic_reference:SL2.40ch11 gene_region:8301-11298 transcript_region:SL2.40ch11:8301..11298- functional_description:"Keratinocytes-associated protein 2 (AHRD V1 ***- B6SJ34_MAIZE); contains Interpro domain(s) IPR018614 Protein of unknown function KRTCAP2 " | 6E-40 |
| 1002 | Contig 1002 | 619 | 1 | GH204632 | Solyc04g082330.2.1 genomic_reference:SL2.40ch04 gene_region:63629294-63637973 transcript_region:SL2.40ch04:63629294..63637973- go_terms:GO:0005515 functional_description:"Pre-rRNA-processing protein TSR1 (AHRD V1 *-*- D0MZI1_PHYIN); contains Interpro domain(s) IPR007034 Protein of unknown function DUF663 " | 1E-117 |
| 1003 | Contig 1003 | 762 | 1 | GH205299 | Solyc01g090720.2.1 genomic_reference:SL2.40ch01 gene_region:76114874-76119446 transcript_region:SL2.40ch01:76114874..76119446+ functional_description:"Unknown Protein (AHRD V1)" | 7E-53 |
| 1004 | Contig 1004 | 435 | 1 | GH203490 | Solyc02g067580.2.1 genomic_reference:SL2.40ch02 gene_region:32316480-32318685 transcript_region:SL2.40ch02:32316480..32318685+ functional_description:"B12D-like protein (AHRD V1 ***- D2XQY6_WOLAR); contains Interpro domain(s) IPR010530 B12D " | 1E-48 |
| 1005 | Contig 1005 | 301 | 1 | GH205335 | Solyc01g110300.2.1 genomic_reference:SL2.40ch01 gene_region:88788002-88793586 transcript_region:SL2.40ch01:88788002..88793586- go_terms:GO:0030276 functional_description:"Clathrin binding protein (AHRD V1 **-* B6STF7_MAIZE); contains Interpro domain(s) IPR001452 Src homology-3 domain " | 5E-30 |
| 1006 | Contig 1006 | 234 | 1 | GH205336 | Solyc03g117980.2.1 genomic_reference:SL2.40ch03 gene_region:61025937-61031702 transcript_region:SL2.40ch03:61025937..61031702+ go_terms:GO:0016174 functional_description:"Respiratory burst oxidase-like protein (AHRD V1 **** C1IHQ9_9ROSI); contains Interpro domain(s) IPR013121 Ferric reductase, NAD binding " | 5E-39 |
| 1007 | Contig 1007 | 102 | 1 | GH203481 | ********* |  |
| 1008 | Contig 1008 | 229 | 1 | GH205294 | ********* |  |
| 1009 | Contig 1009 | 260 | 1 | GH203514 | Solyc01g107030.2.1 genomic_reference:SL2.40ch01 gene_region:86465054-86468000 transcript_region:SL2.40ch01:86465054..86468000+ functional_description:"Somatic embryogenesis related protein (AHRD V1 ***- B6TIS5_MAIZE)" | 2E-23 |
| 1010 | Contig 1010 | 385 | 1 | GH203513 | Solyc02g063360.2.1 genomic_reference:SL2.40ch02 gene_region:30004241-30007916 transcript_region:SL2.40ch02:30004241..30007916- functional_description:"C2 domain-containing protein (AHRD V1 **-- D7MIH2_ARALY); contains Interpro domain(s) IPR018029 C2 membrane targeting protein " | 2E-38 |
| 1011 | Contig 1011 | 337 | 1 | GH205305 | Solyc03g114150.2.1 genomic_reference:SL2.40ch03 gene_region:58222807-58227323 transcript_region:SL2.40ch03:58222807..58227323+ go_terms:GO:0004029 functional_description:"Aldehyde dehydrogenase (AHRD V1 **** Q1AFF6_9ROSI); contains Interpro domain(s) IPR015590 Aldehyde dehydrogenase " | 9E-62 |
| 1012 | Contig 1012 | 297 | 1 | GH204633 | ********* |  |
| 1013 | Contig 1013 | 385 | 1 | GH203518 | Solyc01g112290.2.1 genomic_reference:SL2.40ch01 gene_region:90114267-90129540 transcript_region:SL2.40ch01:90114267..90129540+ go_terms:GO:0005515 functional_description:"Glutamyl-tRNA synthetase (AHRD V1 ***- Q52JJ5_NICBE); contains Interpro domain(s) IPR004527 Glutamyl-tRNA synthetase, class Ic, bacterial/mitochondrial " | 2E-71 |
| 1014 | Contig 1014 | 301 | 1 | GH203533 | Solyc01g111170.2.1 genomic_reference:SL2.40ch01 gene_region:89284136-89285146 transcript_region:SL2.40ch01:89284136..89285146+ go_terms:GO:0006457 functional_description:"Peptidyl-prolyl cis-trans isomerase (AHRD V1 ***- Q9XF12_SOLTU); contains Interpro domain(s) IPR002130 Peptidyl-prolyl cis-trans isomerase, cyclophilin-type " | 8E-18 |
| 1015 | Contig 1015 | 574 | 1 | GH203532 | Solyc10g080660.1.1 evidence_code:10F1H1E1IEG genomic_reference:SL2.40ch10 gene_region:61208446-61211985 transcript_region:SL2.40ch10:61208446..61211985- go_terms:GO:0005737 functional_description:"Eukaryotic translation initiation factor 4E (AHRD V1 ***- A6XJR1_PIG); contains Interpro domain(s) IPR001040 Eukaryotic translation initiation factor 4E (eIF-4E) " | 2E-83 |
| 1016 | Contig 1016 | 443 | 1 | GH203515 | Solyc03g118620.2.1 genomic_reference:SL2.40ch03 gene_region:61519525-61527461 transcript_region:SL2.40ch03:61519525..61527461- go_terms:GO:0005515 functional_description:"Leucine-rich repeat family protein (AHRD V1 *--- D7KD26_ARALY)" | 1E-60 |
| 1017 | Contig 1017 | 261 | 1 | GH203526 | Solyc05g009600.2.1 genomic_reference:SL2.40ch05 gene_region:3800023-3807291 transcript_region:SL2.40ch05:3800023..3807291+ go_terms:GO:0017018 functional_description:"Phosphatase 2A regulatory A subunit (AHRD V1 **** Q9S7C5_ORYSA); contains Interpro domain(s) IPR011989 Armadillo-like helical " | 1E-43 |
| 1018 | Contig 1018 | 650 | 1 | GH203466 | Solyc12g056410.1.1 evidence_code:10F0H1E1IEG genomic_reference:SL2.40ch12 gene_region:47699541-47701524 transcript_region:SL2.40ch12:47699541..47701524+ functional_description:"F-box protein PP2-B1 (AHRD V1 ***- PP2B1_ARATH); contains Interpro domain(s) IPR001810 Cyclin-like F-box " | 1E-100 |
| 1019 | Contig 1019 | 934 | 1 | GH203607 | Solyc03g116780.2.1 genomic_reference:SL2.40ch03 gene_region:60110746-60114311 transcript_region:SL2.40ch03:60110746..60114311+ functional_description:"CUE domain containing protein (AHRD V1 **-- B6TCB4_MAIZE); contains Interpro domain(s) IPR009060 UBA-like " | 1E-138 |
| 1020 | Contig 1020 | 691 | 1 | GH205241 | Solyc05g013990.2.1 genomic_reference:SL2.40ch05 gene_region:7492470-7499923 transcript_region:SL2.40ch05:7492470..7499923+ go_terms:GO:0051082 functional_description:"T-complex protein 1 subunit epsilon (AHRD V1 ***- B6TSC6_MAIZE); contains Interpro domain(s) IPR012718 T-complex protein 1, epsilon subunit " | 1E-118 |
| 1021 | Contig 1021 | 414 | 1 | GH205249 | Solyc05g052570.2.1 genomic_reference:SL2.40ch05 gene_region:61926638-61928431 transcript_region:SL2.40ch05:61926638..61928431- go_terms:GO:0008270 functional_description:"Zinc finger CCCH domain-containing protein 66 (AHRD V1 ***- C3H66_ARATH); contains Interpro domain(s) IPR002110 Ankyrin " | 2E-61 |
| 1022 | Contig 1022 | 271 | 1 | GH203608 | Solyc08g063070.2.1 genomic_reference:SL2.40ch08 gene_region:49834397-49841552 transcript_region:SL2.40ch08:49834397..49841552+ go_terms:GO:0003824 functional_description:"Esterase/lipase/thioesterase (Fragment) (AHRD V1 **-- B3VW20_POPTN); contains Interpro domain(s) IPR000073 Alpha/beta hydrolase fold-1 " | 1E-19 |
| 1023 | Contig 1023 | 831 | 1 | GH205232 | Solyc07g064810.2.1 genomic_reference:SL2.40ch07 gene_region:64017771-64025505 transcript_region:SL2.40ch07:64017771..64025505- go_terms:GO:0000107 functional_description:"Imidazole glycerol phosphate synthase subunit hisF (AHRD V1 *-** D2RFM3_ARCPA); contains Interpro domain(s) IPR014640 Imidazole glycerol phosphate synthase HisHF " | 1E-145 |
| 1024 | Contig 1024 | 736 | 1 | GH204640 | Solyc04g056270.2.1 genomic_reference:SL2.40ch04 gene_region:53071198-53081628 transcript_region:SL2.40ch04:53071198..53081628- go_terms:GO:0016563 functional_description:"Calmodulin-binding transcription activator 3 (AHRD V1 *--* Q0WW70_ARATH); contains Interpro domain(s) IPR005559 CG-1 " | 1E-119 |
| 1025 | Contig 1025 | 673 | 1 | GH203621 | Solyc06g082370.2.1 genomic_reference:SL2.40ch06 gene_region:44499210-44505243 transcript_region:SL2.40ch06:44499210..44505243+ functional_description:"Os07g0202900 protein (Fragment) (AHRD V1 ***- Q0D7X1_ORYSJ); contains Interpro domain(s) IPR019368 Ribosomal protein S23/S29, mitochondrial " | 8E-87 |
| 1026 | Contig 1026 | 927 | 1 | GH203633 | Solyc07g053280.2.1 genomic_reference:SL2.40ch07 gene_region:59072715-59077834 transcript_region:SL2.40ch07:59072715..59077834- go_terms:GO:0000287,GO:0070402 functional_description:"Ketol-acid reductoisomerase (AHRD V1 ***- D6QSY0_CATRO); contains Interpro domain(s) IPR016206 Ketol-acid reductoisomerase, plant " | 1E-146 |
| 1027 | Contig 1027 | 580 | 1 | GH203642 | Solyc09g075360.2.1 genomic_reference:SL2.40ch09 gene_region:62584484-62587919 transcript_region:SL2.40ch09:62584484..62587919+ go_terms:GO:0008810 functional_description:"Endoglucanase 1 (AHRD V1 ***- B6U0P7_MAIZE); contains Interpro domain(s) IPR008928 Six-hairpin glycosidase-like IPR012341 Six-hairpin glycosidase IPR018221 Glycoside hydrolase, family 9, active site IPR001701 Glycoside hydrolase, family 9 " | 1E-99 |
| 1028 | Contig 1028 | 385 | 1 | GH205248 | Solyc06g083110.1.1 evidence_code:10F0H1E1IEG genomic_reference:SL2.40ch06 gene_region:44952089-44952757 transcript_region:SL2.40ch06:44952089..44952757- go_terms:GO:0016020 functional_description:"Conserved hypothetical membrane protein (AHRD V1 *--- A2C9X4_PROM3); contains Interpro domain(s) IPR003425 Protein of unknown function YGGT " | 5E-70 |
| 1029 | Contig 1029 | 387 | 1 | GH204641 | ********* |  |
| 1030 | Contig 1030 | 627 | 1 | GH203663 | Solyc12g055850.1.1 evidence_code:10F0H1E0IEG genomic_reference:SL2.40ch12 gene_region:47218869-47222331 transcript_region:SL2.40ch12:47218869..47222331+ functional_description:"NC domain-containing protein (AHRD V1 **-- A6MD14_9ORYZ); contains Interpro domain(s) IPR007053 NC " | 4E-85 |
| 1031 | Contig 1031 | 351 | 1 | GH205211 | Solyc06g053400.2.1 genomic_reference:SL2.40ch06 gene_region:32654699-32664611 transcript_region:SL2.40ch06:32654699..32664611+ go_terms:GO:0003852 functional_description:"2-isopropylmalate synthase 1 (AHRD V1 **** Q30DX9_9BRAS); contains Interpro domain(s) IPR005671 Bacterial 2-isopropylmalate synthase " | 6E-63 |
| 1032 | Contig 1032 | 762 | 1 | GH204564 | Solyc01g106390.2.1 genomic_reference:SL2.40ch01 gene_region:86065693-86068605 transcript_region:SL2.40ch01:86065693..86068605- go_terms:GO:0008152,GO:0005737,GO:0055114 functional_description:"Glutamyl-tRNA reductase (AHRD V1 ***- A5BZY3_VITVI); contains Interpro domain(s) IPR000343 Tetrapyrrole biosynthesis, glutamyl-tRNA reductase " | 1E-76 |
| 1033 | Contig 1033 | 292 | 1 | GH203660 | Solyc08g066530.2.1 genomic_reference:SL2.40ch08 gene_region:52464757-52475814 transcript_region:SL2.40ch08:52464757..52475814- go_terms:GO:0003993 functional_description:"Acid phosphatase-like protein (AHRD V1 **-- Q9M0F5_ARATH); contains Interpro domain(s) IPR010028 Acid phosphatase, plant " | 5E-52 |
| 1034 | Contig 1034 | 256 | 1 | GH204566 | Solyc05g010120.2.1 genomic_reference:SL2.40ch05 gene_region:4302127-4308688 transcript_region:SL2.40ch05:4302127..4308688+ go_terms:GO:0004012 functional_description:"Phospholipid-transporting ATPase (AHRD V1 **** C1HB29_PARBA); contains Interpro domain(s) IPR006539 ATPase, P-type, phospholipid-translocating, flippase " | 1E-29 |
| 1035 | Contig 1035 | 557 | 1 | GH205243 | Solyc01g109500.2.1 genomic_reference:SL2.40ch01 gene_region:88187324-88189158 transcript_region:SL2.40ch01:88187324..88189158- functional_description:"BURP domain-containing protein (AHRD V1 *--- B2ZPK5_SOLTU); contains Interpro domain(s) IPR004873 BURP " | 4E-80 |
| 1036 | Contig 1036 | 407 | 1 | GH205244 | Solyc10g077030.1.1 evidence_code:10F1H1E1IEG genomic_reference:SL2.40ch10 gene_region:59270503-59275704 transcript_region:SL2.40ch10:59270503..59275704- go_terms:GO:0019773,GO:0005839 functional_description:"Proteasome subunit alpha type (AHRD V1 ***- Q93X34_TOBAC); contains Interpro domain(s) IPR001353 Proteasome, subunit alpha/beta " | 1E-33 |
| 1037 | Contig 1037 | 360 | 1 | GH203610 | Solyc01g107870.2.1 genomic_reference:SL2.40ch01 gene_region:87021438-87028206 transcript_region:SL2.40ch01:87021438..87028206+ go_terms:GO:0008143 functional_description:"Poly(A) RNA binding protein (AHRD V1 **** B2CJ74_9HYPO); contains Interpro domain(s) IPR006515 Polyadenylate binding protein, human types 1, 2, 3, 4 " | 5E-26 |
| 1038 | Contig 1038 | 810 | 1 | GH203619 | Solyc01g108660.2.1 genomic_reference:SL2.40ch01 gene_region:87650490-87656659 transcript_region:SL2.40ch01:87650490..87656659- go_terms:GO:0008800 functional_description:"N-acetyl-gamma-glutamyl- phosphate reductase (AHRD V1 ***- A7WPL0_TOBAC); contains Interpro domain(s) IPR000706 N-acetyl-gamma-glutamyl-phosphate reductase " | 2E-86 |
| 1039 | Contig 1039 | 429 | 1 | GH205347 | Solyc03g111340.2.1 genomic_reference:SL2.40ch03 gene_region:55998016-56004770 transcript_region:SL2.40ch03:55998016..56004770+ go_terms:GO:0005515,GO:0008641 functional_description:"Ubiquitin-like modifier-activating enzyme 5 (AHRD V1 **-* D3PG49_9MAXI); contains Interpro domain(s) IPR009036 Molybdenum cofactor biosynthesis, MoeB " | 2E-77 |
| 1040 | Contig 1040 | 293 | 1 | GH203468 | Solyc03g082590.2.1 genomic_reference:SL2.40ch03 gene_region:46063003-46070268 transcript_region:SL2.40ch03:46063003..46070268+ go_terms:GO:0005622 functional_description:"TBC1 domain family member 15 (AHRD V1 *--- Q6P4X9_XENTR); contains Interpro domain(s) IPR000195 RabGAP/TBC " | 2E-43 |
| 1041 | Contig 1041 | 262 | 1 | GH204579 | ********* |  |
| 1042 | Contig 1042 | 630 | 1 | GH205354 | Solyc02g080370.2.1 genomic_reference:SL2.40ch02 gene_region:39185287-39192371 transcript_region:SL2.40ch02:39185287..39192371+ go_terms:GO:0005515 functional_description:"Tobamovirus multiplication protein (Fragment) (AHRD V1 ***- B0ZZ80_ARAHY); contains Interpro domain(s) IPR009457 Protein of unknown function DUF1084 " | 1E-96 |
| 1043 | Contig 1043 | 444 | 1 | GH203469 | Solyc09g056390.1.1 evidence_code:10F1H0E1IEG genomic_reference:SL2.40ch09 gene_region:44524229-44524456 transcript_region:SL2.40ch09:44524229..44524456+ functional_description:"Unknown Protein (AHRD V1)" | 1E-36 |
| 1044 | Contig 1044 | 524 | 1 | GH205353 | Solyc01g008080.2.1 genomic_reference:SL2.40ch01 gene_region:2219639-2224108 transcript_region:SL2.40ch01:2219639..2224108- go_terms:GO:0003735 functional_description:"Ribosomal protein S27 (AHRD V1 ***- Q3HVK9_SOLTU); contains Interpro domain(s) IPR000592 Ribosomal protein S27e " | 9E-47 |
| 1045 | Contig 1045 | 396 | 1 | GH203467 | Solyc07g006640.2.1 genomic_reference:SL2.40ch07 gene_region:1506417-1517122 transcript_region:SL2.40ch07:1506417..1517122+ go_terms:GO:0005525,GO:0005622 functional_description:"ADP-ribosylation factor 3 (AHRD V1 ***- B4FM64_MAIZE); contains Interpro domain(s) IPR006688 ADP-ribosylation factor " | 5E-65 |
| 1046 | Contig 1046 | 215 | 1 | GH205346 | Solyc11g073250.1.1 evidence_code:10F1H1E1IEG genomic_reference:SL2.40ch11 gene_region:53355429-53355963 transcript_region:SL2.40ch11:53355429..53355963- go_terms:GO:0003684 functional_description:"Histone H2A (AHRD V1 ***- C6TMV8_SOYBN); contains Interpro domain(s) IPR002119 Histone H2A " | 4E-16 |
| 1047 | Contig 1047 | 179 | 1 | GH203464 | Solyc01g109110.2.1 genomic_reference:SL2.40ch01 gene_region:87904937-87909600 transcript_region:SL2.40ch01:87904937..87909600- go_terms:GO:0007186,GO:0007165 functional_description:"Guanine nucleotide-binding protein alpha-1 subunit (AHRD V1 *-*- B6TWS6_MAIZE); contains Interpro domain(s) IPR001019 Guanine nucleotide binding protein (G-protein), alpha subunit " | 2E-28 |
| 1048 | Contig 1048 | 438 | 1 | GH203457 | Solyc12g009650.1.1 evidence_code:10F0H1E1IEG genomic_reference:SL2.40ch12 gene_region:2922153-2923552 transcript_region:SL2.40ch12:2922153..2923552- go_terms:GO:0005199,GO:0006869 functional_description:"Proline rich protein (Fragment) (AHRD V1 *--- P93274_MALDO); contains Interpro domain(s) IPR013770 Plant lipid transfer protein and hydrophobic protein, helical " | 3E-14 |
| 1049 | Contig 1049 | 170 | 1 | GH203534 | Solyc07g064620.1.1 evidence_code:10F1H1E1IEG genomic_reference:SL2.40ch07 gene_region:63909140-63909478 transcript_region:SL2.40ch07:63909140..63909478- go_terms:GO:0005515,GO:0003743 functional_description:"Translation initiation factor SUI1 (AHRD V1 **** A4QR10_MAGGR); contains Interpro domain(s) IPR005874 Eukaryotic translation initiation factor SUI1 " | 7E-19 |
| 1050 | Contig 1050 | 364 | 1 | GH203460 | Solyc03g117980.2.1 genomic_reference:SL2.40ch03 gene_region:61025937-61031702 transcript_region:SL2.40ch03:61025937..61031702+ go_terms:GO:0016174 functional_description:"Respiratory burst oxidase-like protein (AHRD V1 **** C1IHQ9_9ROSI); contains Interpro domain(s) IPR013121 Ferric reductase, NAD binding " | 8E-50 |
| 1051 | Contig 1051 | 644 | 1 | GH205236 | Solyc07g066610.2.1 genomic_reference:SL2.40ch07 gene_region:65209792-65212260 transcript_region:SL2.40ch07:65209792..65212260- go_terms:GO:0005524 functional_description:"Phosphoglycerate kinase (AHRD V1 ***- O81394_SOLTU); contains Interpro domain(s) IPR001576 Phosphoglycerate kinase " | 1E-105 |
| 1052 | Contig 1052 | 121 | 1 | GH205237 | Solyc02g068770.2.1 genomic_reference:SL2.40ch02 gene_region:33264577-33268399 transcript_region:SL2.40ch02:33264577..33268399+ go_terms:GO:0005840,GO:0015934 functional_description:"50S ribosomal protein L30 (AHRD V1 --*- C0B6M9_9FIRM); contains Interpro domain(s) IPR005996 Ribosomal protein L30, bacterial-type " | 3E-17 |
| 1053 | Contig 1053 | 172 | 1 | GH203459 | Solyc09g064370.2.1 genomic_reference:SL2.40ch09 gene_region:57150099-57154745 transcript_region:SL2.40ch09:57150099..57154745+ go_terms:GO:0004024 functional_description:"Alcohol dehydrogenase (AHRD V1 **** Q1HQD0_BOMMO); contains Interpro domain(s) IPR014183 Alcohol dehydrogenase class III/S-(hydroxymethyl)glutathione dehydrogenase " | 1E-21 |
| 1054 | Contig 1054 | 369 | 1 | GH205368 | Solyc10g085850.1.1 evidence_code:10F1H1E1IEG genomic_reference:SL2.40ch10 gene_region:64228620-64228796 transcript_region:SL2.40ch10:64228620..64228796+ functional_description:"TPSI1 (AHRD V1 ***- Q41336_SOLLC)" | 6E-29 |
| 1055 | Contig 1055 | 366 | 1 | GH203455 | Solyc01g110520.2.1 genomic_reference:SL2.40ch01 gene_region:88954731-88956779 transcript_region:SL2.40ch01:88954731..88956779- go_terms:GO:0004044 functional_description:"Glucosamine--fructose-6- phosphate aminotransferase (AHRD V1 *-*- GLMS_METKA); contains Interpro domain(s) IPR005854 Amidophosphoribosyl transferase " | 1E-42 |
| 1056 | Contig 1056 | 303 | 1 | GH203463 | Solyc03g116100.2.1 genomic_reference:SL2.40ch03 gene_region:59662688-59664351 transcript_region:SL2.40ch03:59662688..59664351- go_terms:GO:0003700 functional_description:"MYB transcription factor (AHRD V1 **-* Q6R036_ARATH); contains Interpro domain(s) IPR015495 Myb transcription factor " | 2E-37 |
| 1057 | Contig 1057 | 379 | 1 | GH203461 | Solyc03g115980.1.1 evidence_code:10F0H1E1IEG genomic_reference:SL2.40ch03 gene_region:59589011-59590405 transcript_region:SL2.40ch03:59589011..59590405+ go_terms:GO:0005515 functional_description:"Geranylgeranyl reductase (AHRD V1 ***- Q1ZYL0_OLEEU); contains Interpro domain(s) IPR011774 Geranylgeranyl reductase, plants and cyanobacteria " | 5E-54 |
| 1058 | Contig 1058 | 532 | 1 | GH204626 | Solyc02g062160.1.1 evidence_code:10F0H1E0IEG genomic_reference:SL2.40ch02 gene_region:28313777-28314241 transcript_region:SL2.40ch02:28313777..28314241- go_terms:GO:0031072 functional_description:"Chaperone protein dnaJ 11 (AHRD V1 *-*- B6SS19_MAIZE); contains Interpro domain(s) IPR001623 Heat shock protein DnaJ, N-terminal " | 2E-32 |
| 1059 | Contig 1059 | 187 | 1 | GH203911 | Solyc02g068030.1.1 evidence_code:10F0H0E1IEG genomic_reference:SL2.40ch02 gene_region:32661807-32662649 transcript_region:SL2.40ch02:32661807..32662649- functional_description:"Unknown Protein (AHRD V1)" | 8E-29 |
| 1060 | Contig 1060 | 392 | 1 | GH205095 | Solyc12g010350.1.1 evidence_code:10F0H0E1IEG genomic_reference:SL2.40ch12 gene_region:3407686-3408755 transcript_region:SL2.40ch12:3407686..3408755+ go_terms:GO:0005840 functional_description:"60S ribosomal protein L39 (AHRD V1 ***- D3GC11_9ROSI); contains Interpro domain(s) IPR000077 Ribosomal protein L39e " | 2E-25 |
| 1061 | Contig 1061 | 577 | 1 | GH203909 | Solyc01g101000.2.1 genomic_reference:SL2.40ch01 gene_region:82623105-82629503 transcript_region:SL2.40ch01:82623105..82629503- go_terms:GO:0006468 functional_description:"Kinase family protein (AHRD V1 ***- D7M6L5_ARALY); contains Interpro domain(s) IPR002290 Serine/threonine protein kinase " | 1E-74 |
| 1062 | Contig 1062 | 279 | 1 | GH205099 | ********* |  |
| 1063 | Contig 1063 | 667 | 1 | GH205084 | Solyc07g020860.2.1 genomic_reference:SL2.40ch07 gene_region:14295272-14300912 transcript_region:SL2.40ch07:14295272..14300912+ go_terms:GO:0008379 functional_description:"Peroxiredoxin (AHRD V1 **-- D2D300_GOSHI); contains Interpro domain(s) IPR013740 Redoxin " | 2E-68 |
| 1064 | Contig 1064 | 288 | 1 | GH203926 | Solyc06g007760.2.1 genomic_reference:SL2.40ch06 gene_region:1716214-1719252 transcript_region:SL2.40ch06:1716214..1719252+ functional_description:"Ycf54 protein (AHRD V1 *--- Q8DIT2_THEEB); contains Interpro domain(s) IPR019616 Protein of unknown function DUF2488 " | 2E-18 |
| 1065 | Contig 1065 | 542 | 1 | GH205086 | Solyc07g008320.2.1 genomic_reference:SL2.40ch07 gene_region:3079524-3097721 transcript_region:SL2.40ch07:3079524..3097721- go_terms:GO:0005388 functional_description:"Calcium-transporting ATPase 1 (AHRD V1 **** Q7XBH9_CERRI); contains Interpro domain(s) IPR006408 ATPase, P-type, calcium-transporting, PMCA-type " | 8E-74 |
| 1066 | Contig 1066 | 191 | 1 | GH203924 | Solyc01g073690.2.1 genomic_reference:SL2.40ch01 gene_region:70303278-70304360 transcript_region:SL2.40ch01:70303278..70304360- go_terms:GO:0033178 functional_description:"V-type ATP synthase subunit D (AHRD V1 ***- B0WRC9_CULQU); contains Interpro domain(s) IPR002699 ATPase, V1/A1 complex, subunit D " | 8E-29 |
| 1067 | Contig 1067 | 218 | 1 | GH203914 | Solyc02g082260.2.1 genomic_reference:SL2.40ch02 gene_region:40528275-40531424 transcript_region:SL2.40ch02:40528275..40531424- go_terms:GO:0042282 functional_description:"Hydroxy-methylglutaryl-coenzyme A reductase (AHRD V1 **** O48624_TOBAC); contains Interpro domain(s) IPR004554 Hydroxymethylglutaryl-CoA reductase, class I, catalytic " | 1E-35 |
| 1068 | Contig 1068 | 342 | 1 | GH203804 | Solyc10g079500.1.1 evidence_code:10F1H1E1IEG genomic_reference:SL2.40ch10 gene_region:60360530-60363081 transcript_region:SL2.40ch10:60360530..60363081+ go_terms:GO:0008152,GO:0055114 functional_description:"Inosine-5&apos-monophosphate dehydrogenase (AHRD V1 ***- A7XZM6_CAMSI); contains Interpro domain(s) IPR005990 IMP dehydrogenase " | 9E-59 |
| 1069 | Contig 1069 | 782 | 1 | GH205153 | Solyc08g081940.2.1 genomic_reference:SL2.40ch08 gene_region:62017281-62021205 transcript_region:SL2.40ch08:62017281..62021205+ go_terms:GO:0004675 functional_description:"Receptor like kinase, RLK" | 1E-136 |
| 1070 | Contig 1070 | 519 | 1 | GH204671 | Solyc10g080610.1.1 evidence_code:10F1H1E1IEG genomic_reference:SL2.40ch10 gene_region:61166125-61167159 transcript_region:SL2.40ch10:61166125..61167159+ functional_description:"Kelch-like protein 14 (AHRD V1 ***- B6T7I5_MAIZE); contains Interpro domain(s) IPR015915 Kelch-type beta propeller " | 1E-101 |
| 1071 | Contig 1071 | 573 | 1 | GH205144 | Solyc03g123630.2.1 genomic_reference:SL2.40ch03 gene_region:64478109-64481386 transcript_region:SL2.40ch03:64478109..64481386- go_terms:GO:0005618,GO:0030599 functional_description:"Pectinesterase (AHRD V1 ***- Q564D7_SOLLC); contains Interpro domain(s) IPR000070 Pectinesterase, catalytic " | 7E-91 |
| 1072 | Contig 1072 | 312 | 1 | GH203817 | Solyc06g076820.1.1 evidence_code:10F0H1E1IEG genomic_reference:SL2.40ch06 gene_region:44108043-44109185 transcript_region:SL2.40ch06:44108043..44109185+ go_terms:GO:0003700 functional_description:"Transcription factor (Fragment) (AHRD V1 *-** D6MKQ1_9ASPA); contains Interpro domain(s) IPR007087 Zinc finger, C2H2-type " | 8E-58 |
| 1073 | Contig 1073 | 396 | 1 | GH205096 | Solyc07g065970.1.1 evidence_code:10F1H0E1IEG genomic_reference:SL2.40ch07 gene_region:64762245-64762739 transcript_region:SL2.40ch07:64762245..64762739- go_terms:GO:0031072 functional_description:"Chaperone protein dnaJ 11 (AHRD V1 *-*- B6SS19_MAIZE); contains Interpro domain(s) IPR001623 Heat shock protein DnaJ, N-terminal " | 1E-39 |
| 1074 | Contig 1074 | 181 | 1 | GH203912 | Solyc12g094620.1.1 evidence_code:10F0H1E1IEG genomic_reference:SL2.40ch12 gene_region:63142846-63144894 transcript_region:SL2.40ch12:63142846..63144894+ go_terms:GO:0055114 functional_description:"Catalase (AHRD V1 ***- Q2PYW5_SOLTU); contains Interpro domain(s) IPR018028 Catalase related subgroup " | 2E-31 |
| 1075 | Contig 1075 | 311 | 1 | GH205150 | Solyc12g013550.1.1 evidence_code:10F1H0E1IEG genomic_reference:SL2.40ch12 gene_region:4397934-4402691 transcript_region:SL2.40ch12:4397934..4402691+ functional_description:"Unknown Protein (AHRD V1)" | 1E-24 |
| 1076 | Contig 1076 | 198 | 1 | GH205124 | Solyc11g062130.1.1 evidence_code:10F0H1E1IEG genomic_reference:SL2.40ch11 gene_region:46084210-46085851 transcript_region:SL2.40ch11:46084210..46085851+ go_terms:GO:0016020,GO:0005743 functional_description:"Mitochondrial ADP/ATP carrier proteins (AHRD V1 ***- Q2UU95_ASPOR); contains Interpro domain(s) IPR002113 Adenine nucleotide translocator 1 " | 9E-17 |
| 1077 | Contig 1077 | 795 | 1 | GH205090 | Solyc05g010420.1.1 evidence_code:10F1H1E1IEG genomic_reference:SL2.40ch05 gene_region:4654381-4655463 transcript_region:SL2.40ch05:4654381..4655463- go_terms:GO:0004014 functional_description:"S-adenosylmethionine decarboxylase proenzyme (AHRD V1 **** Q7XZQ9_VITVI); contains Interpro domain(s) IPR018167 S-adenosylmethionine decarboxylase subgroup IPR001985 S-adenosylmethionine decarboxylase " | 1E-119 |
| 1078 | Contig 1078 | 233 | 1 | GH203894 | Solyc03g025340.1.1 evidence_code:10F1H1E1IEG genomic_reference:SL2.40ch03 gene_region:7153156-7154205 transcript_region:SL2.40ch03:7153156..7154205- functional_description:"C2 domain-containing protein (AHRD V1 *--- Q5DVL6_HORVD); contains Interpro domain(s) IPR018029 C2 membrane targeting protein " | 1E-25 |
| 1079 | Contig 1079 | 470 | 1 | GH203905 | Solyc05g056460.2.1 genomic_reference:SL2.40ch05 gene_region:64888777-64892027 transcript_region:SL2.40ch05:64888777..64892027- functional_description:"Unknown Protein (AHRD V1)" | 3E-23 |
| 1080 | Contig 1080 | 327 | 1 | GH203872 | Solyc08g078700.2.1 genomic_reference:SL2.40ch08 gene_region:59635844-59637072 transcript_region:SL2.40ch08:59635844..59637072+ functional_description:"Heat shock protein 22 (AHRD V1 ***- Q27JQ0_METAN); contains Interpro domain(s) IPR002068 Heat shock protein Hsp20 " | 8E-21 |
| 1081 | Contig 1081 | 372 | 1 | GH203898 | Solyc11g011220.1.1 evidence_code:10F1H1E1IEG genomic_reference:SL2.40ch11 gene_region:4270031-4275016 transcript_region:SL2.40ch11:4270031..4275016- functional_description:"Unknown Protein (AHRD V1)" | 4E-38 |
| 1082 | Contig 1082 | 446 | 1 | GH203903 | Solyc04g008290.2.1 genomic_reference:SL2.40ch04 gene_region:1974768-1980633 transcript_region:SL2.40ch04:1974768..1980633+ go_terms:GO:0008017 functional_description:"Microtubule-associated protein MAP65-1a (AHRD V1 **** C0JA45_ORYPU); contains Interpro domain(s) IPR007145 MAP65/ASE1 " | 1E-50 |
| 1083 | Contig 1083 | 460 | 1 | GH203737 | Solyc01g090230.2.1 genomic_reference:SL2.40ch01 gene_region:75636426-75660453 transcript_region:SL2.40ch01:75636426..75660453- functional_description:"GRAM domain containing protein (AHRD V1 *-*- B4FWB9_MAIZE); contains Interpro domain(s) IPR004182 GRAM " | 1E-54 |
| 1084 | Contig 1084 | 686 | 1 | GH205109 | Solyc11g006660.1.1 evidence_code:10F0H1E1IEG genomic_reference:SL2.40ch11 gene_region:1269001-1270314 transcript_region:SL2.40ch11:1269001..1270314+ go_terms:GO:0005737 functional_description:"Eukaryotic peptide chain release factor subunit 1-3-like (AHRD V1 ***- Q2VCK6_SOLTU); contains Interpro domain(s) IPR004403 Peptide chain release factor eRF/aRF subunit 1 " | 1E-127 |
| 1085 | Contig 1085 | 512 | 1 | GH203890 | Solyc05g009870.2.1 genomic_reference:SL2.40ch05 gene_region:4076564-4086234 transcript_region:SL2.40ch05:4076564..4086234- go_terms:GO:0008270 functional_description:"Baculoviral IAP repeat-containing 8 (AHRD V1 *--- Q6PIA0_HUMAN); contains Interpro domain(s) IPR019396 Transmembrane Fragile-X-F-associated protein " | 7E-25 |
| 1086 | Contig 1086 | 673 | 1 | GH203889 | Solyc04g005040.1.1 evidence_code:10F0H1E1IEG genomic_reference:SL2.40ch04 gene_region:44947-46038 transcript_region:SL2.40ch04:44947..46038+ go_terms:GO:0004222 functional_description:"Matrix metalloproteinase (AHRD V1 **-- B7TVN4_PINTA); contains Interpro domain(s) IPR001818 Peptidase M10A and M12B, matrixin and adamalysin " | 1E-127 |
| 1087 | Contig 1087 | 393 | 1 | GH203873 | Solyc03g119790.2.1 genomic_reference:SL2.40ch03 gene_region:62415402-62423234 transcript_region:SL2.40ch03:62415402..62423234+ go_terms:GO:0006367 functional_description:"Transcription initiation factor IIE alpha subunit family protein (AHRD V1 ***- D7KCD4_ARALY); contains Interpro domain(s) IPR002853 Transcription factor TFIIE, alpha subunit " | 2E-71 |
| 1088 | Contig 1088 | 548 | 1 | GH205089 | Solyc02g081330.2.1 genomic_reference:SL2.40ch02 gene_region:39913208-39917007 transcript_region:SL2.40ch02:39913208..39917007+ go_terms:GO:0046905,GO:0016767 functional_description:"Phytoene synthase 2" | 1E-105 |
| 1089 | Contig 1089 | 214 | 1 | GH204694 | ********* |  |
| 1090 | Contig 1090 | 345 | 1 | GH205092 | Solyc02g022930.2.1 genomic_reference:SL2.40ch02 gene_region:15135802-15149888 transcript_region:SL2.40ch02:15135802..15149888- go_terms:GO:0008679 functional_description:"3-hydroxyisobutyrate dehydrogenase-like protein (AHRD V1 ***- Q1ECP8_ARATH); contains Interpro domain(s) IPR011548 3-hydroxyisobutyrate dehydrogenase " | 9E-25 |
| 1091 | Contig 1091 | 482 | 1 | GH203922 | Solyc05g052570.2.1 genomic_reference:SL2.40ch05 gene_region:61926638-61928431 transcript_region:SL2.40ch05:61926638..61928431- go_terms:GO:0008270 functional_description:"Zinc finger CCCH domain-containing protein 66 (AHRD V1 ***- C3H66_ARATH); contains Interpro domain(s) IPR002110 Ankyrin " | 1E-82 |
| 1092 | Contig 1092 | 878 | 1 | GH203874 | Solyc08g022240.1.1 evidence_code:10F0H1E1IEG genomic_reference:SL2.40ch08 gene_region:14568792-14570240 transcript_region:SL2.40ch08:14568792..14570240+ go_terms:GO:0047714,GO:0008970 functional_description:"Lipase-like protein (AHRD V1 ***- Q8LF19_ARATH); contains Interpro domain(s) IPR002921 Lipase, class 3 " | 1E-165 |
| 1093 | Contig 1093 | 764 | 1 | GH203870 | Solyc04g080960.2.1 genomic_reference:SL2.40ch04 gene_region:62610006-62612431 transcript_region:SL2.40ch04:62610006..62612431+ go_terms:GO:0004197 functional_description:"Cysteine proteinase cathepsin F (AHRD V1 **-* D3TN89_GLOMM); contains Interpro domain(s) IPR013128 Peptidase C1A, papain " | 1E-123 |
| 1094 | Contig 1094 | 134 | 1 | GH203869 | ********* |  |
| 1095 | Contig 1095 | 139 | 1 | GH203882 | Solyc08g076530.2.1 genomic_reference:SL2.40ch08 gene_region:57680356-57685856 transcript_region:SL2.40ch08:57680356..57685856- go_terms:GO:0003676,GO:0000166,GO:0055114 functional_description:"RNA recognition motif-containing protein (AHRD V1 **-- D7M9G3_ARALY); contains Interpro domain(s) IPR001395 Aldo/keto reductase " | 3E-19 |
| 1096 | Contig 1096 | 652 | 1 | GH203879 | Solyc05g051660.1.1 evidence_code:10F0H1E1IEG genomic_reference:SL2.40ch05 gene_region:61195972-61196940 transcript_region:SL2.40ch05:61195972..61196940+ go_terms:GO:0004091 functional_description:"Gibberellin receptor GID1L2 (AHRD V1 **-- B6T2M3_MAIZE); contains Interpro domain(s) IPR013094 Alpha/beta hydrolase fold-3 " | 1E-115 |
| 1097 | Contig 1097 | 190 | 1 | GH203820 | Solyc01g011000.2.1 genomic_reference:SL2.40ch01 gene_region:6848050-6851207 transcript_region:SL2.40ch01:6848050..6851207- go_terms:GO:0003743,GO:0047485 functional_description:"Eukaryotic translation initiation factor 5A (AHRD V1 **** D6N3G5_GOSHI); contains Interpro domain(s) IPR001884 Translation elongation factor, IF5A " | 3E-33 |
| 1098 | Contig 1098 | 123 | 1 | GH204571 | ********* |  |
| 1099 | Contig 1099 | 548 | 1 | GH205209 | Solyc02g068770.2.1 genomic_reference:SL2.40ch02 gene_region:33264577-33268399 transcript_region:SL2.40ch02:33264577..33268399+ go_terms:GO:0005840,GO:0015934 functional_description:"50S ribosomal protein L30 (AHRD V1 --*- C0B6M9_9FIRM); contains Interpro domain(s) IPR005996 Ribosomal protein L30, bacterial-type " | 6E-12 |
| 1100 | Contig 1100 | 462 | 1 | GH205328 | Solyc02g065170.2.1 genomic_reference:SL2.40ch02 gene_region:30910851-30914781 transcript_region:SL2.40ch02:30910851..30914781+ go_terms:GO:0055114 functional_description:"Laccase-22 (AHRD V1 **-- LAC22_ORYSJ); contains Interpro domain(s) IPR001117 Multicopper oxidase, type 1 " | 4E-88 |
| 1101 | Contig 1101 | 587 | 1 | GH203503 | Solyc11g051160.1.1 evidence_code:10F0H1E1IEG genomic_reference:SL2.40ch11 gene_region:42742933-42744970 transcript_region:SL2.40ch11:42742933..42744970+ functional_description:"Phosphatidylinositol transfer protein SFH5 (AHRD V1 *--- C5JFB2_AJEDS); contains Interpro domain(s) IPR001251 Cellular retinaldehyde-binding/triple function, C-terminal " | 1E-112 |
| 1102 | Contig 1102 | 655 | 1 | GH203840 | Solyc04g051370.2.1 genomic_reference:SL2.40ch04 gene_region:49587657-49596538 transcript_region:SL2.40ch04:49587657..49596538- go_terms:GO:0005515,GO:0004175 functional_description:"26S proteasome non-ATPase regulatory subunit 12 (AHRD V1 ***- B5X386_SALSA); contains Interpro domain(s) IPR000717 Proteasome component region PCI " | 6E-98 |
| 1103 | Contig 1103 | 785 | 1 | GH205135 | Solyc02g082120.2.1 genomic_reference:SL2.40ch02 gene_region:40419675-40422095 transcript_region:SL2.40ch02:40419675..40422095+ go_terms:GO:0006284,GO:0006281 functional_description:"DNA-3-methyladenine glycosylase I (AHRD V1 ***- B6TPB7_MAIZE); contains Interpro domain(s) IPR005019 Methyladenine glycosylase " | 2E-62 |
| 1104 | Contig 1104 | 443 | 1 | GH203837 | Solyc06g050980.2.1 genomic_reference:SL2.40ch06 gene_region:30550363-30552503 transcript_region:SL2.40ch06:30550363..30552503+ go_terms:GO:0008198,GO:0042802 functional_description:"Ferritin (AHRD V1 ***- Q308A9_SOLTU); contains Interpro domain(s) IPR001519 Ferritin, N-terminal " | 1E-72 |
| 1105 | Contig 1105 | 138 | 1 | GH203839 | Solyc05g005690.1.1 evidence_code:10F1H1E1IEG genomic_reference:SL2.40ch05 gene_region:510033-510476 transcript_region:SL2.40ch05:510033..510476- go_terms:GO:0032183,GO:0003723 functional_description:"30S ribosomal protein S9 (AHRD V1 ***- C5JX04_AJEDS); contains Interpro domain(s) IPR000754 Ribosomal protein S9 " | 1E-18 |
| 1106 | Contig 1106 | 724 | 1 | GH205134 | Solyc09g090580.2.1 genomic_reference:SL2.40ch09 gene_region:65410646-65414029 transcript_region:SL2.40ch09:65410646..65414029- go_terms:GO:0000340 functional_description:"Eukaryotic translation initiation factor 4E-2 (AHRD V1 ***- B6UD29_MAIZE); contains Interpro domain(s) IPR001040 Eukaryotic translation initiation factor 4E (eIF-4E) " | 1E-115 |
| 1107 | Contig 1107 | 178 | 1 | GH203495 | ********* |  |
| 1108 | Contig 1108 | 488 | 1 | GH205339 | Solyc02g080810.2.1 genomic_reference:SL2.40ch02 gene_region:39515754-39519204 transcript_region:SL2.40ch02:39515754..39519204- go_terms:GO:0004375,GO:0005515,GO:0031405 functional_description:"Aminomethyltransferase (AHRD V1 ***- C6TF02_SOYBN); contains Interpro domain(s) IPR006223 Glycine cleavage system T protein " | 6E-86 |
| 1109 | Contig 1109 | 209 | 1 | GH203477 | Solyc02g032950.2.1 genomic_reference:SL2.40ch02 gene_region:20198127-20200157 transcript_region:SL2.40ch02:20198127..20200157- go_terms:GO:0005515,GO:0010843 functional_description:"Transcription factor WRKY (AHRD V1 ***- C7E5X8_CAPAN); contains Interpro domain(s) IPR003657 DNA-binding WRKY " | 3E-29 |
| 1110 | Contig 1110 | 371 | 1 | GH205341 | Solyc01g103480.2.1 genomic_reference:SL2.40ch01 gene_region:83833887-83841434 transcript_region:SL2.40ch01:83833887..83841434- go_terms:GO:0005515 functional_description:"Coatomer subunit delta (AHRD V1 ***- B6TDR8_MAIZE); contains Interpro domain(s) IPR008968 Clathrin adaptor, mu subunit, C-terminal " | 4E-41 |
| 1111 | Contig 1111 | 255 | 1 | GH203471 | Solyc12g005330.1.1 evidence_code:10F1H1E1IEG genomic_reference:SL2.40ch12 gene_region:208843-210348 transcript_region:SL2.40ch12:208843..210348+ go_terms:GO:0005840 functional_description:"50S ribosomal protein L2 (AHRD V1 ***- RL2_EDWI9); contains Interpro domain(s) IPR002171 Ribosomal protein L2 " | 5E-44 |
| 1112 | Contig 1112 | 604 | 1 | GH203493 | Solyc05g041200.2.1 genomic_reference:SL2.40ch05 gene_region:49398356-49402874 transcript_region:SL2.40ch05:49398356..49402874- go_terms:GO:0003868,GO:0016763 functional_description:"4-hydroxyphenylpyruvate dioxygenase (AHRD V1 **** B7X935_HEVBR); contains Interpro domain(s) IPR005956 4-hydroxyphenylpyruvate dioxygenase " | 1E-113 |
| 1113 | Contig 1113 | 666 | 1 | GH203510 | Solyc01g100590.2.1 genomic_reference:SL2.40ch01 gene_region:82330045-82336272 transcript_region:SL2.40ch01:82330045..82336272- go_terms:GO:0055114 functional_description:"Unknown Protein (AHRD V1); contains Interpro domain(s) IPR019020 Cytochrome c-552/DMSO reductase-like, haem-binding domain " | 3E-95 |
| 1114 | Contig 1114 | 172 | 1 | GH203508 | ********* |  |
| 1115 | Contig 1115 | 139 | 1 | GH203506 | Solyc02g083280.2.1 genomic_reference:SL2.40ch02 gene_region:41305686-41308404 transcript_region:SL2.40ch02:41305686..41308404+ go_terms:GO:0004792 functional_description:"Thiosulfate sulfurtransferase/rhodanese-like domain-containing protein 1 (AHRD V1 *--* TSTD1_HUMAN); contains Interpro domain(s) IPR001763 Rhodanese-like " | 7E-11 |
| 1116 | Contig 1116 | 455 | 1 | GH203505 | Solyc09g061750.1.1 evidence_code:10F0H1E1IEG genomic_reference:SL2.40ch09 gene_region:55888369-55889505 transcript_region:SL2.40ch09:55888369..55889505+ functional_description:"Os07g0419800 protein (Fragment) (AHRD V1 *--- Q0D6Y1_ORYSJ); contains Interpro domain(s) IPR012866 Protein of unknown function DUF1644 " | 1E-35 |
| 1117 | Contig 1117 | 635 | 1 | GH203830 | Solyc05g050980.2.1 genomic_reference:SL2.40ch05 gene_region:60337084-60344001 transcript_region:SL2.40ch05:60337084..60344001+ go_terms:GO:0003866 functional_description:"3-phosphoshikimate 1-carboxyvinyltransferase (AHRD V1 **** Q30CZ8_FAGSY); contains Interpro domain(s) IPR001986 3-phosphoshikimate 1-carboxyvinyltransferase, core " | 1E-111 |
| 1118 | Contig 1118 | 512 | 1 | GH203803 | Solyc11g011960.1.1 evidence_code:10F1H1E1IEG genomic_reference:SL2.40ch11 gene_region:4912805-4919067 transcript_region:SL2.40ch11:4912805..4919067- go_terms:GO:0008152 functional_description:"UTP-glucose 1 phosphate uridylyltransferase (AHRD V1 ***- B3VDY8_EUCGR); contains Interpro domain(s) IPR016267 UTP--glucose-1-phosphate uridylyltransferase, subgroup " | 1E-78 |
| 1119 | Contig 1119 | 443 | 1 | GH203801 | Solyc01g111630.2.1 genomic_reference:SL2.40ch01 gene_region:89609244-89612993 transcript_region:SL2.40ch01:89609244..89612993+ go_terms:GO:0008266,GO:0030267 functional_description:"Glyoxylate/hydroxypyruvate reductase B (AHRD V1 **** GHRB_YERPS); contains Interpro domain(s) IPR006140 D-isomer specific 2-hydroxyacid dehydrogenase, NAD-binding " | 8E-34 |
| 1120 | Contig 1120 | 313 | 1 | GH205156 | Solyc06g082010.2.1 genomic_reference:SL2.40ch06 gene_region:44251649-44254674 transcript_region:SL2.40ch06:44251649..44254674+ go_terms:GO:0003700 functional_description:"Zinc finger CCCH domain-containing protein 66 (AHRD V1 ***- C3H66_ARATH); contains Interpro domain(s) IPR002110 Ankyrin " | 2E-53 |
| 1121 | Contig 1121 | 186 | 1 | GH203799 | Solyc07g054820.2.1 genomic_reference:SL2.40ch07 gene_region:60318652-60325343 transcript_region:SL2.40ch07:60318652..60325343+ go_terms:GO:0005515 functional_description:"Protein thf1 (AHRD V1 ***- A0YRG1_LYNSP)" | 6E-31 |
| 1122 | Contig 1122 | 551 | 1 | GH203795 | Solyc03g113400.2.1 genomic_reference:SL2.40ch03 gene_region:57608874-57615656 transcript_region:SL2.40ch03:57608874..57615656+ go_terms:GO:0008553 functional_description:"H-ATPase (AHRD V1 **** Q8L6I3_ORYSJ); contains Interpro domain(s) IPR006534 ATPase, P-type, plasma-membrane proton-efflux " | 3E-50 |
| 1123 | Contig 1123 | 468 | 1 | GH203797 | Solyc01g006430.2.1 genomic_reference:SL2.40ch01 gene_region:1033539-1035178 transcript_region:SL2.40ch01:1033539..1035178- go_terms:GO:0042389 functional_description:"Omega-6 fatty acid desaturase (AHRD V1 **** Q461Q1_HEVBR); contains Interpro domain(s) IPR005804 Fatty acid desaturase, type 1 " | 7E-92 |
| 1124 | Contig 1124 | 617 | 1 | GH203822 | Solyc04g080570.2.1 genomic_reference:SL2.40ch04 gene_region:62282687-62288606 transcript_region:SL2.40ch04:62282687..62288606+ go_terms:GO:0008415,GO:0004089 functional_description:"2,3,4,5-tetrahydropyridine-2, 6-dicarboxylate N-acetyltransferase (AHRD V1 *--- DAPH_CLOB6); contains Interpro domain(s) IPR011004 Trimeric LpxA-like " | 2E-69 |
| 1125 | Contig 1125 | 399 | 1 | GH203794 | Solyc01g112000.2.1 genomic_reference:SL2.40ch01 gene_region:89896494-89898383 transcript_region:SL2.40ch01:89896494..89898383+ go_terms:GO:0005576 functional_description:"Expansin-like protein (AHRD V1 ***- A7X331_SOLLC); contains Interpro domain(s) IPR007112 Expansin 45, endoglucanase-like IPR007117 Pollen allergen/expansin, C-terminal " | 1E-26 |
| 1126 | Contig 1126 | 256 | 1 | GH203798 | Solyc11g006540.1.1 evidence_code:10F1H1E1IEG genomic_reference:SL2.40ch11 gene_region:1191915-1194459 transcript_region:SL2.40ch11:1191915..1194459- go_terms:GO:0055114 functional_description:"FAD-dependent pyridine nucleotide-disulphide oxidoreductase (AHRD V1 **-- D3M425_9ACTO); contains Interpro domain(s) IPR001327 Pyridine nucleotide-disulphide oxidoreductase, NAD-binding region " | 3E-35 |
| 1127 | Contig 1127 | 391 | 1 | GH203857 | Solyc07g008270.2.1 genomic_reference:SL2.40ch07 gene_region:3030451-3032090 transcript_region:SL2.40ch07:3030451..3032090- go_terms:GO:0005840 functional_description:"50S ribosomal protein L36 (AHRD V1 --*- A6CGQ0_9PLAN); contains Interpro domain(s) IPR000473 Ribosomal protein L36 " | 9E-35 |
| 1128 | Contig 1128 | 508 | 1 | GH203859 | Solyc02g021470.2.1 genomic_reference:SL2.40ch02 gene_region:13552226-13582979 transcript_region:SL2.40ch02:13552226..13582979- go_terms:GO:0030332 functional_description:"Cullin 4 (AHRD V1 ***- A9LK40_SOLLC); contains Interpro domain(s) IPR001373 Cullin, N-terminal " | 1E-71 |
| 1129 | Contig 1129 | 714 | 1 | GH204544 | Solyc02g089820.2.1 genomic_reference:SL2.40ch02 gene_region:46089148-46092029 transcript_region:SL2.40ch02:46089148..46092029- go_terms:GO:0005515 functional_description:"Proteasome maturation protein (AHRD V1 ***- A5HKJ3_RAT); contains Interpro domain(s) IPR008012 Proteasome maturation factor UMP1 " | 1E-55 |
| 1130 | Contig 1130 | 399 | 1 | GH203838 | Solyc01g008960.2.1 genomic_reference:SL2.40ch01 gene_region:2976372-2984961 transcript_region:SL2.40ch01:2976372..2984961+ go_terms:GO:0019899 functional_description:"Argonaute 4-like protein (AHRD V1 ***- D5FQ91_PELHO); contains Interpro domain(s) IPR003165 Stem cell self-renewal protein Piwi " | 2E-34 |
| 1131 | Contig 1131 | 442 | 1 | GH205140 | Solyc03g118020.2.1 genomic_reference:SL2.40ch03 gene_region:61058382-61065557 transcript_region:SL2.40ch03:61058382..61065557- go_terms:GO:0003713,GO:0003723 functional_description:"Tudor / nuclease domain-containing protein (AHRD V1 ***- B6KG97_TOXGO); contains Interpro domain(s) IPR016685 RNA-induced silencing complex, nuclease component Tudor-SN " | 4E-68 |
| 1132 | Contig 1132 | 445 | 1 | GH203843 | Solyc09g057670.2.1 genomic_reference:SL2.40ch09 gene_region:45726675-45731083 transcript_region:SL2.40ch09:45726675..45731083- go_terms:GO:0005528 functional_description:"FK506-binding protein 2 (EC 5.2.1.8)(Peptidyl-prolyl cis-trans isomerase)(PPIase)(Rotamase) (AHRD V1 **-* C8VE36_EMENI); contains Interpro domain(s) IPR001179 Peptidyl-prolyl cis-trans isomerase, FKBP-type " | 1E-37 |
| 1133 | Contig 1133 | 749 | 1 | GH205125 | Solyc02g085570.2.1 genomic_reference:SL2.40ch02 gene_region:42978856-42984886 transcript_region:SL2.40ch02:42978856..42984886+ go_terms:GO:0070742 functional_description:"Splicing factor U2AF large subunit (AHRD V1 ***- D8U7V9_VOLCA); contains Interpro domain(s) IPR006529 U2 snRNP auxilliary factor, large subunit, splicing factor " | 3E-72 |
| 1134 | Contig 1134 | 342 | 1 | GH203858 | Solyc02g089700.2.1 genomic_reference:SL2.40ch02 gene_region:46022095-46023951 transcript_region:SL2.40ch02:46022095..46023951+ functional_description:"Leaf senescence protein-like (AHRD V1 ***- Q8RV74_ORYSJ); contains Interpro domain(s) IPR004253 Protein of unknown function DUF231, plant " | 1E-65 |
| 1135 | Contig 1135 | 110 | 1 | GH203846 | ********* |  |
| 1136 | Contig 1136 | 444 | 1 | GH205127 | Solyc09g098240.2.1 genomic_reference:SL2.40ch09 gene_region:67365058-67377466 transcript_region:SL2.40ch09:67365058..67377466- go_terms:GO:0008536 functional_description:"Importin-7 (Imp7) (Ran-binding protein 7) (RanBP7) (AHRD V1 **** D8LDH6_ECTSI); contains Interpro domain(s) IPR011989 Armadillo-like helical " | 5E-26 |

* In case of contig, GenBank Accession Number of the longest EST is mentioned.
