# Supplementary material for: Induction of Senescence and Identification of Differentially Expressed Genes in Tomato in Response to Monoterpene
Source: PLoS One. 2013 Sep 30;8(9):e76029. doi: 10.1371/journal.pone.0076029 (PMC3786903; doi:10.1371/journal.pone.0076029)
Supplement: Table S3 — List of primers used for quantitative RT-PCR analysis. (DOC) [file pone.0076029.s006.doc]

**Table S3. List of primers used for quantitative RT-PCR analysis.**

| **Sl No** | **Contig ID** | **Target gene** |  | **Primer sequences (5′ to 3′)** |
| --- | --- | --- | --- | --- |
| 1 | Contig 64 (G1) | Aquaporin (GH205009) | F | GGCAGCGCCTAGGCTTCTA |
|  |  |  | R | GGCTCCTCTCCCAATTGGAT |
| 2 | Contig 288 (G2) | DCN1-like protein (GH204140) | F | GGGATGTGGCAATTGCTGTT |
|  |  |  | R | ATCCCGTGAAATAGCCCTTGT |
| 3 | Contig 281 (G3) | ATP-dependent Clp protease (GH205151) | F | GTGCACGAGTTGGGCTCAA |
|  |  |  | R | GCCAGTGCCTTTGCCAGTT |
| 4 | Contig 287 (G4) | Monodehydroascorbate reductase (GH204608) | F | TGCCTCGGCTTTTCACAGA |
|  |  |  | R | TCAAACCCAACAGCCACTGTAC |
| 5 | Contig 51 (G5) | Peroxidase (GH203637) | F | TACGATCGCCTAACAAGTTCGA |
|  |  |  | R | GGATGGGTTCCTTGCTTGG |
| 6 | Contig 1 (G6) | Putative senescence-associated protein (GH205039) | F | TCCCAGCTCACGTTCCCTAT |
|  |  |  | R | TTGCTTTTTGATCCTTCGATGTC |
| 7 | Contig 38 (G8) | NAM-like protein (GH203680) | F | CACCGCTACCTCCACATCCT |
|  |  |  | R | ATCGGACGGCAGGTAGAAGA |
| 8 | Contig 301 (G9) | ABC transporter homolog (GH204334) | F | AGTTCCCTCAACCCTATAGCAGTAAG |
|  |  |  | R | AACTATGGCAGACGTTATGGTTTG |
| 9 | Contig 242 (G10) | 14-3-3 protein (GH205206) | F | GAGAAGGTTGCAAAGACAGCAGAT |
|  |  |  | R | AGGCTCTTCTTGCGCCAATT |
| 10 | Contig 77 (G11) | Xyloglucan endotransglucosylase-hydrolase 9 (GH205301) | F | CAATTTCATCTTTGGTTTGATCCA |
|  |  |  | R | ACACTATGCGATGAGGATTCCA |
| 11 | Contig 304 (G12) | Cellulose synthase (GH203512) | F | TTCAGGAGGCAAGCACTTTATG |
|  |  |  | R | CGGCCAACAATTGCATGTT |
| 12 | Contig 133 (G13) | C2H2-type zinc finger protein (GH203536) | F | GGCGTGGGTTCAACAATCAG |
|  |  |  | R | GAACCAAATCCAGGCCAGAA |
| 13 | Contig 368 (G14) | Putative scarecrow protein (GH204307) | F | GCTTCCTTGTTCCACGCTTT |
|  |  |  | R | CCAGCCCTTATCTTGGTGAAAA |
| 14 | Contig 3 (G15) | Heat shock protein 70 (GH205308) | F | CCTCAGATCACCGTGTGCTT |
|  |  |  | R | GGAGAGTCTACCCTTGTCGTTG |
| 15 | Contig 83 (G16) | Cystathionine gamma synthase (GH203522) | F | GGCGGGATGTTCTTATAGAAAGG |
|  |  |  | R | AGGCACTGTGAAGTTGGTTTCA |
| 16 | Contig 150 (G18) | Chitin-inducible gibberellin-responsive protein, GRAS4 (GH205154) | F | TCATTAGCTGTTCTGCTATCATAGTTTG |
|  |  |  | R | TGGTGGATTTGAGGGCTTTG |
| 17 | Contig 414 (G23) | Late embryogenesis (Lea)-like protein (GH204788) | F | AAGCGCCACCAGGGTAATC |
|  |  |  | R | GTGGAGTCATTTGCCTTTATGCT |
| 18 | Contig 221 (G25) | Wound-induced protein (GH205046) | F | CGAAAAACGTTAATGTCCAAATCTAG |
|  |  |  | R | GAGCTCAAACAACGGTCAGAATC |
| 19 | Contig 129 (G27) | Ripening regulated protein DDTFR10-like (GH203604) | F | CTCTGGTGTATCCGGTGAAGGT |
|  |  |  | R | TGAGGCCTTACTGTCAGCAACA |
| 20 | Contig 198 (G28) | Pectinesterase (GH204445) | F | GAACGGATACAACCTTCGTGATG |
|  |  |  | R | AATGTATATCGCGGTCCATGACT |
| 21 | Contig 820 (G30) | Beta-mannosidase (GH203943) | F | GATGGCGAAGCTGAACTTTGA |
|  |  |  | R | AGTTCACTTTACCAGTTCCATTTGG |
| 22 | Contig 78(G32) | Xyloglucan endotransglucosylase-hydrolase 3 (GH205029) | F | GGATATGAAACTCCAATCGATTCA |
|  |  |  | R | GGAATCCACAACGCATCATATTT |
| 23 | Contig 795 (G33) | bZIP DNA-binding protein (GH203992) | F | CCTGAAATTCCAGACACTTTGCT |
|  |  |  | R | GCAGAGGCAGTAATGGGTTGA |
| 24 | Contig 1120 (G34) | Cys-3-His zinc finger protein (GH205156) | F | ACAAACCCTCCTATTGCAATTTG |
|  |  |  | R | TGGCTTCACCCTGCACAA |
| 25 | Contig 817 (G35) | ABA 8'-hydroxylase (GH204050) | F | GGATCCATGTAAGAACACTAGCAGTAGT |
|  |  |  | R | TCACTGACGAACAAATTGCAGAT |
| 26 | Contig 94 (G36) | Auxin-regulated protein (GH205212) | F | TCTTCAACGATTCGCATTCCT |
|  |  |  | R | CCGACGCTTCAACTCAAACA |
| 27 | Contig 792 (G37) | Expansin (GH204001) | F | CTAAGTTGTGGAGCTTGTTTTGAGAT |
|  |  |  | R | GGACTTCCAGGATGACACCATT |
| 28 | Contig 67 (G38) | Plasma membrane intrinsic protein PIP2 (GH204919), aquaporin | F | GGATTTGCTGTTTTCATGGTTCA |
|  |  |  | R | AAGCTCCTTGCTGGGTTAATACC |
| 29 | Contig 642 (G39) | Vitamin B6 biosynthesis protein (GH204679) | F | GCCCTCGCCGACGAA |
|  |  |  | R | CTCGACATCCACAGACAAAAGG |
| 30 | Contig 13 (G40) | Metallothionein-like protein (GH205017) | F | CCGAACCAAACATGTCAAGCT |
|  |  |  | R | CATCCATGATAACAGTCTCCAGGTA |
| 31 | Contig 646 (G41) | Pectinesterase (GH205013) | F | ATCGACTGTGAATGTAACACCAT |
|  |  |  | R | GCAGGAAGTTACCTTTCCCTG |
| 32 | Contig 80 (G43) | Xyloglucan endotransglycosylase-hydrolase 16 (GH203511) | F | AAGGCGGGCTCGTTAAGAC |
|  |  |  | R | GTTAATCTCAAATCCCTTATAGGATGCT |
| 33 | Contig 185 (G44) | BAG-domain protein 1/regulator of cell death (GH204276) | F | CAGCATCAGCGTAGGTATTCCA |
|  |  |  | R | CGCCTGCTGCCGTTCT |
| 34 | Contig 575 (G45) | Ripening-related protein (GH204349) | F | TGAAGGTTTGCAGACAATTGGT |
|  |  |  | R | TCTGGGATCTTTGACATTATTGGA |
| 35 | Contig 531 (G46) | CCR4 associated factor 1-related protein (GH204364) | F | ACCGGCCTGGTGACATTTT |
|  |  |  | R | TTAGACCGGCTTGCGAAGAC |
| 36 | Contig 546 (G47) | Lipoxygenase (GH204408) | F | GGGCCTGTTAAAGTTCCATATACATT |
|  |  |  | R | CACACTATTAGGTATTCCTTTGCCT |
| 37 | Contig 59 (G48) | Transcriptional activator CBF1 (GH204429) | F | TGCTTGTTTGAATTTCTCTGATTCTG |
|  |  |  | R | GGCCGCCTTTTGAATATCTTT |
| 38 | Contig 1088 (G49) | Phytoene synthase 2 (GH205089) | F | CGGCCATTTGATATGCTTGAT |
|  |  |  | R | CCATACGCATTCCTTCAACCA |
| 39 | Contig 1077 (G50) | S-adenosylmethionine decarboxylase (GH205090) | F | TGAACCCTGTGGTTATTCTATGAATT |
|  |  |  | R | TTCAAAGCTGGCATAGCTAAAGC |
| 40 | Contig 1057 (G52) | Geranylgeranyl reductase (GH203461) | F | GGTGGAATTGAGACCTTTTTGATAG |
|  |  |  | R | TCGCCCACCATGCAGAGT |
| 41 | Contig 79 (G53) | Xyloglycan endo-transglycosylase (GH203550) | F | GGAGCCATAGACCAATCTGTCTTC |
|  |  |  | R | TTTACGCGAGTCTATGGAATGC |
| 42 | Contig 888 (G54) | RING/C3HC4/PHD zinc finger-like protein (GH203682) | F | TCCTGTTCTTGCCCCAATTC |
|  |  |  | R | GCTGATCTTACTCCTAAACCTGATGA |
| 43 | Contig 183 (G55) | Transcription factor C2H2 (GH204988) | F | TGATCACCCGACTTTCCATAAA |
|  |  |  | R | ACGAGTGCTGTTTCGACTTCAA |
| 44 | Contig 142 (G56) | Protein farnesyltransferase/ geranylgeranyltransferase (GH204686) | F | CCGCTAAGGTTGAGACCAGAA |
|  |  |  | R | CGGAACCACCGGATTAGGT |
| 45 | Contig 830 (G57) | Pathogenesis related protein P69G, subtilisin-like protease (GH204177) | F | GAGGCATTCTTGTGAGTTGCTC |
|  |  |  | R | GGGCTTCGTTTGATACAGAACTT |
| 46 | Contig 73 (G58) | Lipid-transfer protein (GH204909) | F | GTTACAAGCAGCTTGACGATCA |
|  |  |  | R | GTTATCTTCAAGGTGGTCCTGGT |
| 47 | Contig 317 (G59) | Ethylene-responsive factor 1 (GH203876) | F | CGTAATGGAATTAGGGTTTGGTTAG |
|  |  |  | R | GAGACCAAGGACCCCTCATTG |
| 48 | Contig 639 (G60) | Pathogenesis-related protein PR-5 (GH204834) | F | TTTGGTGGTTTACCCCATCCT |
|  |  |  | R | GGAAGAGGTTGGTGCCAGACT |
| 49 | Contig 663 (G61) | Beta-fructofuranosidase (GH204847) | F | AGTGGTCCGGCATACACACA |
|  |  |  | R | CCCATGCTTCGCCGATTAT |
| 50 | Contig 941 (G63) | Protodermal Factor 1 (GH203862) | F | ACCCTGGCACTCCAAGCA |
|  |  |  | R | GTCCTCCAGTAATCGCATGTAAAA |
| 51 |  | Tomato actin | F | TTATCACCATTGGTGCTGAG |
|  |  |  | R | CGATGTTTCCATACAGATCCTT |

F and R represent forward and reverse primer sequences, respectively.
